# The Transcription Factor ERG Regulates Super-Enhancers Associated With an Endothelial-Specific Gene Expression Program

**DOI:** 10.1161/CIRCRESAHA.118.313788

**Published:** 2019-03-20

**Authors:** Viktoria Kalna, Youwen Yang, Claire R. Peghaire, Karen Frudd, Rebecca Hannah, Aarti V. Shah, Lourdes Osuna Almagro, Joseph J. Boyle, Berthold Göttgens, Jorge Ferrer, Anna M. Randi, Graeme M. Birdsey

**Affiliations:** 1 From the National Heart and Lung Institute (V.K., Y.Y., C.R.P., K.F., A.V.S., L.O.A., J.J.B., A.M.R., G.M.B.), Imperial College London, United Kingdom; 2 Epigenomics and Disease, Department of Medicine (J.F.), Imperial College London, United Kingdom; 3 Department of Haematology, Wellcome Trust and MRC Cambridge Stem Cell Institute, University of Cambridge, United Kingdom (R.H., B.G.).

**Keywords:** chromatin, endothelial cells, endothelium, homeostasis, transcription factors

## Abstract

**Rationale:**

The ETS (E-26 transformation-specific) transcription factor ERG (ETS-related gene) is essential for endothelial homeostasis, driving expression of lineage genes and repressing proinflammatory genes. Loss of ERG expression is associated with diseases including atherosclerosis. ERG’s homeostatic function is lineage-specific, because aberrant ERG expression in cancer is oncogenic. The molecular basis for ERG lineage-specific activity is unknown. Transcriptional regulation of lineage specificity is linked to enhancer clusters (super-enhancers).

**Objective:**

To investigate whether ERG regulates endothelial-specific gene expression via super-enhancers.

**Methods and Results:**

Chromatin immunoprecipitation with high-throughput sequencing in human umbilical vein endothelial cells showed that ERG binds 93% of super-enhancers ranked according to H3K27ac, a mark of active chromatin. These were associated with endothelial genes such as *DLL4* (Delta-like protein 4), *CLDN5* (claudin-5), *VWF* (von Willebrand factor), and *CDH5* (VE-cadherin). Comparison between human umbilical vein endothelial cell and prostate cancer TMPRSS2 (transmembrane protease, serine-2):ERG fusion-positive human prostate epithelial cancer cell line (VCaP) cells revealed distinctive lineage-specific transcriptome and super-enhancer profiles. At a subset of endothelial super-enhancers (including DLL4 and CLDN5), loss of ERG results in significant reduction in gene expression which correlates with decreased enrichment of H3K27ac and MED (Mediator complex subunit)-1, and reduced recruitment of acetyltransferase p300. At these super-enhancers, co-occupancy of GATA2 (GATA-binding protein 2) and AP-1 (activator protein 1) is significantly lower compared with super-enhancers that remained constant following ERG inhibition. These data suggest distinct mechanisms of super-enhancer regulation in endothelial cells and highlight the unique role of ERG in controlling a core subset of super-enhancers. Most disease-associated single nucleotide polymorphisms from genome-wide association studies lie within noncoding regions and perturb transcription factor recognition sequences in relevant cell types. Analysis of genome-wide association studies data shows significant enrichment of risk variants for cardiovascular disease and other diseases, at ERG endothelial enhancers and super-enhancers.

**Conclusions:**

The transcription factor ERG promotes endothelial homeostasis via regulation of lineage-specific enhancers and super-enhancers. Enrichment of cardiovascular disease-associated single nucleotide polymorphisms at ERG super-enhancers suggests that ERG-dependent transcription modulates disease risk.

## Abstract 2

Supplemental Digital Content is available in the text.

Maintenance of endothelial homeostasis is essential for vascular health. Disruption of endothelial homeostasis, as observed in atherosclerosis and in chronic inflammatory diseases, leads to profound changes in the phenotype of endothelial cells (ECs) with upregulation of proinflammatory pathways and loss of anti-inflammatory pathways. Loss of endothelial lineage identity is associated with endothelial-to-mesenchymal transition, a process implicated in multiple diseases.^1^ The transcriptional mechanisms regulating EC lineage identity and maintenance of endothelial homeostasis are also areas of immense interest for vascular regenerative therapies but remain poorly understood.

**Meet the First Author, see p 1278**

The ETS (E-26 transformation-specific) transcription factor (TF) ERG (ETS-related gene) is a critical regulator of endothelial homeostasis (reviewed in Shah et al^2^). In the endothelium, ERG expression appears around developmental day E8.5 and is maintained into adulthood.^3^ ERG is required for endothelial lineage specification,^4^ vascular development, and angiogenesis^5^; endothelial-specific deletion of *Erg* in mouse results in embryonic lethality due to vascular defects.^6,7^ ERG drives expression of lineage-specific genes such as *CDH5* (VE-cadherin), *DLL4* (Delta-like protein 4), *CLDN5* (claudin-5), and *VWF* (von Willebrand factor) and controls processes including survival, permeability, and cytoskeletal dynamics (reviewed in Shah et al^2^). Molecular pathways through which ERG promotes vascular stability and angiogenesis include Wnt/β-catenin signaling^6^ and angiopoietin-1–dependent Notch signaling.^8^ ERG maintains vascular homeostasis also by repressing expression of proinflammatory genes such as *ICAM1* and *IL8*^9,10^ and by protecting from endothelial-to-mesenchymal transition.^11^ In line with its homeostatic role, ERG’s expression is lost in vascular diseases such as the activated endothelium overlying human atherosclerotic plaques.^10^

However, aberrant expression of ERG in non-EC can be detrimental. ERG overexpression as the result of chromosomal translocations in prostate cancer correlates with malignancy and invasiveness, poor prognosis and shorter survival times.^12^ In these circumstances, ERG acts as an oncogene. Since the first report of gene fusions between ERG and the regulatory region of the androgen-dependent TMPRSS-2 (transmembrane protease, serine-2) gene,^13^ the molecular mechanisms through which ERG aberrant expression is oncogenic have been investigated in detail (reviewed in Adamo and Ladomery^14^). The striking difference between the homeostatic versus oncogenic roles of ERG has important implications for the possible therapeutic potential of this pathway in pathologies associated with cardiovascular disease (CVD).

The mechanistic basis for the difference in ERG’s lineage-specific activity may lie in the chromatin landscape. Genetic regulatory elements, called enhancers, play a key role in mediating the transcriptional regulation of lineage-specific gene expression.^15^ Enhancer activation by TFs is cooperative and hierarchical.^15–17^ Members of the ETS, AP-1 (activator protein 1), and GATA families have been shown to functionally interact at enhancers associated with lineage-specific genes.^16^ Recently, clusters of enhancer elements variably termed super-enhancers, stretch enhancers or enhancer clusters, have been described in numerous cell types, including EC.^18–21^ These regulatory elements are associated with an extremely high abundance of TFs, H3K27ac-modified nucleosomes and MED1 (Mediator complex subunit 1) and drive cell type-specific gene expression.^18,19^

Furthermore, super-enhancers are enriched in single nucleotide polymorphisms (SNPs) associated with specific diseases, in a cell type-specific manner.^18^ The majority of SNPs identified by genome-wide association studies (GWASs) associated with human disease traits are localized to noncoding regions of the genome,^22^ including promoters and enhancers, and frequently perturb TF recognition sequences. Thus, SNPs associated with CVD risk may affect TF-binding sites within super-enhancers in EC and other cell types relevant to CVD.

In this study, we show that ERG regulates a subset of endothelial super-enhancers, and that CVD-associated SNPs are enriched at ERG enhancers and super-enhancers. The association of ERG-bound loci with CVD risk variants provides candidate SNPs for future studies on the epigenomic pathways underlying cardiovascular pathologies.

## Methods

All supporting data are available within the article and in the Online Data Supplement. Sequencing data generated in this study (chromatin immunoprecipitation with deep sequencing [ChIP-seq] data sets for ERG, H3K27ac, and MED1 in human umbilical vein endothelial cells [HUVEC]) have been made publicly available at the NCBI Gene Expression Omnibus and can be retrieved through accession number GSE124893.

An expanded Methods section is available in the Online Data Supplement.

## Results

### Integrated Genomic Analysis Reveals an ERG-Regulated Transcriptional Program for the Vascular Endothelium

**Figure 1.**
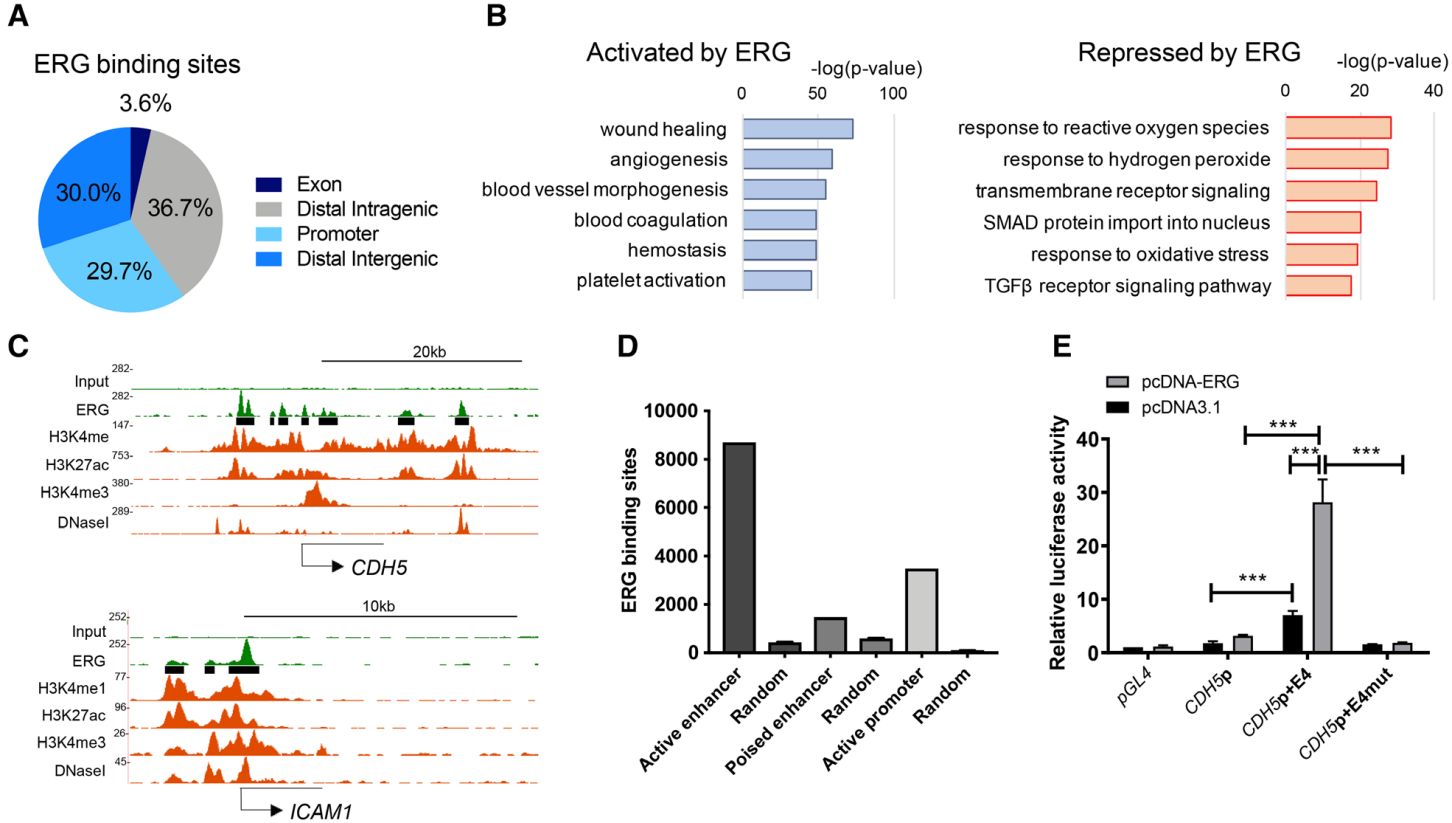
**Characterization of ERG (ETS-related gene)-bound enhancers in human umbilical vein endothelial cells (HUVEC). A**, Percentage genomic distribution of ERG chromatin immunoprecipitation with deep sequencing (ChIP-seq) peaks. **B**, Gene ontology analysis showing pathways associated with differentially regulated ERG-bound endothelial target genes (genes activated in blue and genes repressed in red). Significance shown as –log (*P* value). **C**, ChIP-seq binding profiles for ERG, H3K4me1, H3K27ac, H3K4me3, and DNase I hypersensitivity in HUVEC; loci of ERG-activated *CDH5* (VE-cadherin; **top**) and ERG-repressed *ICAM1* (**bottom**). The *x* axis represents the genomic position; the *y* axis the ChIP-seq signal in reads per million per base pair (rpm/bp). ERG-binding sites are shown as black bars. **D**, Number of ERG-binding sites associated with specific epigenomic features vs size-matched random regions. Active enhancers are defined by combined H3K4me1 and H3K27ac, poised enhancers by H3K4me1 and H3K27me3, and active promoters by H3K4me3 and H3K27ac. **E**, Luciferase reporter plasmids (pGL4) containing the *CDH5* promoter with or without region E4 or a mutant enhancer (E4mut) were cotransfected into HUVEC along with an ERG cDNA expression plasmid (pcDNA-ERG) or empty vector control, pcDNA3.1. Values are represented as the fold change in relative luciferase activity over the empty vector alone. Values are mean±SEM, n=3. A 2-way ANOVA showed significance (*P*<0.001) and a post hoc test using Tukey multiple comparisons test shows pairwise differences between specific groups, ***P<0.001. TGF indicates transforming growth factor.

ChIP-seq analysis in HUVEC identified 40 821 genomic ERG-binding sites associated with 14 786 genes. Selected ERG peaks in the promoters of known ERG target genes *CDH5* and *ICAM1* were validated by ChIP-quantitative polymerase chain reaction (qPCR; Online Figure IA). As expected, the canonical ERG motif (C/a/g)(A/C)GGAA(G/A)^23^ was the most represented at ERG sites in HUVEC (Online Figure IB). Globally, analysis of the distribution of ERG-binding sites relative to annotated transcription start sites (TSSs) revealed that most ERG-bound regions were intragenic and intergenic sites located distally from the promoter (±2 kb from TSS; Figure 1A).

Integrated analysis of global expression profiling^24^ with ERG ChIP-seq showed that ERG binds 85% (1232/1454) of its activated targets and 80% (939/1180) of its repressed targets (Online Figure IC). Gene ontology pathway analysis revealed that directly activated ERG targets clustered in functions related to angiogenesis, blood vessel morphogenesis, and hemostasis, whereas repressed genes associated with TGF (transforming growth factor)-β/SMAD signaling and stress pathways (Figure 1B), in line with its known roles.

### ERG-Bound Enhancers Drive Endothelial Gene Expression

We next examined the relationship between ERG binding and chromatin states in HUVEC using data from the Encyclopedia of DNA Elements Consortium.^25^ Ninety-seven percent of ERG peaks mapped to regions of DNase I hypersensitivity, a marker of accessible open chromatin.^26^ Analysis of known ERG target genes showed ERG genomic loci overlapping histone marks of active promoters (H3K4me3 and H3K27ac) and enhancers (H3K4me1 and H3K27ac) at sites of DNase I hypersensitivity (Figure 1C; Online Figure ID). Globally, ERG binding in HUVEC was greatest at active enhancers (Figure 1D).

ERG-bound enhancers were identified in known ERG targets, including *CDH5*. Four ERG-bound active enhancers, named E1 to E4, were selected based on H3K27ac and H3K4me1 enrichment in a 23 kb region along the *CDH5* locus either side of the TSS (Online Figure IIA). The E1, E2, and E4 enhancers, but not the repeat DNA-containing E3 enhancer, were individually cloned into luciferase reporter vectors containing the *CDH5* promoter, previously characterized as transactivated by ERG.^27^ In HeLa cells (which do not express endogenous ERG), all enhancers increased ERG-dependent transactivation of the *CDH5* promoter, with E4 being the most active (data not shown). This was then validated in HUVEC, where the E4 enhancer increased basal *CDH5* promoter activity >4-fold (Figure 1E); moreover, this region was responsive to ERG transactivation, which further increased luciferase activity by 9-fold compared with *CDH5* promoter alone (Figure 1E). Mutation of the 9 AGGAA putative ERG-binding motifs in region E4 (Online Figure IIB and IIC) completely abolished enhancer activity and the response to ERG (Figure 1E).

These findings support a key role for ERG-mediated transactivation of gene expression through EC enhancers.

### ERG Binds to HUVEC Super-Enhancers

**Figure 2.**
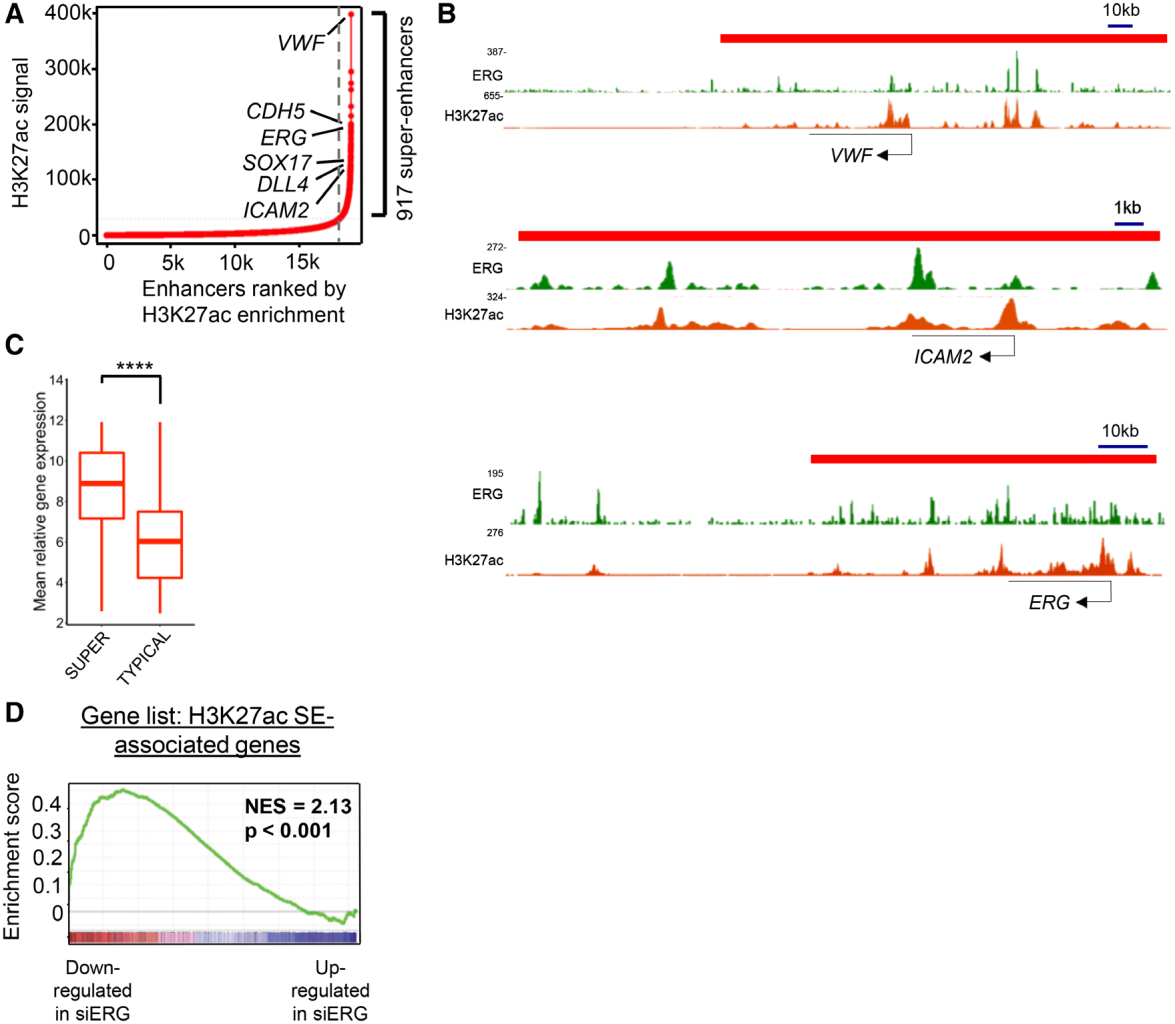
**Characterization of human umbilical vein endothelial cell (HUVEC) super-enhancers (SEs) identifies enrichment for ERG (ETS-related gene) and ERG-target genes. A**, HUVEC enhancer regions identified by H3K27ac and H3K4me1 were ranked by enrichment of H3K27ac chromatin immunoprecipitation with deep sequencing (ChIP-seq) signal in rpm/bp. SE clusters are shown to the right of the gray dashed line (see also Online Table V). **B**, HUVEC SE regions indicated as red bars above density plots of ERG ChIP-seq signal alongside H3K27ac at distal enhancers of *VWF* (von Willebrand factor), *ICAM2*, and *ERG* loci. **C**, Transcriptome profiling of HUVEC shows significantly higher gene expression in SE than in typical enhancers. ***P<0.0001, Wilcoxon rank-sum test. **D**, Gene set enrichment analysis of the top 500 SE-associated genes compared with the ranked gene list from transcriptome profiling of ERG-deficient HUVEC. Normalized enrichment score (NES)=2.13; *P*<0.001.

Multiple ERG-bound enhancers were found in close proximity with each other in ERG-activated genes, such as *CDH5* (see Figure 1C). Clusters of enhancers, known as super-enhancers can be distinguished from isolated typical enhancers by enrichment in lineage-specific TF, coactivators such as MED1 and the histone modification mark H3K27ac.^18,19,28^ Super-enhancers preferentially associate with genes that define cell-lineage identity.^18,19^ To define endothelial super-enhancers, we identified enhancer regions by co-occupancy of H3K27ac and H3K4me1 in HUVEC.^25^ Enhancers within 12.5 kb of each other were then concatenated to define a single entity, and ranked by increasing H3K27ac enrichment, as described.^28^ The analysis identified 917 super-enhancers (Figure 2A; Online Table I) that mapped to 822 genes, including ERG-activated targets *VWF*, *CDH5*, *ICAM2*, *SOX17*, *DLL4*, as well as *ERG* itself (Figure 2A and 2B). A similar super-enhancer profile was obtained when ranked by MED1 enrichment (Online Figure IIIA).

Genes associated with endothelial super-enhancers were found to have significantly higher mean expression levels compared to those associated with typical enhancers (Figure 2C). Gene set enrichment analysis showed significant enrichment of ERG driven genes with the top 500 ranked super-enhancer genes (Figure 2D). These data suggest that ERG regulates endothelial gene expression via super-enhancers.

**Figure 3.**
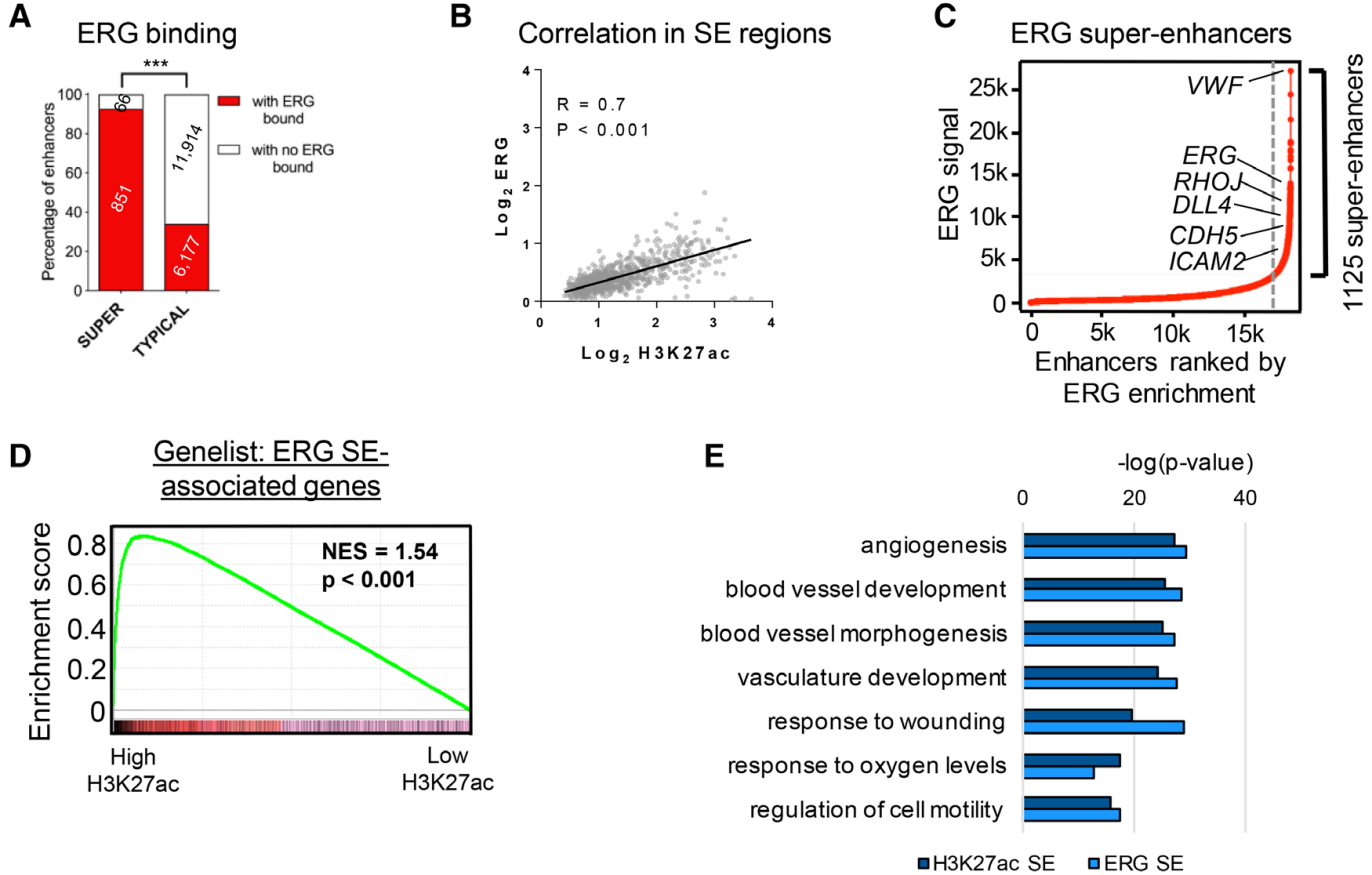
**ERG (ETS-related gene) defines super-enhancers (SEs) in human umbilical vein endothelial cells (HUVEC). A**, ERG binding at SE and typical enhancer regions. SE have significantly more ERG bound than typical enhancers; ***P<0.0001, Fisher exact test. **B**, Scatterplot of the correlation between ERG and H3K27ac occupancy (log_2_ transformed) in the 917 SE regions. Each point is the mean chromatin immunoprecipitation with deep sequencing signal across each SE region; *P*<0.001. **C**, SE ranked by ERG enrichment on identified enhancer regions. Known ERG-target genes proximal to SE are indicated (see also Online Table VI). **D**, Gene set enrichment analysis of the top 500 genes associated with ERG-identified SE compared with the ranked gene list from 917 H3K27ac-enriched SE. Normalized enrichment score (NES)=1.54; *P*<0.001. **E**, Gene ontology analysis was performed on the genes associated with SE identified by enrichment for H3K27ac or ERG. The top 7 biological processes ranked by significance (−log (*P* value)) are depicted.

Remarkably, the vast majority of super-enhancers (93%) were bound by ERG, compared with only 34% of typical enhancers (Figure 3A). In keeping with higher TF occupancy at super-enhancers,^19^ the canonical ERG motif was significantly more bound by ERG in super-enhancers compared with typical enhancers (35% versus 25%, respectively; Online Figure IIIB). Furthermore, within the 917 super-enhancers, H3K27ac and ERG-binding signal significantly correlated (Figure 3B). We thus tested whether ERG itself could be used to identify super-enhancers in EC. Using ERG enrichment at active enhancers as the ranking parameter, we identified 1125 super-enhancers in HUVEC (Figure 3C), associated with a similar gene set as the H3K27ac super-enhancers (see Figure 2A). Indeed, gene set enrichment analysis demonstrated a strong positive correlation between super-enhancers defined by ERG and those by H3K27ac (Figure 3D). Moreover, functional clustering of H3K27ac super-enhancers and ERG super-enhancers revealed shared pathways essential to EC identity and function (Figure 3E).

Thus, ERG binding identifies super-enhancers in differentiated ECs and supports the prominent role of ERG as a lineage-determining TF for the vascular endothelium.

### Differential Super-Enhancer Binding Underlies the Lineage-Specific Activity of ERG

**Figure 4.**
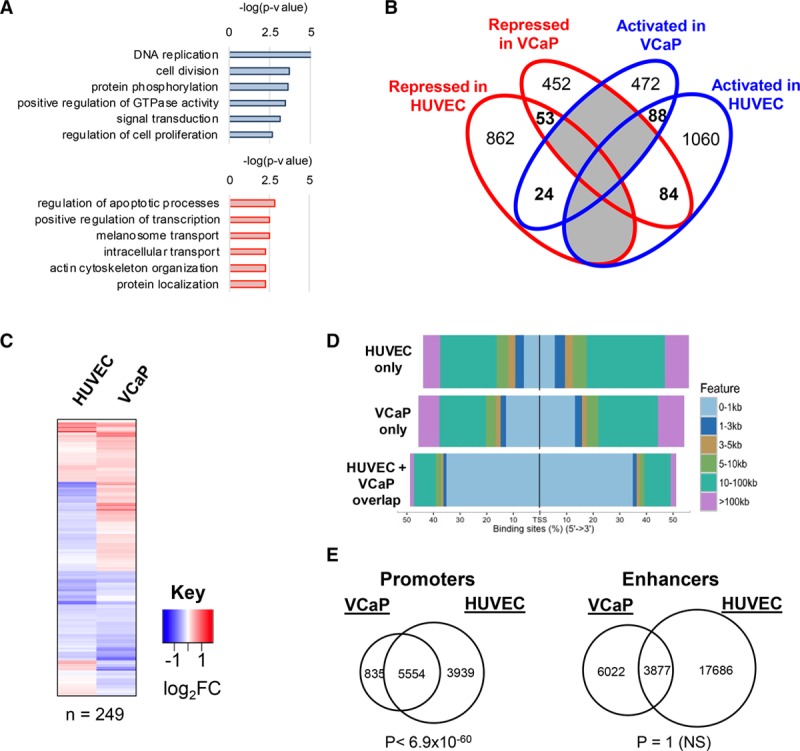
**Chromatin immunoprecipitation with deep sequencing analysis identifies a lineage-specific program for ERG (ETS-related gene): ERG expression and binding profile in prostate cancer cells. A**, Gene ontology pathway analysis of differentially regulated ERG-bound target genes in human prostate epithelial cancer cell line (VCaP) prostate cancer cells (activated genes in blue and repressed genes in red). Significance of pathway enrichment as −log (*P* value). **B**, Venn diagram comparing ERG bound, differentially regulated genes in human umbilical vein endothelial cell (HUVEC) and VCaP cells. Only 249 genes (in bold) are common to the 2 cell types. None of the fractions overlap significantly between the 2-way comparisons using a hypergeometric distribution test. **C**, Heatmap of expression levels of the shared 249 bound and regulated putative ERG target genes following ERG inhibition in HUVEC and in VCaP cells. **D**, Genomic view of percentage distribution of ERG peaks relative to transcription start site (TSS) in regions bound by ERG that are shared between HUVEC and VCaP cells, HUVEC only, or VCaP cells only. **E**, Overlap of ERG-binding sites at H3K4me3-enriched and RefSeq assigned TSS promoter regions (**left**), and H3K27ac/H3K4me1-enriched enhancers (**right**), in HUVEC and VCaP cells. Significance reported by a hypergeometric test *P* value. NS indicates not significant.

At odds with its homeostatic role in EC, aberrant ERG expression due to chromosomal translocations, such as the TMPRSS-2:ERG gene fusions in prostate cells, is a hallmark of cancer (reviewed in Adamo and Ladomery^14^). To exploit ERG’s therapeutic potential in the vasculature, it is crucial to understand the molecular basis of its lineage-specific role. Therefore, we compared ERG-bound gene targets in HUVEC with those from human prostate epithelial cancer cell line (VCaP) prostate cancer cells carrying the TMPRSS-2:ERG gene fusion. Two publicly available ERG ChIP-seq data sets in VCaP cells^29,30^ were found to correlate highly (Online Figure IVA); we used the data in Chng et al^29^ for further analysis. Integration of this ChIP-seq data with transcriptome profiling of ERG-depleted VCaP cells^31^ identified 584 genes bound and activated by ERG and 589 genes bound and repressed by ERG (Online Figure IVB). Gene ontology pathway analysis showed regulation of genes involved in DNA replication, cell proliferation, cytoskeletal remodeling, and apoptosis (Figure 4A). We next examined the overlap between genes directly bound by ERG in VCaP cells versus HUVEC (Online Figure IVC). Interestingly, only 249 ERG-bound target genes (8%) were found in common between HUVEC and VCaP cells (Figure 4B; Online Table II); of these only 58% were activated or repressed concurrently in both cell types (Figure 4C). Interestingly, even pathways controlled by ERG in both HUVEC and VCaP cells (such as cell migration, adhesion, and Notch signaling) are regulated in a lineage-specific manner (Online Figure IVD). Selected ERG transcriptional targets were validated in HUVEC and VCaP cells by RT-qPCR (Online Figure IVE).

**Figure 5.**
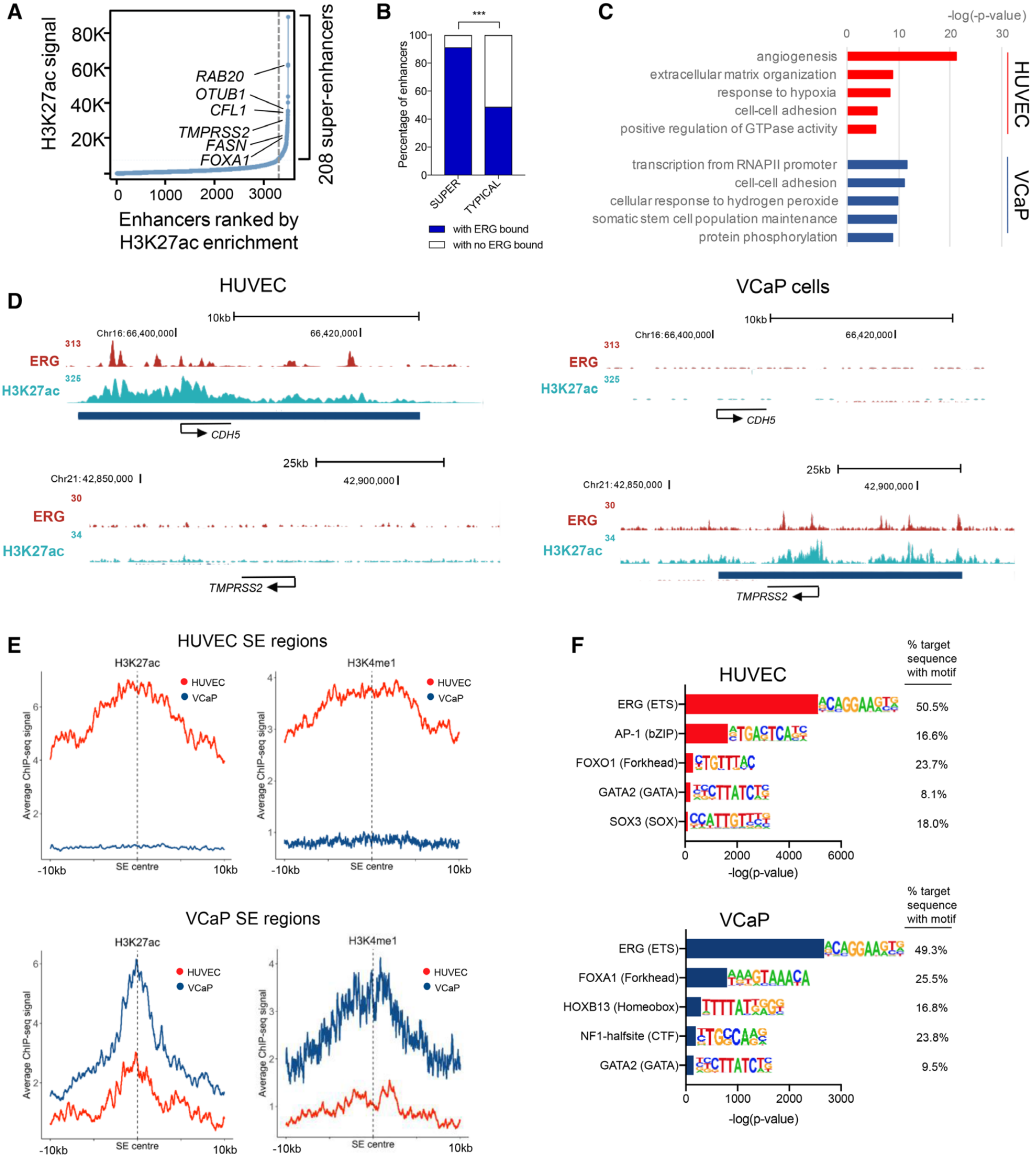
**ERG (ETS-related gene) associates with lineage-specific super-enhancers (SEs). A**, Identification of human prostate epithelial cancer cell line (VCaP) SE by H3K27ac chromatin immunoprecipitation with deep sequencing (ChIP-seq) signal in rpm/bp (see also Online Table VII). **B**, ERG binding at SE and typical enhancers in VCaP cells. SE have significantly more ERG binding than typical enhancers; ***P<0.0001, Fisher exact test. **C**, Gene ontology analysis on the SE-associated genes in human umbilical vein endothelial cell (HUVEC; red) and VCaP cells (blue). The top 5 biological processes in both cells are ranked by significance (−log (*P* value)). **D**, ChIP-seq binding profiles for ERG and H3K27ac occupancy in HUVEC (**left**) and VCaP cells (**right**). H3K27ac-defined SE regions are depicted as blue bars below tracks. **E**, Aggregate plots of active histone modifications H3K27ac and H3K4me1 histone modifications from HUVEC and VCaP cells in HUVEC SE (**top**) and VCaP SE (**bottom**). Plots centered on the SE center. The average size of VCaP SE is smaller than those identified in HUVEC. **F**, Motif analysis at ERG-binding sites (±200 bp) in HUVEC and VCaP. Top 5 most enriched transcription factor families are shown with the most highly occurring member of each family represented. Motifs for the binding of critical lineage transcription factors coincide with the ERG motif in both HUVEC and VCaP cells.

Comparison of ChIP-seq data sets between HUVEC and VCaP cells showed that only 23% of ERG-bound sites are in common between the HUVEC and VCaP genomes (Online Figure IVF). Interestingly, the majority (70%) of these shared sites are located close (±1 kb) to the TSS (Figure 4D). In contrast, the proportion of ERG-binding sites unique to HUVEC or VCaP cells are preferentially located at sites distal to the TSS (Figure 4D). Mapping of ERG genomic binding with histone modification marks confirmed a significant overlap of ERG binding to promoters in HUVEC and VCaP cells (Figure 4E, left). However, no significant overlap was observed between ERG binding at enhancers in HUVEC and VCaP cells, with only 18% of ERG-binding sites found at enhancers in VCaP cells (Figure 4E, right). We next asked whether ERG binding in VCaP cells was also associated with super-enhancers. Using ranked enrichment of H3K27ac from ChIP-seq in VCaP cells,^32^ we identified 208 super-enhancers (Figure 5A). Genes associated with super-enhancers in VCaP had significantly higher average expression levels compared to those associated with isolated typical enhancers (Online Figure VA), as expected. The vast majority of VCaP super-enhancers were bound by ERG (91%), compared with 48% of typical enhancers (Figure 5B). Interestingly, gene set enrichment analysis showed no significant relationship between super-enhancer–associated genes in VCaP cells and HUVEC (Online Figure VB). Gene ontology analysis revealed different pathways regulated by super-enhancer–associated genes in HUVEC compared with VCaP cells (Figure 5C). Finally, super-enhancers occupied by ERG showed cell-lineage specificity: endothelial ERG-regulated genes (such as *CDH5*) are associated with super-enhancers in HUVEC but not VCaP cells, and vice versa for VCaP cell genes (such as *TMPRSS2*; Figure 5D; Online Figure VC).

The difference between super-enhancer profiles in HUVEC versus VCaP cells suggests that the chromatin landscape is unique to the particular cell type. Analysis of histone modifications associated with active (H3K27ac and H3K4me1) or repressed (H3K27me3) chromatin in HUVEC and VCaP super-enhancer regions supports this hypothesis. Genomic regions corresponding to HUVEC super-enhancers were enriched in active histone marks in HUVEC but not VCaP cells (Figure 5E, top). Conversely, genomic regions corresponding to VCaP super-enhancers were enriched in active marks in VCaP cells but not HUVEC (Figure 5E, bottom). The reverse pattern was observed for the repressive mark H3K27me3 (Online Figure VD).

To further define the lineage-specific machinery of super-enhancers, we focused on TFs that may collaborate with ERG to regulate gene transcription in a cell type-specific manner. We searched for TF DNA motifs located ±200 bp from the ERG-binding sites in HUVEC and VCaP cells. Interestingly, different TF motifs are enriched in ERG-binding sites in the 2 cell types. In HUVEC, these include AP-1, FOXO-1 (forkhead box O1), GATA-2 (GATA-binding protein 2), and SOX-3 (SRY-box 3), TFs known to be important in endothelial gene expression^16,33^ (Figure 5F, top). In VCaP cells, the enriched motifs included TFs FOXA-1 (forkhead box A1) and HOXB-13 (homeobox B13), TFs previously described to play a role in prostate cancer gene expression^34^ (Figure 5F, bottom).

These data indicate that ERG’s lineage-specific transcriptional activity is associated with binding to cell type-specific super-enhancers and suggests cooperativity with distinct lineage-specific factors.

### ERG Controls the Gene Expression Profile in HUVEC by Regulating the Enhancer and Super-Enhancer Landscape

**Figure 6.**
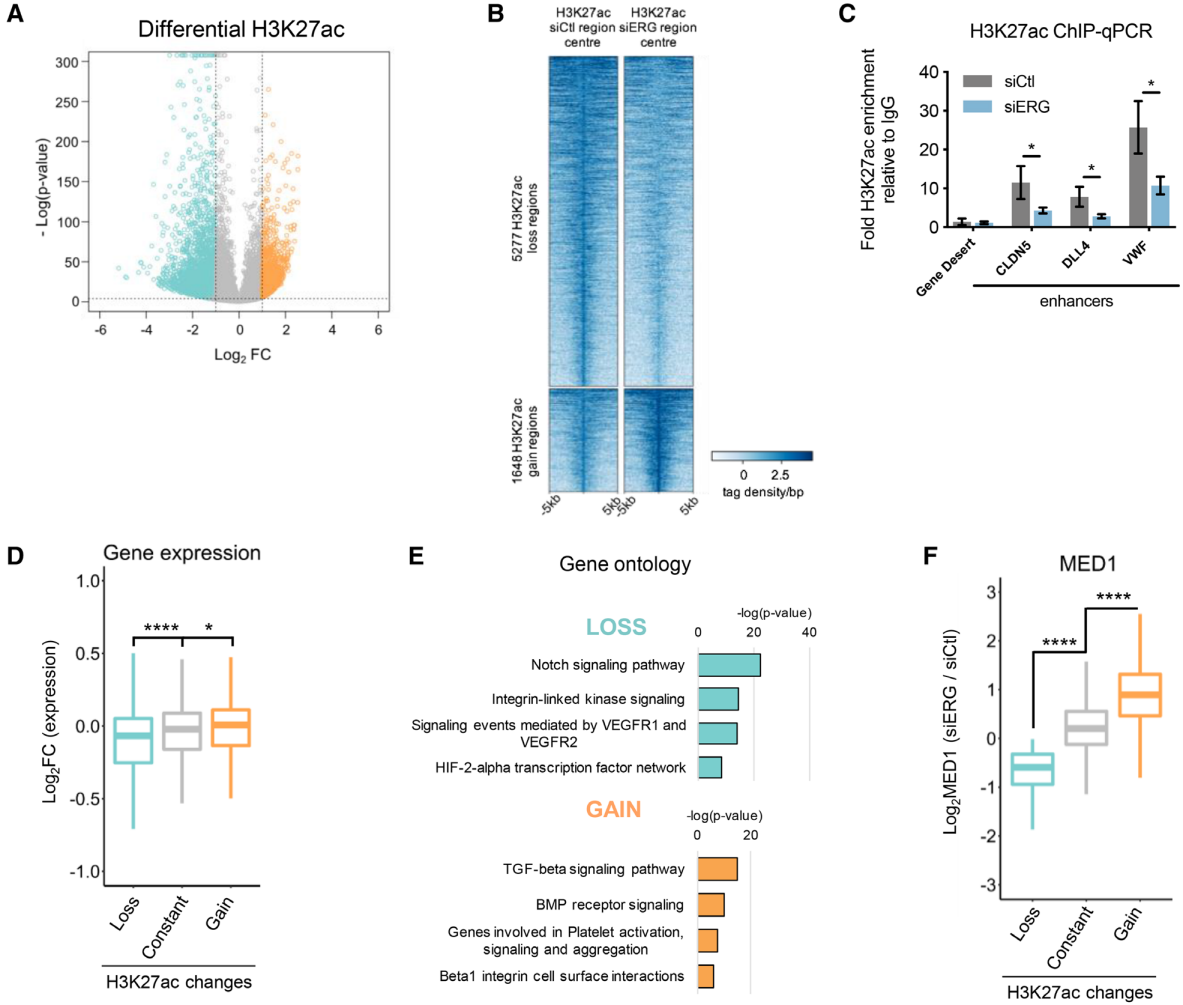
**ERG (ETS-related gene) contributes to enhancer activation in human umbilical vein endothelial cell (HUVEC). A**, Volcano plot showing log_2_ fold change (FC) vs –log (*P* value) of differential H3K27ac enrichment at siCtl H3K27ac regions in response to ERG knockdown. Loss and gain enhancer regions are selected by −1≥log_2_FC≥1; −log (*P* value)≥4. **B**, Heatmap of H3K27ac enrichment over input in all loss and gain regions. Signal ±5 kb from the center of H3K27ac siCtl or siERG regions as tag density/bp. **C**, Chromatin immunoprecipitation-quantitative polymerase chain reaction (ChIP-qPCR) of H3K27ac at enhancers of *CLDN5* (claudin-5), *DLL4* (Delta-like protein 4), and *VWF* (von Willebrand factor) with a negative control gene desert, in siCtl vs siERG-treated HUVEC. Graph represents fold change over IgG, n=3. *P<0.05, paired 2-tailed *t* test. **D**, Boxplot representing log_2_FC from transcriptome profiling data following ERG knockdown in H3K27ac regions changed in response to siERG (as defined in **A**). *P*<0.0001, Kruskal-Wallis test and post hoc test using Wilcoxon rank-sum test, ****P<0.0001; *P<0.05. **E**, Pathway analysis of genes associated with loss and gain of H3K27ac. **F**, Boxplot showing the log_2_FC of MED (Mediator complex subunit)-1 occupancy in siERG-treated HUVEC in H3K27ac regions changed in response to siERG. *P*<0.0001, Kruskal-Wallis test and post hoc test using Wilcoxon rank-sum test, ****P<0.0001. TGF indicates transforming growth factor. BMP indicates bone morphogenetic protein; HIF, hypoxia inducible factor; TGF, transforming growth factor; and VEGFR, vascular endothelial growth factor receptor.

We investigated whether ERG is required for H3K27 acetylation at endothelial enhancers by performing H3K27ac ChIP-seq analysis in HUVEC treated with control or ERG-siRNA (Online Figure VIA and VIB). In control HUVEC, 56 347 H3K27ac-bound regions were identified, which significantly correlated with those reported in the Encyclopedia of DNA Elements data^25^ (Online Figure VIC). In ERG-deficient cells, H3K27ac was modulated globally, with a decrease in H3K27ac at 5277 regions (loss) and an increase in 1648 regions (gain; Figure 6A and 6B). Changes in H3K27ac enrichment were validated by ChIP-qPCR across regions associated with selected endothelial ERG target genes (Figure 6C). Globally, in ERG-deficient cells changes in H3K27ac also correlated with the expression profile: expression of genes associated with loss of H3K27ac was significantly downregulated while expression of genes associated with gain of H3K27ac was significantly upregulated (Figure 6D; Online Figure VID). Genomic Regions Enrichment of Annotations Tool analysis of ERG-depleted HUVEC showed that loss of H3K27ac was associated with enrichment of Notch and VEGF (vascular endothelial growth factor) receptor signaling, pathways positively controlled by ERG^8,35^ (Figure 6E). In contrast, gain of H3K27ac was associated with TGF-β-SMAD signaling, a pathway repressed by ERG^11^ (Figure 6E).

**Figure 7.**
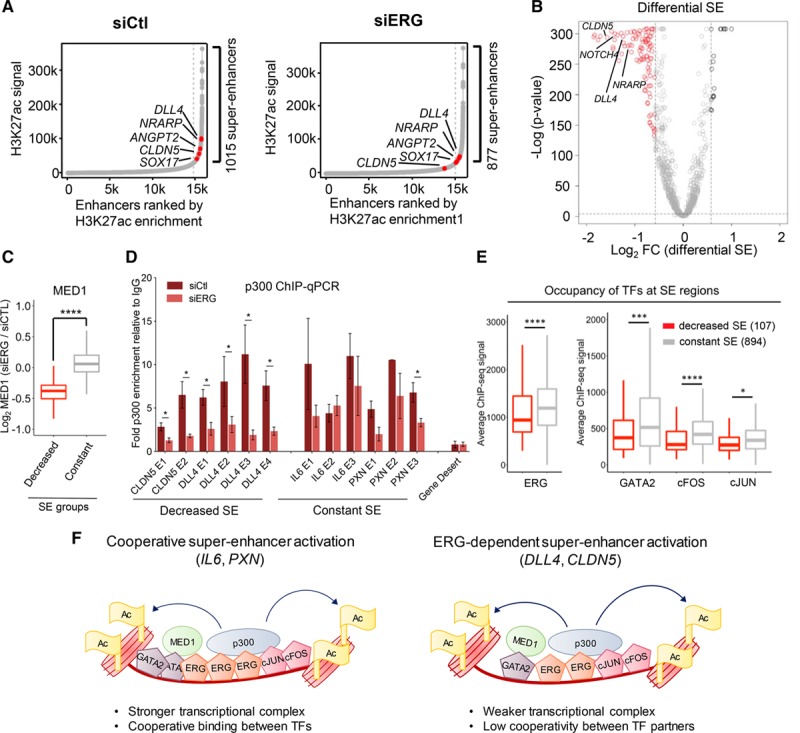
**ERG (ETS-related gene) regulates distinct endothelial super-enhancers (SEs). A**, SE identification on siCtl H3K27ac using H3K27ac enrichment from siCtl and siERG-treated human umbilical vein endothelial cell (HUVEC). Selected genes relevant to endothelial function are indicated in red (see also Online Table VIII). **B**, Volcano plot depicting the log_2_FC vs –log (*P* value) of differential SEs at 1015 H3K27ac SE identified in siCtl-treated cells. Significantly up or downregulated SEs are selected according to −0.58≥log_2_FC≥0.58 (−1.5≥FC≥1.5); −log (*P* value)≥4. **C**, Boxplot representing the log_2_FC of MED (Mediator complex subunit)-1 occupancy in siERG-treated HUVEC in decreased SE and constant SE. ****P<0.0001, Wilcoxon rank-sum test. **D**, Chromatin immunoprecipitation-quantitative polymerase chain reaction (ChIP-qPCR) of p300 enrichment in siCtl or siERG-treated HUVEC at selected SE constituent enhancers (E) associated with decreased SE (*CLDN5* [claudin-5], *DLL4* [Delta-like protein 4] or constant SE (*IL6*, *PXN*). Data are represented as fold change over IgG, n=4. *P<0.05, paired 2-tailed *t* test. **E**, Genomic occupancy of ERG and collaborative TFs: GATA-2 (GATA-binding protein 2), cFOS, and cJUN at decreased SE compared with constant SE, measured by the chromatin immunoprecipitation with deep sequencing signal in HUVEC. ****P<0.0001; ***P<0.001; *P<0.05, Wilcoxon rank-sum test. **F**, Model of ERG-dependent SE assembly in EC. Cooperative SE activation is associated with a strong transcriptional complex (ERG, AP-1 [activator protein 1], GATA-2) at constant SE (**left**). In a subset of SE, activation of SE is strongly dependent on ERG due to less abundance of transcription factor network partners with reduced cooperativity (**right**).

To investigate the effect of ERG depletion on the recruitment of basal transcriptional machinery to enhancers, we performed ChIP-seq for MED1 in control and ERG-deficient HUVEC. Loss or gain of MED1 occupancy in ERG-deficient cells coincided with a decrease or increase in H3K27ac, respectively (Figure 6F). These data indicate that ERG plays a role in modulating endothelial enhancers. MED1 is part of a large complex (Mediator) which interacts with super-enhancers.^19^ We therefore investigated the role of ERG in the organization of endothelial super-enhancers. H3K27ac ChIP-seq analysis in control HUVEC identified 1015 super-enhancer clusters (Figure 7A; siCtl), in line with the HUVEC super-enhancers profile identified from the Encyclopedia of DNA Elements data (see Figure 2A). ERG depletion by siRNA caused changes in H3K27ac levels leading to a redistribution of endothelial super-enhancers (Figure 7A; siERG). Comparison of H3K27ac super-enhancers in control versus ERG-depleted HUVEC identified a subset of 107 super-enhancers with decreased H3K27ac levels following loss of ERG. Among the ERG-regulated super-enhancers were those associated with key endothelial genes including *DLL4*, *NRARP*, and *CLDN5* (Figure 7B). The majority of super-enhancers showed no significant changes following ERG-siRNA, and only a few super-enhancers showed increased H3K27ac. MED-1 occupancy was also reduced in the subset of ERG-regulated super-enhancers (decreased super-enhancer) compared to those unchanged (constant super-enhancer; Figure 7C). Importantly, the ERG-dependent decrease in H3K27ac levels correlates with reduced expression of ERG target genes (Online Figure VIIA). Thus, ERG is functionally required to dynamically modulate H3K27ac levels in ECs leading to redistribution of a subset of super-enhancers.

### Cooperative TF Binding and p300 Recruitment in the Regulation of HUVEC Super-Enhancers

ERG has been shown to bind to p300^35^; thus, we hypothesized that ERG-dependent changes in H3K27ac at super-enhancers might be linked to the recruitment of p300 by ERG. This was tested by ChIP-qPCR for p300 enrichment on selected loci associated with validated ERG targets (*CLDN5*and *DLL4*), where H3K27ac was decreased on loss of ERG (decreased super-enhancer). These showed a significant decrease in p300 occupancy following ERG inhibition, suggesting that ERG is required to recruit p300 at these sites (Figure 7D). However, ERG inhibition did not consistently affect p300 recruitment at constant super-enhancers typified by *IL6* and *PXN* (Figure 7D). Online Figure VIIB illustrates the ERG-dependent decrease in H3K27ac and MED1 occupancy observed at the *CLDN5* and *DLL4* gene loci, compared to loci associated with constant super-enhancers, *IL6* and *PXN*. These findings suggest that ERG regulates a subset of super-enhancers partly through recruitment of the histone acetyltransferase p300.

We speculated that in the super-enhancers that remain constant following loss of ERG, other TFs might compensate for its absence. Previous studies have identified GATA and AP-1 (FOS/JUN) TF families as cooperating with ETS factors in regulating endothelial gene expression^16,36^; moreover, cJUN has been shown to bind p300.^37^ Analysis of ChIP-seq data from the Encyclopedia of DNA Elements for GATA-2, cFOS, and cJUN in HUVEC^25^ showed significant global overlap with ERG-bound sites (Online Figure VIIC). Higher occupancy of GATA-2, cFOS, and cJUN was present at constant super-enhancers compared with decreased super-enhancers (Figure 7E). This global distribution is reflected at representative loci for the 2 groups; *CLDN5, DLL4* (decreased super-enhancer) and *IL6*, *PXN* (constant super-enhancer; Online Figure VIIB).

These data suggest a model (Figure 7F) in which the majority of super-enhancers are regulated by a cooperative TF network involving ERG, AP-1, and GATA-2 that provide a strong transcriptional complex; thus loss of ERG can be compensated. However, in a subset of super-enhancer–associated lineage genes including *CLDN5* and *DLL4*, AP-1 and GATA-2 are less abundant, and therefore there is low cooperativity and super-enhancer assembly and gene expression are strongly dependent on ERG.

### Risk Variants for Cardiovascular and Other Diseases Are Enriched at ERG Super-Enhancers

**Figure 8.**
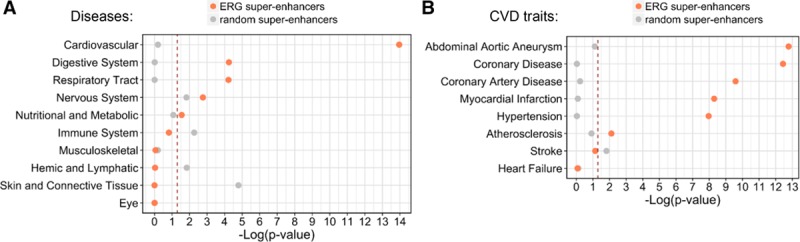
**ERG (ETS-related gene) super-enhancers (SEs) prioritize cardiovascular disease (CVD) variants. A**, Overlap of genome-wide association studies single nucleotide polymorphisms (SNPs) associated with disease trait classes within ERG-defined SE (orange) with chromosome and size-matched random SE (gray). Significance of enrichment was calculated by binomial distribution test with red dashed line indicating *P*=0.05. Some points overlay. **B**, Test for enrichment of selected CVD trait-associated SNPs within ERG-defined SE (orange) with chromosome and size-matched random SE (gray), as performed in **A**. Random region were restricted to open chromatin by excluding placement in repressed chromatin states in human umbilical vein endothelial cell.

Several studies have recently shown that disease-associated SNPs identified through genome-wide association studies are preferentially enriched in the super-enhancer regions of disease-relevant cells and can map to TF-binding sites.^18,38^ Endothelial dysfunction is implicated in many diseases. We examined the enrichment of disease-associated variants at ERG-binding loci, ERG-bound enhancers and ERG super-enhancers, using SNPs reported in the NCBI dbGaP^39^ and NHGRI-GWAS catalogs.^40^ We determined enrichment by using a null distribution of background population variants. Analysis at ERG-binding loci identified association with SNPs for immune diseases and CVD (Online Figure VIIIA). At ERG-bound enhancers, the significance of enrichment is greatly amplified, with a similar repertoire of disease traits (Online Figure VIIIB). Interestingly, analysis at ERG super-enhancers identified SNPs for CVD as the most highly associated disease trait (*P*=1.1×10^−^^14^; Figure 8A). ERG super-enhancers were also enriched in diseases for digestive system (*P*=5.7×10^−^^5^) and respiratory tract (*P*=6.1×10^−^^5^; Figure 8A). This enrichment was not identified at size- and chromosome-matched regions randomly shuffled (permuted) across permissive chromatin. Further interrogation of the CVD-associated SNPs revealed strong significant enrichment for traits such as abdominal aortic aneurysm (*P*=1.7×10^−^^13^), coronary artery disease (*P*=2.6×10^−^^10^), myocardial infarction (*P*=5.1×10^−^^9^), and hypertension (*P*=1.1×10^−^^8^), but not heart failure (Figure 8B), suggesting a closer association with diseases where EC contribute most to the pathogenesis.

These results identify a novel mechanism through which disease-associated noncoding SNPs may cause vascular dysfunction and increased disease risk, and suggest a possible functional link between ERG-dependent transcriptional regulation of endothelial gene expression and the predisposition to CVD.

## Discussion

In this study, we characterize the TF ERG as a crucial regulator of enhancers and super-enhancers in HUVEC. Multiple studies have shown that ERG is essential to maintain endothelial homeostasis (reviewed in Shah et al^2^). Here, we define the ERG-dependent endothelial epigenome and associate this with genetic variants linked to CVD and other diseases, suggesting novel potential strategies for biomarkers and target identification.

Our study identifies ERG as a positive regulator of a core set of putative endothelial super-enhancers in HUVEC. We describe an ERG-dependent subset of super-enhancers, associated with essential endothelial genes such as *DLL4*, *CLDN5*, and *NRARP*. Crucially, ERG-dependent super-enhancers are significantly associated with ERG-activated genes. Analysis of *DLL4* and *CLDN5* loci shows that recruitment of p300 at these super-enhancers is controlled by ERG; direct interaction between ERG and p300^35^ suggests a mechanism by which ERG recruits p300 to genomic loci for H3K27 acetylation. However, inhibition of ERG expression in HUVEC did not perturb activity in most super-enhancer regions. This is not surprising because super-enhancers are characterized by the presence of multiple TF-binding sites and a high degree of enrichment of transcriptional coactivators, providing opportunities for cooperative binding and synergistic gene activation.^18,19^ Transcriptional networks consisting of members of the ETS (including ERG’s closest homolog FLI-1 [friend leukemia integration 1 transcription factor]), AP-1 and GATA families have been shown to bind endothelial enhancers.^16,41^ Furthermore, ERG and AP-1 have been shown to functionally interact at composite DNA-binding sites in non-EC.^42^ As suggested from studies on AP-1,^43^ endothelial enhancer selection may be facilitated by cooperative ERG-AP-1 binding, where ERG is acting as the endothelial-specific TF. A recent study investigated the effect of combined knockdown of ERG and its closest homolog FLI-1 on global H3K27ac levels in HUVEC and found a significant loss of H3K27ac on key endothelial genes,^41^ supporting the notion that multiple TFs are directing cooperative transcriptional regulation. In our study, we found that the majority of endothelial super-enhancers are co-occupied by ERG, AP-1 members cFOS/cJUN, and GATA-2. Interestingly, we found that the subset of super-enhancers not affected by ERG depletion is highly co-occupied by ERG, AP-1, and GATA-2, while lower levels of all TFs are present at the subset sensitive to ERG depletion. We propose that a cooperative TF network is able to compensate for ERG depletion at most super-enhancers; however, a specific subset of core super-enhancers is strictly dependent on ERG function, highlighting its key role in regulating endothelial gene expression.

ERG may modulate super-enhancer activity through mechanisms other than p300 recruitment. A potential mechanism may be through targeting the activity of the BAF (BRG-1–associated factors) chromatin remodeling complex which disrupts histone-DNA interactions to control access to DNA.^44^ Recent studies have indicated a role for both AP-1 and ERG in binding to BAF subunits to establish accessible chromatin for enhancer selection and target gene regulation.^43,45^ The mechanism through which ERG modulates MED-1 recruitment remains unclear. Mediator complex has been implicated in long-range chromatin interactions functionally combining enhancers from many kilobases away. In fact, super-enhancers have been shown to form higher-order 3-dimensional (3D) chromatin structures which are likely to coordinate their activity in an orchestrated manner.^38,46^ In this study, we followed the current standard convention when annotating super-enhancer–associated regions with ERG-bound loci, namely by their linear distance along the epigenome.^28^ This methodology does not take into account the complex 3D chromatin structure. Further studies will be required to map super-enhancers using long-range chromatin interactions in the HUVEC genome.

Aberrant expression of lineage-specific TF in other tissues cause deregulated activation of a transcriptome profile detrimental to the cell^15^; this is indeed the case with ERG, which acts as an oncogene when overexpressed in cells such as the prostate epithelium.^13^ We show that ERG-associated super-enhancer profiles are markedly different in HUVEC compared with VCaP cells. Thus, ERG does not co-opt an endothelial genomic profile in VCaP cells but controls fundamentally different pathways in these 2 cell types, partly through selective super-enhancer binding. These findings provide some insight into the molecular basis for ERG’s homeostatic versus oncogenic functions. We postulate that different ERG-dependent gene expression between HUVEC and VCaP cells may be regulated in part by the activity of cell-specific pioneer factors which act to prime chromatin for accessibility at lineage-specific sites.^47^

Super-enhancer regions are commonly enriched in cell type-specific disease-related SNPs.^18,38^ Hogan et al^16^ identified disease trait-associated SNPs for coronary artery disease and hypertension within aortic endothelial enhancers. Our analysis of ERG-bound super-enhancers revealed enrichment for SNPs associated with diseases that have a vascular component, including predisposition to CVDs, such as atherosclerosis and coronary artery disease. These data support the notion that active maintenance of endothelial homeostasis through transcriptional programs is essential protection against a number of diseases, most of all CVD. Interestingly, recent genome-wide association studies meta-analysis revealed a novel risk locus for abdominal aortic aneurysm within the ERG gene itself.^48^ Further studies will determine the functional role of noncoding variants associated with ERG enhancers, and will provide crucial insight into the contribution of ERG, cooperative TF and cofactor binding in complex disease susceptibility.

In conclusion, this study provides novel evidence on the transcriptional and epigenetic mechanisms which controls lineage-specific gene expression in EC and identifies a possible functional link between regulation of ERG activity and human disease. These associations will provide valuable insights for investigating the role of ERG-dependent regulatory programs in maintaining endothelial homeostasis and protecting against vascular diseases.

## Acknowledgments

We thank Dr Joan Ponsà (Imperial College London, United Kingdom) for helpful discussions.

## Sources of Funding

This study was funded by grants from the British Heart Foundation (RG/11/17/29256; RG/17/4/32662; FS/15/65/32036; and PG/17/33/32990) and Cancer Research UK.

## Disclosures

None.

## Supplementary Material

## Novelty and Significance

### What Is Known?

- The ETS (E-26 transformation-specific) transcription factor (TF) ERG (ETS-related gene) is essential for endothelial homeostasis, whereas its aberrant overexpression in cancer is oncogenic.
- Lineage-specific TFs bind to chromatin regulatory elements, called super-enhancers, which are enriched in single nucleotide polymorphisms (SNPs) associated with specific diseases, including cardiovascular disease (CVD).
- Epigenomic mechanisms are emerging as key players in CVD; little is known about the epigenetic regulation of endothelial function and its links to CVD.
