# Supplementary material for: The Transcription Factor ERG Regulates Super-Enhancers Associated With an Endothelial-Specific Gene Expression Program
Source: Circ Res. 2019 Mar 20;124(9):1337–49. doi: 10.1161/CIRCRESAHA.118.313788 (PMC6493686; doi:10.1161/CIRCRESAHA.118.313788)
Supplement: Supplementary file 1 [file res-124-1337-s001.pdf]

SUPPLEMENTAL MATERIAL

Expanded Methods Section

Primary endothelial cells and VCaP cell lines

Pooled human umbilical vein endothelial cells (HUVEC) were purchased from Lonza (Wokingham, United Kingdom) and cultured in EGM-2 media (Lonza) or isolated from umbilical cords, as previously described[1] and cultured in M199 media. Cells were used up to passage 4. The prostate cancer cell line VCaP were obtained from American Type Culture Collection (ATCC) and cultured in DMEM supplemented with 10 % fetal bovine serum (Biosera, Labtech).

Gene silencing and validation by RT-qPCR and immunoblotting

HUVEC were cultured on 1% gelatin in EGM2 and transfected with 20 nM siRNA against ERG exon 6 (Qiagen; 5′-CAGATCCTACGCTATGGAGTA-3′) or 20 nM AllStars Negative Control siRNA (Qiagen) using AtuFECT01 lipid (1 µg/ml, Silence Therapeutics, Berlin, Germany). VCaP cells were transfected using 100 nM siRNA targeting exon 7 of ERG (Invitrogen; 5′-ACTCTCCACGGTTAATGCATGCTAG-3′) or Stealth negative control siRNA (Invitrogen) using Lipofectamine RNAiMAX (Invitrogen). SiRNA treatments were carried out for 48 hrs. RNA was extracted from siRNA-treated HUVEC using the RNeasy kit (Qiagen). Synthesis of cDNA was carried out using Superscript III Reverse Transcriptase (Invitrogen). Quantitative real-time PCR was performed on a Bio-Rad CFX96 system using PerfeCTa SYBR Green Fastmix (Quanta Biosciences). Sequences of the oligonucleotides used for qPCR are listed in Online Table IV.

In some experiments, HUVEC were transfected with siRNA to ERG or Ctrl, as above for 48 hr. Cells were fixed in 4% formaldehyde and cell pellets collected for chromatin immunoprecipitation (ChIP) sequencing for H3K27ac and MED1.

Whole cell protein lysates were prepared from HUVEC using CelLytic reagent (Sigma). Immunoblotting of cell lysates was performed according to standard conditions. Immunoblots were labelled with the following primary antibodies: anti-ERG (ab133264, 1:1000, Abcam) and anti-GAPDH (MAB374, 1:10000, Millipore). Primary antibodies were detected using fluorescently labelled secondary antibodies: goat anti-rabbit IgG DyLight 680 and goat anti-mouse IgG Dylight 800 (Thermo Scientific). Detection and quantification of fluorescence intensity were performed using an Odyssey® CLx imaging system (LI-COR Biosciences, Lincoln) and Odyssey® Image Studio v4.0 software.

Plasmid Transfections and Reporter Assays

A 1.2 kb DNA fragment containing the *CDH5* promoter sequence was cloned into pGL4.10 luciferase reporter vector (Promega), as previously described[1]. Three putative *CDH5* enhancers (E1, E2 and E4) were amplified by PCR from human genomic DNA and cloned into *Sal*I and *Bam*HI sites of pGL4.10 located approximately 2 kb upstream of the *CDH5* promoter insert. Mutation of the putative ERG DNA binding

sites in the *CDH5* E4 enhancer region was carried out using QuikChange Lightning Multi Site-Directed Mutagenesis Kit (Agilent, Berkshire, UK), according to the manufacturer's instructions. In total, nine AGGAA motifs were changed to ACCAA to eliminate the ERG DNA binding motifs present within the E4 region. Sequences of the mutagenic oligonucleotides are listed in Online Table IV.

HUVEC were plated into a 24-well plate 24 h before transfection in EGM2 media. Cells were transiently transfected with plasmid DNA using GeneJuice transfection reagent (Merck Chemicals, Nottingham, UK), according to the manufacturer's instructions. For transactivation assays, the *CDH5* promoter reporter constructs were co-transfected into cells along with *Renilla* luciferase and either an ERG cDNA expression plasmid (pcDNA-ERG) or an empty plasmid (pcDNA3.1). Luciferase reporter activity was normalized to the internal *Renilla* luciferase control and is expressed relative to pcDNA3.1 empty vector control using the Dual-Luciferase Reporter Assay System (Promega) and a Synergy HT microplate reader.

Chromatin immunoprecipitation (ChIP)-qPCR

ChIP experiments were performed using the ChIP-IT express kit (Active Motif). HUVEC transfected with control or ERG siRNA were cross-linked with 1 % formaldehyde. Chromatin was sheared for four cycles of 30s on and 30s off using a Bioruptor ICD-200 ultrasound sonicator (Diagenode). Generated fragments of 200-1,000 bp were chromatin immunoprecipitated using 2 μg anti-H3K27ac (cat no. 39133, Active Motif) and 3 μg anti-p300 (ab14984, Abcam) antibodies. The respective negative controls were rabbit IgG (PP64, Chemicon, Millipore) and mouse IgG (12-371, Millipore). QPCR for selected genomic regions were performed on immunoprecipitated DNA using primers listed in Online Table III. Statistical significance was determined using a two-tailed paired sample t-test in R with a statistical significance cut-off p-value < 0.05. Data were plotted using Prism 8.0 (Graph Pad).

ChIP sequencing

ChIP assays using chromatin from pooled HUVEC (10^7 cells) cultured in EGM2 media were performed using 2 µg of rabbit polyclonal antibody against ERG (sc-354X, Santa Cruz Biotechnology), as described[2], with minor modifications by introducing an additional washing step with high salt buffer (0.1 % SDS, 1 % Triton X-100, 2 mM EDTA, 20 mM Tris-HCl, 500 mM NaCl). Immunoprecipitated chromatin was validated by quantitative real-time polymerase chain reaction (qPCR) with primers to previously characterized ERG-bound loci (*CDH5, ICAM1*). QPCR was performed using PerfeCTa SYBR Green Fastmix (Quanta Biosciences) on a Bio-Rad CFX96 system. See Online Table III for list of oligonucleotides used for ChIP experiments in this study. Library generation was performed using TruSeq ChIP Sample Prep Kit (Illumina). In brief, 10 ng DNA ends were polished, 5′-phosphorylated, and 3′-dATP added before genomic adapters were ligated onto the samples, following the manufacturer's instructions (Illumina). A library was also generated for an input sample which was an aliquot of sonicated cell lysate with no subsequent immunoprecipitation step. Agarose gel electrophoresis showed that the size fractionated ligated DNA migrated at 250–300 bp. The DNA libraries were amplified by PCR and sequenced using the Illumina HiSeq 2000.

H3K27ac and MED1 ChIP-seq in HUVEC were performed using a custom protocol (Active Motif Inc). Immunoprecipitation was carried out with 4 µg anti-H3K27ac (cat no. 39133, Active Motif) and 5 µg anti-MED1 (A300-793A, Bethyl Laboratories) antibodies. H3K27ac and MED1 ChIP and input libraries were prepared for sequencing on an Illumina NextSeq 500 platform.

ChIP-seq dataset analyses

Fastq files generated from sequencing were mapped to the human reference genome (build hg19) using Bowtie 2 with default parameters, to produce SAM files[3]. SAM files were converted to BAM files and subsequently sorted and indexed using SAMTools[4]. Peak calling on aligned sequencing data was performed using Model-based Analysis of ChIP-seq (MACS version 2.1.2)[5] with input datasets as the control. To identify transcription factor and co-factor binding sites (e.g. ERG, MED1) a cutoff detection p-value of 1e-5 was used, whereas for histone modifications a cutoff detection p-value of 1e-9 was used. Called peaks overlapping ENCODE blacklisted genomic regions[6] were excluded. BAM files were also converted to Bedgraph and then to BigWig using genomeCoverageBed and wigToBigWig from BedTools, respectively[7]. Tracks were visualized using the UCSC Genome Browser database (https://genome.ucsc.edu). ChIP-seq dataset annotation was performed using the Bioconductor package ChIPseeker[8] in R. Peak-to-gene annotation is based on the peak genomic region and distance to the nearest peak.

Sequencing data generated in this study (ChIP-Seq datasets for ERG, H3K27ac and MED1 in HUVEC) can be downloaded from the NCBI Gene Expression Omnibus (GEO) portal, accession number GSE124893.

Publicly available data were downloaded as fastq files from the European Nucleotide Archive (ENA) and processed as described above. All datasets accessed or generated in this study are listed in Online Table IX. Genome-wide ChIP-seq data for histone modifications and DNase I hypersensitivity in HUVEC were obtained from the ENCODE/Broad Institute under GEO accession number GSE29611. ChIP-seq data for other transcription factors: GATA2, cFOS and cJUN were analyzed (GSE31477). ChIP-seq datasets for VCaP cells were obtained from published sources: ERG from *Chng et al.* (GSE28951), ERG from *Yu et al.* (GSE14092), H3K27ac (GSE55064), H3K4me1 and H3K27me3 (GSE14092).

Gene expression profiling

Human transcriptome profiling datasets following knockdown of ERG were obtained from Birdsey *et al.*[9] for HUVEC (GSE32984) and Wang *et al.*[10] for ERG:TMPRSS2 fusion-positive VCaP prostate cancer cells (GSE53994). Differential expression analysis of the Affymetrix and Illumina BeadChip microarray datasets were performed in R version 3.2.4 using the Limma package[11]. The analysis considered significant differentially expressed genes with a Benjamini and Hochberg adjusted p-value < 0.10 which allowed a representational proportion of potentially meaningful gene expression changes to be validated. Heatmaps to visualise gene expression changes were plotted in R.

Defining super-enhancers

Enhancer regions were identified by profiling H3K27ac and H3K4me1 ChIP-Seq enrichment in HUVEC using the method by Loven *et al.*[12]. We used the ROSE algorithm to define super-enhancers[12, 13]. Briefly, active enhancers spaced by < 12.5kb and located further than 2 kb from annotated promoters were concatenated. The ChIP-seq occupancy of H3K27ac in reads per million per bp (rpm/bp) was used to separate super-enhancers from typical enhancers by ranking stitched enhancers based on an increasing H3K27ac ChIP-seq signal. The inflection point provided a cut-off to define super-enhancers. An analogous procedure was used to define super-enhancer regions by enrichment of ERG at enhancers. Super-enhancer associated genes were assigned as described earlier using ChIPseeker.

Analysis of genomic regions

ChIP-seq data for correlation, heatmap amd aggregate plots were normalized to input data using the bamCompare tool in deepTools[14] and reported as log_2 of the ratio. Resulting bigWig files were used to obtain a multiBigwigSummary in deepTools from which correlations were plotted in R using the ggplot2 package. Scores in defined genomic regions were calculated from computeMatrix to generate the heatmaps in Figure 6B and Online Figure VIIC.

To generate randomly shuffled genomic intervals to serve as a background for null hypothesis testing, shuffleBED from BEDTools[7] was used. For Figure 1D size-matched regions (relative to the true genomic regions identified by histone modifications) were obtained across the hg19 reference genome. Overlap between genomic regions of different peak sets were compared using BEDTools intersect[7].

ChIP-seq peak annotations in Figure 4D were visualised using the ChIPseeker[10] function *plotDistToTSS*.

Hypergeometric distribution testing to compare the overlap of promoters and enhancers between HUVEC and VCaP cells in Figure 4E used genome-wide occurrences of promoter and enhancer regions as the expected (background) frequency. Promoter regions were those defined by RefSeq annotations from NCBI[15] and enhancers were from the FANTOM5 project using Cap Analysis of Gene Expression (CAGE)[16].

Functional enrichment and motif analysis

GSEA software (v2.2.0) from the Broad Institute at MIT[17] was used to identify how gene sets distribute in gene lists ranked by either microarray gene expression fold change values or by H3K27ac ChIP-seq enrichment on enhancers. The significance of the normalized enrichment score (NES) was based on a p-value adjusted for multiple comparisons and a false discovery rate (FDR).

To find the most enriched motifs in a given peak set the 'findMotifsGenome.pl' script from HOMER was performed[18]. For transcription factor binding sites the genomic interval ±200bp from the center of the binding site was used. For H3K27ac regions the exact region size was analyzed. The script adopts a set of sequences from the hg19 reference genome build as a background control that is matched in size and GC

content. The significance of enriched motifs reported is reflected in the number and size of genomic regions assessed.

To identify transcription factor motifs for ERG, GATA2, cFOS and cJUN across the human reference genome hg19, the scanMotifGenomeWide.pl script was applied from HOMER[18].

Differential binding analysis

Tag directories of ChIP-seq alignment files were generated using the makeTagDirectory command in HOMER. Called H3K27ac peaks assigned using MACS (cutoff detection p-value of 1e-9) in the control siRNA (siCtl) sample. These were assessed by getDifferentialPeaks to define loss and gain H3K27ac regions. The command was used to determine the peaks with more or less density tags in the siCtl dataset relative to the siERG dataset. Differential peaks were reported with log_2 fold changes and a cumulative Poisson p-value < 0.0001. Similarly, this command was used to find differential super-enhancers in the control siRNA super-enhancer set relative to siERG. Volcano plots were produced with log_2 fold changes and p-value cutoffs in R.

Functional annotation analyses

Biological functions were assigned to regulatory regions using Genomic Regions Enrichment of Annotations Tool (GREAT)[19] with enriched gene ontology (GO) Biological Processes or MSigDB Pathways reported at a significance cutoff FDR < 0.001. Biological network pathways for Online Figure IVD were derived from the Reactome database[20].

GWAS SNP enrichment analysis

To perform GWAS SNP enrichment analysis, the R package traseR was used[21]. SNP-trait associations were obtained by combining dbGaP[22] and NHGRI GWAS Catalog[23]. We also considered SNPs in LD from the 1000 Genomes Project ($r^2 > 0.8$, and located within 100kb of the lead SNP) giving a total of 78,247 unique trait-associated SNPs. Background SNPs obtained from Utah residents with ancestry from Northern and Western Europe (CEU) population, available from the 1000 Genomes Project[24], were used for hypothesis testing to assess the enrichment of trait-associated SNPs in genomic intervals of ERG super-enhancers, ERG binding loci and ERG-bound enhancer in HUVEC. The enrichment test follows the assumptions of the null hypothesis where the number of observed disease-associated SNPs out of background SNPs in ERG genomic intervals follows a binomial distribution with probability equal to the proportion of all genome-wide disease-associated SNPs out of background SNPs. Significance of enrichment was tested by binomial distribution. Analysis was performed for the enrichment of a select 10 trait class categories and all 573 available traits. A statistical p-value < 0.05 was used to as a threshold cutoff for significance. To provide a background to test the null hypothesis that random genomic intervals were significantly enriched for disease-associated SNPs we generated chromosome and size matched genomic regions (relative to ERG super-enhancers, ERG binding loci and ERG-bound enhancers) using shuffleBED from BEDTools[7]. These regions were also excluded from repressed (high H3K27me3) chromatin states in the HUVEC genome to provide a more rigorous test set.

Statistical Analyses

All statistical analyses were performed in R. Associated statistics for specific analyses are mentioned elsewhere in the Methods. Fisher's exact test is performed across contingency tables when sample sizes were small (less than ~1000) and a Chi-squared test for larger sample sizes (more than 1000). For data represented in a boxplot the sample distribution was tested for normality using a Shapiro-Wilk test. All data were found to be significantly not normally distributed and therefore a non-parametric test was used to test for significance between different groups. A Wilcoxon sum-ranks test was performed when comparing two groups. A Kruskal-Wallis test was used when comparing multiple groups (more than two) followed by a Wilcoxon sum-ranks test as the post-hoc test.

Reference List

1. Birdsey GM, Dryden NH, Amsellem V, Gebhardt F, Sahnan K, Haskard DO, Dejana E, Mason JC, Randi AM. Transcription factor erg regulates angiogenesis and endothelial apoptosis through ve-cadherin. *Blood*. 2008;111:3498-3506
2. Wilson NK, Foster SD, Wang X, Knezevic K, Schutte J, Kaimakis P, Chilarska PM, Kinston S, Ouwehand WH, Dzierzak E, Pimanda JE, de Bruijn MF, Gottgens B. Combinatorial transcriptional control in blood stem/progenitor cells: Genome-wide analysis of ten major transcriptional regulators. *Cell Stem Cell*. 2010;7:532-544
3. Langmead B, Salzberg SL. Fast gapped-read alignment with bowtie 2. *Nat Methods*. 2012;9:357-359
4. Li H, Handsaker B, Wysoker A, Fennell T, Ruan J, Homer N, Marth G, Abecasis G, Durbin R. The sequence alignment/map format and samtools. *Bioinformatics*. 2009;25:2078-2079
5. Zhang Y, Liu T, Meyer CA, Eeckhoute J, Johnson DS, Bernstein BE, Nusbaum C, Myers RM, Brown M, Li W, Liu XS. Model-based analysis of chip-seq (macs). *Genome Biol*. 2008;9:R137
6. Maher B. Encode: The human encyclopaedia. *Nature*. 2012;489:46-48
7. Quinlan AR, Hall IM. Bedtools: A flexible suite of utilities for comparing genomic features. *Bioinformatics*. 2010;26:841-842
8. Yu G, Wang LG, He QY. Chipseeker: An r/bioconductor package for chip peak annotation, comparison and visualization. *Bioinformatics*. 2015;31:2382-2383
9. Birdsey GM, Dryden NH, Shah AV, Hannah R, Hall MD, Haskard DO, Parsons M, Mason JC, Zvelebil M, Gottgens B, Ridley AJ, Randi AM. The transcription factor erg regulates expression of histone deacetylase 6 and multiple pathways involved in endothelial cell migration and angiogenesis. *Blood*. 2012;119:894-903
10. Wang S, Kollipara RK, Srivastava N, Li R, Ravindranathan P, Hernandez E, Freeman E, Humphries CG, Kapur P, Lotan Y, Fazli L, Gleave ME, Plymate SR, Raj GV, Hsieh JT, Kittler R. Ablation of the oncogenic transcription factor

erg by deubiquitinase inhibition in prostate cancer. *Proc Natl Acad Sci U S A*. 2014;111:4251-4256

11. Ritchie ME, Phipson B, Wu D, Hu Y, Law CW, Shi W, Smyth GK. Limma powers differential expression analyses for rna-sequencing and microarray studies. *Nucleic Acids Res*. 2015;43:e47
12. Loven J, Hoke HA, Lin CY, Lau A, Orlando DA, Vakoc CR, Bradner JE, Lee TI, Young RA. Selective inhibition of tumor oncogenes by disruption of super-enhancers. *Cell*. 2013;153:320-334
13. Whyte WA, Orlando DA, Hnisz D, Abraham BJ, Lin CY, Kagey MH, Rahl PB, Lee TI, Young RA. Master transcription factors and mediator establish super-enhancers at key cell identity genes. *Cell*. 2013;153:307-319
14. Ramirez F, Ryan DP, Gruning B, Bhardwaj V, Kilpert F, Richter AS, Heyne S, Dundar F, Manke T. Deeptools2: A next generation web server for deep-sequencing data analysis. *Nucleic Acids Res*. 2016;44:W160-165
15. Coordinators NR. Database resources of the national center for biotechnology information. *Nucleic Acids Res*. 2013;41:D8-D20
16. FANTOM Consortium. Forrest AR, Kawaji H, Rehli M, *et al.* A promoter-level mammalian expression atlas. *Nature*. 2014;507:462-470
17. Subramanian A, Tamayo P, Mootha VK, Mukherjee S, Ebert BL, Gillette MA, Paulovich A, Pomeroy SL, Golub TR, Lander ES, Mesirov JP. Gene set enrichment analysis: A knowledge-based approach for interpreting genome-wide expression profiles. *Proc. Natl. Acad. Sci. U. S. A*. 2005;102:15545-15550
18. Heinz S, Benner C, Spann N, Bertolino E, Lin YC, Laslo P, Cheng JX, Murre C, Singh H, Glass CK. Simple combinations of lineage-determining transcription factors prime cis-regulatory elements required for macrophage and b cell identities. *Mol. Cell*. 2010;38:576-589
19. McLean CY, Bristor D, Hiller M, Clarke SL, Schaar BT, Lowe CB, Wenger AM, Bejerano G. Great improves functional interpretation of cis-regulatory regions. *Nat. Biotechnol*. 2010;28:495-501
20. Fabregat A, Sidiropoulos K, Garapati P, Gillespie M, Hausmann K, Haw R, Jassal B, Jupe S, Korninger F, McKay S, Matthews L, May B, Milacic M, Rothfels K, Shamovsky V, Webber M, Weiser J, Williams M, Wu G, Stein L, Hermjakob H, D'Eustachio P. The reactome pathway knowledgebase. *Nucleic Acids Res*. 2016;44:D481-487
21. Chen L, Qin ZS. Traser: An r package for performing trait-associated snp enrichment analysis in genomic intervals. *Bioinformatics*. 2016;32:1214-1216
22. Mailman MD, Feolo M, Jin Y, Kimura M, Tryka K, Bagoutdinov R, Hao L, Kiang A, Paschall J, Phan L, Popova N, Pretel S, Ziyabari L, Lee M, Shao Y, Wang ZY, Sirotkin K, Ward M, Kholodov M, Zbicz K, Beck J, Kimelman M, Shevelev S, Preuss D, Yaschenko E, Graeff A, Ostell J, Sherry ST. The ncbi dbgap database of genotypes and phenotypes. *Nat Genet*. 2007;39:1181-1186
23. MacArthur J, Bowler E, Cerezo M, Gil L, Hall P, Hastings E, Junkins H, McMahon A, Milano A, Morales J, Pendlington ZM, Welter D, Burdett T, Hindorff L, Flicek P, Cunningham F, Parkinson H. The new nhgri-ebi catalog of published genome-wide association studies (gwas catalog). *Nucleic Acids Res*. 2017;45:D896-D901
24. Genomes Project Consortium, Auton A, Brooks LD, Durbin RM, Garrison EP, Kang HM, Korbel JO, Marchini JL, McCarthy S, McVean GA, Abecasis GR. A global reference for human genetic variation. *Nature*. 2015;526:68-74.

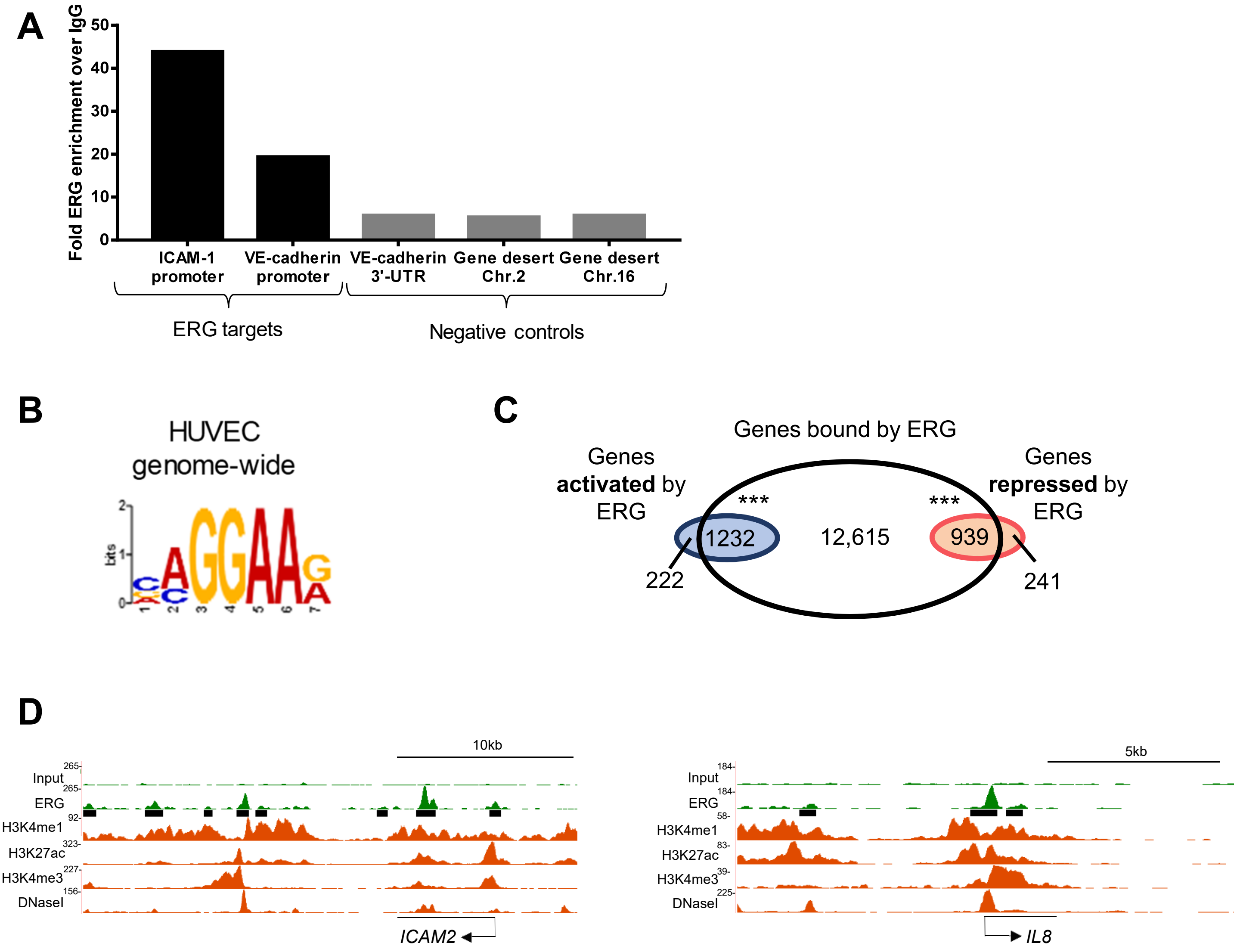

Online Figure I. Regulation of endothelial gene expression by integrated ERG DNA binding and transcriptome profiling. (**A**) ChIP-qPCR validation of ERG binding to the promoter regions of *CDH5* (VE-cadherin) and *ICAM1* (ERG targets) shows ERG enrichment to these sites compared to regions identified in the ChIP-seq which displayed no ERG binding (negative controls). (**B**) *De novo* motif discovery algorithms define the ERG DNA binding motif in HUVEC. (**C**) Comparison of global transcriptome profiling of ERG-deficient HUVEC with ERG ChIP-Seq data defines bound ERG transcriptional target genes. ERG activated and repressed genes are significantly more bound than expected by chance, ***P< 0.0001, Fisher's exact test. (**D**) ChIP-seq binding profiles for ERG, H3K4me1, H3K4me3, H3K27ac and DNase I hypersensitivity in HUVEC. ERG binding sites are shown as black bars.

A

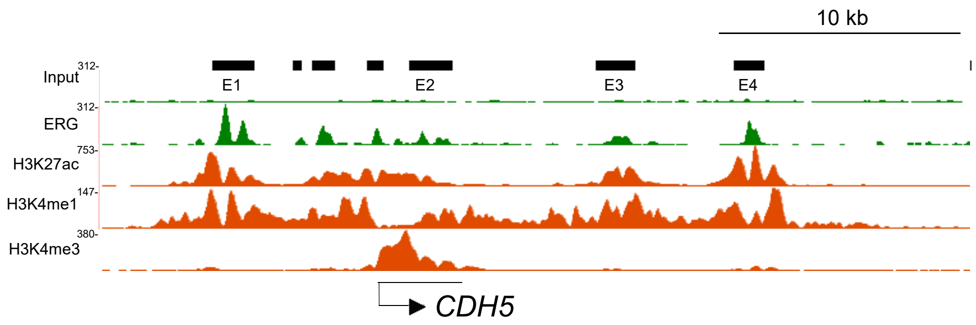

B

Enhancer Region E 4

```
TAGGAAAACCAGCATTCCTGACTCTCAACACAAATGCGAGCTCTGTGTCATACAGGAAAATGTCGTCAATC
CACCTGCTTCCTTCCCGGTGGGATAATTGTGCCTTGAATCAGCCTCTAAAAAGAATGTCTGACCTCAAGGA
AAATCTATGGAGTGAGTCAAGGAAAGTCCGTAGAAAGTGGGGGCTACCTTTCAAGGGCTCGCCCAAGGGTT
TCTAGCAGCAGGAAGAACGCCAGGCCTGCTGTCATTTGCCAACAGCATTCACTTGGCCCCTGGCATGGGGC
TGGGTCTCCGAGGAACCACAAGGGGTCGTCACGCTGGGAAGAAACAAGGGTGCCTGGAAAAGACAAGGACA
GGGCTCCAAGGATCACCTCCGGCTCCTGAGGATGTAATCTGAGGGAAGGAA
```

C

Mutagenesis of putative ERG binding motifs in region E4

```
Wt     AGGAA..TTCCT….AGGAA……TTCCT…AGGAA….AGGAA…..AGGAA…..AGGAA.…AGGAA
Mut    AccAA….TTggT….AccAA….TTggT…AccAA….AccAA…..AccAA…..AccAA….AccAA
```

Online Figure II. ERG binds distal enhancers along the *CDH5* gene loci. (**A**) ERG binding and histone modification enrichment along the promoter and distal regions of the proximal VE-cadherin (*CDH5*) locus. Four ERG-bound enhancers distal to the TSS are indicated (E1-E4). (**B**) Nucleotide sequences of the ERG-bound distal VE-cadherin enhancer region E4 (chr16: 66,416,321 - 66,416,726). Clusters of predicted ERG DNA binding motifs are indicated in yellow. (**C**) Mutagenesis of the nine putative ERG binding motifs contained within region E4. Lower case letters indicate the base changes.

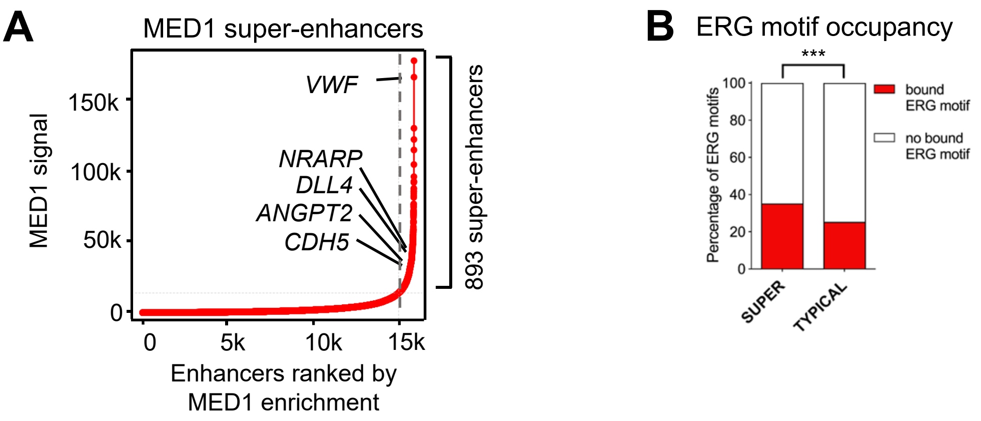

Online Figure III. SE characterization and MED1 defined SE. (**A**) Enhancer regions were defined by H3K4me1 and H3K27ac co-occupancy in HUVEC. Enhancer regions ranked by enrichment of MED1 ChIP-seq signal in rpm/bp. Super-enhancer clusters are shown to the right of the grey dashed line. (**B**) ERG motif occupancy at SE and typical enhancer regions defined by H3K27ac. The frequency of ERG binding on an identified ERG motif in SE (35%) versus typical enhancers (25%) is illustrated. SE have significantly higher ERG motif occupancy than typical enhancers; ***P<0.0001, Pearson's Chi-squared test. To note, typical enhancer had a higher frequency of ERG motifs per kb, however these motifs were less utilised than those in SE.

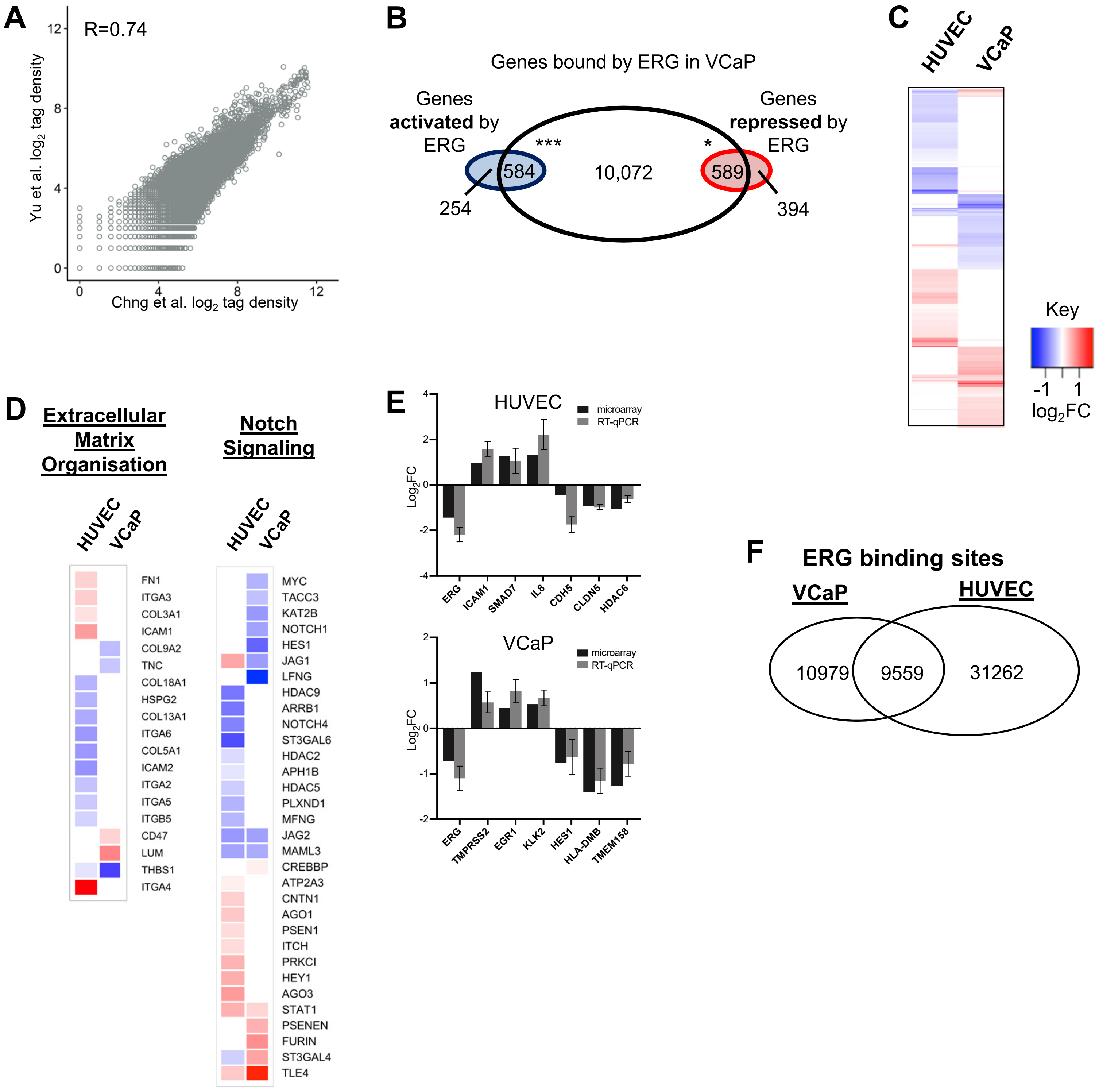

Online Figure IV. ChIP-seq analysis and transcriptome profiling shows lineage-specific ERG regulation of gene expression. (**A**) Correlation of two independent ERG ChIP-seq experiments in VCaP as log2 transformed ChIP tag density. Pearson correlation with R = 0.74, P<0.0001. (**B**) Comparison of global transcriptome profiling of ERG-deficient VCaP cells with ERG ChIP-seq data defines the expression pattern of genes bound by ERG. ERG activated and repressed genes are significantly more bound than expected by chance. *P<0.05 and ***P<0.0001, Fisher's exact test. (**C**) Heatmap of expression levels of the 3660 bound and regulated putative ERG target genes following ERG inhibition in HUVEC and in VCaP cells. (**D**) Heatmaps of ERG-bound target gene expression levels in ERG-deficient HUVEC and VCaP cells associated with extracellular matrix organization (left), and Notch signaling (right). (**E**) Validation of microarray transcriptome profiing by RT-qPCR. Gene expression as log_2FC between siCtl and siERG-treated HUVEC (top) or VCaP (bottom). RT-qPCR values are mean ± SEM, n = 4. (**F**) Venn diagram shows the overlapping ERG binding sites in HUVEC and VCaP prostate cancer cells.

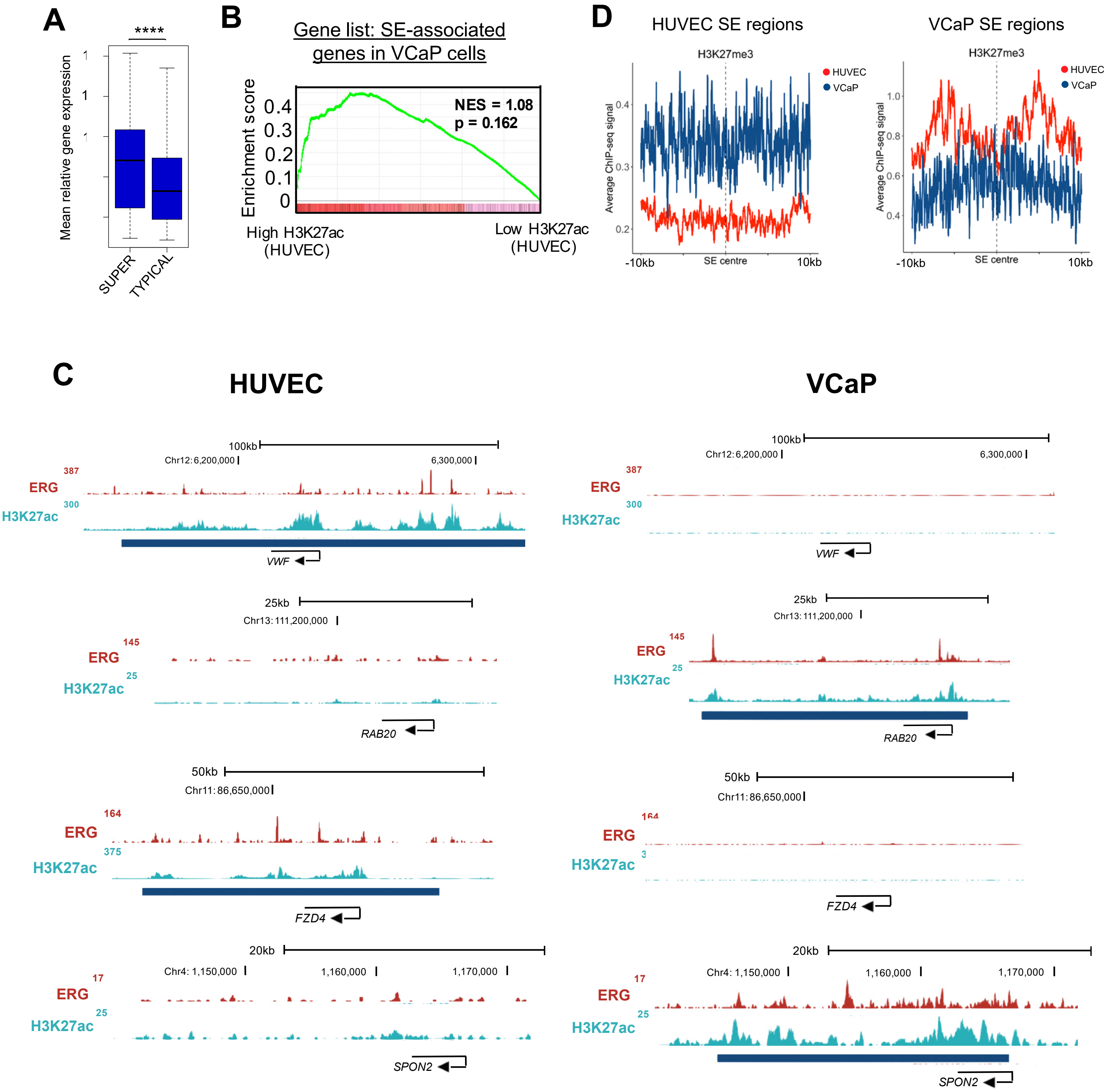

Online Figure V. Comparison of SE regions in ERG targets identified in HUVEC and VCaP cells. (**A**) Average gene expression from transcriptome profiling of VCaP cells shows significantly higher gene expression in SE than in typical enhancers. ****P<0.0001, Wilcoxon rank-sum test. (**B**) GSEA of the 208 genes associated with H3K27ac-identified SEs in VCaP cells compared with the ranked gene list from 917 H3K27ac-enriched SE in HUVEC. Normalised enrichment score (NES) = 1.08, not significant. (**C**) Gene tracks showing denhancer elements proximal to selected genes bound by ERG in HUVEC and VCaP cells. SE regions indicated as blue bars, alongside ERG binding and H3K27ac enrichment. (**D**) Aggregate plots of repressive histone modification H3K27me3 from HUVEC and VCaP cells in HUVEC SE (left) and VCaP SE (right). Plots are centered on the SE center.

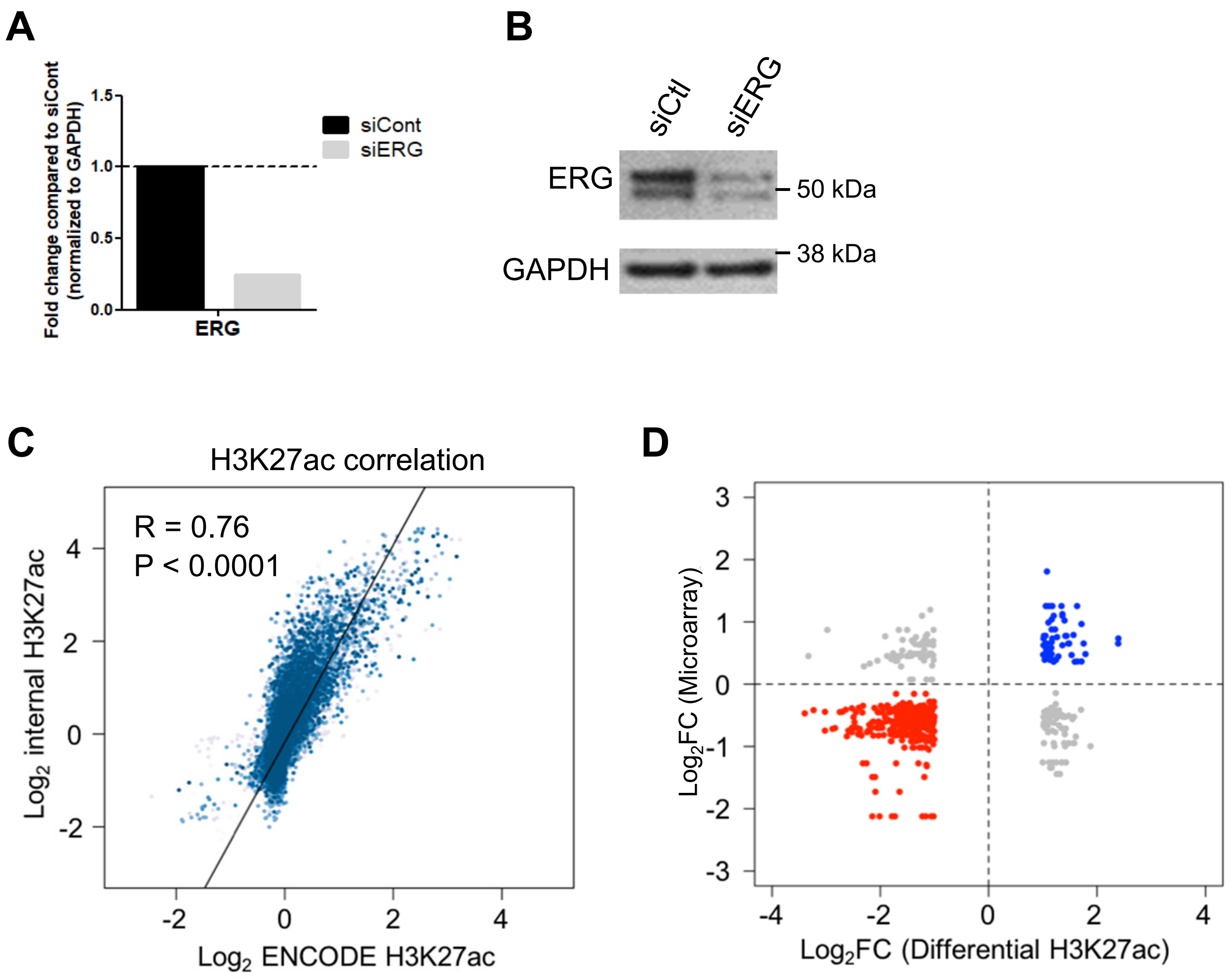

Online Figure VI. Histone acetylation in ERG-deficient HUVEC. (**A**) Validation of siRNA knockdown of ERG in total RNA from samples for H3K27ac ChIP-seq by RT-qPCR in siCtl and siERG treated HUVEC. (**B**) Validation of siRNA knockdown of ERG in samples for H3K27ac ChIP-seq by immunoblotting in siCtl and siERG-treated HUVEC. (**C**) Correlation plot comparing $\log_2$-normalized H3K27ac enrichment from publically-available data from ENCODE/Broad Institute to control siRNA-treated HUVEC. Each dot is a region representing a 50bp window. Pearson correlation with line of best fit R = 0.76, P<0.0001. (**D**) Scatterplot correlation between differential H3K27ac and differential gene expression. Genes associated with a loss in active region and downregulation in expression are shown in red, and genes associated with a gain in active region and upregulation in expression are in blue.

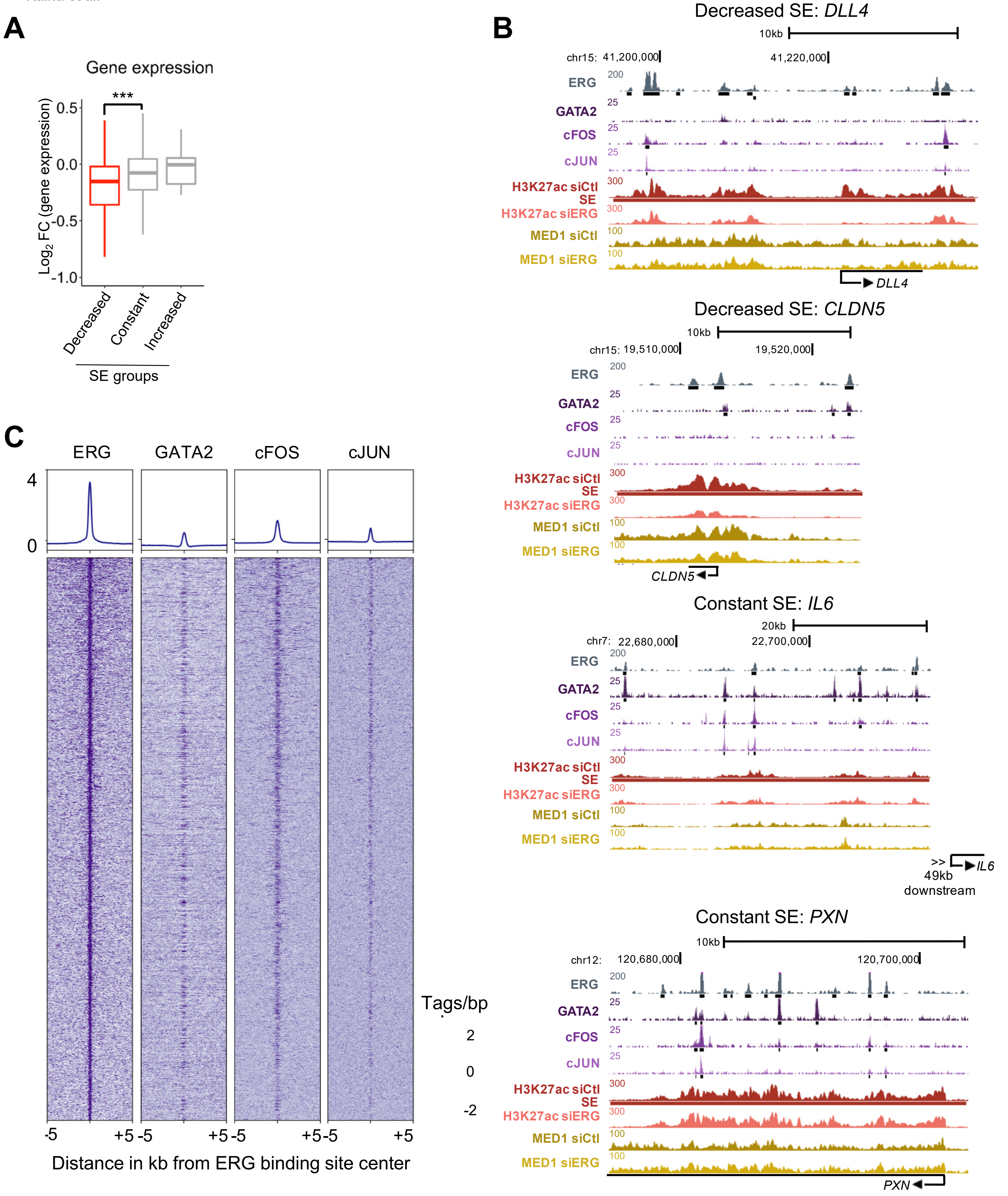

Online Figure VII. Transcription factor enrichment at ERG-bound sites in HUVEC. (**A**) Boxplot illustrating the $\log_2$FC from gene profiling microarray following ERG knockdown in SE-associated genes identified as decreased, increased or remaining constant from the differential SE analysis defined in ***B***. P < 0.0001, Kruskal-Wallis test and post-hoc test ***P < 0.001, Wilcoxon rank-sum test. (**B**) ChIP-seq profiles of SE that decreased following ERG depletion (*DLL4*, *CLDN5*) and SE that remained constant (*IL6*, *PXN*). The H3K27ac and MED1 signal in siCtl or siERG treated HUVEC is shown with the SE indicated under the H3K27ac siCtl from which it was identified. ERG and cooperative TF's GATA2, cFOS and cJUN binding profiles are represented and binding sites are black bars below each TF profile. **(C**) Heatmap of ERG ChIP-seq tag density showing co-occupancy of GATA2, cFOS and cJUN transcription factors around ±5kb from ERG peak centers. Rows are ERG-bound loci, repeated for each TF in columns.

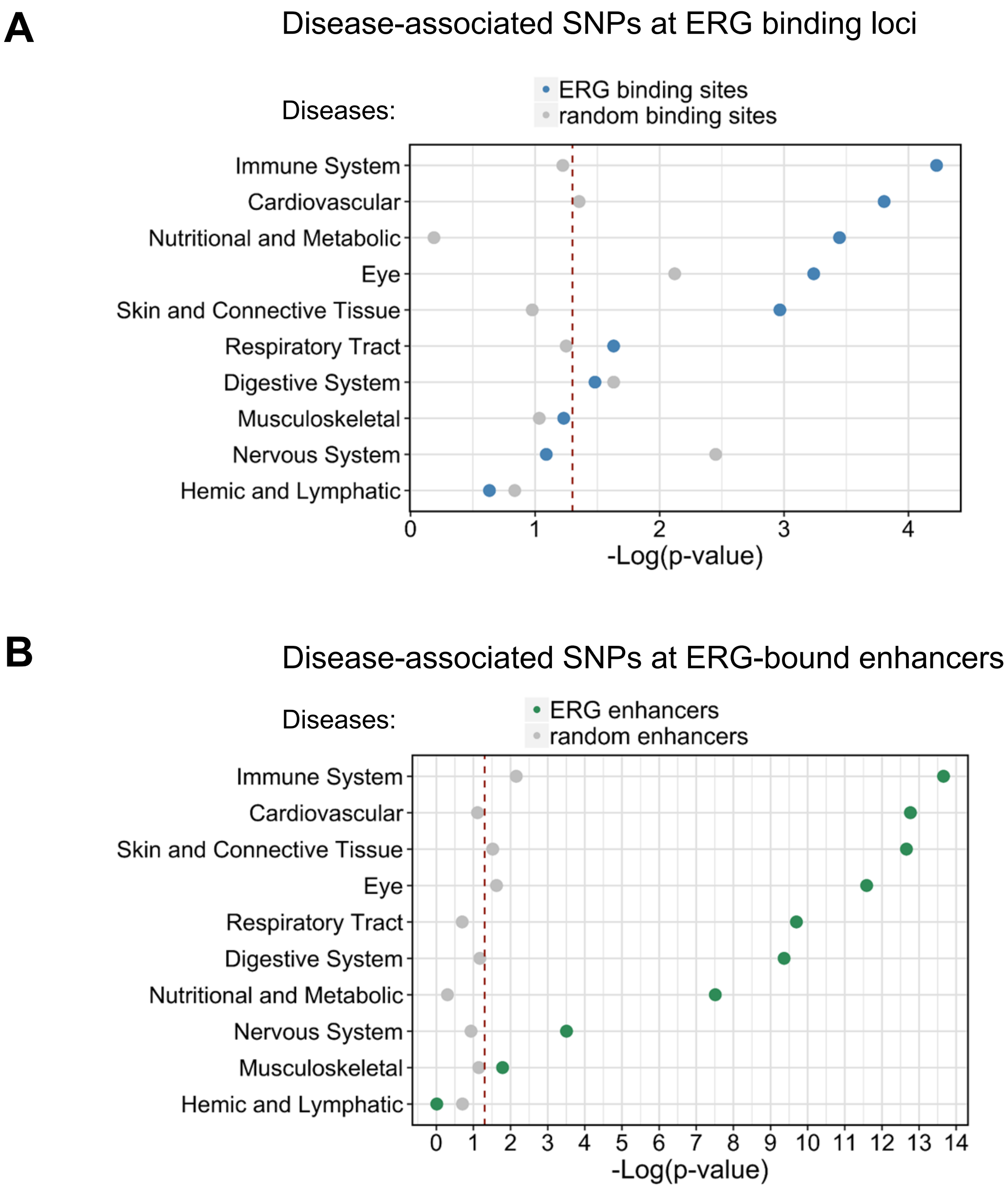

Online Figure VIII. ERG-dependent regulatory SNPs are associated with diseases of a vascular nature. Overlap of GWAS SNPs associated with disease trait classes within ERG binding loci (**A**; blue) or ERG-bound enhancers (**B**; green) versus chromosome and size matched random regions (gray). Significance of enrichment was calculated by binomial distribution test with red dashed line indicating P = 0.05. Both ERG binding sites and ERG-bound enhancers most highly prioritize immune system disease and cardiovascular disease associated SNPs.

	Super-enhancer ranking parameter				
	H3K27ac (HUVEC; ENCODE)	MED1	ERG	H3K27ac (VCaP)	H3K27ac (HUVEC; internal)
**Total super-enhancers**	917	893	1125	208	1015
**Associated genes**	822	840	995	199	949
**Mean size (bp)**	35508	45227	39708	13413	42843

Online Table I. Characteristics of all super-enhancers identified in this study from HUVEC and VCaP cells.

Activated in HUVEC and Repressed in VCap (84)
ABHD2, ABI2, ADAMTS1, ADARB1, ADRB1, AGAP2-AS1, AHNAK, AKAP13, AKAP7, AKT3, APLP2, ARHGAP24, ARHGAP28, ARMC8, ASTN2, ATP1B1, BMPR1B, CAMK2G, CAPZB, CASC4, CRIP2, CSRNP2, CUL4B, CYP2R1, CYTH1, DNAJB6, EFHC1, EFR3B, ELK4, ELL2, ENDOD1, ERCC1, EXT1, FAM107A, FAM49A, FEZ2, FLNB, FRAT1, GNA12, HOPX, HSD17B4, IFT43, IGF1R, IPPK, LIFR, LPP, MAP2K6, MIPEP, MSI2, MYLK, MYO5A, NBEA, NFE2L3, NLGN1, OLMALINC, PCCA, PDLIM5, PHF20L1, PIP4K2A, PKIA, PPP3CA, PTPRM, PXDN, RASSF2, RASSF3, RNF41, RTCA, RUFY3, SLC45A3, SNRK, SPATA2, SRPK2, ST3GAL4, SYNJ2, TM9SF3, TNFAIP8, UBE2E3, XPR1, YIPF1, ZC2HC1A, ZFP36L2, ZMAT3, ZNF671, ZSCAN2

Repressed in HUVEC and Activated in VCap (24)
ANXA1, BHLHE40, BTG3, CLN6, ECT2, ENC1, FBXO3, FTH1, GDF11, GOLIM4, IFIH1, IRF7, JAG1, KIF16B, MAP4K5, MFSD6, RAB11FIP1, RBBP8, RGS3, SPOPL, SPRED2, TCP11L1, TXNRD1, ZBTB20

Activated in HUVEC and VCap (88)
AGFG2, ANGPTL2, ANO6, APBA2, APP, ARHGAP27, ATL3, BCHE, BTG3, C15orf54, CD58, CENPF, COLGALT1, CSRP1, CTDSPL, DAB2, DCLK1, DHX37, DOCK1, DYM, ELAVL2, EPAS1, ERG, ERICH1, ETV6, FAM172A, FAM198B, FCHSD2, FOCAD, FRMD3, FYN, FZD4, HSD17B11, HSPA4, IGFBP3, IL17D, ITPRIP, KCTD15, KHDRBS3, KIAA0368, KIAA0586, LIG1, LPXN, MAML3, MAN2C1, MAP2K5, MERTK, MRPS6, MTR, NFE2L3, NFIA, NOP14, NRARP, NRCAM, NRP1, OAF, OSBPL10, OSBPL8, PAM, PEX10, PHF19, PRSS23, PSMG4, RALGAPA2, RCC1, RGS12, RGS3, RGS7, RIMKLB, RNF135, RSU1, SAMD13, SH3KBP1, SHISA2, SIPA1L2, SLC22A23, SMYD3, ST6GAL1, SVIL, THBS1, TRNP1, TSHZ1, XPOT, ZDHHC13, ZDHHC23, ZNF618, ZNF638, ZNF704

Repressed in HUVEC and VCap (53)
ACAA1, AQP3, ARID5B, AZIN1, BAIAP2L1, BCAS4, BROX, CCDC134, CCDC136, CDC42EP3, CDKN1A, DUSP10, EGR1, FAM117B, GOLGA2, GPX3, HERC5, HIF1AN, HIST1H2AC, HIST1H2BD, HIST1H2BK, HIST1H4H, IP6K2, IRS2, KDM5B, KIF5C, KRAS, LIMS1, NSD1, NXPE3, OCIAD2, PASK, PFKFB4, PPM1K, RCOR2, RDH10, SC5D, SLC38A4, SLC39A6, SPPL2A, STAT1, STK38, TIPARP, TLE4, TMEFF2, TP53BP1, TSPAN8, TTC12, TTPAL, ZBTB8OS, ZCCHC6, ZKSCAN3, ZSCAN31

Online Table II. Shared ERG bound targets in HUVEC and VCaP cells.

Name	Orientation	Oligonucleotide sequences
ICAM1 promoter	Forward	TTGGAAATTCCGGAGCTGAA
	Reverse	TGCTGCAGTTATTTCCGGACT
VE-Cadherin promoter	Forward	AGCCAGCCCAGCCCTCAC
	Reverse	CCTGTCAGCCGACCGTCTTTG
VE-Cadherin 3'-UTR	Forward	GGTAAGGCTGGTGAGGTC
	Reverse	CTTGGAGTGGAGTATGGAGTTG
Gene desert on chr2	Forward	TGAATAAGCCAATGAAACAATGACA
	Reverse	TGAAACATAGTATGGGTGGCAACT
Gene desert on chr16	Forward	GTCTCTTTCTTGTTTTTAAGCTGGG
	Reverse	TGAGCTCATTGAGACATTTGG
CLDN5 enhancer (E1)	Forward	CCGGAAGCCAACTTGGAGTTT
	Reverse	GTGCAGAAGAATGCCCGGAA
DLL4 enhancer (E3)	Forward	GTTTCCTGCGGGTTATTTTT
	Reverse	CTTTCCAAAGGAGCGGAAT
VWF enhancer	Forward	AGGGGATTGGCCTCCTTTTA
	Reverse	CCATTTCCTTTCATTGTTTCC
CLDN5 E2	Forward	TCCTGCATCCCTGACCACTG
	Reverse	CTGGATGCTGCTCACATCGT
DLL4 E1	Forward	GCAGGTTGAGGGTGAATGGT
	Reverse	TGCCCAAGCACCAGAACTTT
DLL4 E2	Forward	CCCCAGGACCTATCCCAAGT
	Reverse	CACCATTTAGCAGAGCCGGA
DLL4 E4	Forward	CATGTGGGGGACAGGTAGGA
	Reverse	GCTCCCCATCTAGTGCATCA
IL6 E1	Forward	TGACTGAGCAAACCCATTTTCC
	Reverse	TCCTTATGTGGGAAGGTATGGC
IL6 E2	Forward	AGATTCCTCCACATTTGCCCA
	Reverse	GGCAACTCCAAGCCAGAACA
IL6 E3	Forward	GGTCACGCCACAACTGGAAT
	Reverse	CCATTCCTCACACCCACTGTT
PXN E1	Forward	GCCTCTCACCCCTGCTAATC
	Reverse	TTTGTTCGGGTCTCTGTGGG
PXN E2	Forward	GCATCACGTAGCAACAGAGC
	Reverse	GGTGTTGCTGACACATTCCG
PXN E3	Forward	GGTGGAGTAAAGCGTGAGCA
	Reverse	TGGGTGTAATGCCTGCCTTC

Online Table III. Oligonucleotides used for ChIP-qPCR in this study

Name	Orientation	Oligonucleotide sequences
ERG	Forward	GGAGTGGGCGGTGAAAGA
	Reverse	AAGGATGTCGGCGTTGTAGC
GAPDH	Forward	CAAGGTCATCCATGACAACTTTG
	Reverse	GGGCCATCCACAGTCTTCTG
ICAM1	Forward	CTGAAACTTGCTGCCTATTGGG
	Reverse	ACACATGTCTATGGAGGGCCA
SMAD7	Forward	TGGTGTGCTGCAACCCCCATCA
	Reverse	GCACAGCATCTGGACAGTCTGC
IL8	Forward	AGGAACCATCTCACTGTGTG
	Reverse	GGCATCTTCACTGATTCTTG
CDH5	Forward	AGCCAGCCCAGCCCTCAC
	Reverse	CCTGTCAGCCGACCGTCTTTG
CLDN5	Forward	GGGAAAGCCCCTGTGCCACC
	Reverse	TCCAGCCCCGCTCTGAGTCC
HDAC6	Forward	CTAGCAGACACCTACGACTCAG
	Reverse	GCAATAGCCATCCATAAGACTGTG
TMPRSS2	Forward	CGGATGCACCTCGTAGACAG
	Reverse	TCACCACCAGCTATTGGACC
EGR1	Forward	CTTCAACCCTCAGGCGGACA
	Reverse	GGAAAAGCGGCCAGTATAGGT
KLK2	Forward	GGTGGCTGTGTACAGTCATGGAT
	Reverse	TGTCTTCAGGCTCAAACAGGTTG
HES1	Forward	AGAAAGATAGCTCGCGGCATT
	Reverse	CGGAGGTGCTTCACTGTCAT
HLA-DMB	Forward	GCTGGCCACTCTAGTTACACTC
	Reverse	GAGGTCCCCCAAGTTGCTAA
TMEM158	Forward	GGCTGAACCGTAAGCCCATT
	Reverse	CTCCACACCACGATGACCAG
VE-Cadherin (Enhancer 1)	Forward	ACTCGCTAGCGTCGACATCAAGAGGTGGGAGGGATT
	Reverse	ATCGTCGACCGTCGACACCACATGTGCTGTGTTCGT
VE-Cadherin (Enhancer 2)	Forward	ACTCGCTAGCGTCGACGAGGGGTAGGGGAGGTGTAT
	Reverse	ATCGTCGACGTCGACCCTCTCTGAGCTTCACTGT
VE-Cadherin (Enhancer 4)	Forward	ACTCGCTAGCGTCGACTAGGAGACCAGGACAGAGC
	Reverse	ATCGTCGACCGTCGACGACTTGCTCCCATTGTCCAG
VE-Cadherin E4 mutant 1	Forward	GGAGAATGCCGCCCTACCAAAACCAGCATTCCTG
VE-Cadherin E4 mutant 2	Forward	CCGCCCTACCAAAACCAGCATTGGTGACTCTCAACAC
VE-Cadherin E4 mutant 3	Forward	GCGAGCTCTGTGTCATACACCAAAATGTCGTCAATCCACC
VE-Cadherin E4 mutant 4	Forward	TGTCGTCAATCCACCTGCATGGTTCCCGGTGGG
VE-Cadherin E4 mutant 5	Forward	TAAAAAGAATGTCTGACCTCAACCAAAATCTATGGAGTGAGTCAGG
VE-Cadherin E4 mutant 6	Forward	AATCTATGGAGTGAGTCAACCAAAGTCCGTAGAAAGTGGG
VE-Cadherin E4 mutant 7	Forward	GGGTTTCTAGCAGCACCAAGAACGCCAGGCCT
VE-Cadherin E4 mutant 8	Forward	GGCTGGGTCTCCGACCAACCACAAGGGGTC
VE-Cadherin E4 mutant 9	Forward	GAGGATGTAATCTGAGGGAACCAAGTCCCTGTTTATCAAGG

Online Table IV. Oligonucleotides used for RT-qPCR and validation of the *CDH5* enhancers

Online Table V: Super-enhancers identified by enrichment of H3K27ac in HUVEC. Ordered by super-enhancer ranking. Chromosome (chr), start and end indicates genomic position on the GRCh37/hg19 assembly. Super-enhancer-associated gene symbols and description are indicated. H3K27ac signal is ChIP-seq read density times length of stitched enhancer. This table spans 21 pages.

Rank	chr	start	end	Gene	Gene description	H3K27ac signal
1	chr12	6150598	6345991	VWF	von Willebrand factor	398660.3379
2	chr15	67356712	67473258	SMAD3	SMAD family member 3	295280.9456
3	chr2	20761375	20819862	HS1BP3	HCLS1 binding protein 3	274350.8196
4	chr3	194817758	194946736	XXYLT1-AS2	XXYLT1 antisense RNA 2	262805.5728
5	chr1	22219550	22302670	HSPG2	heparan sulfate proteoglycan 2	232519.888
6	chr3	171474760	171595496	TMEM212	transmembrane protein 212	215380.9504
7	chr22	36709337	36796144	MYH9	myosin, heavy chain 9, non-muscle	201001.6085
8	chr6	158422671	158493989	SYNJ2	synaptojanin 2	199155.515
9	chr6	151320145	151393521	MTHFD1L	methylenetetrahydrofolate dehydrogenase (NADP+ dependent) 1-like	196809.1072
10	chr12	109195156	109252637	SSH1	slingshot protein phosphatase 1	192607.3348
11	chr3	149242874	149379253	WWTR1-AS1	WWTR1 antisense RNA 1	188148.4684
12	chr4	7779369	7921553	AFAP1	actin filament associated protein 1	188038.34
13	chr16	66390760	66423157	CDH5	cadherin 5	185220.1284
14	chr20	36700423	36802692	TGM2	transglutaminase 2	183818.3006
15	chr1	208371601	208419074	PLXNA2	plexin A2	177463.5686
16	chr8	128842258	128964124	PVT1	Pvt1 oncogene (non-protein coding)	172623.189
17	chr11	33708994	33760268	C11orf91	chromosome 11 open reading frame 91	166455.9136
18	chr7	100736576	100783380	SERPINE1	serpin peptidase inhibitor, clade E (nexin, plasminogen activator inhibitor type 1), member 1	162625.1784
19	chr12	96753048	96844725	CDK17	cyclin-dependent kinase 17	162469.9794
20	chr5	172279904	172339762	ERGIC1	endoplasmic reticulum-golgi intermediate compartment 1	162113.4214
21	chr2	36579345	36731285	LOC100288911	uncharacterized LOC100288911	160266.312
22	chr1	235095440	235149137	SNORA14B	small nucleolar RNA, H/ACA box 14B	157740.3072
23	chr7	27168902	27221257	HOXA-AS3	HOXA cluster antisense RNA 3	157499.5465
24	chr14	74178270	74230072	MIR4505	microRNA 4505	157483.2602
25	chr9	116339406	116421278	RGS3	regulator of G-protein signaling 3	156350.9584
26	chr20	45931031	45991894	LOC100131496	uncharacterized LOC100131496	156241.4073
27	chr3	129294413	129347691	PLXND1	plexin D1	151943.5282
28	chr19	13946293	13965188	LOC284454	uncharacterized LOC284454	151707.955
29	chr8	17503830	17557739	MTUS1	microtubule associated tumor suppressor 1	150761.9094
30	chr9	130587945	130645966	MIR4672	microRNA 4672	150721.1517
31	chr7	130565017	130607898	NA	NA	143994.398
32	chr15	42209440	42265576	EHD4	EH domain containing 4	143865.3408
33	chr12	120661895	120704645	PXN	paxillin	142866.225
34	chr13	97873415	97962677	MBNL2	muscleblind like splicing regulator 2	142863.831
35	chr11	12177950	12263570	MICAL2	microtubule associated monooxygenase, calponin and LIM domain containing 2	141975.084
36	chr15	96859967	96903973	MIR1469	microRNA 1469	141100.8384
37	chr17	62604109	62746017	SMURF2	SMAD specific E3 ubiquitin protein ligase 2	140829.4992
38	chr12	52277472	52322378	ANKRD33	ankyrin repeat domain 33	140385.1372
39	chr6	148683214	148788098	SASH1	SAM and SH3 domain containing 1	139254.4868

Rank	chr	start	end	Gene	Gene description	H3K27ac signal
40	chr10	95162521	95242935	MYOF	myoferlin	138054.7552
41	chr21	39806397	39879807	ERG	v-ets avian erythroblastosis virus E26 oncogene homolog	133716.315
42	chr1	94485752	94539432	ABCA4	ATP binding cassette subfamily A member 4	133609.52
43	chr3	11195553	11281478	HRH1	histamine receptor H1	127598.625
44	chr1	156055108	156101097	LMNA	lamin A/C	127403.3267
45	chr6	11585122	11619428	TMEM170B	transmembrane protein 170B	126873.8798
46	chr3	57990579	58107732	FLNB	filamin B	126513.5247
47	chr16	81710741	81764822	LOC100129617	uncharacterized LOC100129617	125786.9979
48	chr1	39567767	39680972	MACF1	microtubule-actin crosslinking factor 1	125283.9735
49	chr6	11193176	11237658	NEDD9	neural precursor cell expressed, developmentally down-regulated 9	123860.129
50	chr17	75100867	75126400	SEC14L1	SEC14 like lipid binding 1	123160.9788
51	chr11	128547516	128601295	SENCR	smooth muscle and endothelial cell enriched migration/differentiation-associated long non-coding RNA	123003.3288
52	chr10	101663548	101770893	DNMBP-AS1	DNMBP antisense RNA 1	122265.955
53	chr6	112522289	112576802	LAMA4	laminin subunit alpha 4	122130.9252
54	chr1	85762318	85834616	BCL10	B-cell CLL/lymphoma 10	120137.5866
55	chr17	2073163	2120642	SMG6	SMG6 nonsense mediated mRNA decay factor	119447.6682
56	chr9	127019384	127073952	NEK6	NIMA related kinase 6	119040.092
57	chr12	96586107	96659334	ELK3	ELK3, ETS transcription factor	118408.059
58	chr2	235859137	235915831	SH3BP4	SH3-domain binding protein 4	118229.6676
59	chr20	1301744	1326255	SDCBP2-AS1	SDCBP2 antisense RNA 1	116843.937
60	chr14	55087612	55159666	SAMD4A	sterile alpha motif domain containing 4A	115488.1512
61	chr19	39137654	39201612	ACTN4	actinin alpha 4	114753.4436
62	chr1	23879628	23908578	MDS2	myelodysplastic syndrome 2 translocation associated	114546.465
63	chr11	65237697	65276784	MALAT1	metastasis associated lung adenocarcinoma transcript 1 (non-protein coding)	113668.9047
64	chr10	104354881	104418662	SUFU	SUFU negative regulator of hedgehog signaling	113434.5085
65	chr3	15670418	15691589	ANKRD28	ankyrin repeat domain 28	113417.2812
66	chr5	34654263	34720278	RAI14	retinoic acid induced 14	113327.9505
67	chr1	68129344	68281895	GADD45A	growth arrest and DNA damage inducible alpha	112338.5564
68	chr12	111833864	111887883	SH2B3	SH2B adaptor protein 3	111932.7699
69	chr9	38006679	38080219	SHB	Src homology 2 domain containing adaptor protein B	110699.762
70	chr15	60650645	60716895	ANXA2	annexin A2	109531.125
71	chr10	33501796	33577318	NRP1	neuropilin 1	109401.1692
72	chr21	33774813	33873638	EVA1C	eva-1 homolog C (C. elegans)	107966.3125
73	chr5	14141967	14238765	TRIO	trio Rho guanine nucleotide exchange factor	107736.174
74	chr2	9340401	9430319	ASAP2	ArfGAP with SH3 domain, ankyrin repeat and PH domain 2	106714.6824
75	chr3	5017629	5038735	BHLHE40-AS1	BHLHE40 antisense RNA 1	106684.4982
76	chr10	104532541	104577425	WBP1L	WW domain binding protein 1-like	106137.1948
77	chr6	3795352	3834058	FAM50B	family with sequence similarity 50 member B	105760.2744
78	chr2	54772239	54842985	SPTBN1	spectrin beta, non-erythrocytic 1	105192.2274
79	chr12	54796490	54827997	ITGA5	integrin subunit alpha 5	104669.4047
80	chr19	13260177	13284020	STX10	syntaxin 10	104227.2902
81	chr3	171842338	171875238	FNDC3B	fibronectin type III domain containing 3B	103786.34
82	chr8	23172880	23216185	LOC100507156	uncharacterized LOC100507156	103542.255
83	chr21	46711695	46741729	LOC642852	uncharacterized LOC642852	102016.4878
84	chr13	111022722	111096837	COL4A2	collagen type IV alpha 2	101737.6605

Rank	chr	start	end	Gene	Gene description	H3K27ac signal
85	chr1	16463917	16509856	EPHA2	EPH receptor A2	101042.8305
86	chr9	130278993	130343647	FAM129B	family with sequence similarity 129 member B	100246.027
87	chr3	30645151	30748081	TGFBR2	transforming growth factor beta receptor II	100243.527
88	chr2	201979149	202057923	CFLAR-AS1	CFLAR antisense RNA 1	99523.0716
89	chr1	172851335	172961123	TNFSF18	tumor necrosis factor superfamily member 18	99149.5428
90	chr1	66796708	66841160	PDE4B	phosphodiesterase 4B	97483.236
91	chr20	31049560	31090763	NOL4L	nucleolar protein 4-like	97094.8695
92	chr1	21577891	21632742	LOC100506801	uncharacterized LOC100506801	97080.7849
93	chr2	105984844	106027805	FHL2	four and a half LIM domains 2	97053.1951
94	chr18	3579034	3608389	DLGAP1-AS1	DLGAP1 antisense RNA 1	96475.2075
95	chr21	37793876	37861255	CLDN14	claudin 14	96358.7079
96	chr6	52353440	52443435	TRAM2-AS1	TRAM2 antisense RNA 1 (head to head)	96078.662
97	chr14	75397956	75493420	PGF	placental growth factor	95301.7112
98	chr10	13850061	13937676	FRMD4A	FERM domain containing 4A	94992.183
99	chr19	6055778	6069260	RFX2	regulatory factor X2	94421.187
100	chr14	75075506	75089712	LTBP2	latent transforming growth factor beta binding protein 2	93876.0892
101	chr7	1552435	1579933	MAFK	v-maf avian musculoaponeurotic fibrosarcoma oncogene homolog K	93438.204
102	chr8	17458742	17478982	PDGFRL	platelet derived growth factor receptor like	93369.144
103	chr9	116261612	116291620	RGS3	regulator of G-protein signaling 3	92919.772
104	chr1	223885566	223965818	CAPN2	calpain 2	92458.3292
105	chr5	151038387	151079942	SPARC	secreted protein acidic and cysteine rich	92376.765
106	chr8	142126368	142175949	DENND3	DENN domain containing 3	92062.0008
107	chr9	139547268	139569757	MIR126	microRNA 126	91995.7523
108	chr16	75270190	75308198	BCAR1	breast cancer anti-estrogen resistance 1	91861.5352
109	chr22	38596816	38638062	MAFF	v-maf avian musculoaponeurotic fibrosarcoma oncogene homolog F	91718.7302
110	chr10	74055316	74098640	DNAJB12	DnaJ heat shock protein family (Hsp40) member B12	91041.0536
111	chr17	38467102	38507762	RARA	retinoic acid receptor alpha	90805.978
112	chr19	41803142	41835555	HNRNPUL1	heterogeneous nuclear ribonucleoprotein U-like 1	90526.2677
113	chr7	22599345	22651757	LOC100506178	uncharacterized LOC100506178	90436.906
114	chr4	41121390	41220028	APBB2	amyloid beta precursor protein binding family B member 2	90352.408
115	chr19	45925964	45982895	ERCC1	excision repair cross-complementation group 1	89916.8214
116	chr3	39186176	39223111	CSRNP1	cysteine and serine rich nuclear protein 1	89220.186
117	chr10	17027311	17074366	CUBN	cubilin	87672.876
118	chr4	141004647	141038815	MAML3	mastermind like transcriptional coactivator 3	86981.4776
119	chr12	89737986	89786004	DUSP6	dual specificity phosphatase 6	86946.1926
120	chr9	112777122	112836210	AKAP2	A-kinase anchoring protein 2	86764.8192
121	chr20	49086817	49191105	PTPN1	protein tyrosine phosphatase, non-receptor type 1	86705.0432
122	chr2	85960385	86002659	ATOH8	atonal bHLH transcription factor 8	86530.6506
123	chr21	27256073	27360856	APP	amyloid beta precursor protein	86246.8873
124	chr20	30248993	30312218	BCL2L1	BCL2 like 1	86125.095
125	chr7	27134521	27166245	HOTAIRM1	HOXA transcript antisense RNA, myeloid-specific 1	86035.488
126	chr2	28608599	28647824	FOSL2	FOS like antigen 2	85569.3375
127	chr7	107610897	107671816	LAMB1	laminin subunit beta 1	85122.1187
128	chr12	13232121	13290136	GSG1	germ cell associated 1	84493.046
129	chr9	134508244	134558764	RAPGEF1	Rap guanine nucleotide exchange factor 1	84262.308

Rank	chr	start	end	Gene	Gene description	H3K27ac signal
130	chr5	77779125	77831452	SCAMP1	secretory carrier membrane protein 1	83990.0677
131	chr19	38488345	38536099	SIPA1L3	signal-induced proliferation-associated 1 like 3	83187.468
132	chr22	23520612	23586038	BCR	breakpoint cluster region	83058.307
133	chr17	32561260	32587095	CCL2	C-C motif chemokine ligand 2	82545.4085
134	chr1	45270550	45280207	BTBD19	BTB domain containing 19	82518.0993
135	chr17	17584029	17631609	RAI1	retinoic acid induced 1	82322.916
136	chr16	86932780	86987079	FOXL1	forkhead box L1	82197.8262
137	chr6	7953617	8007754	PIP5K1P1	phosphatidylinositol-4-phosphate 5-kinase, type I, pseudogene 1	81519.4946
138	chr12	105820885	105884788	C12orf75	chromosome 12 open reading frame 75	81386.8608
139	chr10	31984188	31999298	ARHGAP12	Rho GTPase activating protein 12	81380.949
140	chr8	6384511	6460648	ANGPT2	angiopoietin 2	81299.0886
141	chr6	11646491	11657976	ADTRP	androgen dependent TFPI regulating protein	81030.1205
142	chr22	39566666	39581677	CBX7	chromobox 7	80738.1646
143	chr1	165851021	165881423	MIR3658	microRNA 3658	80620.0236
144	chr16	56638226	56649793	MT1A	metallothionein 1A	80388.3366
145	chr21	33886213	33898952	TCP10L	t-complex 10-like	80055.6977
146	chr14	77490511	77527029	IRF2BPL	interferon regulatory factor 2 binding protein-like	79448.5608
147	chr18	55435265	55514768	ATP8B1	ATPase phospholipid transporting 8B1	79447.3479
148	chr10	3109332	3148029	PFKP	phosphofructokinase, platelet	79340.4591
149	chr10	106080794	106103813	ITPRIP	inositol 1,4,5-trisphosphate receptor interacting protein	79185.36
150	chr16	20908010	20924292	ERI2	ERI1 exoribonuclease family member 2	78163.3692
151	chr13	114872138	114898518	RASA3	RAS p21 protein activator 3	77346.16
152	chr11	65183244	65201127	NEAT1	nuclear paraspeckle assembly transcript 1 (non-protein coding)	77263.5015
153	chr20	48635583	48665653	TRERNA1	translation regulatory long non-coding RNA 1	76783.745
154	chr9	67292852	67304232	AQP7P1	aquaporin 7 pseudogene 1	76623.816
155	chr1	234852748	234882254	LINC01132	long intergenic non-protein coding RNA 1132	76520.8604
156	chr12	95997866	96033544	PGAM1P5	phosphoglycerate mutase 1 pseudogene 5	76058.3604
157	chr2	238580987	238654797	LRRFIP1	leucine rich repeat (in FLII) interacting protein 1	75891.442
158	chr22	38567054	38583949	PLA2G6	phospholipase A2 group VI	75846.7235
159	chr12	12928214	12994430	DDX47	DEAD-box helicase 47	75764.3472
160	chr11	64618415	64647572	EHD1	EH domain containing 1	75761.5488
161	chr13	28895394	28933838	FLT1	fms related tyrosine kinase 1	75753.902
162	chr1	112929782	112950521	CTTNBP2NL	CTTNBP2 N-terminal like	75655.872
163	chr10	123870693	123910081	TACC2	transforming acidic coiled-coil containing protein 2	75266.5292
164	chr12	124851552	124879957	NCOR2	nuclear receptor corepressor 2	74932.39
165	chr5	139670398	139727157	HBEGF	heparin binding EGF like growth factor	74921.88
166	chr6	30707093	30723131	FLOT1	flotillin 1	74839.7232
167	chr1	234734161	234769328	LINC00184	long intergenic non-protein coding RNA 184	74838.8927
168	chr3	50263544	50299319	GNAI2	G protein subunit alpha i2	74340.45
169	chr1	36807484	36854230	STK40	serine/threonine kinase 40	74059.6878
170	chr1	159878907	159896233	TAGLN2	transgelin 2	73895.39
171	chr19	4367253	4404218	CHAF1A	chromatin assembly factor 1 subunit A	73885.642
172	chr9	140188102	140217008	NRARP	NOTCH-regulated ankyrin repeat protein	73808.5804
173	chr3	193850430	193861638	HES1	hes family bHLH transcription factor 1	73264.4544
174	chr18	3245189	3284288	MYL12B	myosin light chain 12B	73009.5627

Rank	chr	start	end	Gene	Gene description	H3K27ac signal
175	chr11	69059906	69089967	MYEOV	myeloma overexpressed	72930.9921
176	chr10	112134837	112181444	SMNDC1	survival motor neuron domain containing 1	72781.4912
177	chr8	13208329	13242131	DLC1	DLC1 Rho GTPase activating protein	72752.0446
178	chr12	13348076	13366140	EMP1	epithelial membrane protein 1	72734.696
179	chr1	38460584	38496741	FHL3	four and a half LIM domains 3	72661.1072
180	chr1	19232814	19284138	IFFO2	intermediate filament family orphan 2	72577.2684
181	chr6	36787358	36797318	CPNE5	copine 5	72575.532
182	chr22	31605437	31641698	LIMK2	LIM domain kinase 2	72449.478
183	chr16	81505802	81561494	CMIP	c-Maf inducing protein	72433.0152
184	chr13	110949666	111001529	COL4A1	collagen type IV alpha 1	72385.1891
185	chr16	27245202	27272126	FLJ21408	uncharacterized LOC400512	72355.5576
186	chr7	102510132	102534051	LRRC17	leucine rich repeat containing 17	72350.1912
187	chr10	126723028	126775223	MIR4296	microRNA 4296	72128.2705
188	chr5	34570828	34629550	RAI14	retinoic acid induced 14	72128.2326
189	chr9	73020184	73055287	KLF9	Kruppel-like factor 9	72048.9075
190	chr10	14587123	14660254	FAM107B	family with sequence similarity 107 member B	71829.2682
191	chr1	156445853	156476372	MEF2D	myocyte enhancer factor 2D	71609.7816
192	chr16	80964376	80983359	CENPN	centromere protein N	71526.0457
193	chr19	2528343	2556675	GADD45B	growth arrest and DNA damage inducible beta	71487.3024
194	chr9	139401748	139446529	MIR4673	microRNA 4673	71340.6111
195	chr8	142007360	142050583	PTK2	protein tyrosine kinase 2	71084.5458
196	chr7	39626787	39674552	RALA	v-ral simian leukemia viral oncogene homolog A (ras related)	70911.919
197	chr10	17240970	17282024	TRDMT1	tRNA aspartic acid methyltransferase 1	70653.934
198	chr18	33035450	33078382	INO80C	INO80 complex subunit C	70532.9828
199	chr17	6922442	6937318	MIR497HG	mir-497-195 cluster host gene	70350.0916
200	chr3	11589842	11646558	VGLL4	vestigial like family member 4	70293.8104
201	chr15	39869932	39892833	THBS1	thrombospondin 1	70244.2373
202	chr20	49969845	50011168	MIR3194	microRNA 3194	70108.6018
203	chr17	45299686	45394524	ITGB3	integrin subunit beta 3	70094.7658
204	chr9	676520	717070	KANK1	KN motif and ankyrin repeat domains 1	69916.31
205	chr9	130522282	130534586	SH2D3C	SH2 domain containing 3C	69758.7584
206	chr2	161060300	161100546	ITGB6	integrin subunit beta 6	69645.703
207	chr5	172174129	172206194	DUSP1	dual specificity phosphatase 1	69645.18
208	chr6	2846238	2878706	MIR4645	microRNA 4645	69549.7028
209	chr16	77598161	77631922	NUDT7	nudix hydrolase 7	69547.66
210	chr22	39624933	39689626	PDGFB	platelet derived growth factor subunit B	69519.0978
211	chr2	207985589	208032776	KLF7	Kruppel-like factor 7 (ubiquitous)	69374.3274
212	chr9	112667356	112686699	PALM2-AKAP2	PALM2-AKAP2 readthrough	69352.3922
213	chr3	43427720	43461413	SNRK-AS1	SNRK antisense RNA 1	68986.4175
214	chr11	124615820	124643202	VSIG2	V-set and immunoglobulin domain containing 2	68575.4808
215	chr22	19860871	19882203	TXNRD2	thioredoxin reductase 2	68543.9824
216	chr17	76369948	76393418	PGS1	phosphatidylglycerophosphate synthase 1	68335.252
217	chr16	89364100	89403432	LOC100287036	uncharacterized LOC100287036	68229.2204
218	chr10	129765110	129817637	PTPRE	protein tyrosine phosphatase, receptor type E	68122.2663
219	chr1	94749745	94801650	ARHGAP29	Rho GTPase activating protein 29	68073.4075

Rank	chr	start	end	Gene	Gene description	H3K27ac signal
220	chr3	43219474	43241913	POMGNT2	protein O-linked mannose N-acetylglucosaminyltransferase 2 (beta 1,4-)	67920.6091
221	chr20	30174985	30200259	MIR3193	microRNA 3193	67842.9982
222	chr6	33736176	33758026	LEMD2	LEM domain containing 2	67667.265
223	chr1	150530393	150553301	MCL1	myeloid cell leukemia 1	67500.7128
224	chr16	16076415	16124453	ABCC1	ATP binding cassette subfamily C member 1	67373.295
225	chr16	82659979	82692499	CDH13	cadherin 13	67092.012
226	chr17	1964953	1995350	SMG6	SMG6 nonsense mediated mRNA decay factor	67019.3056
227	chr15	39520384	39595133	C15orf54	chromosome 15 open reading frame 54	66840.5558
228	chr22	47129753	47155769	CERK	ceramide kinase	66489.0912
229	chr11	9707628	9750951	SWAP70	SWAP switching B-cell complex 70kDa subunit	66392.4975
230	chr12	125135279	125181009	NCOR2	nuclear receptor corepressor 2	66308.5
231	chr8	103799507	103824070	AZIN1	antizyme inhibitor 1	65909.8979
232	chr8	8989929	9009398	PPP1R3B	protein phosphatase 1 regulatory subunit 3B	65776.0165
233	chr12	50037880	50069731	FMNL3	formin like 3	65714.9832
234	chr6	129943794	130032337	ARHGAP18	Rho GTPase activating protein 18	65698.906
235	chr10	112247002	112290867	DUSP5	dual specificity phosphatase 5	65670.2915
236	chr8	8923227	8947797	MIR4660	microRNA 4660	65651.04
237	chr17	57829891	57875903	MIR21	microRNA 21	65599.3084
238	chr10	80872033	80921265	ZMIZ1	zinc finger MIZ-type containing 1	65572.1008
239	chr12	125397715	125426199	MIR5188	microRNA 5188	65541.684
240	chr1	101700210	101731414	S1PR1	sphingosine-1-phosphate receptor 1	65475.3532
241	chr1	100103112	100159722	PALMD	palmdelphin	65316.618
242	chr12	25041524	25103335	BCAT1	branched chain amino acid transaminase 1	65117.8885
243	chr17	40669226	40684512	ATP6V0A1	ATPase H+ transporting V0 subunit a1	65051.1016
244	chr22	22290513	22308177	PPM1F	protein phosphatase, Mg2+/Mn2+ dependent 1F	64991.1552
245	chr20	23029205	23088549	CD93	CD93 molecule	64684.96
246	chr1	86032463	86052272	DDAH1	dimethylarginine dimethylaminohydrolase 1	64652.6142
247	chr5	75979048	76018544	NCRUPAR	non-protein coding RNA, upstream of F2R/PAR1	64548.3128
248	chr4	86681232	86766007	ARHGAP24	Rho GTPase activating protein 24	64352.7025
249	chr5	95897241	95951367	CAST	calpastatin	64215.0864
250	chr2	36762322	36800715	FEZ2	fasciculation and elongation protein zeta 2	64208.4532
251	chr11	65653947	65671575	CCDC85B	coiled-coil domain containing 85B	64076.0172
252	chr11	86166775	86197958	CCDC81	coiled-coil domain containing 81	64056.1186
253	chr15	39393712	39435307	C15orf54	chromosome 15 open reading frame 54	63860.8035
254	chr17	73840861	73875143	WBP2	WW domain binding protein 2	63829.6558
255	chr7	75920179	76023287	SSC4D	scavenger receptor cysteine rich family, 4 domains	63782.6088
256	chr11	86258599	86345784	ME3	malic enzyme 3, NADP(+)-dependent, mitochondrial	63505.554
257	chr16	24986883	25007567	ARHGAP17	Rho GTPase activating protein 17	63386.118
258	chr10	13715819	13759358	PRPF18	pre-mRNA processing factor 18	63231.6897
259	chr22	50549071	50559979	MOV10L1	Mov10 RISC complex RNA helicase like 1	63210.7692
260	chr2	159974227	160026948	TANC1	tetratricopeptide repeat, ankyrin repeat and coiled-coil containing 1	63175.5743
261	chr10	3815685	3830096	KLF6	Kruppel-like factor 6	63072.6237
262	chr15	95827713	95849368	LINC01197	long intergenic non-protein coding RNA 1197	62459.5165
263	chr12	66216228	66289670	RPSAP52	ribosomal protein SA pseudogene 52	62440.3884
264	chr20	1350206	1382567	FKBP1A-SDCBP2	FKBP1A-SDCBP2 readthrough (NMD candidate)	62294.925

Rank	chr	start	end	Gene	Gene description	H3K27ac signal
265	chr2	109251897	109296727	LIMS1	LIM zinc finger domain containing 1	62210.591
266	chr9	134581825	134615685	RAPGEF1	Rap guanine nucleotide exchange factor 1	62177.118
267	chr1	36598548	36654745	TRAPPC3	trafficking protein particle complex 3	62137.0229
268	chr11	118478263	118494747	PHLDB1	pleckstrin homology like domain family B member 1	62106.7668
269	chr15	63171185	63191564	MIR190A	microRNA 190a	62047.9413
270	chr15	66992522	67068022	SMAD6	SMAD family member 6	62015.7
271	chr1	94126759	94168135	BCAR3	breast cancer anti-estrogen resistance 3	61935.7344
272	chr2	56211127	56246415	MIR216B	microRNA 216b	61863.3928
273	chr8	23384979	23420264	SLC25A37	solute carrier family 25 member 37	61769.921
274	chr2	46523336	46595382	EPAS1	endothelial PAS domain protein 1	61714.6036
275	chr21	46781936	46789530	COL18A1	collagen type XVIII alpha 1	61443.054
276	chr11	12100713	12113876	MICAL2	microtubule associated monooxygenase, calponin and LIM domain containing 2	61168.461
277	chr22	25347837	25425402	KIAA1671	KIAA1671	61167.759
278	chr8	38852016	38901539	TM2D2	TM2 domain containing 2	61101.4774
279	chr3	185394602	185465225	IGF2BP2-AS1	IGF2BP2 antisense RNA 1	61095.9573
280	chr5	76077074	76135449	F2RL1	F2R like trypsin receptor 1	61042.7375
281	chr14	73108164	73147405	DPF3	double PHD fingers 3	60682.2824
282	chr7	115953501	116019012	CAV1	caveolin 1	60440.4486
283	chr14	69401382	69449544	ACTN1-AS1	ACTN1 antisense RNA 1	60289.1916
284	chr16	86694268	86716690	FOXL1	forkhead box L1	59947.4592
285	chr14	100196359	100226465	EML1	echinoderm microtubule associated protein like 1	59805.569
286	chr13	99127033	99163851	STK24	serine/threonine kinase 24	59774.023
287	chr8	13100908	13135549	DLC1	DLC1 Rho GTPase activating protein	59662.1943
288	chr3	50192378	50210933	SEMA3F	semaphorin 3F	59554.128
289	chr17	74365017	74383419	SPHK1	sphingosine kinase 1	59480.7846
290	chr11	114150710	114180316	NNMT	nicotinamide N-methyltransferase	59214.9606
291	chr7	43675602	43715196	STK17A	serine/threonine kinase 17a	59208.8676
292	chr16	53535789	53555066	AKTIP	AKT interacting protein	59084.005
293	chr22	44415533	44456138	PARVB	parvin beta	59080.275
294	chr15	89163947	89194984	ISG20	interferon stimulated exonuclease gene 20kDa	59038.5814
295	chr12	48193067	48216827	HDAC7	histone deacetylase 7	58967.568
296	chr2	46272094	46326612	EPAS1	endothelial PAS domain protein 1	58955.7652
297	chr11	128361680	128394507	ETS1	ETS proto-oncogene 1, transcription factor	58885.0726
298	chr4	57929006	57986466	IGFBP7-AS1	IGFBP7 antisense RNA 1	58603.454
299	chr18	3445975	3479417	TGIF1	TGFB induced factor homeobox 1	58583.6956
300	chr6	132380260	132409920	LINC01013	long intergenic non-protein coding RNA 1013	58542.908
301	chr9	114764125	114847374	MIR4668	microRNA 4668	58532.3719
302	chr6	151176897	151217964	MTHFD1L	methylenetetrahydrofolate dehydrogenase (NADP+ dependent) 1-like	58499.9415
303	chr19	45088988	45130994	IGSF23	immunoglobulin superfamily member 23	58253.9208
304	chr11	124745532	124791853	ROBO4	roundabout guidance receptor 4	58012.4204
305	chr3	42054220	42116156	TRAK1	trafficking protein, kinesin binding 1	57972.096
306	chr1	9456681	9475075	SPSB1	splA/ryanodine receptor domain and SOCS box containing 1	57841.7724
307	chr5	138836915	138876666	TMEM173	transmembrane protein 173	57479.946
308	chr9	6550326	6568985	UHRF2	ubiquitin like with PHD and ring finger domains 2	57342.8388
309	chr12	46822728	46889054	SLC38A2	solute carrier family 38 member 2	57080.1556

Rank	chr	start	end	Gene	Gene description	H3K27ac signal
310	chr6	12566975	12609976	PHACTR1	phosphatase and actin regulator 1	57015.0259
311	chr1	156861922	156878393	PEAR1	platelet endothelial aggregation receptor 1	57004.4839
312	chr12	11945824	11994797	ETV6	ETS variant 6	56598.0961
313	chr1	59218437	59252850	LINC01135	long intergenic non-protein coding RNA 1135	56347.8462
314	chr8	124472826	124503350	FBXO32	F-box protein 32	56332.042
315	chr19	11184244	11208642	LDLR	low density lipoprotein receptor	56203.2328
316	chr14	85981997	86004008	FLRT2	fibronectin leucine rich transmembrane protein 2	56172.072
317	chr6	166988327	167054753	RPS6KA2	ribosomal protein S6 kinase A2	56023.6884
318	chr1	32027644	32063653	TINAGL1	tubulointerstitial nephritis antigen like 1	55993.995
319	chr7	116138951	116195548	CAV1	caveolin 1	55940.4748
320	chr6	121824182	121846032	GJA1	gap junction protein alpha 1	55911.965
321	chr11	114028798	114052764	NNMT	nicotinamide N-methyltransferase	55718.5534
322	chr10	82252859	82272649	SH2D4B	SH2 domain containing 4B	55635.627
323	chr17	38169818	38196043	SNORD124	small nucleolar RNA, C/D box 124	55518.325
324	chr6	37204060	37228201	TMEM217	transmembrane protein 217	55495.3308
325	chr8	42036063	42074167	PLAT	plasminogen activator, tissue type	55387.9744
326	chr6	142692082	142738972	ADGRG6	adhesion G protein-coupled receptor G6	55372.401
327	chr8	8141652	8173485	FAM86B3P	family with sequence similarity 86, member A pseudogene	55360.7703
328	chrX	64886529	64944161	MSN	moesin	55217.2192
329	chr7	154996107	155005952	INSIG1	insulin induced gene 1	54993.1855
330	chr9	133707819	133739533	ABL1	ABL proto-oncogene 1, non-receptor tyrosine kinase	54985.7332
331	chr3	159478762	159516479	IQCJ-SCHIP1	IQCJ-SCHIP1 readthrough	54900.8652
332	chr17	73670335	73704724	SAP30BP	SAP30 binding protein	54884.844
333	chr3	14472158	14531301	GRIP2	glutamate receptor interacting protein 2	54878.7897
334	chr16	29271765	29284462	SNX29P2	sorting nexin 29 pseudogene 2	54842.1521
335	chr10	129704859	129748683	PTPRE	protein tyrosine phosphatase, receptor type E	54709.8816
336	chr3	16091523	16128598	GALNT15	polypeptide N-acetylgalactosaminyltransferase 15	54685.625
337	chr17	7743298	7749282	KDM6B	lysine demethylase 6B	54301.808
338	chr7	22670184	22717894	IL6	interleukin 6	54241.499
339	chr17	8041292	8062386	PER1	period circadian clock 1	54241.1116
340	chr14	61763760	61834459	PRKCH	protein kinase C eta	54105.9447
341	chr20	30131771	30162419	MCTS2P	malignant T-cell amplified sequence 2, pseudogene	54047.748
342	chr10	21602547	21662898	MIR1915	microRNA 1915	54032.2503
343	chr20	48752880	48789894	TMEM189	transmembrane protein 189	54014.5302
344	chr12	124982325	125009826	NCOR2	nuclear receptor corepressor 2	53943.2115
345	chr10	3918513	3940336	KLF6	Kruppel-like factor 6	53826.4295
346	chr11	86625033	86681653	LOC100506368	uncharacterized LOC100506368	53602.154
347	chr7	22456641	22486999	STEAP1B	STEAP family member 1B	53466.5096
348	chr1	17214518	17233340	CROCC	ciliary rootlet coiled-coil, rootletin	53379.192
349	chr9	139536090	139545666	EGFL7	EGF like domain multiple 7	53290.44
350	chr8	10632651	10683624	MIR1322	microRNA 1322	53001.7254
351	chr12	70989165	71066393	PTPRB	protein tyrosine phosphatase, receptor type B	52978.408
352	chr13	21606726	21655987	LATS2	large tumor suppressor kinase 2	52847.2008
353	chr2	216692935	216717695	LINC00607	long intergenic non-protein coding RNA 607	52681.852
354	chr9	35744305	35762735	GBA2	glucosidase, beta (bile acid) 2	52637.923

Rank	chr	start	end	Gene	Gene description	H3K27ac signal
355	chr3	10230316	10249580	IRAK2	interleukin 1 receptor associated kinase 2	52588.7936
356	chr6	7877002	7916727	TXNDC5	thioredoxin domain containing 5 (endoplasmic reticulum)	52587.955
357	chr2	47258607	47272398	TTC7A	tetratricopeptide repeat domain 7A	52569.9129
358	chr3	170027858	170084730	SKIL	SKI-like proto-oncogene	52538.3536
359	chr4	77921623	77945294	Sep-11	septin 11	52471.5057
360	chr14	23004002	23059643	DAD1	defender against cell death 1	52213.5144
361	chr5	71736476	71820549	ZNF366	zinc finger protein 366	52167.2965
362	chr17	57904840	57934505	MIR21	microRNA 21	52020.544
363	chr1	65326422	65366272	JAK1	Janus kinase 1	51904.625
364	chr10	124060043	124072216	BTBD16	BTB domain containing 16	51881.326
365	chr11	47414747	47449338	PSMC3	proteasome 26S subunit, ATPase 3	51806.9407
366	chr3	134079996	134094933	AMOTL2	angiomotin like 2	51768.6546
367	chr10	8081461	8109662	GATA3	GATA binding protein 3	51692.433
368	chr17	25658699	25713076	TBC1D3P5	TBC1 domain family member 3 pseudogene 5	51473.2682
369	chr10	14025589	14053301	FRMD4A	FERM domain containing 4A	51458.4128
370	chr5	72110560	72169936	TNPO1	transportin 1	51348.3648
371	chr7	2662714	2685753	TTYH3	tweety family member 3	51284.814
372	chr7	107918917	107954929	NRCAM	neuronal cell adhesion molecule	51245.076
373	chr5	110885681	110901351	STARD4	StAR related lipid transfer domain containing 4	51137.478
374	chr1	12192344	12247089	TNFRSF1B	tumor necrosis factor receptor superfamily member 1B	51077.085
375	chr2	201625536	201655055	BZW1	basic leucine zipper and W2 domains 1	51070.8219
376	chr1	153569257	153586333	S100A16	S100 calcium binding protein A16	50906.9712
377	chr17	2294412	2311449	MNT	MAX network transcriptional repressor	50545.3716
378	chr5	60558584	60631810	ZSWIM6	zinc finger SWIM-type containing 6	50357.5202
379	chr10	92671251	92694036	ANKRD1	ankyrin repeat domain 1	50227.254
380	chr2	110856191	110874869	MALL	mal, T-cell differentiation protein-like	50227.0098
381	chr2	37869801	37917334	CDC42EP3	CDC42 effector protein 3	50223.3678
382	chr11	118779259	118801210	MIR4492	microRNA 4492	50206.3272
383	chr3	50353720	50363956	HYAL2	hyaluronoglucosaminidase 2	50189.1552
384	chr2	159885124	159958832	TANC1	tetratricopeptide repeat, ankyrin repeat and coiled-coil containing 1	50150.9232
385	chr6	86158635	86176475	NT5E	5'-nucleotidase ecto	50137.536
386	chr12	46757347	46797912	SLC38A2	solute carrier family 38 member 2	50093.7185
387	chr14	105939416	105949914	CRIP2	cysteine rich protein 2	49965.231
388	chr7	137637767	137688169	LOC100130880	uncharacterized LOC100130880	49787.0956
389	chr16	73085818	73105845	ZFHX3	zinc finger homeobox 3	49628.9087
390	chr12	52400605	52440853	GRASP	GRP1 (general receptor for phosphoinositides 1)-associated scaffold protein	49589.5608
391	chr19	3422827	3461405	SMIM24	small integral membrane protein 24	49534.152
392	chr18	8776869	8815094	MTCL1	microtubule crosslinking factor 1	49512.8425
393	chr21	46284762	46302887	PTTG1IP	pituitary tumor-transforming 1 interacting protein	49470.375
394	chr1	201453974	201477419	CSRP1	cysteine and glycine rich protein 1	49426.749
395	chr3	184048335	184057433	FAM131A	family with sequence similarity 131 member A	49421.2458
396	chr5	141184158	141231551	PCDH1	protocadherin 1	49369.2881
397	chr3	141107232	141146918	ZBTB38	zinc finger and BTB domain containing 38	49337.6352
398	chr5	73923462	73940423	HEXB	hexosaminidase subunit beta	49286.9699
399	chr15	40615254	40637429	C15orf52	chromosome 15 open reading frame 52	49250.675

Rank	chr	start	end	Gene	Gene description	H3K27ac signal
400	chr20	19781960	19821850	RIN2	Ras and Rab interactor 2	49224.26
401	chr5	139623295	139648921	PFDN1	prefoldin subunit 1	49222.4208
402	chr14	77412839	77429282	IRF2BPL	interferon regulatory factor 2 binding protein-like	49125.1068
403	chr14	61564757	61572746	PRKCH	protein kinase C eta	49119.5676
404	chr8	68853654	68944316	PREX2	phosphatidylinositol-3,4,5-trisphosphate-dependent Rac exchange factor 2	49039.0758
405	chr5	148667027	148730130	AFAP1L1	actin filament associated protein 1 like 1	48955.3074
406	chr6	32934116	32942892	BRD2	bromodomain containing 2	48927.0776
407	chr5	141570711	141632115	SPRY4	sprouty RTK signaling antagonist 4	48896.0052
408	chr20	23120881	23161834	LINC00656	long intergenic non-protein coding RNA 656	48885.5961
409	chr16	88267259	88280235	ZNF469	zinc finger protein 469	48836.4736
410	chr15	64124559	64153381	HERC1	HECT and RLD domain containing E3 ubiquitin protein ligase family member 1	48746.6486
411	chr3	99352188	99390233	COL8A1	collagen type VIII alpha 1	48716.6225
412	chr8	129179298	129198763	MIR1208	microRNA 1208	48683.9115
413	chr5	66298589	66313565	MAST4	microtubule associated serine/threonine kinase family member 4	48589.632
414	chr2	161225492	161291735	MIR4785	microRNA 4785	48509.7489
415	chr14	62026571	62033942	FLJ22447	uncharacterized LOC400221	48508.551
416	chr2	238511196	238527885	LRRFIP1	leucine rich repeat (in FLII) interacting protein 1	48464.856
417	chr8	49319045	49346166	EFCAB1	EF-hand calcium binding domain 1	48115.3661
418	chr1	150575472	150596397	ENSA	endosulfine alpha	48089.835
419	chr9	14170062	14252321	NFIB	nuclear factor I/B	47841.8344
420	chr6	12288091	12299871	EDN1	endothelin 1	47774.968
421	chr2	112247116	112254781	MIR4435-2HG	MIR4435-2 host gene	47763.681
422	chr13	28952320	28988985	FLT1	fms related tyrosine kinase 1	47756.1625
423	chr1	154938549	154949909	MIR4258	microRNA 4258	47738.128
424	chr1	161154787	161177615	NDUFS2	NADH:ubiquinone oxidoreductase core subunit S2	47575.8348
425	chr3	57934962	57971838	FLNB	filamin B	47474.1624
426	chr1	59346222	59377199	LINC01135	long intergenic non-protein coding RNA 1135	47357.6376
427	chr1	43935912	43944156	HYI	hydroxypyruvate isomerase (putative)	47266.974
428	chr22	30804023	30832897	SEC14L2	SEC14 like lipid binding 2	47217.6522
429	chr1	231522153	231559441	EGLN1	egl-9 family hypoxia-inducible factor 1	47169.32
430	chr5	39500217	39548277	DAB2	Dab, mitogen-responsive phosphoprotein, homolog 2 (Drosophila)	47127.636
431	chr9	116713898	116721461	ZNF618	zinc finger protein 618	47029.0029
432	chr11	111784395	111818713	DIXDC1	DIX domain containing 1	46933.2968
433	chr9	37915738	37970760	SLC25A51	solute carrier family 25 member 51	46900.7528
434	chr4	56025571	56054076	KDR	kinase insert domain receptor	46890.725
435	chrX	99896826	99941255	SRPX2	sushi repeat containing protein, X-linked 2	46783.737
436	chr10	3844622	3854912	KLF6	Kruppel-like factor 6	46734.093
437	chr9	125576840	125591571	PDCL	phosducin like	46709.0548
438	chr3	71083083	71117008	FOXP1	forkhead box P1	46456.895
439	chr6	3735967	3754926	PXDC1	PX domain containing 1	46373.714
440	chr14	105657930	105666947	NUDT14	nudix hydrolase 14	46351.8885
441	chr6	132450957	132457836	LINC01013	long intergenic non-protein coding RNA 1013	46304.6127
442	chr19	48610048	48630948	PLA2G4C	phospholipase A2 group IVC	46276.78
443	chr8	55221482	55252806	SOX17	SRY-box 17	46271.8128
444	chr22	46460348	46478683	MIRLET7BHG	MIRLET7B host gene	46242.7035

Rank	chr	start	end	Gene	Gene description	H3K27ac signal
445	chr9	95414886	95434813	IPPK	inositol-pentakisphosphate 2-kinase	46204.7349
446	chr16	55504280	55540769	MMP2	matrix metallopeptidase 2	46180.4784
447	chr18	22806590	22835080	ZNF521	zinc finger protein 521	46116.763
448	chr11	86428643	86456548	ME3	malic enzyme 3, NADP(+)-dependent, mitochondrial	46096.2695
449	chr7	104648466	104658800	KMT2E-AS1	KMT2E antisense RNA 1 (head to head)	46074.139
450	chr19	16175998	16208100	TPM4	tropomyosin 4	46040.6884
451	chr9	117651646	117665848	TNFSF8	tumor necrosis factor superfamily member 8	46024.4214
452	chr9	132243652	132269839	LINC00963	long intergenic non-protein coding RNA 963	45979.1346
453	chr20	23329337	23356027	NXT1	nuclear transport factor 2-like export factor 1	45880.11
454	chr2	71717691	71733523	DYSF	dysferlin	45863.7208
455	chr3	177060668	177079906	LINC00578	long intergenic non-protein coding RNA 578	45824.916
456	chr10	31252447	31303765	ZNF438	zinc finger protein 438	45708.9426
457	chr19	16425720	16463285	KLF2	Kruppel-like factor 2	45671.527
458	chr2	151319138	151349487	RND3	Rho family GTPase 3	45638.8262
459	chr5	141057635	141064107	ARAP3	ArfGAP with RhoGAP domain, ankyrin repeat and PH domain 3	45628.8944
460	chr12	66022868	66054582	HMGA2	high mobility group AT-hook 2	45573.018
461	chr6	17829101	17871005	KIF13A	kinesin family member 13A	45537.0768
462	chr6	34190771	34217791	C6orf1	chromosome 6 open reading frame 1	45531.402
463	chr2	237778562	237799406	COPS8	COP9 signalosome subunit 8	45437.8356
464	chr22	32020743	32059185	PISD	phosphatidylserine decarboxylase	45388.4694
465	chr6	136355016	136400144	PDE7B	phosphodiesterase 7B	45213.7432
466	chr6	134489094	134501143	SGK1	serum/glucocorticoid regulated kinase 1	45163.2667
467	chr12	45608157	45630339	ANO6	anoctamin 6	45160.3338
468	chr14	69500314	69527724	ACTN1-AS1	ACTN1 antisense RNA 1	45127.824
469	chr1	16073486	16089848	FBLIM1	filamin binding LIM protein 1	44799.156
470	chr9	131888494	131909455	PPP2R4	protein phosphatase 2A regulatory subunit 4	44743.3506
471	chr16	86596680	86625076	FOXL1	forkhead box L1	44726.5396
472	chr3	193971756	193990785	LINC00887	long intergenic non-protein coding RNA 887	44666.7717
473	chr22	50323285	50364932	PIM3	Pim-3 proto-oncogene, serine/threonine kinase	44553.9606
474	chr14	90848345	90886727	CALM1	calmodulin 1 (phosphorylase kinase, delta)	44465.547
475	chr18	19746590	19774999	GATA6-AS1	GATA6 antisense RNA 1 (head to head)	44462.9259
476	chr18	8704044	8752689	MTCL1	microtubule crosslinking factor 1	44354.511
477	chr10	5983368	6020478	IL15RA	interleukin 15 receptor subunit alpha	44186.877
478	chr1	214574393	214630384	PTPN14	protein tyrosine phosphatase, non-receptor type 14	44165.7008
479	chr11	103456279	103505834	MIR4693	microRNA 4693	44079.1725
480	chr16	81638015	81669172	CMIP	c-Maf inducing protein	43946.9485
481	chr9	5487467	5512950	PDCD1LG2	programmed cell death 1 ligand 2	43917.4022
482	chr4	145546782	145589364	HHIP	hedgehog interacting protein	43855.2018
483	chr9	124039564	124052655	GSN	gelsolin	43826.0498
484	chr16	87983588	88001097	BANP	BTG3 associated nuclear protein	43798.7635
485	chr1	68295105	68323002	GNG12-AS1	GNG12 antisense RNA 1	43736.9166
486	chr1	227615035	227621411	CDC42BPA	CDC42 binding protein kinase alpha	43700.4664
487	chr10	59995571	60044501	IPMK	inositol polyphosphate multikinase	43684.704
488	chr21	38729253	38749227	DYRK1A	dual specificity tyrosine phosphorylation regulated kinase 1A	43665.1614
489	chr3	141079485	141090213	ZBTB38	zinc finger and BTB domain containing 38	43617.9024

Rank	chr	start	end	Gene	Gene description	H3K27ac signal
490	chr16	66541738	66559144	TK2	thymidine kinase 2, mitochondrial	43603.7706
491	chr12	125196895	125232693	SCARB1	scavenger receptor class B member 1	43598.3842
492	chr19	11241065	11291803	SPC24	SPC24, NDC80 kinetochore complex component	43589.0158
493	chr10	99293582	99304969	ANKRD2	ankyrin repeat domain 2	43584.8812
494	chr17	36603015	36630028	ARHGAP23	Rho GTPase activating protein 23	43566.5664
495	chr10	94571418	94610800	EXOC6	exocyst complex component 6	43532.8628
496	chr3	45672008	45712383	LIMD1-AS1	LIMD1 antisense RNA 1	43532.325
497	chr22	36835024	36868015	TXN2	thioredoxin 2	43525.0263
498	chr18	20804035	20819467	CABLES1	Cdk5 and Abl enzyme substrate 1	43484.2896
499	chr5	72898836	72952298	ARHGEF28	Rho guanine nucleotide exchange factor 28	43469.9522
500	chr3	145873369	145915695	PLOD2	procollagen-lysine, 2-oxoglutarate 5-dioxygenase 2	43443.4064
501	chr1	94642448	94732671	ARHGAP29	Rho GTPase activating protein 29	43415.3076
502	chr15	41196458	41235507	DLL4	delta-like 4 (Drosophila)	43352.1998
503	chr2	87753142	87758634	LINC00152	long intergenic non-protein coding RNA 152	43094.6256
504	chr12	6430963	6457470	TNFRSF1A	tumor necrosis factor receptor superfamily member 1A	43071.2243
505	chr18	46448305	46487531	SMAD7	SMAD family member 7	43011.309
506	chr16	87489826	87501185	ZCCHC14	zinc finger CCHC-type containing 14	42997.2227
507	chr3	159540667	159603318	SCHIP1	schwannomin interacting protein 1	42922.2001
508	chr8	41980787	42013983	AP3M2	adaptor related protein complex 3 mu 2 subunit	42919.1084
509	chr9	35508637	35522483	RUSC2	RUN and SH3 domain containing 2	42838.1394
510	chr3	111564152	111594186	PHLDB2	pleckstrin homology like domain family B member 2	42780.4296
511	chr3	111619534	111686992	PHLDB2	pleckstrin homology like domain family B member 2	42754.8804
512	chr4	55933762	55947530	KDR	kinase insert domain receptor	42745.5096
513	chr5	169396127	169418027	FAM196B	family with sequence similarity 196 member B	42731.28
514	chr14	74249742	74270201	ELMSAN1	ELM2 and Myb/SANT-like domain containing 1	42687.7035
515	chr12	11849871	11901402	ETV6	ETS variant 6	42683.1273
516	chr3	15332026	15374833	SH3BP5	SH3-domain binding protein 5	42588.6843
517	chr7	47979412	47991415	PKD1L1	polycystin 1 like 1, transient receptor potential channel interacting	42494.2209
518	chr10	127729274	127752801	FANK1	fibronectin type III and ankyrin repeat domains 1	42395.654
519	chr20	37427180	37460293	PPP1R16B	protein phosphatase 1 regulatory subunit 16B	42361.4609
520	chr11	2398438	2424305	TSSC4	tumor suppressing subtransferable candidate 4	42186.4903
521	chr8	145001890	145027401	PLEC	plectin	42164.5808
522	chr8	10566986	10589116	SOX7	SRY-box 7	42126.668
523	chr1	23491911	23526140	HTR1D	5-hydroxytryptamine receptor 1D	42074.2868
524	chr19	45347509	45374496	NECTIN2	nectin cell adhesion molecule 2	42070.0343
525	chr12	118099654	118126726	KSR2	kinase suppressor of ras 2	42013.0368
526	chr8	134531969	134597137	ST3GAL1	ST3 beta-galactoside alpha-2,3-sialyltransferase 1	42007.2928
527	chr1	25030753	25099953	CLIC4	chloride intracellular channel 4	41997.48
528	chr7	131303077	131328571	PODXL	podocalyxin like	41940.1794
529	chr6	126101265	126162051	NCOA7	nuclear receptor coactivator 7	41918.0256
530	chr22	35694489	35727481	TOM1	target of myb1 membrane trafficking protein	41893.2416
531	chr2	56109410	56152051	EFEMP1	EGF containing fibulin-like extracellular matrix protein 1	41822.2928
532	chr2	1710953	1760482	PXDN	peroxidasin	41802.476
533	chr8	128805922	128829620	MIR1204	microRNA 1204	41755.876
534	chr7	50879731	50908227	GRB10	growth factor receptor bound protein 10	41726.6928

Rank	chr	start	end	Gene	Gene description	H3K27ac signal
535	chr4	78837398	78855428	MRPL1	mitochondrial ribosomal protein L1	41606.028
536	chr3	128198376	128212235	GATA2	GATA binding protein 2	41522.9499
537	chr8	26114376	26126416	PPP2R2A	protein phosphatase 2 regulatory subunit B, alpha	41504.288
538	chr5	131592525	131603943	PDLIM4	PDZ and LIM domain 4	41454.1908
539	chr17	62078249	62103649	ICAM2	intercellular adhesion molecule 2	41351.2
540	chr22	29998610	30032565	NF2	neurofibromin 2 (merlin)	41336.817
541	chr7	640445	660691	PRKAR1B	protein kinase cAMP-dependent type I regulatory subunit beta	41332.209
542	chr8	55046974	55093698	MRPL15	mitochondrial ribosomal protein L15	41322.7056
543	chr3	5054602	5069518	BHLHE40-AS1	BHLHE40 antisense RNA 1	41318.8116
544	chr2	28805346	28846017	PLB1	phospholipase B1	41289.1992
545	chr22	25506006	25543365	LOC100128531	uncharacterized LOC100128531	41251.8078
546	chr19	47676285	47715750	MIR3190	microRNA 3190	41043.6
547	chr19	6085543	6111915	RFX2	regulatory factor X2	41026.9204
548	chr2	178102987	178140882	MIR3128	microRNA 3128	41013.7585
549	chr14	61927232	61947049	PRKCH	protein kinase C eta	40896.3429
550	chrX	45602036	45669243	MIR221	microRNA 221	40895.4595
551	chr11	128041074	128082002	ETS1	ETS proto-oncogene 1, transcription factor	40764.288
552	chr10	75645774	75672136	PLAU	plasminogen activator, urokinase	40563.2094
553	chr20	10602727	10656474	JAG1	jagged 1	40562.8609
554	chr12	96864863	96910767	CDK17	cyclin-dependent kinase 17	40551.5936
555	chr3	149083873	149120825	TM4SF1	transmembrane 4 L six family member 1	40547.4296
556	chr7	139734253	139763720	PARP12	poly(ADP-ribose) polymerase family member 12	40520.0717
557	chr5	42988228	42997098	LOC648987	uncharacterized LOC648987	40495.985
558	chr5	133884985	133918251	JADE2	jade family PHD finger 2	40488.0486
559	chr1	235046954	235070019	LINC01132	long intergenic non-protein coding RNA 1132	40430.6385
560	chr12	124905710	124952336	NCOR2	nuclear receptor corepressor 2	40270.8762
561	chr15	39109282	39139297	C15orf53	chromosome 15 open reading frame 53	40238.109
562	chr19	12887958	12905706	JUNB	jun B proto-oncogene	40225.842
563	chr2	225263189	225283614	FAM124B	family with sequence similarity 124 member B	40200.485
564	chr22	37877566	37922556	CARD10	caspase recruitment domain family member 10	40099.587
565	chr1	94073030	94103055	BCAR3	breast cancer anti-estrogen resistance 3	40059.355
566	chr22	37571920	37596635	C1QTNF6	C1q and tumor necrosis factor related protein 6	40058.072
567	chr10	3493243	3515373	PITRM1	pitrilysin metallopeptidase 1	40039.809
568	chr9	101078720	101139000	TBC1D2	TBC1 domain family member 2	40007.836
569	chr17	68153074	68170765	KCNJ2	potassium voltage-gated channel subfamily J member 2	39992.2746
570	chr4	129423270	129451406	PGRMC2	progesterone receptor membrane component 2	39975.6288
571	chr15	39649179	39659831	C15orf54	chromosome 15 open reading frame 54	39968.4344
572	chr17	79517761	79551904	NPLOC4	NPL4 homolog, ubiquitin recognition factor	39906.3384
573	chr1	209995527	210018047	DIEXF	digestive organ expansion factor homolog (zebrafish)	39889.676
574	chr5	141658957	141678494	SPRY4	sprouty RTK signaling antagonist 4	39876.9707
575	chr13	99596419	99636897	DOCK9	dedicator of cytokinesis 9	39874.8778
576	chr13	40436169	40475462	MIR4305	microRNA 4305	39858.8192
577	chr2	109195333	109214139	LIMS1	LIM zinc finger domain containing 1	39832.9886
578	chr1	21648348	21673112	ECE1	endothelin converting enzyme 1	39788.3188
579	chr4	10089401	10126639	WDR1	WD repeat domain 1	39710.6032

Rank	chr	start	end	Gene	Gene description	H3K27ac signal
580	chr3	81772370	81812120	GBE1	glucan (1,4-alpha-), branching enzyme 1	39626.775
581	chr21	43345567	43382942	C2CD2	C2 calcium-dependent domain containing 2	39576.3875
582	chr15	95381573	95390967	LOC440311	glioma tumor suppressor candidate region gene 2 pseudogene	39529.952
583	chr11	107689032	107731012	SLC35F2	solute carrier family 35 member F2	39486.388
584	chr8	22905960	22950966	LOC254896	uncharacterized LOC254896	39465.7614
585	chr12	52557396	52589292	KRT80	keratin 80	39433.0248
586	chr17	38253769	38292089	MSL1	male-specific lethal 1 homolog (Drosophila)	39431.28
587	chr19	6218116	6245386	MLLT1	myeloid/lymphoid or mixed-lineage leukemia; translocated to, 1	39416.058
588	chr6	157448972	157474230	ARID1B	AT-rich interaction domain 1B	39387.3252
589	chr9	128393381	128439454	MAPKAP1	mitogen-activated protein kinase associated protein 1	39364.7712
590	chr8	119015570	119039511	EXT1	exostosin glycosyltransferase 1	39318.3043
591	chr1	68637892	68647865	MIR1262	microRNA 1262	39302.5957
592	chr3	52078870	52090426	DUSP7	dual specificity phosphatase 7	39285.7776
593	chr7	30498531	30519537	NOD1	nucleotide binding oligomerization domain containing 1	39268.6164
594	chr1	120173338	120192169	ZNF697	zinc finger protein 697	39264.5181
595	chr8	26464187	26473273	DPYSL2	dihydropyrimidinase like 2	39230.6222
596	chr16	27323910	27348285	IL4R	interleukin 4 receptor	39109.6875
597	chr5	150011549	150025370	SYNPO	synaptopodin	39092.6985
598	chr1	19755550	19813809	CAPZB	capping actin protein of muscle Z-line beta subunit	39062.6595
599	chr1	47673162	47708150	TAL1	T-cell acute lymphocytic leukemia 1	39057.1044
600	chr6	86110120	86116848	NT5E	5'-nucleotidase ecto	39021.7272
601	chr15	42782047	42804497	SNAP23	synaptosome associated protein 23kDa	38995.65
602	chr17	61476061	61529605	CYB561	cytochrome b561	38953.26
603	chr1	43388603	43411293	SLC2A1-AS1	SLC2A1 antisense RNA 1	38945.116
604	chr1	61408245	61430322	NFIA	nuclear factor I/A	38842.2738
605	chr19	39918325	39927420	RPS16	ribosomal protein S16	38715.596
606	chr1	16248952	16294373	ZBTB17	zinc finger and BTB domain containing 17	38621.4763
607	chr12	12866720	12890992	CDKN1B	cyclin-dependent kinase inhibitor 1B	38587.6256
608	chr1	12654570	12680436	DHRS3	dehydrogenase/reductase (SDR family) member 3	38586.8988
609	chr1	65886194	65898412	LEPR	leptin receptor	38562.4516
610	chr10	82166660	82178604	FAM213A	family with sequence similarity 213 member A	38382.044
611	chr20	43200748	43245818	ADA	adenosine deaminase	38363.584
612	chr11	73017243	73047294	ARHGEF17	Rho guanine nucleotide exchange factor 17	38306.0097
613	chr17	47891374	47929568	KAT7	lysine acetyltransferase 7	38262.7492
614	chr19	1245872	1267385	MIDN	midnolin	38133.9438
615	chr6	34981838	35017423	ANKS1A	ankyrin repeat and sterile alpha motif domain containing 1A	38132.886
616	chr5	52278788	52342057	ITGA2	integrin subunit alpha 2	38132.2263
617	chr1	33445542	33473551	RNF19B	ring finger protein 19B	38081.0364
618	chr9	35904285	35912492	LINC00961	long intergenic non-protein coding RNA 961	38058.3211
619	chr2	64487092	64504998	LINC00309	long intergenic non-protein coding RNA 309	38052.0406
620	chr6	148625044	148669159	SASH1	SAM and SH3 domain containing 1	38044.776
621	chr11	65138006	65159729	SLC25A45	solute carrier family 25 member 45	38034.8007
622	chr16	67549482	67607064	CTCF	CCCTC-binding factor	37975.329
623	chr6	134739441	134759287	LINC01010	long intergenic non-protein coding RNA 1010	37969.3672
624	chr11	58340187	58348883	ZFP91-CNTF	ZFP91-CNTF readthrough (NMD candidate)	37965.8664

Rank	chr	start	end	Gene	Gene description	H3K27ac signal
625	chr8	40010065	40033670	C8orf4	chromosome 8 open reading frame 4	37947.398
626	chr14	35852457	35889861	NFKBIA	NFKB inhibitor alpha	37942.6176
627	chr19	54958298	54983353	LENG8	leukocyte receptor cluster (LRC) member 8	37923.248
628	chr15	101706473	101742517	CHSY1	chondroitin sulfate synthase 1	37864.222
629	chr13	40763789	40782083	LINC00332	long intergenic non-protein coding RNA 332	37819.1862
630	chr14	69237210	69263600	ZFP36L1	ZFP36 ring finger protein-like 1	37793.119
631	chr15	93159265	93199772	FAM174B	family with sequence similarity 174 member B	37793.031
632	chr13	110867562	110920082	COL4A1	collagen type IV alpha 1	37767.132
633	chr6	3909183	3918045	FAM50B	family with sequence similarity 50 member B	37707.81
634	chr1	175474597	175491714	TNR	tenascin R	37705.3276
635	chr7	22520082	22541230	STEAP1B	STEAP family member 1B	37692.0804
636	chr10	35656896	35677623	CCNY	cyclin Y	37685.8314
637	chr4	38664315	38691862	KLF3	Kruppel-like factor 3 (basic)	37659.5037
638	chr5	14427667	14478864	FAM105A	family with sequence similarity 105 member A	37609.3162
639	chr19	6268650	6280173	MLLT1	myeloid/lymphoid or mixed-lineage leukemia; translocated to, 1	37590.3306
640	chr17	57499677	57519934	MIR4729	microRNA 4729	37586.8635
641	chr3	46966789	47004011	CCDC12	coiled-coil domain containing 12	37553.2758
642	chr1	94555951	94562194	ABCA4	ATP binding cassette subfamily A member 4	37532.916
643	chr15	77773007	77801872	HMG20A	high mobility group 20A	37521.6135
644	chr16	87869342	87903756	SLC7A5	solute carrier family 7 member 5	37449.3148
645	chr1	43669919	43675016	EBNA1BP2	EBNA1 binding protein 2	37366.107
646	chr20	51105716	51115050	ZFP64	ZFP64 zinc finger protein	37359.335
647	chr15	86121513	86165183	AKAP13	A-kinase anchoring protein 13	37342.217
648	chr17	76856936	76881348	TIMP2	TIMP metallopeptidase inhibitor 2	37330.8304
649	chr12	26829765	26859907	ITPR2	inositol 1,4,5-trisphosphate receptor type 2	37315.796
650	chr5	71882103	71902689	ZNF366	zinc finger protein 366	37291.539
651	chr6	44007801	44041264	C6orf223	chromosome 6 open reading frame 223	37230.9338
652	chr1	153536703	153542142	S100A2	S100 calcium binding protein A2	37154.8968
653	chr20	62312379	62332473	RTEL1-TNFRSF6B	RTEL1-TNFRSF6B readthrough (NMD candidate)	37097.5428
654	chr7	129587614	129611611	UBE2H	ubiquitin conjugating enzyme E2H	37068.1659
655	chr12	104669615	104701025	EID3	EP300 interacting inhibitor of differentiation 3	37057.518
656	chr1	183837877	183859119	COLGALT2	collagen beta(1-O)galactosyltransferase 2	36980.1978
657	chr10	33268357	33276050	ITGB1	integrin subunit beta 1	36937.1702
658	chr20	20710803	20747438	RALGAPA2	Ral GTPase activating protein catalytic alpha subunit 2	36873.1275
659	chr3	156873798	156895065	CCNL1	cyclin L1	36840.8241
660	chr3	41010770	41019459	CTNNB1	catenin beta 1	36815.293
661	chr2	204546039	204557836	CD28	CD28 molecule	36788.9445
662	chr19	3140172	3163540	GNA15	G protein subunit alpha 15	36767.2112
663	chr7	4763505	4789954	AP5Z1	adaptor related protein complex 5 zeta 1 subunit	36708.5671
664	chr2	43384880	43407124	ZFP36L2	ZFP36 ring finger protein-like 2	36651.4388
665	chr10	15237001	15262865	NMT2	N-myristoyltransferase 2	36623.424
666	chr20	10330777	10358238	MKKS	McKusick-Kaufman syndrome	36597.2747
667	chr1	157962070	157991455	KIRREL	kin of IRRE like (Drosophila)	36563.7555
668	chr5	106800702	106814852	EFNA5	ephrin-A5	36535.3
669	chr2	231621362	231661962	CAB39	calcium binding protein 39	36491.28

Rank	chr	start	end	Gene	Gene description	H3K27ac signal
670	chr9	91388493	91397760	MIR4289	microRNA 4289	36460.0848
671	chr15	99431644	99468676	IGF1R	insulin like growth factor 1 receptor	36380.2368
672	chr21	45540579	45561900	C21orf33	chromosome 21 open reading frame 33	36375.7581
673	chr6	18361792	18409413	RNF144B	ring finger protein 144B	36372.9198
674	chr5	148344105	148365045	SH3TC2	SH3 domain and tetratricopeptide repeats 2	36347.652
675	chr6	132266906	132277675	CTGF	connective tissue growth factor	36342.1443
676	chr2	216261075	216304089	FN1	fibronectin 1	36299.5146
677	chr4	8184172	8208567	SH3TC1	SH3 domain and tetratricopeptide repeats 1	36292.4415
678	chr3	32439956	32465877	CMTM7	CKLF like MARVEL transmembrane domain containing 7	36266.0711
679	chr5	32272579	32279044	MTMR12	myotubularin related protein 12	36264.771
680	chr3	170443413	170478626	RPL22L1	ribosomal protein L22 like 1	36255.3048
681	chr6	163755538	163790262	DKFZp451B082	uncharacterized LOC401282	36192.8252
682	chr6	114175314	114183165	MARCKS	myristoylated alanine rich protein kinase C substrate	36091.047
683	chr5	95192142	95207380	LINC01554	long intergenic non-protein coding RNA 1554	36077.4888
684	chr1	156714463	156739339	HDGF	hepatoma-derived growth factor	36040.3488
685	chr15	89632849	89687934	ABHD2	abhydrolase domain containing 2	36031.0985
686	chr21	44895932	44919168	LINC00313	long intergenic non-protein coding RNA 313	36027.418
687	chr17	75133362	75151827	SEC14L1	SEC14 like lipid binding 1	35995.671
688	chr6	74403674	74435543	CD109	CD109 molecule	35983.2879
689	chr7	108042675	108097635	NRCAM	neuronal cell adhesion molecule	35943.84
690	chr5	59039102	59066226	PDE4D	phosphodiesterase 4D	35939.3
691	chr2	169847646	169870771	ABCB11	ATP binding cassette subfamily B member 11	35859.9375
692	chr10	114804459	114854425	TCF7L2	transcription factor 7 like 2	35850.605
693	chr7	111798628	111847952	ZNF277	zinc finger protein 277	35848.6832
694	chr3	105069904	105089954	ALCAM	activated leukocyte cell adhesion molecule	35839.375
695	chr2	191833915	191850641	STAT1	signal transducer and activator of transcription 1	35810.366
696	chr22	43316939	43337750	PACSIN2	protein kinase C and casein kinase substrate in neurons 2	35713.7571
697	chr20	19864864	19872345	RIN2	Ras and Rab interactor 2	35700.0801
698	chr10	44341102	44364929	LINC00840	long intergenic non-protein coding RNA 840	35597.538
699	chr15	90904797	90964446	IQGAP1	IQ motif containing GTPase activating protein 1	35586.5934
700	chr14	63640432	63683445	RHOJ	ras homolog family member J	35545.9432
701	chr8	8186677	8222693	SGK223	homolog of rat pragma of Rnd2	35540.5888
702	chr3	11674239	11686447	VGLL4	vestigial like family member 4	35537.488
703	chr19	49375064	49380301	PPP1R15A	protein phosphatase 1 regulatory subunit 15A	35506.3363
704	chr11	101781761	101787865	ANGPTL5	angiopoietin like 5	35504.5264
705	chr3	129199688	129220316	IFT122	intraflagellar transport 122	35473.9716
706	chr2	206544825	206579157	NRP2	neuropilin 2	35410.0248
707	chr12	76413064	76434074	PHLDA1	pleckstrin homology like domain family A member 1	35338.82
708	chr10	82202296	82238765	TSPAN14	tetraspanin 14	35305.6389
709	chr8	36998716	37006779	KCNU1	potassium calcium-activated channel subfamily U member 1	35302.2329
710	chr14	61643482	61656707	PRKCH	protein kinase C eta	35193.0475
711	chr15	57842076	57855285	MYZAP	myocardial zonula adherens protein	35171.6043
712	chr17	62392089	62410912	PECAM1	platelet/endothelial cell adhesion molecule 1	35159.4817
713	chr7	107219602	107263790	BCAP29	B-cell receptor-associated protein 29	35151.554
714	chr3	191045181	191097926	CCDC50	coiled-coil domain containing 50	35117.621

Rank	chr	start	end	Gene	Gene description	H3K27ac signal
715	chr14	51860021	51866506	LINC00640	long intergenic non-protein coding RNA 640	35101.3595
716	chr8	23000610	23039961	TNFRSF10D	tumor necrosis factor receptor superfamily member 10d	34986.9741
717	chr7	41225958	41231243	SUGCT	succinyl-CoA:glutarate-CoA transferase	34972.4305
718	chr17	55944220	55972067	CUEDC1	CUE domain containing 1	34967.4779
719	chr5	176915939	176925995	PDLIM7	PDZ and LIM domain 7	34958.6784
720	chr11	57528249	57570724	CTNND1	catenin delta 1	34922.945
721	chr1	22089321	22111221	USP48	ubiquitin specific peptidase 48	34821
722	chr8	22452294	22459781	C8orf58	chromosome 8 open reading frame 58	34817.5448
723	chr22	37940992	37962201	CDC42EP1	CDC42 effector protein 1	34806.0899
724	chr6	32162032	32167049	NOTCH4	notch 4	34791.8916
725	chr2	43132807	43157673	HAAO	3-hydroxyanthranilate 3,4-dioxygenase	34775.101
726	chr11	76287442	76301207	EMSY	EMSY, BRCA2 interacting transcriptional repressor	34766.2605
727	chr5	131628047	131633648	SLC22A4	solute carrier family 22 member 4	34746.9237
728	chr11	19726620	19746419	LOC100126784	uncharacterized LOC100126784	34693.7877
729	chr14	91573803	91586814	C14orf159	chromosome 14 open reading frame 159	34670.4117
730	chr17	26660262	26676349	TNFAIP1	TNF alpha induced protein 1	34656.2241
731	chr18	20836566	20863493	TMEM241	transmembrane protein 241	34655.049
732	chr5	55461898	55510615	ANKRD55	ankyrin repeat domain 55	34652.4021
733	chr5	43033287	43043719	LOC153684	uncharacterized LOC153684	34644.672
734	chr19	18473960	18501035	MIR3189	microRNA 3189	34588.3125
735	chr2	192695705	192744828	SDPR	serum deprivation response	34533.469
736	chr2	202558524	202574002	MPP4	membrane protein, palmitoylated 4	34475.6972
737	chr16	2201404	2222826	SNORD60	small nucleolar RNA, C/D box 60	34450.8604
738	chr10	4805114	4824922	AKR1E2	aldo-keto reductase family 1, member E2	34418.3808
739	chr1	39844584	39878154	KIAA0754	KIAA0754	34389.108
740	chr8	59729023	59793441	NSMAF	neutral sphingomyelinase activation associated factor	34386.3284
741	chr4	7956808	7974763	ABLIM2	actin binding LIM protein family member 2	34373.052
742	chr15	71566007	71589810	THSD4	thrombospondin type 1 domain containing 4	34354.8699
743	chr3	30551398	30592399	TGFBR2	transforming growth factor beta receptor II	34305.5367
744	chr11	10312641	10352480	ADM	adrenomedullin	34269.5078
745	chr17	1615175	1622438	WDR81	WD repeat domain 81	34204.3722
746	chr19	48745076	48764191	CARD8-AS1	CARD8 antisense RNA 1	34183.3545
747	chr1	113159593	113181131	ST7L	suppression of tumorigenicity 7 like	34182.9598
748	chr3	101848372	101901316	ZPLD1	zona pellucida-like domain containing 1	34170.0576
749	chr18	77243605	77269153	NFATC1	nuclear factor of activated T-cells, cytoplasmic, calcineurin-dependent 1	34050.3744
750	chr1	16159402	16179284	SPEN	spen family transcriptional repressor	34049.9132
751	chr3	120134545	120170855	FSTL1	follistatin like 1	33957.112
752	chr7	48123676	48139344	UPP1	uridine phosphorylase 1	33954.1228
753	chr5	148824693	148845462	MIR143	microRNA 143	33953.1612
754	chr6	26203027	26208300	HIST1H4E	histone cluster 1, H4e	33911.1903
755	chr15	91411734	91430330	FES	FES proto-oncogene, tyrosine kinase	33852.1584
756	chr11	95939449	95997914	MIR1260B	microRNA 1260b	33845.3885
757	chr13	99532268	99581959	DOCK9	dedicator of cytokinesis 9	33814.7255
758	chr3	9433391	9444963	THUMPD3-AS1	THUMPD3 antisense RNA 1	33764.7816
759	chr12	122230554	122245307	SETD1B	SET domain containing 1B	33757.8146

Rank	chr	start	end	Gene	Gene description	H3K27ac signal
760	chr12	76369500	76393387	PHLDA1	pleckstrin homology like domain family A member 1	33735.6101
761	chr3	134190041	134207139	ANAPC13	anaphase promoting complex subunit 13	33664.2522
762	chr17	80801535	80845650	ZNF750	zinc finger protein 750	33606.807
763	chr12	123353922	123381501	VPS37B	VPS37B, ESCRT-I subunit	33591.222
764	chr5	143541531	143572636	KCTD16	potassium channel tetramerization domain containing 16	33559.1845
765	chr11	119184732	119194510	MCAM	melanoma cell adhesion molecule	33534.6288
766	chr11	93860686	93886826	PANX1	pannexin 1	33519.322
767	chr1	59277806	59290178	LINC01135	long intergenic non-protein coding RNA 1135	33491.004
768	chr20	32374212	32403291	CHMP4B	charged multivesicular body protein 4B	33469.929
769	chr2	105940383	105956133	C2orf49	chromosome 2 open reading frame 49	33419.925
770	chr2	109780045	109803355	MIR4265	microRNA 4265	33405.561
771	chr20	50240016	50249752	NFATC2	nuclear factor of activated T-cells, cytoplasmic, calcineurin-dependent 2	33396.4272
772	chr16	86851567	86872241	FOXL1	forkhead box L1	33394.7122
773	chr10	86054160	86074705	CCSER2	coiled-coil serine rich protein 2	33346.5895
774	chr7	141349034	141377140	KIAA1147	KIAA1147	33299.9888
775	chr3	99577330	99646009	FILIP1L	filamin A interacting protein 1-like	33281.8434
776	chr9	14308527	14324168	NFIB	nuclear factor I/B	33280.9198
777	chr8	67433287	67449788	C8orf46	chromosome 8 open reading frame 46	33275.9166
778	chr19	41220174	41228905	ADCK4	aarF domain containing kinase 4	33271.2217
779	chr10	71560808	71631314	COL13A1	collagen type XIII alpha 1	33243.579
780	chr12	66318181	66358000	HMGA2	high mobility group AT-hook 2	33145.3356
781	chr2	216555908	216594850	LINC00607	long intergenic non-protein coding RNA 607	33143.5362
782	chr12	10168306	10195644	CLEC9A	C-type lectin domain family 9 member A	33117.2532
783	chr6	142617695	142665657	ADGRG6	adhesion G protein-coupled receptor G6	33112.9648
784	chr3	194297945	194315698	TMEM44-AS1	TMEM44 antisense RNA 1	33109.345
785	chr11	65678259	65688266	DRAP1	DR1 associated protein 1	33105.1574
786	chr16	87397076	87443486	FBXO31	F-box protein 31	33099.612
787	chr14	38718547	38728269	CLEC14A	C-type lectin domain family 14 member A	33088.827
788	chr7	23371703	23419064	IGF2BP3	insulin like growth factor 2 mRNA binding protein 3	33086.3946
789	chr5	66123699	66158132	MAST4	microtubule associated serine/threonine kinase family member 4	33041.9068
790	chr6	21726111	21774018	CASC15	cancer susceptibility candidate 15 (non-protein coding)	33031.8765
791	chr13	113363965	113378398	ATP11A	ATPase phospholipid transporting 11A	33016.9308
792	chr1	205565556	205581814	MFSD4A	major facilitator superfamily domain containing 4A	32984.2304
793	chr10	33226337	33252978	ITGB1	integrin subunit beta 1	32970.9016
794	chr15	90337697	90395919	MIR5094	microRNA 5094	32930.3632
795	chr17	4402305	4442011	SPNS2	spinster homolog 2 (Drosophila)	32908.3328
796	chr16	53105210	53135206	CHD9	chromodomain helicase DNA binding protein 9	32893.6136
797	chr2	27993620	28034446	MRPL33	mitochondrial ribosomal protein L33	32889.4256
798	chr11	126204366	126226969	ST3GAL4-AS1	ST3GAL4 antisense RNA 1 (head to head)	32869.2826
799	chr3	187453562	187464459	BCL6	B-cell CLL/lymphoma 6	32844.6477
800	chr16	75321500	75357754	BCAR1	breast cancer anti-estrogen resistance 1	32795.3684
801	chr14	24886739	24909521	KHNYN	KH and NYN domain containing	32783.298
802	chr18	53165228	53185115	TCF4	transcription factor 4	32741.9568
803	chr15	78325837	78370609	TBC1D2B	TBC1 domain family member 2B	32732.8092
804	chr11	9633484	9650154	SWAP70	SWAP switching B-cell complex 70kDa subunit	32678.201

Rank	chr	start	end	Gene	Gene description	H3K27ac signal
805	chr19	40922264	40951420	SERTAD3	SERTA domain containing 3	32637.2264
806	chr9	112849466	112916920	AKAP2	A-kinase anchoring protein 2	32566.7912
807	chr9	84002460	84006826	TLE1	transducin like enhancer of split 1	32533.249
808	chr9	91924125	91935890	MIR3153	microRNA 3153	32531.4015
809	chr13	31437580	31452348	MEDAG	mesenteric estrogen-dependent adipogenesis	32485.1696
810	chr2	136992354	137001490	CXCR4	chemokine (C-X-C motif) receptor 4	32476.6528
811	chr8	42109356	42159062	IKBKB	inhibitor of kappa light polypeptide gene enhancer in B-cells, kinase beta	32418.2532
812	chrX	12964454	13004127	TMSB4X	thymosin beta 4, X-linked	32412.841
813	chr11	75016703	75064229	ARRB1	arrestin, beta 1	32407.9794
814	chr3	129105748	129119309	RPL32P3	ribosomal protein L32 pseudogene 3	32387.7363
815	chr16	1581792	1600264	TMEM204	transmembrane protein 204	32374.0272
816	chr4	103729887	103751180	UBE2D3	ubiquitin conjugating enzyme E2D 3	32371.7479
817	chr2	113381328	113407385	FLJ42351	uncharacterized LOC400999	32341.9484
818	chr4	54925218	54952382	CHIC2	cysteine rich hydrophobic domain 2	32227.3696
819	chr20	46906636	46928808	LINC00494	long intergenic non-protein coding RNA 494	32158.2688
820	chr1	154451768	154475583	TDRD10	tudor domain containing 10	32107.383
821	chr3	69057260	69064279	EOGT	EGF domain-specific O-linked N-acetylglucosamine (GlcNAc) transferase	32042.4369
822	chr2	150982018	151002933	RND3	Rho family GTPase 3	32010.4075
823	chr11	65414253	65431995	MIR4489	microRNA 4489	31974.6324
824	chr1	23064479	23083980	MIR4684	microRNA 4684	31960.1889
825	chr18	59267545	59277462	CDH20	cadherin 20	31954.5574
826	chr6	21586690	21599986	SOX4	SRY-box 4	31897.104
827	chr15	75320325	75341381	PPCDC	phosphopantothenoylcysteine decarboxylase	31893.5232
828	chr9	33800419	33821432	UBE2R2	ubiquitin conjugating enzyme E2R 2	31889.3288
829	chr20	349096	363936	TRIB3	tribbles pseudokinase 3	31876.32
830	chr10	16985697	17012619	CUBN	cubilin	31867.5714
831	chr19	780709	803768	PTBP1	polypyrimidine tract binding protein 1	31862.9262
832	chr7	22755972	22770958	IL6	interleukin 6	31861.7346
833	chr19	2461021	2508526	GADD45B	growth arrest and DNA damage inducible beta	31799.847
834	chr7	143075296	143091201	ZYX	zyxin	31786.1425
835	chr3	194748841	194755276	XXYLT1	xyloside xylosyltransferase 1	31750.9335
836	chr6	30646517	30659753	NRM	nurim (nuclear envelope membrane protein)	31697.5728
837	chr15	95866794	95874474	LINC01197	long intergenic non-protein coding RNA 1197	31683.84
838	chr13	29014019	29070474	FLT1	fms related tyrosine kinase 1	31682.546
839	chr13	30086710	30140562	MTUS2	microtubule associated tumor suppressor candidate 2	31659.5908
840	chr5	173190446	173210698	CPEB4	cytoplasmic polyadenylation element binding protein 4	31657.9264
841	chr11	122026517	122074151	MIR100HG	mir-100-let-7a-2 cluster host gene	31581.342
842	chr3	99260678	99298856	COL8A1	collagen type VIII alpha 1	31542.6636
843	chr21	36246930	36262924	RUNX1	runt related transcription factor 1	31498.5836
844	chr20	39764833	39782062	PLCG1	phospholipase C gamma 1	31494.612
845	chr19	3655118	3685635	PIP5K1C	phosphatidylinositol-4-phosphate 5-kinase, type I, gamma	31490.4923
846	chr1	218632189	218673693	TGFB2-AS1	TGFB2 antisense RNA 1 (head to head)	31484.9344
847	chr7	134343786	134357768	BPGM	bisphosphoglycerate mutase	31448.3144
848	chr5	14034146	14040417	DNAH5	dynein axonemal heavy chain 5	31430.8791
849	chr18	20031688	20052817	CTAGE1	cutaneous T-cell lymphoma-associated antigen 1	31429.3875

Rank	chr	start	end	Gene	Gene description	H3K27ac signal
850	chr22	27501281	27513974	MIAT	myocardial infarction associated transcript (non-protein coding)	31426.5987
851	chr20	43269931	43275978	ADA	adenosine deaminase	31334.9493
852	chr6	26019607	26029275	HIST1H3A	histone cluster 1, H3a	31326.2536
853	chr14	100824738	100853577	WARS	tryptophanyl-tRNA synthetase	31316.2701
854	chr18	47185467	47228859	LIPG	lipase G, endothelial type	31289.9712
855	chr14	23316822	23323227	MMP14	matrix metallopeptidase 14	31248.0735
856	chr8	38236897	38245107	LETM2	leucine zipper and EF-hand containing transmembrane protein 2	31149.561
857	chr13	31469209	31483673	MEDAG	mesenteric estrogen-dependent adipogenesis	31106.2784
858	chr3	187974205	188017014	LPP	LIM domain containing preferred translocation partner in lipoma	31096.4576
859	chr3	152031952	152078765	TMEM14EP	transmembrane protein 14E, pseudogene	31074.4694
860	chr4	55794250	55812907	KDR	kinase insert domain receptor	31035.9195
861	chr13	34726279	34738009	RFC3	replication factor C subunit 3	31034.061
862	chr12	13050692	13081202	MIR614	microRNA 614	31019.517
863	chr17	46300657	46324790	MIR1203	microRNA 1203	31010.905
864	chr2	70311904	70323619	PCBP1-AS1	PCBP1 antisense RNA 1	31003.7475
865	chr5	148922409	148944942	CSNK1A1	casein kinase 1 alpha 1	30973.8618
866	chr4	74958117	74984482	CXCL2	C-X-C motif chemokine ligand 2	30970.9655
867	chr19	4557340	4577795	SEMA6B	semaphorin 6B	30954.5515
868	chr2	203237672	203245509	BMPR2	bone morphogenetic protein receptor type II	30949.8804
869	chr4	139140752	139165259	SLC7A11	solute carrier family 7 member 11	30937.6368
870	chr19	34755273	34767071	KIAA0355	KIAA0355	30934.356
871	chr1	201503948	201532721	RPS10P7	ribosomal protein S10 pseudogene 7	30922.3431
872	chr7	22149085	22170660	RAPGEF5	Rap guanine nucleotide exchange factor 5	30904.03
873	chr5	42944147	42954451	LOC648987	uncharacterized LOC648987	30883.1488
874	chr9	134268320	134313669	PRRC2B	proline rich coiled-coil 2B	30869.0643
875	chr12	31867431	31904691	AMN1	antagonist of mitotic exit network 1 homolog	30847.554
876	chr17	6913463	6922411	ALOX12-AS1	ALOX12 antisense RNA 1	30834.808
877	chr13	45491016	45510462	NUFIP1	nuclear fragile X mental retardation protein interacting protein 1	30804.4086
878	chr17	21185148	21194371	MAP2K3	mitogen-activated protein kinase kinase 3	30778.9956
879	chr16	3069845	3074828	TNFRSF12A	tumor necrosis factor receptor superfamily member 12A	30761.5539
880	chr6	11314793	11320502	NEDD9	neural precursor cell expressed, developmentally down-regulated 9	30749.8158
881	chr1	16120119	16127909	UQCRHL	ubiquinol-cytochrome c reductase hinge protein like	30736.224
882	chr3	69222027	69264209	FRMD4B	FERM domain containing 4B	30721.1506
883	chr3	49376199	49397938	GPX1	glutathione peroxidase 1	30712.8592
884	chr5	141692440	141706855	SPRY4	sprouty RTK signaling antagonist 4	30673.6785
885	chr16	15107268	15157004	NTAN1	N-terminal asparagine amidase	30617.4816
886	chr8	119067375	119126218	EXT1	exostosin glycosyltransferase 1	30586.5914
887	chr20	60929675	60958035	LAMA5	laminin subunit alpha 5	30557.9
888	chr12	122879185	122908878	CLIP1-AS1	CLIP1 antisense RNA 1	30530.3426
889	chr20	52506728	52567222	SUMO1P1	SUMO1 pseudogene 1	30519.223
890	chr11	96035220	96049448	MIR1260B	microRNA 1260b	30497.718
891	chr5	54275388	54303198	ESM1	endothelial cell specific molecule 1	30446.388
892	chr10	31099770	31111333	ZNF438	zinc finger protein 438	30428.0345
893	chr1	72746308	72753422	NEGR1	neuronal growth regulator 1	30418.7526
894	chr6	157921035	157958550	MIR3692	microRNA 3692	30409.659

Rank	chr	start	end	Gene	Gene description	H3K27ac signal
895	chr21	30534434	30592865	LINC00189	long intergenic non-protein coding RNA 189	30349.0614
896	chr8	131489679	131542160	ASAP1	ArfGAP with SH3 domain, ankyrin repeat and PH domain 1	30344.5142
897	chr16	19118465	19144771	ITPRIPL2	inositol 1,4,5-trisphosphate receptor interacting protein-like 2	30296.6202
898	chr13	42162162	42190758	MIR5006	microRNA 5006	30277.4448
899	chr18	53052938	53090760	TCF4	transcription factor 4	30246.2534
900	chr14	69542946	69569128	DCAF5	DDB1 and CUL4 associated factor 5	30219.2644
901	chr17	71286487	71309200	CDC42EP4	CDC42 effector protein 4	30201.4761
902	chr5	56728928	56748497	ACTBL2	actin, beta-like 2	30149.9583
903	chr10	73595271	73649750	ANAPC16	anaphase promoting complex subunit 16	30148.6786
904	chr3	11313460	11349694	ATG7	autophagy related 7	30117.7008
905	chr8	38643554	38666290	TACC1	transforming acidic coiled-coil containing protein 1	30107.0112
906	chr2	219146685	219163535	TMBIM1	transmembrane BAX inhibitor motif containing 1	30075.565
907	chrX	9968923	9992390	WWC3	WWC family member 3	30058.8803
908	chr10	17090606	17127859	CUBN	cubilin	30014.7421
909	chr3	149047813	149062038	TM4SF18	transmembrane 4 L six family member 18	30011.905
910	chr1	173158044	173177356	TNFSF4	tumor necrosis factor superfamily member 4	30010.848
911	chr5	88108952	88133571	MEF2C	myocyte enhancer factor 2C	29978.5563
912	chr10	80731404	80736477	ZMIZ1-AS1	ZMIZ1 antisense RNA 1	29971.7913
913	chr10	1088232	1111934	WDR37	WD repeat domain 37	29907.1836
914	chr14	34024761	34040868	NPAS3	neuronal PAS domain protein 3	29833.3854
915	chr14	91260745	91273141	TTC7B	tetratricopeptide repeat domain 7B	29811.1404
916	chr16	75372935	75387145	BCAR1	breast cancer anti-estrogen resistance 1	29801.212
917	chr1	32389209	32422735	PTP4A2	protein tyrosine phosphatase type IVA, member 2	29757.6776

Online Table VI: Super-enhancers identified by enrichment of ERG in HUVEC. Ordered by super-enhancer ranking. Chromosome (chr), start and end indicates genomic position on the GRCh37/hg19 assembly. Super-enhancer-associated gene symbols and description are indicated. ERG signal is ChIP-seq read density times length of stitched enhancer. This table spans 26 pages.

Rank	chr	start	end	Gene	Gene description	ERG signal
1	chr12	6150598	6345991	VWF	von Willebrand factor	27237.7842
2	chr2	36579345	36731285	LOC100288911	uncharacterized LOC100288911	24462.34
3	chr1	68129344	68281895	GADD45A	growth arrest and DNA damage inducible alpha	21524.9461
4	chr4	7779369	7921553	AFAP1	actin filament associated protein 1	18896.2536
5	chr3	149242874	149379253	WWTR1-AS1	WWTR1 antisense RNA 1	18820.302
6	chr3	194817758	194946736	XXYLT1-AS2	XXYLT1 antisense RNA 2	18701.81
7	chr15	67356712	67473258	SMAD3	SMAD family member 3	17948.084
8	chr3	171474760	171595496	TMEM212	transmembrane protein 212	17736.1184
9	chr8	128842258	128964124	PVT1	Pvt1 oncogene (non-protein coding)	17158.7328
10	chr20	36700423	36802692	TGM2	transglutaminase 2	16751.6622
11	chr3	57990579	58107732	FLNB	filamin B	15768.7938
12	chr3	30645151	30748081	TGFBR2	transforming growth factor beta receptor 2	15758.583
13	chr1	172851335	172961123	TNFSF18	tumor necrosis factor superfamily member 18	14019.9276
14	chr6	148683214	148788098	SASH1	SAM and SH3 domain containing 1	13802.7344
15	chr1	39567767	39680972	MACF1	microtubule-actin crosslinking factor 1	13629.882
16	chr21	27256073	27360856	APP	amyloid beta precursor protein	13579.8768
17	chr5	14141967	14238765	TRIO	trio Rho guanine nucleotide exchange factor	13503.321
18	chr13	97873415	97962677	MBNL2	muscleblind like splicing regulator 2	13469.6358
19	chr4	41121390	41220028	APBB2	amyloid beta precursor protein binding family B member 2	13375.3128
20	chr22	36709337	36796144	MYH9	myosin heavy chain 9	13290.1517
21	chr6	52353440	52443435	TRAM2-AS1	TRAM2 antisense RNA 1 (head to head)	13247.264
22	chr9	116339406	116421278	RGS3	regulator of G-protein signaling 3	13173.2048
23	chr20	49086817	49191105	PTPN1	protein tyrosine phosphatase, non-receptor type 1	12816.9952
24	chr17	62604109	62746017	SMURF2	SMAD specific E3 ubiquitin protein ligase 2	12800.1016
25	chr2	9340401	9430319	ASAP2	ArfGAP with SH3 domain, ankyrin repeat and PH domain 2	12624.4872
26	chr1	94642448	94732671	ARHGAP29	Rho GTPase activating protein 29	12378.5956
27	chr12	96753048	96844725	CDK17	cyclin dependent kinase 17	12339.7242
28	chr8	68853654	68944316	PREX2	phosphatidylinositol-3,4,5-trisphosphate dependent Rac exchange factor 2	12248.4362
29	chr5	71736476	71820549	ZNF366	zinc finger protein 366	12140.1412
30	chr1	22219550	22302670	HSPG2	heparan sulfate proteoglycan 2	12093.96
31	chr11	12177950	12263570	MICAL2	microtubule associated monooxygenase, calponin and LIM domain containing 2	12063.858
32	chr6	129943794	130032337	ARHGAP18	Rho GTPase activating protein 18	12024.1394
33	chr10	101663548	101770893	DNMBP-AS1	DNMBP antisense RNA 1	11990.4365
34	chr21	39806397	39879807	ERG	ERG, ETS transcription factor	11811.669
35	chr11	86258599	86345784	ME3	malic enzyme 3	11735.101
36	chr9	14170062	14252321	NFIB	nuclear factor I B	11614.9708
37	chr4	86681232	86766007	ARHGAP24	Rho GTPase activating protein 24	11503.9675
38	chr12	96586107	96659334	ELK3	ELK3, ETS transcription factor	11496.639
39	chr7	75920179	76023287	SSC4D	scavenger receptor cysteine rich family member with 4 domains	11434.6772

Rank	chr	start	end	Gene	Gene description	ERG signal
40	chr1	85762318	85834616	BCL10	B-cell CLL/lymphoma 10	11177.2708
41	chr2	46523336	46595382	EPAS1	endothelial PAS domain protein 1	10979.8104
42	chr12	70989165	71066393	PTPRB	protein tyrosine phosphatase, receptor type B	10927.762
43	chr10	13850061	13937676	FRMD4A	FERM domain containing 4A	10908.0675
44	chr14	55087612	55159666	SAMD4A	sterile alpha motif domain containing 4A	10793.6892
45	chr10	14587123	14660254	FAM107B	family with sequence similarity 107 member B	10567.4295
46	chr3	129294413	129347691	PLXND1	plexin D1	10565.0274
47	chr1	223885566	223965818	CAPN2	calpain 2	10496.9616
48	chr2	54772239	54842985	SPTBN1	spectrin beta, non-erythrocytic 1	10491.6318
49	chr2	238580987	238654797	LRRFIP1	LRR binding FLII interacting protein 1	10473.639
50	chr10	33501796	33577318	NRP1	neuropilin 1	10467.3492
51	chr12	66216228	66289670	RPSAP52	ribosomal protein SA pseudogene 52	10340.6336
52	chr10	71560808	71631314	COL13A1	collagen type XIII alpha 1 chain	10202.2182
53	chr10	95162521	95242935	MYOF	myoferlin	10196.4952
54	chr15	66992522	67068022	SMAD6	SMAD family member 6	10184.95
55	chr5	60558584	60631810	ZSWIM6	zinc finger SWIM-type containing 6	10149.1236
56	chr10	104354881	104418662	SUFU	SUFU negative regulator of hedgehog signaling	10134.8009
57	chr12	111833864	111887883	SH2B3	SH2B adaptor protein 3	10112.3568
58	chr14	61763760	61834459	PRKCH	protein kinase C eta	10060.4677
59	chr7	27168902	27221257	HOXA-AS3	HOXA cluster antisense RNA 3	10031.218
60	chr11	128547516	128601295	SENCR	smooth muscle and endothelial cell enriched migration/differentiation-associated long non-coding RNA	10024.4056
61	chr9	38006679	38080219	SHB	SH2 domain containing adaptor protein B	9986.732
62	chr8	6384511	6460648	ANGPT2	angiopoietin 2	9981.5607
63	chr2	159885124	159958832	TANC1	tetratricopeptide repeat, ankyrin repeat and coiled-coil containing 1	9862.1304
64	chr6	158422671	158493989	SYNJ2	synaptojanin 2	9834.7522
65	chr18	55435265	55514768	ATP8B1	ATPase phospholipid transporting 8B1	9723.2169
66	chr15	39520384	39595133	C15orf54	chromosome 15 open reading frame 54	9694.9453
67	chr9	139536090	139569757	MIR126	microRNA 126	9655.6956
68	chr2	20761375	20819862	HS1BP3	HCLS1 binding protein 3	9586.0193
69	chr21	33774813	33873638	EVA1C	eva-1 homolog C	9516.8475
70	chr2	201979149	202057923	CFLAR-AS1	CFLAR antisense RNA 1	9476.5122
71	chr5	34654263	34720278	RAI14	retinoic acid induced 14	9440.145
72	chr9	130587945	130645966	MIR4672	microRNA 4672	9364.5894
73	chr12	105820885	105884788	C12orf75	chromosome 12 open reading frame 75	9265.935
74	chr12	46822728	46889054	SLC38A2	solute carrier family 38 member 2	9239.2118
75	chr7	115953501	116019012	CAV1	caveolin 1	9230.4999
76	chr13	111022722	111096837	COL4A2	collagen type IV alpha 2 chain	9197.6715
77	chr9	112849466	112916920	AKAP2	A-kinase anchoring protein 2	9193.9802
78	chr3	111619534	111686992	PHLDB2	pleckstrin homology like domain family B member 2	9160.7964
79	chr20	23029205	23088549	CD93	CD93 molecule	9115.2384
80	chr7	107610897	107671816	LAMB1	laminin subunit beta 1	9083.0229
81	chr3	11209877	11281478	HRH1	histamine receptor H1	9057.5265
82	chr3	185394602	185465225	IGF2BP2-AS1	IGF2BP2 antisense RNA 1	9046.8063
83	chr8	134531969	134597137	ST3GAL1	ST3 beta-galactoside alpha-2,3-sialyltransferase 1	9025.768
84	chr2	161225492	161291735	MIR4785	microRNA 4785	9022.2966

Rank	chr	start	end	Gene	Gene description	ERG signal
85	chr20	45931031	45991894	LOC100131496	uncharacterized LOC100131496	9019.8966
86	chr15	60650645	60716895	ANXA2	annexin A2	9010
87	chr9	114764125	114847374	MIR4668	microRNA 4668	8982.5671
88	chr12	89737986	89786004	DUSP6	dual specificity phosphatase 6	8964.9606
89	chr15	42209440	42265576	EHD4	EH domain containing 4	8852.6472
90	chr20	30248993	30312218	BCL2L1	BCL2 like 1	8813.565
91	chr6	112522289	112576802	LAMA4	laminin subunit alpha 4	8792.9469
92	chr3	157123850	157194827	PTX3	pentraxin 3	8779.8549
93	chr3	99577330	99646009	FILIP1L	filamin A interacting protein 1 like	8729.1009
94	chr5	52278788	52342057	ITGA2	integrin subunit alpha 2	8674.1799
95	chr5	148667027	148730130	AFAP1L1	actin filament associated protein 1 like 1	8651.4213
96	chr5	172279904	172339762	ERGIC1	endoplasmic reticulum-golgi intermediate compartment 1	8583.6372
97	chr1	94485752	94539432	ABCA4	ATP binding cassette subfamily A member 4	8583.432
98	chr22	23520612	23586038	BCR	BCR, RhoGEF and GTPase activating protein	8557.7208
99	chr7	116138951	116195548	CAV1	caveolin 1	8551.8067
100	chr8	59729023	59793441	NSMAF	neutral sphingomyelinase activation associated factor	8548.2686
101	chr18	68647276	68716976	LINC01541	long intergenic non-protein coding RNA 1541	8545.22
102	chr22	39624933	39689626	PDGFB	platelet derived growth factor subunit B	8474.783
103	chr1	208371601	208419074	PLXNA2	plexin A2	8435.9521
104	chr1	100103112	100159722	PALMD	palmdelphin	8406.585
105	chr16	81505802	81561494	CMIP	c-Maf inducing protein	8398.3536
106	chr12	109195156	109252637	SSH1	slingshot protein phosphatase 1	8369.2336
107	chr6	126101265	126162051	NCOA7	nuclear receptor coactivator 7	8327.682
108	chr12	12928214	12994430	DDX47	DEAD-box helicase 47	8204.1624
109	chr9	130278993	130337520	FAM129B	family with sequence similarity 129 member B	8199.6327
110	chr8	119067375	119126218	EXT1	exostosin glycosyltransferase 1	8190.9456
111	chr22	25347837	25425402	KIAA1671	KIAA1671	8167.5945
112	chr19	39137654	39201612	ACTN4	actinin alpha 4	8167.4366
113	chr3	159540667	159603318	SCHIP1	schwannomin interacting protein 1	8138.3649
114	chr9	101078720	101139000	TBC1D2	TBC1 domain family member 2	8137.8
115	chr14	74178270	74230072	MIR4505	microRNA 4505	8127.7338
116	chr1	16463917	16509856	EPHA2	EPH receptor A2	8122.0152
117	chr6	151320145	151393521	MTHFD1L	methylenetetrahydrofolate dehydrogenase (NADP+ dependent) 1-like	8108.048
118	chr8	17503830	17557739	MTUS1	microtubule associated tumor suppressor 1	8102.5227
119	chr7	111616551	111677685	DOCK4	dedicator of cytokinesis 4	8100.255
120	chr9	127019384	127073952	NEK6	NIMA related kinase 6	8043.3232
121	chr1	235095440	235149137	SNORA14B	small nucleolar RNA, H/ACA box 14B	8038.4409
122	chr3	191045181	191097926	CCDC50	coiled-coil domain containing 50	8022.5145
123	chr14	75397956	75457164	PGF	placental growth factor	8016.7632
124	chr7	111798628	111847952	ZNF277	zinc finger protein 277	8000.3528
125	chr5	71402815	71463379	MAP1B	microtubule associated protein 1B	7994.448
126	chr5	95897241	95951367	CAST	calpastatin	7913.2212
127	chr12	13232121	13290136	GSG1	germ cell associated 1	7907.4445
128	chr2	235859137	235915831	SH3BP4	SH3 domain binding protein 4	7897.4742
129	chr16	81710741	81764822	LOC100129617	uncharacterized LOC100129617	7868.7855

Rank	chr	start	end	Gene	Gene description	ERG signal
130	chr3	14472158	14531301	GRIP2	glutamate receptor interacting protein 2	7824.6189
131	chr12	120661895	120704645	PXN	paxillin	7801.875
132	chr17	61476061	61529605	CYB561	cytochrome b561	7790.652
133	chr16	86932780	86987079	FOXL1	forkhead box L1	7786.4766
134	chr5	34570828	34629550	RAI14	retinoic acid induced 14	7780.665
135	chr1	25030753	25099953	CLIC4	chloride intracellular channel 4	7764.24
136	chr2	159974227	160026948	TANC1	tetratricopeptide repeat, ankyrin repeat and coiled-coil containing 1	7755.2591
137	chr14	68924231	68973711	ZFP36L1	ZFP36 ring finger protein like 1	7743.62
138	chr3	42054220	42116156	TRAK1	trafficking kinesin protein 1	7729.6128
139	chr5	72110560	72169936	TNPO1	transportin 1	7724.8176
140	chr11	95939449	95997914	MIR1260B	microRNA 1260b	7717.38
141	chr1	144906644	144942213	PDE4DIP	phosphodiesterase 4D interacting protein	7697.1316
142	chr13	29014019	29070474	FLT1	fms related tyrosine kinase 1	7683.5255
143	chr2	46272094	46326612	EPAS1	endothelial PAS domain protein 1	7670.6826
144	chr21	37793876	37861255	CLDN14	claudin 14	7660.9923
145	chr1	21577891	21632742	LOC100506801	uncharacterized LOC100506801	7646.2294
146	chr4	158898061	158955914	FAM198B	family with sequence similarity 198 member B	7613.4548
147	chr7	22599345	22651757	LOC100506178	uncharacterized LOC100506178	7589.2576
148	chr4	141143186	141199775	SCOC	short coiled-coil protein	7577.2671
149	chr2	192695705	192744828	SDPR	serum deprivation response	7574.7666
150	chr11	33708994	33760268	C11orf91	chromosome 11 open reading frame 91	7562.915
151	chr1	66796708	66841160	PDE4B	phosphodiesterase 4B	7556.84
152	chr9	79265360	79333580	PRUNE2	prune homolog 2	7524.666
153	chr11	108724134	108778731	DDX10	DEAD-box helicase 10	7518.0069
154	chr12	25041524	25103335	BCAT1	branched chain amino acid transaminase 1	7516.2176
155	chr4	57929006	57986466	IGFBP7-AS1	IGFBP7 antisense RNA 1	7510.022
156	chr2	188262068	188314011	CALCRL	calcitonin receptor like receptor	7505.7635
157	chr13	110949666	111001529	COL4A1	collagen type IV alpha 1 chain	7504.5761
158	chr1	214574393	214630384	PTPN14	protein tyrosine phosphatase, non-receptor type 14	7485.9967
159	chr5	77779125	77831452	SCAMP1	secretory carrier membrane protein 1	7467.0629
160	chr14	69401382	69449544	ACTN1-AS1	ACTN1 antisense RNA 1	7455.4776
161	chr7	137637767	137688169	LOC100130880	uncharacterized LOC100130880	7454.4558
162	chr2	45464108	45521250	LINC01121	long intergenic non-protein coding RNA 1121	7439.8884
163	chr20	10602727	10656474	JAG1	jagged 1	7438.5848
164	chr2	207985589	208032776	KLF7	Kruppel like factor 7	7422.5151
165	chr10	112247002	112290867	DUSP5	dual specificity phosphatase 5	7421.958
166	chr1	94749745	94801650	ARHGAP29	Rho GTPase activating protein 29	7396.4625
167	chr12	52277472	52322378	ANKRD33	ankyrin repeat domain 33	7378.0558
168	chr10	31252447	31303765	ZNF438	zinc finger protein 438	7359.0012
169	chr6	7953617	8007754	PIP5K1P1	phosphatidylinositol-4-phosphate 5-kinase type 1 pseudogene 1	7335.5635
170	chr1	56921546	56973803	PLPP3	phospholipid phosphatase 3	7331.6571
171	chr1	12192344	12247089	TNFRSF1B	TNF receptor superfamily member 1B	7324.881
172	chr5	39500217	39548277	DAB2	DAB2, clathrin adaptor protein	7290.702
173	chr17	38467102	38507762	RARA	retinoic acid receptor alpha	7290.338
174	chr2	37869801	37917334	CDC42EP3	CDC42 effector protein 3	7267.7957

Rank	chr	start	end	Gene	Gene description	ERG signal
175	chr9	112777122	112836210	AKAP2	A-kinase anchoring protein 2	7191.0096
176	chr5	139670398	139727157	HBEGF	heparin binding EGF like growth factor	7180.0135
177	chr15	68632510	68684237	ITGA11	integrin subunit alpha 11	7179.7076
178	chr11	86625033	86681653	LOC100506368	uncharacterized LOC100506368	7162.43
179	chr10	129765110	129817637	PTPRE	protein tyrosine phosphatase, receptor type E	7159.4301
180	chr10	80872033	80921265	ZMIZ1	zinc finger MIZ-type containing 1	7158.3328
181	chr2	135154002	135205383	MGAT5	mannosyl (alpha-1,6-)-glycoprotein beta-1,6-N-acetyl-glucosaminyltransferase	7157.3733
182	chr7	39626787	39674552	RALA	RAS like proto-oncogene A	7150.4205
183	chr7	100736576	100783380	SERPINE1	serpin family E member 1	7118.8884
184	chr18	3245189	3284288	MYL12B	myosin light chain 12B	7096.4685
185	chr12	11945824	11994797	ETV6	ETS variant 6	7096.1877
186	chr7	108042675	108097635	NRCAM	neuronal cell adhesion molecule	7089.84
187	chr17	2073163	2120642	SMG6	SMG6, nonsense mediated mRNA decay factor	7088.6147
188	chr13	99532268	99581959	DOCK9	dedicator of cytokinesis 9	7075.9984
189	chr15	96859967	96903973	MIR1469	microRNA 1469	7067.3636
190	chr9	37915738	37970760	SLC25A51	solute carrier family 25 member 51	7064.8248
191	chr10	112134837	112181444	SMNDC1	survival motor neuron domain containing 1	7037.657
192	chr3	11589842	11646558	VGLL4	vestigial like family member 4	7032.784
193	chr9	139401748	139446529	MIR4673	microRNA 4673	7030.617
194	chr1	19232814	19284138	IFFO2	intermediate filament family orphan 2	7015.9908
195	chr17	45333484	45394524	ITGB3	integrin subunit beta 3	7007.392
196	chr15	89632849	89687934	ABHD2	abhydrolase domain containing 2	6984.778
197	chr14	23004002	23059643	DAD1	defender against cell death 1	6982.9455
198	chr11	124745532	124791853	ROBO4	roundabout guidance receptor 4	6957.4142
199	chr18	47185467	47228859	LIPG	lipase G, endothelial type	6921.024
200	chr7	116311272	116357883	MET	MET proto-oncogene, receptor tyrosine kinase	6907.7502
201	chr15	65132749	65191149	PLEKHO2	pleckstrin homology domain containing O2	6897.04
202	chr1	19755550	19805613	CAPZB	capping actin protein of muscle Z-line beta subunit	6888.6688
203	chr20	31049560	31090763	NOL4L	nucleolar protein 4 like	6880.901
204	chr8	10632651	10677678	MIR1322	microRNA 1322	6866.6175
205	chr3	152031952	152078765	TMEM14EP	transmembrane protein 14E, pseudogene	6834.698
206	chr8	42328245	42394362	SLC20A2	solute carrier family 20 member 2	6829.8861
207	chr6	142692082	142738972	ADGRG6	adhesion G protein-coupled receptor G6	6808.428
208	chr1	156055108	156101097	LMNA	lamin A/C	6801.7731
209	chr6	142617695	142665657	ADGRG6	adhesion G protein-coupled receptor G6	6791.4192
210	chr22	24774026	24831106	ADORA2A	adenosine A2a receptor	6786.812
211	chr8	38852016	38901539	TM2D2	TM2 domain containing 2	6749.9849
212	chr6	11193176	11237658	NEDD9	neural precursor cell expressed, developmentally down-regulated 9	6743.4712
213	chr7	22670184	22717894	IL6	interleukin 6	6717.568
214	chr12	11849871	11901402	ETV6	ETS variant 6	6714.4893
215	chr22	37877566	37922556	CARD10	caspase recruitment domain family member 10	6708.009
216	chr1	151917047	151968416	S100A10	S100 calcium binding protein A10	6703.6545
217	chr21	30534434	30592865	LINC00189	long intergenic non-protein coding RNA 189	6696.1926
218	chr8	142007360	142050583	PTK2	protein tyrosine kinase 2	6677.9535
219	chr8	142126368	142175949	DENND3	DENN domain containing 3	6673.6026

Rank	chr	start	end	Gene	Gene description	ERG signal
220	chr11	75016703	75064229	ARRB1	arrestin beta 1	6667.8978
221	chr14	63640432	63683445	RHOJ	ras homolog family member J	6667.015
222	chr5	151038387	151079942	SPARC	secreted protein acidic and cysteine rich	6652.9555
223	chr17	17584029	17631609	RAI1	retinoic acid induced 1	6604.104
224	chr20	49969845	50011168	MIR3194	microRNA 3194	6603.4154
225	chr15	41196458	41235507	DLL4	delta like canonical Notch ligand 4	6591.4712
226	chr5	72898836	72952298	ARHGEF28	Rho guanine nucleotide exchange factor 28	6575.826
227	chr6	18361792	18409413	RNF144B	ring finger protein 144B	6557.4117
228	chr3	101848372	101901316	ZPLD1	zona pellucida like domain containing 1	6554.4672
229	chr13	30086710	30140562	MTUS2	microtubule associated tumor suppressor candidate 2	6553.7884
230	chr16	66390760	66423157	CDH5	cadherin 5	6550.6734
231	chr9	134508244	134558764	RAPGEF1	Rap guanine nucleotide exchange factor 1	6532.236
232	chr20	52506728	52567222	SUMO1P1	SUMO1 pseudogene 1	6497.0556
233	chr10	126723028	126775223	MIR4296	microRNA 4296	6493.058
234	chr15	90337697	90395919	MIR5094	microRNA 5094	6480.1086
235	chr10	74055316	74098640	DNAJB12	DnaJ heat shock protein family (Hsp40) member B12	6472.6056
236	chr1	101601773	101654083	S1PR1	sphingosine-1-phosphate receptor 1	6449.823
237	chr7	130565017	130607898	NA	NA	6449.3024
238	chr6	7877002	7916727	TXNDC5	thioredoxin domain containing 5	6435.45
239	chr6	136355016	136400144	PDE7B	phosphodiesterase 7B	6435.2528
240	chr1	36598548	36654745	TRAPPC3	trafficking protein particle complex 3	6428.9368
241	chr11	122026517	122069685	MIR100	microRNA 100	6423.3984
242	chr8	131489679	131542160	ASAP1	ArfGAP with SH3 domain, ankyrin repeat and PH domain 1	6407.9301
243	chr13	110867562	110920082	COL4A1	collagen type IV alpha 1 chain	6402.188
244	chr1	36807484	36854230	STK40	serine/threonine kinase 40	6390.1782
245	chr10	129704859	129748683	PTPRE	protein tyrosine phosphatase, receptor type E	6385.1568
246	chr4	95448637	95498909	PDLIM5	PDZ and LIM domain 5	6379.5168
247	chr3	145873369	145915695	PLOD2	procollagen-lysine,2-oxoglutarate 5-dioxygenase 2	6353.1326
248	chr12	54796490	54827997	ITGA5	integrin subunit alpha 5	6351.8112
249	chr5	141184158	141231551	PCDH1	protocadherin 1	6345.9227
250	chr15	39393712	39435307	C15orf54	chromosome 15 open reading frame 54	6309.9615
251	chr5	14427667	14478864	FAM105A	family with sequence similarity 105 member A	6307.4704
252	chr1	208095464	208138534	CD34	CD34 molecule	6292.527
253	chr3	50263544	50299319	GNAI2	G protein subunit alpha i2	6285.6675
254	chr2	56109410	56152051	EFEMP1	EGF containing fibulin like extracellular matrix protein 1	6263.9629
255	chr3	15332026	15374833	SH3BP5	SH3 domain binding protein 5	6262.6641
256	chr10	114804459	114854425	TCF7L2	transcription factor 7 like 2	6255.7432
257	chr15	90904797	90964446	IQGAP1	IQ motif containing GTPase activating protein 1	6251.2152
258	chr4	10089401	10126639	WDR1	WD repeat domain 1	6237.365
259	chr12	79801578	79849830	MIR1252	microRNA 1252	6229.3332
260	chr2	105984844	106027805	FHL2	four and a half LIM domains 2	6194.9762
261	chr7	23371703	23419064	IGF2BP3	insulin like growth factor 2 mRNA binding protein 3	6180.6105
262	chr12	109015324	109062920	SELPLG	selectin P ligand	6168.4416
263	chr2	161060300	161100546	ITGB6	integrin subunit beta 6	6153.6134
264	chr14	61964468	62005635	PRKCH	protein kinase C eta	6146.2331

Rank	chr	start	end	Gene	Gene description	ERG signal
265	chr15	78325837	78370609	TBC1D2B	TBC1 domain family member 2B	6133.764
266	chr11	65237697	65276784	MALAT1	metastasis associated lung adenocarcinoma transcript 1 (non-protein coding)	6132.7503
267	chr10	17027311	17074366	CUBN	cubilin	6121.8555
268	chr13	40436169	40475462	MIR4305	microRNA 4305	6110.0615
269	chr11	103456279	103505834	MIR4693	microRNA 4693	6105.176
270	chr6	12566975	12609976	PHACTR1	phosphatase and actin regulator 1	6101.8419
271	chr12	706727	756637	NINJ2	ninjurin 2	6099.002
272	chr10	94571418	94610800	EXOC6	exocyst complex component 6	6096.3336
273	chr15	39869932	39892833	THBS1	thrombospondin 1	6096.2462
274	chr17	25658699	25713076	TBC1D3P5	TBC1 domain family member 3 pseudogene 5	6090.224
275	chr20	43200748	43245818	ADA	adenosine deaminase	6088.957
276	chr10	30315030	30353229	KIAA1462	KIAA1462	6088.9206
277	chr7	107219602	107263790	BCAP29	B-cell receptor associated protein 29	6084.6876
278	chr18	46448305	46487531	SMAD7	SMAD family member 7	6032.9588
279	chr15	74273779	74318968	PML	promyelocytic leukemia	6028.2126
280	chr1	2159960	2188985	SKI	SKI proto-oncogene	6025.59
281	chr15	86121513	86165183	AKAP13	A-kinase anchoring protein 13	6013.359
282	chr12	125135279	125181009	NCOR2	nuclear receptor corepressor 2	6004.349
283	chr20	23120881	23161834	LINC00656	long intergenic non-protein coding RNA 656	6003.7098
284	chr5	141570711	141632115	SPRY4	sprouty RTK signaling antagonist 4	5999.1708
285	chr11	10312641	10352480	ADM	adrenomedullin	5991.7856
286	chr16	77598161	77631922	NUDT7	nudix hydrolase 7	5982.4492
287	chr2	28608599	28647824	FOSL2	FOS like 2, AP-1 transcription factor subunit	5981.8125
288	chr13	28952320	28988985	FLT1	fms related tyrosine kinase 1	5976.395
289	chr6	100705470	100754004	SIM1	single-minded family bHLH transcription factor 1	5974.5354
290	chr3	71552454	71601729	MIR1284	microRNA 1284	5947.4925
291	chr18	33035450	33072612	INO80C	INO80 complex subunit C	5938.4876
292	chr2	102419441	102462030	MAP4K4	mitogen-activated protein kinase kinase kinase kinase 4	5936.9066
293	chr15	63503648	63554166	APH1B	aph-1 homolog B, gamma-secretase subunit	5920.7096
294	chr4	120014010	120061776	MYOZ2	myozenin 2	5918.2074
295	chr1	59218437	59252850	LINC01135	long intergenic non-protein coding RNA 1135	5912.1534
296	chr1	47673162	47708150	TAL1	TAL bHLH transcription factor 1, erythroid differentiation factor	5895.478
297	chr13	21606726	21655987	LATS2	large tumor suppressor kinase 2	5871.9112
298	chr10	96988528	97036814	PDLIM1	PDZ and LIM domain 1	5852.2632
299	chr1	94126759	94168135	BCAR3	breast cancer anti-estrogen resistance 3	5842.2912
300	chr14	59771321	59810200	DAAM1	dishevelled associated activator of morphogenesis 1	5835.7379
301	chr10	97237939	97279206	SORBS1	sorbin and SH3 domain containing 1	5826.9004
302	chr6	34981838	35017423	ANKS1A	ankyrin repeat and sterile alpha motif domain containing 1A	5814.589
303	chr1	65326422	65366272	JAK1	Janus kinase 1	5814.115
304	chr10	104532541	104577425	WBP1L	WW domain binding protein 1-like	5807.9896
305	chr12	66318181	66358000	HMGA2	high mobility group AT-hook 2	5801.6283
306	chr15	71053164	71100652	UACA	uveal autoantigen with coiled-coil domains and ankyrin repeats	5798.2848
307	chr10	17240970	17282024	TRDMT1	tRNA aspartic acid methyltransferase 1	5796.8248
308	chr10	13715819	13759358	PRPF18	pre-mRNA processing factor 18	5795.0409
309	chr4	145546782	145589364	HHIP	hedgehog interacting protein	5782.6356

Rank	chr	start	end	Gene	Gene description	ERG signal
310	chr16	75270190	75308198	BCAR1	BCAR1, Cas family scaffolding protein	5781.0168
311	chr18	8704044	8752689	MTCL1	microtubule crosslinking factor 1	5779.026
312	chr2	64993514	65040880	SERTAD2	SERTA domain containing 2	5754.969
313	chr9	27108464	27156219	TEK	TEK receptor tyrosine kinase	5744.9265
314	chr3	149083873	149120825	TM4SF1	transmembrane 4 L six family member 1	5742.3408
315	chr5	76077074	76135449	F2RL1	F2R like trypsin receptor 1	5720.75
316	chr6	30456672	30512885	HLA-E	major histocompatibility complex, class I, E	5711.2408
317	chr11	9707628	9750951	SWAP70	SWAP switching B-cell complex 70kDa subunit	5705.6391
318	chr13	97978840	98022356	MBNL2	muscleblind like splicing regulator 2	5704.9476
319	chr13	41130535	41167057	FOXO1	forkhead box O1	5697.432
320	chr21	27477358	27523185	APP	amyloid beta precursor protein	5696.2961
321	chr8	23172880	23216185	LOC100507156	uncharacterized LOC100507156	5690.277
322	chr1	59346222	59377199	LINC01135	long intergenic non-protein coding RNA 1135	5681.1818
323	chr3	171842338	171875238	FNDC3B	fibronectin type III domain containing 3B	5678.54
324	chr7	131195597	131243357	PODXL	podocalyxin like	5664.336
325	chr14	90848345	90886727	CALM1	calmodulin 1	5645.9922
326	chr16	55558042	55603700	LPCAT2	lysophosphatidylcholine acyltransferase 2	5638.763
327	chr22	38596816	38638062	MAFF	MAF bZIP transcription factor F	5638.3282
328	chr2	216261075	216304089	FN1	fibronectin 1	5617.6284
329	chr1	162640068	162681118	DDR2	discoidin domain receptor tyrosine kinase 2	5611.535
330	chr5	39072725	39120383	RICTOR	RPTOR independent companion of MTOR complex 2	5609.3466
331	chr2	36762322	36800715	FEZ2	fasciculation and elongation protein zeta 2	5609.2173
332	chr13	28895394	28933838	FLT1	fms related tyrosine kinase 1	5608.9796
333	chr10	21602547	21662898	MIR1915	microRNA 1915	5600.5728
334	chr7	27134521	27166245	HOTAIRM1	HOXA transcript antisense RNA, myeloid-specific 1	5599.286
335	chr22	50323285	50364932	PIM3	Pim-3 proto-oncogene, serine/threonine kinase	5597.3568
336	chr3	187974205	188017014	LPP	LIM domain containing preferred translocation partner in lipoma	5590.8554
337	chr2	99306978	99348975	MGAT4A	mannosyl (alpha-1,3-)-glycoprotein beta-1,4-N-acetylglucosaminyltransferase, isozyme A	5585.601
338	chr2	43651625	43697034	THADA	THADA, armadillo repeat containing	5585.307
339	chr14	75017968	75062638	LTBP2	latent transforming growth factor beta binding protein 2	5579.283
340	chr13	99596419	99636897	DOCK9	dedicator of cytokinesis 9	5577.8684
341	chr2	161125504	161167477	ITGB6	integrin subunit beta 6	5574.0144
342	chr1	218632189	218673693	TGFB2-AS1	TGFB2 antisense RNA 1 (head to head)	5569.8368
343	chr5	16699130	16745840	MYO10	myosin X	5553.819
344	chr2	56211127	56246415	MIR216B	microRNA 216b	5550.8024
345	chr3	99352188	99390233	COL8A1	collagen type VIII alpha 1 chain	5543.1565
346	chr12	52400605	52440853	GRASP	general receptor for phosphoinositides 1 associated scaffold protein	5542.1496
347	chr6	166988327	167031931	RPS6KA2	ribosomal protein S6 kinase A2	5537.708
348	chr4	141004647	141038815	MAML3	mastermind like transcriptional coactivator 3	5535.216
349	chr10	59995571	60044501	IPMK	inositol polyphosphate multikinase	5529.09
350	chr19	41803142	41835555	HNRNPUL1	heterogeneous nuclear ribonucleoprotein U like 1	5519.9339
351	chr5	75979048	76018544	NCRUPAR	non-protein coding RNA, upstream of F2R/PAR1	5517.5912
352	chr12	124905710	124952336	NCOR2	nuclear receptor corepressor 2	5515.8558
353	chr16	69401345	69452143	TERF2	telomeric repeat binding factor 2	5511.583
354	chr11	124615820	124643202	VSIG2	V-set and immunoglobulin domain containing 2	5506.5202

Rank	chr	start	end	Gene	Gene description	ERG signal
355	chr2	216555908	216594850	LINC00607	long intergenic non-protein coding RNA 607	5506.3988
356	chr8	129032630	129073009	MIR1207	microRNA 1207	5503.6577
357	chr9	18456734	18494619	ADAMTSL1	ADAMTS like 1	5493.325
358	chr9	116261612	116291620	RGS3	regulator of G-protein signaling 3	5491.464
359	chr9	97674539	97715969	C9orf3	chromosome 9 open reading frame 3	5489.475
360	chr16	89364100	89403432	LOC100287036	uncharacterized LOC100287036	5482.8808
361	chr8	42036063	42074167	PLAT	plasminogen activator, tissue type	5471.7344
362	chr13	95735116	95790436	ABCC4	ATP binding cassette subfamily C member 4	5471.148
363	chr13	99127033	99163851	STK24	serine/threonine kinase 24	5460.1094
364	chr3	182961918	183006368	B3GNT5	UDP-GlcNAc:betaGal beta-1,3-N-acetylglucosaminyltransferase 5	5458.46
365	chr1	153721288	153763769	SLC27A3	solute carrier family 27 member 3	5454.5604
366	chr6	3795352	3834058	FAM50B	family with sequence similarity 50 member B	5449.8048
367	chr6	56554400	56598202	DST	dystonin	5448.9688
368	chr3	170027858	170084730	SKIL	SKI like proto-oncogene	5448.3376
369	chr1	32027644	32063653	TINAGL1	tubulointerstitial nephritis antigen like 1	5437.359
370	chr1	204041971	204078982	SOX13	SRY-box 13	5436.9159
371	chr12	46757347	46797912	SLC38A2	solute carrier family 38 member 2	5431.6535
372	chr10	33445447	33488222	NRP1	neuropilin 1	5428.1475
373	chr3	159478762	159516479	IQCJ-SCHIP1	IQCJ-SCHIP1 readthrough	5427.4763
374	chr16	16076415	16124453	ABCC1	ATP binding cassette subfamily C member 1	5423.4902
375	chr5	55461898	55510615	ANKRD55	ankyrin repeat domain 55	5422.2021
376	chr3	43427720	43461413	SNRK-AS1	SNRK antisense RNA 1	5411.0958
377	chr13	52977737	53025313	VPS36	vacuolar protein sorting 36 homolog	5399.876
378	chr2	225773892	225815449	DOCK10	dedicator of cytokinesis 10	5389.9429
379	chr2	178102987	178140882	MIR3128	microRNA 3128	5377.3005
380	chr11	130741168	130780749	SNX19	sorting nexin 19	5375.0998
381	chr11	86166775	86197958	CCDC81	coiled-coil domain containing 81	5372.8309
382	chr9	75732722	75774461	ANXA1	annexin A1	5363.4615
383	chr9	128393381	128439454	MAPKAP1	mitogen-activated protein kinase associated protein 1	5353.6826
384	chr1	38460584	38496741	FHL3	four and a half LIM domains 3	5351.236
385	chr6	46842811	46887859	ADGRF5	adhesion G protein-coupled receptor F5	5338.188
386	chr6	36702660	36739224	CPNE5	copine 5	5334.6876
387	chr5	147758113	147798192	FBXO38	F-box protein 38	5314.4754
388	chr12	125196895	125232693	SCARB1	scavenger receptor class B member 1	5308.8434
389	chr9	134268320	134313669	PRRC2B	proline rich coiled-coil 2B	5305.833
390	chr12	96864863	96910767	CDK17	cyclin dependent kinase 17	5301.912
391	chr3	71083083	71117008	FOXP1	forkhead box P1	5299.085
392	chr17	38253769	38292089	MSL1	male specific lethal 1 homolog	5288.16
393	chr16	87397076	87443486	FBXO31	F-box protein 31	5286.099
394	chr7	43675602	43715196	STK17A	serine/threonine kinase 17a	5281.8396
395	chr7	107918917	107954929	NRCAM	neuronal cell adhesion molecule	5279.3592
396	chr2	151319138	151349487	RND3	Rho family GTPase 3	5274.6562
397	chr20	1350206	1382567	FKBP1A-SDCBP2	FKBP1A-SDCBP2 readthrough (NMD candidate)	5258.6625
398	chr1	8432498	8474031	RERE	arginine-glutamic acid dipeptide repeats	5253.9245
399	chr16	82659979	82692499	CDH13	cadherin 13	5251.98

Rank	chr	start	end	Gene	Gene description	ERG signal
400	chr10	8081461	8109662	GATA3	GATA binding protein 3	5251.0262
401	chr1	98779454	98818023	LOC729987	uncharacterized LOC729987	5249.2409
402	chr15	101706473	101742517	CHSY1	chondroitin sulfate synthase 1	5248.0064
403	chr1	234734161	234769328	LINC00184	long intergenic non-protein coding RNA 184	5243.3997
404	chr8	42109356	42159062	IKBKB	inhibitor of kappa light polypeptide gene enhancer in B-cells, kinase beta	5239.0124
405	chr3	16091523	16128598	GALNT15	polypeptide N-acetylgalactosaminyltransferase 15	5234.99
406	chr3	99260678	99298856	COL8A1	collagen type VIII alpha 1 chain	5234.2038
407	chr9	73020184	73055287	KLF9	Kruppel like factor 9	5233.8573
408	chr7	22456641	22486999	STEAP1B	STEAP family member 1B	5230.6834
409	chr11	57528249	57570724	CTNND1	catenin delta 1	5224.425
410	chr2	206544825	206579157	NRP2	neuropilin 2	5221.8972
411	chr1	16248952	16294373	ZBTB17	zinc finger and BTB domain containing 17	5205.2466
412	chr13	21360958	21403451	EEF1AKMT1	eukaryotic translation elongation factor 1 alpha lysine methyltransferase 1	5201.1432
413	chr17	4402305	4442011	SPNS2	sphingolipid transporter 2	5193.5448
414	chr6	72092870	72131383	MIR30A	microRNA 30a	5191.5524
415	chr18	53052938	53090760	TCF4	transcription factor 4	5185.3962
416	chr8	22905960	22950966	LOC254896	uncharacterized LOC254896	5184.6912
417	chr2	28805346	28846017	PLB1	phospholipase B1	5181.4854
418	chr3	39186176	39223111	CSRNP1	cysteine and serine rich nuclear protein 1	5174.5935
419	chr3	30551398	30592399	TGFBR2	transforming growth factor beta receptor 2	5174.3262
420	chr19	2461021	2508526	GADD45B	growth arrest and DNA damage inducible beta	5168.544
421	chr1	101700210	101731414	S1PR1	sphingosine-1-phosphate receptor 1	5167.3824
422	chr6	143246919	143282358	HIVEP2	human immunodeficiency virus type I enhancer binding protein 2	5167.0062
423	chr14	73108164	73147405	DPF3	double PHD fingers 3	5164.1156
424	chr13	95905533	95954644	ABCC4	ATP binding cassette subfamily C member 4	5161.5661
425	chr15	93159265	93199772	FAM174B	family with sequence similarity 174 member B	5160.5918
426	chr9	16687492	16728370	BNC2	basonuclin 2	5158.8036
427	chr22	31605437	31641698	LIMK2	LIM domain kinase 2	5152.6881
428	chr15	35964208	35999227	DPH6	diphthamine biosynthesis 6	5147.793
429	chr4	15653349	15694213	FBXL5	F-box and leucine rich repeat protein 5	5144.7776
430	chr1	90155902	90191826	LRRC8C	leucine rich repeat containing 8 family member C	5129.9472
431	chr20	20710803	20747438	RALGAPA2	Ral GTPase activating protein catalytic alpha subunit 2	5121.573
432	chr17	73670335	73704724	SAP30BP	SAP30 binding protein	5120.5221
433	chr17	47891374	47929568	KAT7	lysine acetyltransferase 7	5114.1766
434	chr6	21726111	21774018	CASC15	cancer susceptibility candidate 15 (non-protein coding)	5111.6769
435	chr16	55504280	55540769	MMP2	matrix metallopeptidase 2	5108.46
436	chr1	236134498	236174786	NID1	nidogen 1	5092.4032
437	chr1	234852748	234882254	LINC01132	long intergenic non-protein coding RNA 1132	5089.785
438	chr9	676520	717070	KANK1	KN motif and ankyrin repeat domains 1	5089.025
439	chr19	11241065	11291803	SPC24	SPC24, NDC80 kinetochore complex component	5078.8738
440	chr17	80801535	80845650	ZNF750	zinc finger protein 750	5077.6365
441	chr3	158361937	158403898	LXN	latexin	5077.281
442	chr11	128041074	128082002	ETS1	ETS proto-oncogene 1, transcription factor	5075.072
443	chrX	45602036	45669243	MIR221	microRNA 221	5074.1285
444	chr7	55600013	55642793	VOPP1	vesicular, overexpressed in cancer, prosurvival protein 1	5073.708

Rank	chr	start	end	Gene	Gene description	ERG signal
445	chr5	39393909	39428614	DAB2	DAB2, clathrin adaptor protein	5066.93
446	chr14	75103235	75140903	LTBP2	latent transforming growth factor beta binding protein 2	5066.346
447	chr3	120134545	120170855	FSTL1	follistatin like 1	5065.245
448	chr8	55046974	55093698	MRPL15	mitochondrial ribosomal protein L15	5064.8816
449	chr6	1770280	1803723	FOXC1	forkhead box C1	5063.2702
450	chr3	46966789	47004011	CCDC12	coiled-coil domain containing 12	5062.192
451	chr8	13208329	13242131	DLC1	DLC1 Rho GTPase activating protein	5056.7792
452	chr2	231621362	231661962	CAB39	calcium binding protein 39	5054.7
453	chr17	67564956	67606295	MAP2K6	mitogen-activated protein kinase kinase 6	5051.6258
454	chr12	50037880	50069731	FMNL3	formin like 3	5035.6431
455	chr12	59433294	59473992	LRIG3	leucine rich repeats and immunoglobulin like domains 3	5030.2728
456	chr2	27993620	28034446	MRPL33	mitochondrial ribosomal protein L33	5025.6806
457	chr1	192490360	192530586	RGS1	regulator of G-protein signaling 1	5024.2274
458	chr3	132122767	132165264	DNAJC13	DnaJ heat shock protein family (Hsp40) member C13	5023.1454
459	chr17	15381962	15420999	CDRT4	CMT1A duplicated region transcript 4	5020.1582
460	chr3	69222027	69264209	FRMD4B	FERM domain containing 4B	5019.658
461	chr18	52985727	53020003	TCF4	transcription factor 4	5018.0064
462	chr19	16425720	16463285	KLF2	Kruppel like factor 2	5014.9275
463	chr2	192758779	192795677	SDPR	serum deprivation response	5014.4382
464	chr3	141107232	141146918	ZBTB38	zinc finger and BTB domain containing 38	5004.4046
465	chr3	99406186	99442758	COL8A1	collagen type VIII alpha 1 chain	4995.7352
466	chr14	77490511	77527029	IRF2BPL	interferon regulatory factor 2 binding protein like	4988.3588
467	chr8	29594076	29632108	LINC00589	long intergenic non-protein coding RNA 589	4966.9792
468	chr22	25506006	25543365	LOC100128531	uncharacterized LOC100128531	4965.0111
469	chr18	52900635	52935540	TCF4	transcription factor 4	4963.491
470	chr8	23000610	23039961	TNFRSF10D	TNF receptor superfamily member 10d	4962.1611
471	chr12	6430963	6457470	TNFRSF1A	TNF receptor superfamily member 1A	4959.4597
472	chr5	180211826	180245081	MGAT1	mannosyl (alpha-1,3-)-glycoprotein beta-1,2-N-acetylglucosaminyltransferase	4954.995
473	chr15	57408983	57446631	TCF12	transcription factor 12	4950.712
474	chr8	141826821	141865802	PTK2	protein tyrosine kinase 2	4950.587
475	chr5	34486223	34527662	RAI14	retinoic acid induced 14	4947.8166
476	chr10	73595271	73649750	ANAPC16	anaphase promoting complex subunit 16	4946.6932
477	chr4	85772482	85809999	WDFY3	WD repeat and FYVE domain containing 3	4944.7406
478	chr20	19781960	19821850	RIN2	Ras and Rab interactor 2	4938.382
479	chr3	157072605	157108241	PTX3	pentraxin 3	4932.0224
480	chr6	144664006	144701029	UTRN	utrophin	4931.4636
481	chr8	23384979	23420264	SLC25A37	solute carrier family 25 member 37	4925.786
482	chr19	4367253	4404218	CHAF1A	chromatin assembly factor 1 subunit A	4916.345
483	chr8	8186677	8222693	SGK223	homolog of rat pragma of Rnd2	4916.184
484	chr4	37648842	37685250	RELL1	RELT like 1	4907.7984
485	chr21	46497948	46531423	ADARB1	adenosine deaminase, RNA specific B1	4907.435
486	chr5	138836915	138876666	TMEM173	transmembrane protein 173	4905.2734
487	chr4	40991513	41032622	APBB2	amyloid beta precursor protein binding family B member 2	4900.1928
488	chr11	73017243	73047294	ARHGEF17	Rho guanine nucleotide exchange factor 17	4898.313
489	chr19	38488345	38536099	SIPA1L3	signal induced proliferation associated 1 like 3	4894.785

Rank	chr	start	end	Gene	Gene description	ERG signal
490	chr2	112654654	112700556	MERTK	MER proto-oncogene, tyrosine kinase	4893.1532
491	chr10	82202296	82238765	TSPAN14	tetraspanin 14	4890.4929
492	chr3	57934962	57971838	FLNB	filamin B	4886.07
493	chr1	78535927	78570988	GIPC2	GIPC PDZ domain containing family member 2	4880.4912
494	chr7	134518453	134552971	CALD1	caldesmon 1	4877.3934
495	chr6	11585122	11619428	TMEM170B	transmembrane protein 170B	4874.8826
496	chr17	47073053	47113806	IGF2BP1	insulin like growth factor 2 mRNA binding protein 1	4869.9835
497	chr11	71638184	71675042	LOC100133315	transient receptor potential cation channel, subfamily C, member 2-like	4868.9418
498	chr13	77426300	77461597	KCTD12	potassium channel tetramerization domain containing 12	4867.4563
499	chr8	13100908	13135549	DLC1	DLC1 Rho GTPase activating protein	4863.5964
500	chr8	32403789	32442545	NRG1	neuregulin 1	4848.3756
501	chr19	45088988	45130994	IGSF23	immunoglobulin superfamily member 23	4843.2918
502	chr3	62546655	62594547	CADPS	calcium dependent secretion activator	4841.8812
503	chr10	123870693	123910081	TACC2	transforming acidic coiled-coil containing protein 2	4840.7852
504	chr6	17829101	17871005	KIF13A	kinesin family member 13A	4839.912
505	chr14	50786960	50831315	ATP5S	ATP synthase, H+ transporting, mitochondrial Fo complex subunit s (factor B)	4839.1305
506	chr10	33589872	33626997	NRP1	neuropilin 1	4829.9625
507	chr14	23529870	23566706	ACIN1	apoptotic chromatin condensation inducer 1	4829.1996
508	chr18	22806590	22835080	ZNF521	zinc finger protein 521	4826.206
509	chr2	1710953	1760482	PXDN	peroxidasin	4824.1246
510	chr12	75943799	75980152	KRR1	KRR1, small subunit processome component homolog	4824.0431
511	chr15	99431644	99468676	IGF1R	insulin like growth factor 1 receptor	4821.5664
512	chr2	101491514	101525227	NPAS2	neuronal PAS domain protein 2	4817.5877
513	chr4	141626150	141665343	TBC1D9	TBC1 domain family member 9	4816.8197
514	chr5	57534553	57570533	PLK2	polo like kinase 2	4814.124
515	chr2	47076143	47110630	LINC01119	long intergenic non-protein coding RNA 1119	4807.4878
516	chr2	187425987	187470811	ITGAV	integrin subunit alpha V	4805.1328
517	chr19	16175998	16208100	TPM4	tropomyosin 4	4799.249
518	chr3	81772370	81812120	GBE1	1,4-alpha-glucan branching enzyme 1	4789.875
519	chr1	165851021	165881423	MIR3658	microRNA 3658	4788.315
520	chr11	47414747	47449338	PSMC3	proteasome 26S subunit, ATPase 3	4787.3944
521	chr19	13260177	13284020	STX10	syntaxin 10	4785.2901
522	chr2	189155080	189189457	GULP1	GULP, engulfment adaptor PTB domain containing 1	4785.2784
523	chrX	114793741	114877731	PLS3	plastin 3	4779.031
524	chr6	44007801	44041264	C6orf223	chromosome 6 open reading frame 223	4778.5164
525	chr8	93081545	93117088	RUNX1T1	RUNX1 translocation partner 1	4773.4249
526	chr14	51986189	52022536	FRMD6	FERM domain containing 6	4768.7264
527	chr9	75601366	75639652	ALDH1A1	aldehyde dehydrogenase 1 family member A1	4766.607
528	chr1	201503948	201532721	RPS10P7	ribosomal protein S10 pseudogene 7	4761.9315
529	chr3	15808578	15849135	ANKRD28	ankyrin repeat domain 28	4761.3918
530	chr9	6412028	6452028	UHRF2	ubiquitin like with PHD and ring finger domains 2	4756
531	chr4	169329139	169373745	DDX60L	DEAD-box helicase 60-like	4754.9996
532	chr1	231522153	231559441	EGLN1	egl-9 family hypoxia inducible factor 1	4746.7624
533	chr10	29922088	29959160	SVIL	supervillin	4745.216
534	chr10	74002103	74036785	DDIT4	DNA damage inducible transcript 4	4741.0294

Rank	chr	start	end	Gene	Gene description	ERG signal
535	chr13	76335103	76373142	LMO7	LIM domain 7	4735.8555
536	chr21	43345567	43382942	C2CD2	C2 calcium dependent domain containing 2	4731.675
537	chr4	160192576	160231043	RAPGEF2	Rap guanine nucleotide exchange factor 2	4731.441
538	chr4	140810997	140844480	MGST2	microsomal glutathione S-transferase 2	4731.1479
539	chr5	172174129	172206194	DUSP1	dual specificity phosphatase 1	4726.381
540	chr5	66123699	66158132	MAST4	microtubule associated serine/threonine kinase family member 4	4720.7643
541	chr3	45672008	45712383	LIMD1-AS1	LIMD1 antisense RNA 1	4711.7625
542	chr5	73034451	73071905	ARHGEF28	Rho guanine nucleotide exchange factor 28	4711.7132
543	chr20	37427180	37460293	PPP1R16B	protein phosphatase 1 regulatory subunit 16B	4708.6686
544	chr2	201240167	201274031	SPATS2L	spermatogenesis associated serine rich 2 like	4707.096
545	chr2	182642509	182679441	SSFA2	sperm specific antigen 2	4686.6708
546	chr6	157921035	157958550	MIR3692	microRNA 3692	4681.872
547	chr11	69059906	69089967	MYEOV	myeloma overexpressed	4677.4916
548	chr10	17090606	17127859	CUBN	cubilin	4675.2515
549	chr12	106455932	106486421	NUAK1	NUAK family kinase 1	4670.9148
550	chr21	46711695	46741729	LOC642852	uncharacterized LOC642852	4670.287
551	chr11	64618415	64647572	EHD1	EH domain containing 1	4665.12
552	chr1	156445853	156476372	MEF2D	myocyte enhancer factor 2D	4660.2513
553	chr1	8228959	8278094	SLC45A1	solute carrier family 45 member 1	4657.998
554	chr5	133884985	133918251	JADE2	jade family PHD finger 2	4657.24
555	chr16	75321500	75357754	BCAR1	BCAR1, Cas family scaffolding protein	4647.7628
556	chr22	37624204	37663752	RAC2	ras-related C3 botulinum toxin substrate 2 (rho family, small GTP binding protein Rac2)	4646.89
557	chr6	143139893	143169601	HIVEP2	human immunodeficiency virus type I enhancer binding protein 2	4646.3312
558	chr2	232023973	232057781	ARMC9	armadillo repeat containing 9	4645.2192
559	chr15	48827010	48860105	FBN1	fibrillin 1	4643.2285
560	chr18	51735540	51779013	SNORA37	small nucleolar RNA, H/ACA box 37	4629.8745
561	chr1	201453974	201477419	CSRP1	cysteine and glycine rich protein 1	4628.043
562	chr17	57829891	57875903	MIR21	microRNA 21	4624.206
563	chr20	6541310	6573333	BMP2	bone morphogenetic protein 2	4601.7051
564	chr3	168932711	168964325	MECOM	MDS1 and EVI1 complex locus	4590.3528
565	chr12	48193067	48216827	HDAC7	histone deacetylase 7	4588.056
566	chr12	27700958	27732556	PPFIBP1	PPFIA binding protein 1	4588.0296
567	chr6	151176897	151217964	MTHFD1L	methylenetetrahydrofolate dehydrogenase (NADP+ dependent) 1-like	4587.1839
568	chr8	55221482	55252806	SOX17	SRY-box 17	4582.7012
569	chr6	111782983	111817348	REV3L	REV3 like, DNA directed polymerase zeta catalytic subunit	4577.418
570	chr6	21913807	21955197	CASC15	cancer susceptibility candidate 15 (non-protein coding)	4569.456
571	chr10	116382745	116415227	ABLIM1	actin binding LIM protein 1	4563.721
572	chr12	123589198	123636651	PITPNM2	phosphatidylinositol transfer protein membrane associated 2	4560.2333
573	chr18	60087029	60119539	TNFRSF11A	TNF receptor superfamily member 11a	4557.902
574	chr4	90796838	90829530	MMRN1	multimerin 1	4553.9956
575	chr12	50576851	50619051	LIMA1	LIM domain and actin binding 1	4553.38
576	chr3	170443413	170478626	RPL22L1	ribosomal protein L22 like 1	4549.5196
577	chr11	95869569	95903004	MIR1260B	microRNA 1260b	4547.16
578	chr12	52607179	52639216	KRT7	keratin 7	4546.0503
579	chr17	1964953	1995350	SMG6	SMG6, nonsense mediated mRNA decay factor	4544.3515

Rank	chr	start	end	Gene	Gene description	ERG signal
580	chr1	23879628	23908578	MDS2	myelodysplastic syndrome 2 translocation associated	4539.36
581	chr12	124851552	124879957	NCOR2	nuclear receptor corepressor 2	4533.438
582	chr9	140188102	140217008	NRARP	NOTCH-regulated ankyrin repeat protein	4532.4608
583	chr16	67570103	67607064	CTCF	CCCTC-binding factor	4531.4186
584	chr18	8776869	8815094	MTCL1	microtubule crosslinking factor 1	4529.6625
585	chr20	48752880	48789894	TMEM189	transmembrane protein 189	4523.1108
586	chr12	52557396	52589292	KRT80	keratin 80	4519.6632
587	chr3	32439956	32465877	CMTM7	CKLF like MARVEL transmembrane domain containing 7	4512.8461
588	chr17	32561260	32587095	CCL2	C-C motif chemokine ligand 2	4510.791
589	chr18	12271312	12311399	TUBB6	tubulin beta 6 class V	4509.7875
590	chr7	90223291	90256274	CDK14	cyclin dependent kinase 14	4502.1795
591	chr17	73840861	73875143	WBP2	WW domain binding protein 2	4501.2266
592	chr6	163755538	163790262	DKFZp451B082	uncharacterized LOC401282	4500.2304
593	chr12	46915712	46958397	SLC38A2	solute carrier family 38 member 2	4498.999
594	chr17	80173445	80206085	SLC16A3	solute carrier family 16 member 3	4497.792
595	chr3	62119129	62157327	PTPRG	protein tyrosine phosphatase, receptor type G	4484.4452
596	chr12	53432362	53462602	TNS2	tensin 2	4481.568
597	chr4	114356488	114391501	ANK2	ankyrin 2	4471.1601
598	chr2	102350953	102385911	MAP4K4	mitogen-activated protein kinase kinase kinase kinase 4	4464.1366
599	chr11	11996598	12028451	DKK3	dickkopf WNT signaling pathway inhibitor 3	4453.0494
600	chr9	134581825	134615685	RAPGEF1	Rap guanine nucleotide exchange factor 1	4452.59
601	chr13	32827902	32860749	FRY	FRY microtubule binding protein	4450.7685
602	chr20	1301744	1326255	SDCBP2-AS1	SDCBP2 antisense RNA 1	4443.8443
603	chr1	2205847	2247261	LOC100129534	small nuclear ribonucleoprotein polypeptide N pseudogene	4443.7222
604	chr1	154451768	154475583	TDRD10	tudor domain containing 10	4441.4975
605	chr9	124505038	124542790	DAB2IP	DAB2 interacting protein	4439.6352
606	chr11	114150710	114180316	NNMT	nicotinamide N-methyltransferase	4432.0182
607	chr10	71204786	71242120	TSPAN15	tetraspanin 15	4431.5458
608	chr8	8141652	8173485	FAM86B3P	family with sequence similarity 86, member A pseudogene	4431.1536
609	chr8	80679387	80709466	HEY1	hes related family bHLH transcription factor with YRPW motif 1	4430.6367
610	chr16	81638015	81669172	CMIP	c-Maf inducing protein	4430.5254
611	chr22	44415533	44456138	PARVB	parvin beta	4430.0055
612	chr15	62399537	62431639	C2CD4B	C2 calcium dependent domain containing 4B	4426.8658
613	chr15	89163947	89194984	ISG20	interferon stimulated exonuclease gene 20	4422.7725
614	chr10	105647010	105679224	OBFC1	oligonucleotide/oligosaccharide binding fold containing 1	4410.0966
615	chr18	72910729	72943788	TSHZ1	teashirt zinc finger homeobox 1	4406.7647
616	chr7	84297215	84332826	SEMA3D	semaphorin 3D	4405.0807
617	chr2	216692935	216717695	LINC00607	long intergenic non-protein coding RNA 607	4394.9
618	chr14	100196359	100226465	EML1	echinoderm microtubule associated protein like 1	4392.4654
619	chr14	35852457	35889861	NFKBIA	NFKB inhibitor alpha	4391.2296
620	chr1	84608530	84642461	PRKACB	protein kinase cAMP-activated catalytic subunit beta	4390.6714
621	chr9	120439196	120470902	TLR4	toll like receptor 4	4388.1104
622	chr8	41980787	42013983	AP3M2	adaptor related protein complex 3 mu 2 subunit	4385.1916
623	chr12	125397715	125426199	MIR5188	microRNA 5188	4383.6876
624	chr11	122573352	122609473	UBASH3B	ubiquitin associated and SH3 domain containing B	4377.8652

Rank	chr	start	end	Gene	Gene description	ERG signal
625	chr6	148625044	148669159	SASH1	SAM and SH3 domain containing 1	4376.208
626	chr12	66022868	66054582	HMGA2	high mobility group AT-hook 2	4360.675
627	chr1	68295105	68323002	GNG12-AS1	GNG12 antisense RNA 1	4349.1423
628	chr1	221982171	222014866	DUSP10	dual specificity phosphatase 10	4348.435
629	chr8	49319045	49346166	EFCAB1	EF-hand calcium binding domain 1	4347.4963
630	chr10	3109332	3148029	PFKP	phosphofructokinase, platelet	4345.6731
631	chr15	77773007	77801872	HMG20A	high mobility group 20A	4344.1825
632	chr22	19954080	19991174	ARVCF	armadillo repeat gene deleted in velocardiofacial syndrome	4339.998
633	chr14	73675149	73713315	PAPLN	papilin, proteoglycan like sulfated glycoprotein	4335.6576
634	chr14	69237210	69263600	ZFP36L1	ZFP36 ring finger protein like 1	4333.238
635	chr17	15847433	15886293	ADORA2B	adenosine A2b receptor	4317.346
636	chr7	139734253	139763720	PARP12	poly(ADP-ribose) polymerase family member 12	4316.9155
637	chr11	60658352	60687832	PRPF19	pre-mRNA processing factor 19	4309.976
638	chr9	16500406	16534123	BNC2	basonuclin 2	4309.0326
639	chr16	86419794	86450283	LINC00917	long intergenic non-protein coding RNA 917	4308.0957
640	chr12	93394534	93428222	EEA1	early endosome antigen 1	4305.3264
641	chr18	19746590	19774999	GATA6-AS1	GATA6 antisense RNA 1 (head to head)	4303.9635
642	chr1	198878255	198907788	MIR181A1HG	MIR181A1 host gene	4300.0048
643	chr4	74958117	74984482	CXCL2	C-X-C motif chemokine ligand 2	4297.495
644	chr1	23423442	23460165	LUZP1	leucine zipper protein 1	4289.2464
645	chr15	52272948	52314792	MAPK6	mitogen-activated protein kinase 6	4289.01
646	chr16	57660900	57688644	ADGRG1	adhesion G protein-coupled receptor G1	4286.448
647	chr4	170164780	170193860	SH3RF1	SH3 domain containing ring finger 1	4286.392
648	chr8	13048845	13083347	DLC1	DLC1 Rho GTPase activating protein	4285.1484
649	chr18	22853702	22880869	ZNF521	zinc finger protein 521	4284.2359
650	chr12	12013795	12046088	ETV6	ETS variant 6	4282.0518
651	chr13	41608291	41650382	WBP4	WW domain binding protein 4	4280.6547
652	chr13	114872138	114898518	RASA3	RAS p21 protein activator 3	4278.836
653	chr7	115898195	115929818	CAV2	caveolin 2	4278.5919
654	chr3	16508745	16539038	RFTN1	raftlin, lipid raft linker 1	4277.3716
655	chr5	143541531	143572636	KCTD16	potassium channel tetramerization domain containing 16	4273.827
656	chr12	31867431	31904691	AMN1	antagonist of mitotic exit network 1 homolog	4273.722
657	chr1	163115999	163145636	RGS5	regulator of G-protein signaling 5	4267.728
658	chr18	20836566	20863493	TMEM241	transmembrane protein 241	4265.2368
659	chr4	38664315	38691862	KLF3	Kruppel like factor 3	4258.7662
660	chr18	21407219	21442950	LAMA3	laminin subunit alpha 3	4255.5621
661	chr8	145001890	145027401	PLEC	plectin	4255.2348
662	chr3	15670418	15691589	ANKRD28	ankyrin repeat domain 28	4253.2539
663	chr8	125248667	125285489	TMEM65	transmembrane protein 65	4249.2588
664	chr10	14025589	14053301	FRMD4A	FERM domain containing 4A	4245.4784
665	chr8	106635515	106673168	ZFPM2	zinc finger protein, FOG family member 2	4243.4931
666	chr4	87998537	88030657	AFF1	AF4/FMR2 family member 1	4239.84
667	chr3	50192378	50210933	SEMA3F	semaphorin 3F	4237.962
668	chr9	133707819	133739533	ABL1	ABL proto-oncogene 1, non-receptor tyrosine kinase	4236.9904
669	chr2	43132807	43157673	HAAO	3-hydroxyanthranilate 3,4-dioxygenase	4232.1932

Rank	chr	start	end	Gene	Gene description	ERG signal
670	chr12	59306175	59336849	LRIG3	leucine rich repeats and immunoglobulin like domains 3	4229.9446
671	chr3	153316720	153348420	C3orf79	chromosome 3 open reading frame 79	4228.78
672	chr3	141242507	141277110	RASA2	RAS p21 protein activator 2	4221.566
673	chr6	11409740	11440242	NEDD9	neural precursor cell expressed, developmentally down-regulated 9	4221.4768
674	chr11	12131065	12161772	MICAL2	microtubule associated monooxygenase, calponin and LIM domain containing 2	4219.1418
675	chr8	94884853	94919572	MIR378D2	microRNA 378d-2	4211.4147
676	chr16	89554103	89599361	ANKRD11	ankyrin repeat domain 11	4208.994
677	chr20	47432221	47464202	PREX1	phosphatidylinositol-3,4,5-trisphosphate dependent Rac exchange factor 1	4208.6996
678	chr8	10566986	10589116	SOX7	SRY-box 7	4202.487
679	chr17	55944220	55972067	CUEDC1	CUE domain containing 1	4199.3276
680	chr10	54067865	54096882	PRKG1-AS1	PRKG1 antisense RNA 1	4195.8582
681	chr1	205239318	205284076	NUAK2	NUAK family kinase 2	4189.3488
682	chr10	71103138	71139805	HK1	hexokinase 1	4187.3714
683	chr5	150442022	150475957	TNIP1	TNFAIP3 interacting protein 1	4180.792
684	chr18	3445975	3479417	TGIF1	TGFB induced factor homeobox 1	4180.25
685	chr3	25520141	25552824	RARB	retinoic acid receptor beta	4180.1557
686	chr10	33226337	33252978	ITGB1	integrin subunit beta 1	4177.3088
687	chr4	120125332	120155665	USP53	ubiquitin specific peptidase 53	4176.8541
688	chr2	197123425	197160771	HECW2	HECT, C2 and WW domain containing E3 ubiquitin protein ligase 2	4171.5482
689	chr4	3323331	3357691	RGS12	regulator of G-protein signaling 12	4171.304
690	chr13	77873780	77905307	MYCBP2	MYC binding protein 2, E3 ubiquitin protein ligase	4171.0221
691	chr4	186682136	186715396	SORBS2	sorbin and SH3 domain containing 2	4167.478
692	chr1	39844584	39878154	KIAA0754	KIAA0754	4162.68
693	chr1	59527257	59551695	HSD52	uncharacterized LOC729467	4152.0162
694	chr3	111564152	111594186	PHLDB2	pleckstrin homology like domain family B member 2	4141.6886
695	chr1	65380615	65417062	JAK1	Janus kinase 1	4140.3792
696	chr20	60929675	60958035	LAMA5	laminin subunit alpha 5	4137.724
697	chr4	129423270	129451406	PGRMC2	progesterone receptor membrane component 2	4133.1784
698	chr6	2102660	2128946	GMDS	GDP-mannose 4,6-dehydratase	4129.5306
699	chr1	95427673	95460737	LOC729970	hCG2028352-like	4126.3872
700	chr17	76800297	76838035	USP36	ubiquitin specific peptidase 36	4124.7634
701	chr1	219469898	219504731	LYPLAL1	lysophospholipase like 1	4124.2272
702	chr7	116209723	116234155	CAV1	caveolin 1	4124.1216
703	chr11	65183244	65201127	NEAT1	nuclear paraspeckle assembly transcript 1 (non-protein coding)	4123.8198
704	chr17	62078249	62103649	ICAM2	intercellular adhesion molecule 2	4122.42
705	chr1	33445542	33473551	RNF19B	ring finger protein 19B	4117.323
706	chr11	128361680	128389726	ETS1	ETS proto-oncogene 1, transcription factor	4114.3482
707	chr17	25877308	25907782	KSR1	kinase suppressor of ras 1	4113.99
708	chr2	70140474	70174476	MXD1	MAX dimerization protein 1	4110.8418
709	chr17	56061666	56085952	SRSF1	serine and arginine rich splicing factor 1	4106.7626
710	chr6	132380260	132409920	LINC01013	long intergenic non-protein coding RNA 1013	4104.944
711	chr14	69500314	69527724	ACTN1-AS1	ACTN1 antisense RNA 1	4103.277
712	chr12	95997866	96033544	PGAM1P5	phosphoglycerate mutase 1 pseudogene 5	4099.4022
713	chr12	26829765	26859907	ITPR2	inositol 1,4,5-trisphosphate receptor type 2	4099.312
714	chr1	21648348	21673112	ECE1	endothelin converting enzyme 1	4098.442

Rank	chr	start	end	Gene	Gene description	ERG signal
715	chr2	109937694	109969240	MIR4266	microRNA 4266	4097.8254
716	chr19	1245872	1267385	MIDN	midnolin	4096.0752
717	chr1	10436882	10478211	PGD	phosphogluconate dehydrogenase	4095.7039
718	chr5	88108952	88133571	MEF2C	myocyte enhancer factor 2C	4094.1397
719	chr1	94073030	94103055	BCAR3	breast cancer anti-estrogen resistance 3	4092.4075
720	chr5	141891092	141926663	FGF1	fibroblast growth factor 1	4090.665
721	chr17	79517761	79551904	NPLOC4	NPL4 homolog, ubiquitin recognition factor	4090.3314
722	chr3	171756088	171787202	FNDC3B	fibronectin type III domain containing 3B	4085.2682
723	chr22	36835024	36868015	TXN2	thioredoxin 2	4084.2858
724	chrX	64886529	64944161	MSN	moesin	4074.5824
725	chr10	104912295	104957958	NT5C2	5'-nucleotidase, cytosolic II	4073.1396
726	chr5	114866975	114896201	FEM1C	fem-1 homolog C	4071.1818
727	chr12	65045761	65077596	RASSF3	Ras association domain family member 3	4065.3295
728	chr5	146886123	146916919	DPYSL3	dihydropyrimidinase like 3	4065.072
729	chr22	22290513	22308177	PPM1F	protein phosphatase, Mg2+/Mn2+ dependent 1F	4062.72
730	chr15	42064681	42098432	MAPKBP1	mitogen-activated protein kinase binding protein 1	4060.2453
731	chr4	152116078	152150928	SH3D19	SH3 domain containing 19	4060.025
732	chr22	32020743	32059185	PISD	phosphatidylserine decarboxylase	4055.631
733	chr3	43783840	43812664	ABHD5	abhydrolase domain containing 5	4055.5368
734	chr9	21575308	21606596	MIR31HG	MIR31 host gene	4054.9248
735	chr10	5983368	6014691	IL15RA	interleukin 15 receptor subunit alpha	4046.9316
736	chr20	11135809	11167972	LOC339593	uncharacterized LOC339593	4042.8891
737	chr20	10330777	10358238	MKKS	McKusick-Kaufman syndrome	4042.2592
738	chr22	30804023	30832897	SEC14L2	SEC14 like lipid binding 2	4039.4726
739	chr3	98481181	98511397	ST3GAL6	ST3 beta-galactoside alpha-2,3-sialyltransferase 6	4030.8144
740	chr9	97757587	97789303	C9orf3	chromosome 9 open reading frame 3	4027.932
741	chr14	65695886	65726439	MIR4708	microRNA 4708	4026.8854
742	chr1	157962070	157991455	KIRREL	kin of IRRE like (Drosophila)	4022.8065
743	chr18	74797764	74828004	MBP	myelin basic protein	4021.92
744	chr9	80408187	80438391	GNAQ	G protein subunit alpha q	4014.1116
745	chr3	11535205	11565864	VGLL4	vestigial like family member 4	4010.1972
746	chr1	150530393	150553301	MCL1	BCL2 family apoptosis regulator	4006.6092
747	chr10	16985697	17012619	CUBN	cubilin	4003.3014
748	chr6	155491111	155524303	TIAM2	T-cell lymphoma invasion and metastasis 2	3999.636
749	chr1	90348223	90377003	LRRC8D	leucine rich repeat containing 8 family member D	3991.786
750	chr19	47404962	47441236	ARHGAP35	Rho GTPase activating protein 35	3990.14
751	chr2	182755045	182783085	SSFA2	sperm specific antigen 2	3990.092
752	chr6	74403674	74435543	CD109	CD109 molecule	3989.9988
753	chr15	71566007	71589810	THSD4	thrombospondin type 1 domain containing 4	3989.3828
754	chr15	40615254	40637429	C15orf52	chromosome 15 open reading frame 52	3989.2825
755	chr1	23491911	23526140	HTR1D	5-hydroxytryptamine receptor 1D	3987.6785
756	chr22	19860871	19882203	TXNRD2	thioredoxin reductase 2	3986.9508
757	chr16	4525756	4567407	CDIP1	cell death inducing p53 target 1	3981.8356
758	chr4	3107627	3137872	HTT	huntingtin	3980.242
759	chr12	118099654	118126726	KSR2	kinase suppressor of ras 2	3979.584

Rank	chr	start	end	Gene	Gene description	ERG signal
760	chr16	30061683	30090540	ALDOA	aldolase, fructose-bisphosphate A	3979.3803
761	chr2	46483641	46506352	EPAS1	endothelial PAS domain protein 1	3978.9672
762	chr2	65269737	65309736	CEP68	centrosomal protein 68	3975.9006
763	chr22	30673095	30702761	GATSL3	GATS protein like 3	3975.244
764	chr13	42162162	42190758	MIR5006	microRNA 5006	3974.844
765	chr10	4691236	4722168	LINC00704	long intergenic non-protein coding RNA 704	3974.762
766	chr14	85981997	86004008	FLRT2	fibronectin leucine rich transmembrane protein 2	3970.7844
767	chr13	106755353	106786538	LINC00460	long intergenic non-protein coding RNA 460	3966.732
768	chr17	38169818	38196043	SNORD124	small nucleolar RNA, C/D box 124	3962.5975
769	chr6	143993616	144023304	PHACTR2	phosphatase and actin regulator 2	3960.3792
770	chr7	92249712	92276507	FAM133B	family with sequence similarity 133 member B	3960.301
771	chr7	107851008	107889400	NRCAM	neuronal cell adhesion molecule	3958.2152
772	chr22	29998610	30032565	NF2	neurofibromin 2	3955.7575
773	chr6	117841464	117872581	DCBLD1	discoidin, CUB and LCCL domain containing 1	3954.9707
774	chr15	70981904	71009041	UACA	uveal autoantigen with coiled-coil domains and ankyrin repeats	3951.1472
775	chr10	51558757	51589676	NCOA4	nuclear receptor coactivator 4	3948.3563
776	chr1	86032463	86052272	DDAH1	dimethylarginine dimethylaminohydrolase 1	3947.9337
777	chr16	53105210	53135206	CHD9	chromodomain helicase DNA binding protein 9	3947.4736
778	chr1	27096980	27128304	PIGV	phosphatidylinositol glycan anchor biosynthesis class V	3943.6916
779	chr1	161154787	161177615	NDUFS2	NADH:ubiquinone oxidoreductase core subunit S2	3937.83
780	chr19	13946293	13965188	LOC284454	uncharacterized LOC284454	3935.8285
781	chr18	3579034	3608389	DLGAP1-AS1	DLGAP1 antisense RNA 1	3933.57
782	chr20	57450093	57484555	GNAS	GNAS complex locus	3932.1142
783	chr3	88079554	88111896	CGGBP1	CGG triplet repeat binding protein 1	3919.8504
784	chr19	3422827	3461405	SMIM24	small integral membrane protein 24	3919.5248
785	chr4	8184172	8208567	SH3TC1	SH3 domain and tetratricopeptide repeats 1	3917.837
786	chr22	47129753	47155769	CERK	ceramide kinase	3915.408
787	chr5	10701698	10735583	DAP	death associated protein	3910.329
788	chr3	27564660	27602521	SLC4A7	solute carrier family 4 member 7	3907.2552
789	chr3	158416921	158446032	RARRES1	retinoic acid receptor responder 1	3906.6962
790	chr5	169114563	169144838	DOCK2	dedicator of cytokinesis 2	3899.42
791	chr12	14517766	14554101	ATF7IP	activating transcription factor 7 interacting protein	3898.7455
792	chr1	59593010	59618271	HSD52	uncharacterized LOC729467	3895.2462
793	chr2	222364592	222396363	EPHA4	EPH receptor A4	3895.1246
794	chr6	85350345	85378560	TBX18	T-box 18	3890.8485
795	chr2	237980549	238008217	COPS8	COP9 signalosome subunit 8	3890.1208
796	chr10	106080794	106103813	ITPRIP	inositol 1,4,5-trisphosphate receptor interacting protein	3883.3053
797	chr6	56704883	56735166	DST	dystonin	3879.2523
798	chr14	50436723	50469162	LINC01588	long intergenic non-protein coding RNA 1588	3876.4605
799	chr1	15494451	15528508	TMEM51	transmembrane protein 51	3875.6866
800	chr7	50879731	50908227	GRB10	growth factor receptor bound protein 10	3872.6064
801	chr12	42958201	42985180	PRICKLE1	prickle planar cell polarity protein 1	3868.7886
802	chr8	11709392	11749639	CTSB	cathepsin B	3867.7367
803	chr4	140956939	140988716	MAML3	mastermind like transcriptional coactivator 3	3867.2609
804	chr4	75542683	75566825	BTC	betacellulin	3865.1342

Rank	chr	start	end	Gene	Gene description	ERG signal
805	chr16	15107268	15157004	NTAN1	N-terminal asparagine amidase	3864.4872
806	chr17	75176087	75208327	SEC14L1	SEC14 like lipid binding 1	3862.352
807	chr18	57540097	57572984	PMAIP1	phorbol-12-myristate-13-acetate-induced protein 1	3860.9338
808	chr2	113381328	113407385	FLJ42351	uncharacterized LOC400999	3859.0417
809	chr14	54634787	54661961	CDKN3	cyclin dependent kinase inhibitor 3	3858.708
810	chr10	92671251	92694036	ANKRD1	ankyrin repeat domain 1	3857.5005
811	chr14	52103387	52134150	FRMD6	FERM domain containing 6	3851.5276
812	chr12	94170210	94200126	CRADD	CASP2 and RIPK1 domain containing adaptor with death domain	3844.206
813	chr6	78155692	78177283	HTR1B	5-hydroxytryptamine receptor 1B	3843.198
814	chr9	22102108	22129706	CDKN2B-AS1	CDKN2B antisense RNA 1	3838.8818
815	chr1	32389209	32422735	PTP4A2	protein tyrosine phosphatase type IVA, member 2	3838.727
816	chr12	43660725	43687994	ADAMTS20	ADAM metallopeptidase with thrombospondin type 1 motif 20	3836.7483
817	chr2	54682061	54711422	SPTBN1	spectrin beta, non-erythrocytic 1	3831.6105
818	chr3	128266045	128288495	LINC01565	long intergenic non-protein coding RNA 1565	3829.97
819	chr11	86428643	86456548	ME3	malic enzyme 3	3828.566
820	chr20	30131771	30162419	MCTS2P	malignant T-cell amplified sequence 2, pseudogene	3827.9352
821	chr11	114028798	114052764	NNMT	nicotinamide N-methyltransferase	3827.3702
822	chr10	80980220	81007880	ZMIZ1	zinc finger MIZ-type containing 1	3819.846
823	chr16	20861191	20892292	LYRM1	LYR motif containing 1	3819.2028
824	chr1	8916762	8941071	ENO1-AS1	ENO1 antisense RNA 1	3818.9439
825	chr6	34190771	34217791	C6orf1	chromosome 6 open reading frame 1	3807.118
826	chr4	19713079	19742243	SLIT2	slit guidance ligand 2	3802.9856
827	chr3	11319953	11349694	ATG7	autophagy related 7	3800.8998
828	chr17	43369876	43399615	MAP3K14	mitogen-activated protein kinase kinase kinase 14	3800.6442
829	chr19	2031335	2062786	MKNK2	MAP kinase interacting serine/threonine kinase 2	3792.9906
830	chr8	119015570	119039511	EXT1	exostosin glycosyltransferase 1	3792.2544
831	chr1	100054478	100080355	PALMD	palmdelphin	3790.9805
832	chr4	77598179	77627422	SHROOM3	shroom family member 3	3789.8928
833	chr11	28759542	28790624	METTL15	methyltransferase like 15	3788.8958
834	chr10	75645774	75672136	PLAU	plasminogen activator, urokinase	3788.2194
835	chr5	66227448	66255812	MAST4	microtubule associated serine/threonine kinase family member 4	3786.594
836	chr5	52656263	52688321	FST	follistatin	3786.0498
837	chr3	193971756	193990785	LINC00887	long intergenic non-protein coding RNA 887	3784.8681
838	chr1	235046954	235070019	LINC01132	long intergenic non-protein coding RNA 1132	3782.66
839	chr21	39008230	39038555	DYRK1A	dual specificity tyrosine phosphorylation regulated kinase 1A	3781.5275
840	chr3	192586514	192620440	MB21D2	Mab-21 domain containing 2	3779.3564
841	chr12	124982325	125009826	NCOR2	nuclear receptor corepressor 2	3773.1372
842	chr14	69141185	69170884	ZFP36L1	ZFP36 ring finger protein like 1	3771.773
843	chr9	35744305	35762735	GBA2	glucosylceramidase beta 2	3770.778
844	chr11	86506185	86530611	PRSS23	protease, serine 23	3768.9318
845	chr15	39109282	39139297	C15orf53	chromosome 15 open reading frame 53	3766.8825
846	chr20	6643030	6666971	BMP2	bone morphogenetic protein 2	3765.9193
847	chr2	201625536	201655055	BZW1	basic leucine zipper and W2 domains 1	3760.7206
848	chr17	40558165	40589453	PTRF	polymerase I and transcript release factor	3757.6888
849	chr4	74706513	74739286	CXCL1	C-X-C motif chemokine ligand 1	3755.7858

Rank	chr	start	end	Gene	Gene description	ERG signal
850	chr7	102510132	102534051	LRRC17	leucine rich repeat containing 17	3755.283
851	chr8	38643554	38666290	TACC1	transforming acidic coiled-coil containing protein 1	3753.7136
852	chr12	95595948	95626385	VEZT	vezatin, adherens junctions transmembrane protein	3752.8821
853	chr7	135343335	135377890	C7orf73	chromosome 7 open reading frame 73	3749.2175
854	chr1	94262225	94284611	MIR760	microRNA 760	3738.462
855	chr16	27245202	27272126	FLJ21408	uncharacterized LOC400512	3737.0512
856	chr1	150112774	150136456	PLEKHO1	pleckstrin homology domain containing O1	3737.0196
857	chr7	151156192	151183746	CRYGN	crystallin gamma N	3736.3224
858	chr14	78046604	78079561	SPTLC2	serine palmitoyltransferase long chain base subunit 2	3734.0281
859	chr7	5700508	5734852	RNF216-IT1	RNF216 intronic transcript 1	3733.1928
860	chr16	80812382	80842653	CDYL2	chromodomain Y-like 2	3732.4143
861	chr8	40010065	40033670	C8orf4	chromosome 8 open reading frame 4	3731.9505
862	chr20	1783031	1807103	LOC100289473	cytoskeleton associated protein 2-like pseudogene	3728.7528
863	chr10	82252859	82272649	SH2D4B	SH2 domain containing 4B	3728.436
864	chr2	96906577	96932932	TMEM127	transmembrane protein 127	3726.597
865	chr12	123353922	123381501	VPS37B	VPS37B, ESCRT-I subunit	3720.4071
866	chr11	93860686	93886826	PANX1	pannexin 1	3717.108
867	chr17	46300657	46324790	MIR1203	microRNA 1203	3716.482
868	chr1	144007222	144016933	FAM72D	family with sequence similarity 72 member D	3716.3997
869	chr16	86596680	86625076	FOXL1	forkhead box L1	3714.1968
870	chr10	1088232	1111934	WDR37	WD repeat domain 37	3714.1034
871	chr19	3365037	3405601	NFIC	nuclear factor I C	3711.606
872	chr12	89605618	89633559	DUSP6	dual specificity phosphatase 6	3707.7707
873	chr1	147252559	147278057	GJA5	gap junction protein alpha 5	3707.4092
874	chr1	209995527	210018047	DIEXF	digestive organ expansion factor homolog (zebrafish)	3706.792
875	chr9	128282785	128314678	MAPKAP1	mitogen-activated protein kinase associated protein 1	3699.588
876	chr7	92380910	92412744	CDK6	cyclin dependent kinase 6	3699.1108
877	chr4	54925218	54952382	CHIC2	cysteine rich hydrophobic domain 2	3697.0204
878	chr8	96693029	96717216	LOC100500773	serine/arginine-rich splicing factor 3 pseudogene	3695.7736
879	chr12	32575129	32611779	FGD4	FYVE, RhoGEF and PH domain containing 4	3694.32
880	chr7	131303077	131328571	PODXL	podocalyxin like	3694.0806
881	chr5	157810644	157842911	CLINT1	clathrin interactor 1	3691.3448
882	chr1	214719175	214744821	PTPN14	protein tyrosine phosphatase, non-receptor type 14	3690.4594
883	chr6	76187853	76213930	FILIP1	filamin A interacting protein 1	3689.8955
884	chr1	60195679	60225680	MIR4711	microRNA 4711	3684.1228
885	chr8	118714062	118744801	MED30	mediator complex subunit 30	3682.5322
886	chr12	104669615	104701025	EID3	EP300 interacting inhibitor of differentiation 3	3681.252
887	chr3	5017629	5038735	BHLHE40-AS1	BHLHE40 antisense RNA 1	3680.8864
888	chr20	62669382	62689549	SOX18	SRY-box 18	3680.4775
889	chr9	134088669	134116278	NUP214	nucleoporin 214	3680.2797
890	chr2	47292421	47317486	C2orf61	chromosome 2 open reading frame 61	3679.542
891	chr2	54166105	54199467	PSME4	proteasome activator subunit 4	3676.4924
892	chr4	170057411	170082588	SH3RF1	SH3 domain containing ring finger 1	3675.842
893	chr1	156627130	156648296	NES	nestin	3674.4176
894	chr10	43888967	43917255	HNRNPF	heterogeneous nuclear ribonucleoprotein F	3671.7824

Rank	chr	start	end	Gene	Gene description	ERG signal
895	chr5	146845277	146868039	DPYSL3	dihydropyrimidinase like 3	3671.5106
896	chr7	107706525	107737061	LAMB4	laminin subunit beta 4	3670.4272
897	chr8	124472826	124503350	FBXO32	F-box protein 32	3668.9848
898	chr1	12654570	12680436	DHRS3	dehydrogenase/reductase 3	3667.7988
899	chr5	136804868	136835194	SPOCK1	SPARC/osteonectin, cwcv and kazal like domains proteoglycan 1	3666.4134
900	chr19	47351252	47382795	AP2S1	adaptor related protein complex 2 sigma 1 subunit	3665.2966
901	chr1	198835070	198864526	MIR181A1	microRNA 181a-1	3664.3264
902	chr4	185303127	185331948	LOC728175	uncharacterized LOC728175	3663.1491
903	chr2	11513976	11545294	LINC00570	long intergenic non-protein coding RNA 570	3657.9424
904	chr8	56742841	56780859	LYN	LYN proto-oncogene, Src family tyrosine kinase	3649.728
905	chr10	45469638	45493321	C10orf10	chromosome 10 open reading frame 10	3647.182
906	chr12	53988319	54021000	ATF7	activating transcription factor 7	3643.9315
907	chr10	93349958	93374525	HECTD2-AS1	HECTD2 antisense RNA 1	3643.2861
908	chr15	64124559	64153381	HERC1	HECT and RLD domain containing E3 ubiquitin protein ligase family member 1	3643.1008
909	chr14	69542946	69569128	DCAF5	DDB1 and CUL4 associated factor 5	3641.9162
910	chr11	118779259	118801210	MIR4492	microRNA 4492	3639.4758
911	chr6	16711125	16739375	ATXN1	ataxin 1	3638.6
912	chr3	188247290	188277788	LPP	LIM domain containing preferred translocation partner in lipoma	3635.3616
913	chr20	48635583	48665653	TRERNA1	translation regulatory long non-coding RNA 1	3632.456
914	chr2	188389632	188420155	TFPI	tissue factor pathway inhibitor	3632.237
915	chr5	148512830	148533267	ABLIM3	actin binding LIM protein family member 3	3629.6112
916	chr5	71658153	71685956	PTCD2	pentatricopeptide repeat domain 2	3628.2915
917	chr3	172022810	172051269	GHSR	growth hormone secretagogue receptor	3625.6766
918	chr7	115849832	115873624	TES	testin LIM domain protein	3618.7632
919	chr5	16983006	17022508	MYO10	myosin X	3618.3832
920	chr20	33889707	33916248	UQCC1	ubiquinol-cytochrome c reductase complex assembly factor 1	3617.5383
921	chr4	77921623	77945294	Sep-11	septin 11	3616.9288
922	chr16	28191726	28225619	XPO6	exportin 6	3616.3831
923	chr8	103799507	103824070	AZIN1	antizyme inhibitor 1	3615.6736
924	chr4	186628706	186660531	SORBS2	sorbin and SH3 domain containing 2	3615.32
925	chr16	87085550	87111540	C16orf95	chromosome 16 open reading frame 95	3615.209
926	chr4	56025571	56054076	KDR	kinase insert domain receptor	3611.5835
927	chr12	13348076	13366140	EMP1	epithelial membrane protein 1	3610.9936
928	chr1	42305877	42337192	HIVEP3	human immunodeficiency virus type I enhancer binding protein 3	3610.6195
929	chr12	113659125	113685746	TPCN1	two pore segment channel 1	3609.8076
930	chr5	59039102	59066226	PDE4D	phosphodiesterase 4D	3607.492
931	chr5	126323048	126351691	Mar-03	membrane associated ring-CH-type finger 3	3606.1537
932	chr20	43964807	43990111	SDC4	syndecan 4	3598.2288
933	chr3	43219474	43241913	POMGNT2	protein O-linked mannose N-acetylglucosaminyltransferase 2 (beta 1,4-)	3594.7278
934	chr2	43384880	43407124	ZFP36L2	ZFP36 ring finger protein like 2	3592.406
935	chr6	157448972	157474230	ARID1B	AT-rich interaction domain 1B	3586.636
936	chr4	138955413	138985047	SLC7A11-AS1	SLC7A11 antisense RNA 1	3579.7872
937	chr22	24684418	24722972	SPECC1L	sperm antigen with calponin homology and coiled-coil domains 1 like	3577.8112
938	chr1	155929483	155954467	ARHGEF2	Rho/Rac guanine nucleotide exchange factor 2	3575.2104
939	chr3	124550789	124580630	ITGB5	integrin subunit beta 5	3574.9518

Rank	chr	start	end	Gene	Gene description	ERG signal
940	chr15	95827713	95849368	LINC01197	long intergenic non-protein coding RNA 1197	3573.075
941	chr6	136903582	136932602	MAP7	microtubule associated protein 7	3572.362
942	chr18	20684999	20718256	CABLES1	Cdk5 and Abl enzyme substrate 1	3571.8018
943	chr4	119672248	119697215	SEC24D	SEC24 homolog D, COPII coat complex component	3570.281
944	chr3	134079996	134094933	AMOTL2	angiomotin like 2	3566.9556
945	chr8	17458742	17478982	PDGFRL	platelet derived growth factor receptor like	3564.264
946	chr5	100162859	100191484	ST8SIA4	ST8 alpha-N-acetyl-neuraminide alpha-2,8-sialyltransferase 4	3555.225
947	chr3	29552841	29580793	RBMS3	RNA binding motif single stranded interacting protein 3	3552.6992
948	chr19	3140172	3163540	GNA15	G protein subunit alpha 15	3551.936
949	chr20	35458587	35487733	SOGA1	suppressor of glucose, autophagy associated 1	3549.9828
950	chr7	132093819	132118369	PLXNA4	plexin A4	3549.93
951	chr17	75100867	75126400	SEC14L1	SEC14 like lipid binding 1	3549.087
952	chr2	188162922	188186447	CALCRL	calcitonin receptor like receptor	3547.57
953	chr19	6085543	6111915	RFX2	regulatory factor X2	3544.3968
954	chr9	130522282	130534586	SH2D3C	SH2 domain containing 3C	3543.552
955	chr11	57172478	57195439	SLC43A3	solute carrier family 43 member 3	3542.8823
956	chr3	98680214	98706191	DCBLD2	discoidin, CUB and LCCL domain containing 2	3540.6651
957	chr5	38782270	38808683	OSMR	oncostatin M receptor	3539.342
958	chr20	32374212	32403291	CHMP4B	charged multivesicular body protein 4B	3538.9143
959	chr1	61408245	61430322	NFIA	nuclear factor I A	3536.7354
960	chr8	81876227	81904587	ZNF704	zinc finger protein 704	3536.492
961	chr10	102105079	102135379	SCD	stearoyl-CoA desaturase	3536.01
962	chr1	85904589	85932549	DDAH1	dimethylarginine dimethylaminohydrolase 1	3534.144
963	chr2	165625970	165652340	COBLL1	cordon-bleu WH2 repeat protein like 1	3533.58
964	chr9	137282867	137308168	RXRA	retinoid X receptor alpha	3532.0196
965	chr15	75320325	75341381	PPCDC	phosphopantothenoylcysteine decarboxylase	3531.0912
966	chr6	86158635	86176475	NT5E	5'-nucleotidase ecto	3530.536
967	chr18	33184673	33216739	MIR3975	microRNA 3975	3530.4666
968	chr4	122606266	122634027	ANXA5	annexin A5	3528.4231
969	chr13	33758977	33783032	STARD13	StAR related lipid transfer domain containing 13	3521.652
970	chr6	144451607	144481489	STX11	syntaxin 11	3520.0996
971	chr11	86213242	86237169	CCDC81	coiled-coil domain containing 81	3517.269
972	chr20	39764833	39782062	PLCG1	phospholipase C gamma 1	3516.4389
973	chr7	129535604	129566072	UBE2H	ubiquitin conjugating enzyme E2 H	3516.0072
974	chrX	13096405	13143786	FAM9C	family with sequence similarity 9 member C	3515.6702
975	chr5	137069452	137091471	HNRNPA0	heterogeneous nuclear ribonucleoprotein A0	3512.0305
976	chr17	36603015	36630028	ARHGAP23	Rho GTPase activating protein 23	3511.69
977	chr4	90390316	90420776	GPRIN3	GPRIN family member 3	3508.992
978	chr3	66477198	66502028	LRIG1	leucine rich repeats and immunoglobulin like domains 1	3503.513
979	chr19	16544844	16570498	EPS15L1	epidermal growth factor receptor pathway substrate 15 like 1	3499.2056
980	chr5	64480351	64507179	ADAMTS6	ADAM metallopeptidase with thrombospondin type 1 motif 6	3498.3712
981	chr9	136795981	136823057	VAV2	vav guanine nucleotide exchange factor 2	3498.2192
982	chr19	780709	803768	PTBP1	polypyrimidine tract binding protein 1	3498.0503
983	chr10	30071311	30097361	SVIL	supervillin	3495.91
984	chr7	116758147	116784644	ST7	suppression of tumorigenicity 7	3494.9543

Rank	chr	start	end	Gene	Gene description	ERG signal
985	chr14	38859962	38889501	CLEC14A	C-type lectin domain family 14 member A	3494.4637
986	chr13	31365490	31393265	LINC00398	long intergenic non-protein coding RNA 398	3494.095
987	chr10	29420231	29450042	LYZL1	lysozyme like 1	3493.8492
988	chr1	54777887	54804840	SSBP3	single stranded DNA binding protein 3	3493.1088
989	chr20	47274365	47302350	PREX1	phosphatidylinositol-3,4,5-trisphosphate dependent Rac exchange factor 1	3492.528
990	chr5	136676513	136699668	SPOCK1	SPARC/osteonectin, cwcv and kazal like domains proteoglycan 1	3491.774
991	chr5	71528761	71553591	MRPS27	mitochondrial ribosomal protein S27	3491.098
992	chr17	41593448	41624784	ETV4	ETS variant 4	3490.8304
993	chr12	110460168	110494609	C12orf76	chromosome 12 open reading frame 76	3488.8733
994	chr5	54275388	54303198	ESM1	endothelial cell specific molecule 1	3487.374
995	chr10	35656896	35677623	CCNY	cyclin Y	3486.2814
996	chr6	33736176	33758026	LEMD2	LEM domain containing 2	3485.075
997	chr3	194586847	194606667	LOC100507391	uncharacterized LOC100507391	3484.356
998	chr14	24886739	24909521	KHNYN	KH and NYN domain containing	3483.3678
999	chr11	12694692	12716983	TEAD1	TEA domain transcription factor 1	3481.8542
1000	chr22	39913923	39929399	ATF4	activating transcription factor 4	3477.4572
1001	chr11	13460502	13485922	BTBD10	BTB domain containing 10	3477.456
1002	chr5	156966985	156992403	ADAM19	ADAM metallopeptidase domain 19	3477.1824
1003	chr2	128925325	128955204	UGGT1	UDP-glucose glycoprotein glucosyltransferase 1	3474.9277
1004	chr1	61899304	61921960	NFIA	nuclear factor I A	3470.8992
1005	chr7	134828229	134853113	TMEM140	transmembrane protein 140	3468.8296
1006	chr8	51527943	51557066	SNTG1	syntrophin gamma 1	3468.5493
1007	chr2	109780045	109803355	MIR4265	microRNA 4265	3468.528
1008	chr18	9091865	9120666	NDUFV2	NADH:ubiquinone oxidoreductase core subunit V2	3467.6404
1009	chr12	88691458	88720316	TMTC3	transmembrane and tetratricopeptide repeat containing 3	3462.96
1010	chr8	79426995	79451666	PKIA	protein kinase (cAMP-dependent, catalytic) inhibitor alpha	3461.3413
1011	chr2	55309877	55340679	RTN4	reticulon 4	3459.0646
1012	chr13	33800889	33827658	STARD13	StAR related lipid transfer domain containing 13	3458.5548
1013	chr4	139094875	139122358	SLC7A11	solute carrier family 7 member 11	3457.3614
1014	chr2	164508769	164540067	FIGN	fidgetin, microtubule severing factor	3455.2992
1015	chr13	49660381	49689242	FNDC3A	fibronectin type III domain containing 3A	3454.6617
1016	chr9	124111133	124133380	STOM	stomatin	3446.0603
1017	chr8	26297287	26324434	PNMA2	paraneoplastic Ma antigen 2	3444.9543
1018	chr5	159875574	159908116	MIR3142	microRNA 3142	3442.9436
1019	chr12	69632435	69661052	CPSF6	cleavage and polyadenylation specific factor 6	3439.7634
1020	chr14	31730908	31766083	HECTD1	HECT domain E3 ubiquitin protein ligase 1	3436.5975
1021	chr19	8404902	8433753	KANK3	KN motif and ankyrin repeat domains 3	3436.1541
1022	chr4	186304538	186335408	ANKRD37	ankyrin repeat domain 37	3435.831
1023	chr4	177689712	177716827	VEGFC	vascular endothelial growth factor C	3435.4705
1024	chr2	20226531	20253281	LAPTM4A	lysosomal protein transmembrane 4 alpha	3434.7
1025	chr3	156873798	156895065	CCNL1	cyclin L1	3434.6205
1026	chr10	62715025	62740785	RHOBTB1	Rho related BTB domain containing 1	3433.808
1027	chr11	35638917	35664297	FJX1	four jointed box 1	3431.376
1028	chr9	33150411	33173466	B4GALT1	beta-1,4-galactosyltransferase 1	3430.584
1029	chr16	4348210	4380078	GLIS2	GLIS family zinc finger 2	3428.9968

Rank	chr	start	end	Gene	Gene description	ERG signal
1030	chr5	55623288	55649409	ANKRD55	ankyrin repeat domain 55	3427.0752
1031	chr6	90006558	90034851	GABRR2	gamma-aminobutyric acid type A receptor rho2 subunit	3426.2823
1032	chr19	12887958	12905706	JUNB	JunB proto-oncogene, AP-1 transcription factor subunit	3425.364
1033	chr10	7989009	8015326	GATA3-AS1	GATA3 antisense RNA 1	3423.8417
1034	chr10	75813727	75842280	VCL	vinculin	3420.6494
1035	chr1	90308100	90333234	LRRC8D	leucine rich repeat containing 8 family member D	3418.224
1036	chr5	149378863	149404335	HMGXB3	HMG-box containing 3	3415.7952
1037	chr12	26780973	26805683	ITPR2	inositol 1,4,5-trisphosphate receptor type 2	3414.922
1038	chr11	126204366	126226969	ST3GAL4-AS1	ST3GAL4 antisense RNA 1 (head to head)	3413.053
1039	chr17	1456914	1494743	PITPNA	phosphatidylinositol transfer protein alpha	3412.1758
1040	chr2	237778562	237799406	COPS8	COP9 signalosome subunit 8	3412.1628
1041	chr2	68457186	68482115	PPP3R1	protein phosphatase 3 regulatory subunit B, alpha	3410.2872
1042	chr17	26496707	26524176	PYY2	peptide YY 2 (pseudogene)	3408.9029
1043	chr1	33398972	33431311	RNF19B	ring finger protein 19B	3408.5306
1044	chrX	99896826	99941255	SRPX2	sushi repeat containing protein, X-linked 2	3407.7043
1045	chr19	47676285	47715750	MIR3190	microRNA 3190	3405.8295
1046	chr11	59539067	59570619	STX3	syntaxin 3	3401.3056
1047	chr8	8923227	8947797	MIR4660	microRNA 4660	3398.031
1048	chr21	43629163	43655827	ABCG1	ATP binding cassette subfamily G member 1	3396.9936
1049	chr1	8746293	8775118	RERE	arginine-glutamic acid dipeptide repeats	3392.7025
1050	chr2	224782653	224810818	WDFY1	WD repeat and FYVE domain containing 1	3385.433
1051	chr1	9351473	9380121	SPSB1	splA/ryanodine receptor domain and SOCS box containing 1	3383.3288
1052	chr13	27743023	27770808	USP12	ubiquitin specific peptidase 12	3381.4345
1053	chr13	77281013	77305650	KCTD12	potassium channel tetramerization domain containing 12	3380.1964
1054	chr8	128217115	128238957	POU5F1B	POU class 5 homeobox 1B	3378.9574
1055	chr5	74952100	74977313	POC5	POC5 centriolar protein	3376.0207
1056	chr14	100824738	100853577	WARS	tryptophanyl-tRNA synthetase	3374.163
1057	chr20	10520306	10548008	JAG1	jagged 1	3374.1036
1058	chr14	85762347	85787554	LINC00911	long intergenic non-protein coding RNA 911	3370.1759
1059	chr13	107142280	107166309	EFNB2	ephrin B2	3368.8658
1060	chr20	17634951	17663857	RRBP1	ribosome binding protein 1	3367.549
1061	chr22	28261235	28285922	PITPNB	phosphatidylinositol transfer protein beta	3364.8381
1062	chr1	15654527	15680271	FHAD1	forkhead associated phosphopeptide binding domain 1	3364.7408
1063	chr7	141349034	141377140	KIAA1147	KIAA1147	3364.2882
1064	chr9	71784333	71808379	TJP2	tight junction protein 2	3364.0354
1065	chr10	122390538	122411494	PLPP4	phospholipid phosphatase 4	3363.438
1066	chr5	172355370	172383777	ERGIC1	endoplasmic reticulum-golgi intermediate compartment 1	3363.3888
1067	chr12	121623957	121654020	P2RX4	purinergic receptor P2X 4	3361.0434
1068	chr10	31984188	31999298	ARHGAP12	Rho GTPase activating protein 12	3360.464
1069	chr16	27323910	27348285	IL4R	interleukin 4 receptor	3358.875
1070	chr10	126823914	126851067	CTBP2	C-terminal binding protein 2	3358.8261
1071	chr1	181057325	181076345	IER5	immediate early response 5	3355.128
1072	chr3	105069904	105089954	ALCAM	activated leukocyte cell adhesion molecule	3352.36
1073	chr17	74683803	74713310	MXRA7	matrix remodeling associated 7	3351.9952
1074	chr12	12866720	12890992	CDKN1B	cyclin dependent kinase inhibitor 1B	3351.9632

Rank	chr	start	end	Gene	Gene description	ERG signal
1075	chr6	3735967	3754926	PXDC1	PX domain containing 1	3351.9512
1076	chr11	9572879	9601106	WEE1	WEE1 G2 checkpoint kinase	3350.5449
1077	chr3	156391248	156413231	TIPARP-AS1	TIPARP antisense RNA 1	3350.2092
1078	chr3	177060668	177079906	LINC00578	long intergenic non-protein coding RNA 578	3347.412
1079	chr10	65458640	65483371	REEP3	receptor accessory protein 3	3346.1043
1080	chr2	25490234	25519251	DNMT3A	DNA methyltransferase 3 alpha	3345.6601
1081	chr10	23104426	23139014	ARMC3	armadillo repeat containing 3	3344.6596
1082	chr3	50353720	50363956	HYAL2	hyaluronoglucosaminidase 2	3342.054
1083	chr2	191833915	191850641	STAT1	signal transducer and activator of transcription 1	3341.8548
1084	chr1	186444099	186469686	MIR548F1	microRNA 548f-1	3336.5448
1085	chr11	74464196	74496144	RNF169	ring finger protein 169	3332.1764
1086	chr14	77412839	77429282	IRF2BPL	interferon regulatory factor 2 binding protein like	3328.0632
1087	chr2	71717691	71733523	DYSF	dysferlin	3327.8864
1088	chr17	40153902	40178140	NKIRAS2	NFKB inhibitor interacting Ras like 2	3325.4536
1089	chr10	127729274	127752801	FANK1	fibronectin type III and ankyrin repeat domains 1	3324.3651
1090	chr6	30707093	30723131	FLOT1	flotillin 1	3321.4698
1091	chr7	106228810	106251022	CCDC71L	coiled-coil domain containing 71-like	3320.694
1092	chr2	39694425	39723372	LOC728730	uncharacterized LOC728730	3320.2209
1093	chr3	64892876	64915477	MIR548A2	microRNA 548a-2	3320.0869
1094	chr12	49882261	49909831	KCNH3	potassium voltage-gated channel subfamily H member 3	3319.428
1095	chr1	208019451	208043291	CD34	CD34 molecule	3318.528
1096	chr1	2120263	2146963	FAAP20	Fanconi anemia core complex associated protein 20	3316.14
1097	chr11	111793244	111818713	DIXDC1	DIX domain containing 1	3316.0638
1098	chr3	124760958	124783099	HEG1	heart development protein with EGF like domains 1	3314.5077
1099	chr8	97323523	97348177	PTDSS1	phosphatidylserine synthase 1	3311.0322
1100	chr22	37940992	37962201	CDC42EP1	CDC42 effector protein 1	3310.7249
1101	chr8	90893536	90918244	OSGIN2	oxidative stress induced growth inhibitor family member 2	3308.4012
1102	chr7	150685431	150706437	NOS3	nitric oxide synthase 3	3306.3444
1103	chr14	61927232	61947049	PRKCH	protein kinase C eta	3305.4756
1104	chr5	95046527	95074872	RHOBTB3	Rho related BTB domain containing 3	3305.027
1105	chr20	46906636	46928808	LINC00494	long intergenic non-protein coding RNA 494	3303.628
1106	chr3	14844486	14866635	FGD5	FYVE, RhoGEF and PH domain containing 5	3302.4159
1107	chr20	52194996	52241227	ZNF217	zinc finger protein 217	3296.2703
1108	chr2	192476276	192504159	NABP1	nucleic acid binding protein 1	3295.7706
1109	chr6	2763990	2785687	WRNIP1	Werner helicase interacting protein 1	3293.6046
1110	chr2	225263189	225283614	FAM124B	family with sequence similarity 124 member B	3292.51
1111	chr15	83760418	83783841	TM6SF1	transmembrane 6 superfamily member 1	3288.5892
1112	chr8	82257275	82280287	FABP5	fatty acid binding protein 5	3283.8124
1113	chr9	100768508	100800450	NANS	N-acetylneuraminate synthase	3283.6376
1114	chr11	65653947	65671575	CCDC85B	coiled-coil domain containing 85B	3280.5708
1115	chr11	128198005	128214420	ETS1	ETS proto-oncogene 1, transcription factor	3278.0755
1116	chr1	183837877	183859119	COLGALT2	collagen beta(1-O)galactosyltransferase 2	3277.6406
1117	chr17	68029134	68050595	KCNJ16	potassium voltage-gated channel subfamily J member 16	3274.9486
1118	chr4	114878565	114902407	ARSJ	arylsulfatase family member J	3273.5066
1119	chr11	6654844	6669965	DCHS1	dachsous cadherin-related 1	3272.1844

Rank	chr	start	end	Gene	Gene description	ERG signal
1120	chr8	29511012	29533901	LINC00589	long intergenic non-protein coding RNA 589	3268.5492
1121	chr12	76413064	76434074	PHLDA1	pleckstrin homology like domain family A member 1	3267.055
1122	chr22	20861096	20888482	MED15	mediator complex subunit 15	3264.4112
1123	chr2	143703574	143733599	KYNU	kynureninase	3263.7175
1124	chr5	171863221	171883769	SH3PXD2B	SH3 and PX domains 2B	3263.0224
1125	chr13	74688190	74711273	KLF12	Kruppel like factor 12	3259.3196

Online Table VII: Super-enhancers identified by enrichment of H3K27ac in VCaP. Ordered by super-enhancer ranking. Chromosome (chr), start and end indicates genomic position on the GRCh37/hg19 assembly. Super-enhancer-associated gene symbols and description indicated. H3K27ac signal is ChIP-seq read density times length of stitched enhancer. This table spans 5 pages.

Rank	chr	start	end	Gene	Gene description	H3K27ac signal
1	chr11	63886137	63955680	STIP1	stress induced phosphoprotein 1	88952.4513
2	chr11	65243451	65292297	MIR548AR	microRNA 548ar	61873.2282
3	chr13	111175330	111216273	RAB20	RAB20, member RAS oncogene family	61213.8793
4	chr11	64032734	64043730	GPR137	G protein-coupled receptor 137	43743.1876
5	chr11	65182177	65198196	NEAT1	nuclear paraspeckle assembly transcript 1 (non-protein coding)	40295.7945
6	chr11	63752577	63777355	OTUB1	OTU deubiquitinase, ubiquitin aldehyde binding 1	35687.7534
7	chr11	65623144	65630267	CFL1	cofilin 1	34887.7417
8	chr11	67034111	67081494	ANKRD13D	ankyrin repeat domain 13D	33495.0427
9	chr1	154903961	154914522	PMVK	phosphomevalonate kinase	32909.1321
10	chr21	42859797	42909078	TMPRSS2	transmembrane protease, serine 2	31529.9838
11	chr17	48980779	49033228	TOB1	transducer of ERBB2, 1	30572.5221
12	chr11	63989728	64001958	DNAJC4	DnaJ heat shock protein family (Hsp40) member C4	29246.822
13	chr11	63847857	63863960	FLRT1	fibronectin leucine rich transmembrane protein 1	28555.4499
14	chr5	180608625	180634929	TRIM7	tripartite motif containing 7	27479.7888
15	chr1	154926182	154936882	PBXIP1	PBX homeobox interacting protein 1	27221.87
16	chr13	111362848	111370472	ING1	inhibitor of growth family member 1	26398.1
17	chr11	64141627	64154286	MIR1237	microRNA 1237	25869.9324
18	chr15	93179478	93211274	FAM174B	family with sequence similarity 174 member B	24924.8844
19	chr13	111561525	111574528	ANKRD10	ankyrin repeat domain 10	24647.1865
20	chr13	111157318	111161605	COL4A2-AS1	COL4A2 antisense RNA 1	24486.0579
21	chr13	110427487	110441741	IRS2	insulin receptor substrate 2	23866.8976
22	chr20	32231665	32256811	C20orf144	chromosome 20 open reading frame 144	23446.1304
23	chr5	176871148	176884814	PRR7-AS1	PRR7 antisense RNA 1	23222.6338
24	chr11	66048281	66060928	YIF1A	Yip1 interacting factor homolog A, membrane trafficking protein	23154.1276
25	chr11	64898317	64905391	SYVN1	synoviolin 1	22991.9148
26	chr11	64079669	64088864	ESRRA	estrogen related receptor alpha	22124.0895
27	chr11	64122270	64130535	RPS6KA4	ribosomal protein S6 kinase A4	21312.129
28	chr4	1144586	1166502	SPON2	spondin 2	21173.0476
29	chr6	33681530	33716116	IP6K3	inositol hexakisphosphate kinase 3	20803.479
30	chr13	111266463	111274529	NAXD	NAD(P)HX dehydratase	20663.4788
31	chr12	52425902	52448137	NR4A1	nuclear receptor subfamily 4 group A member 1	19780.256
32	chr1	154972106	154979732	ZBTB7B	zinc finger and BTB domain containing 7B	19294.5426
33	chr1	226297238	226323776	H3F3AP4	H3 histone, family 3A, pseudogene 4	19285.1646
34	chr1	154944830	154951020	MIR4258	microRNA 4258	19236.663
35	chr6	33941828	33960377	MIR1275	microRNA 1275	17710.5852
36	chr11	65413041	65421766	MIR4489	microRNA 4489	17572.15
37	chr17	80052835	80073000	FASN	fatty acid synthase	17307.6195
38	chr8	128101730	128117517	PCAT1	prostate cancer associated transcript 1 (non-protein coding)	17177.8347
39	chr11	64876509	64887045	TM7SF2	transmembrane 7 superfamily member 2	17093.6064
40	chr14	38051914	38069502	FOXA1	forkhead box A1	16606.5896

Rank	chr	start	end	Gene	Gene description	H3K27ac signal
41	chr5	176816534	176832900	PFN3	profilin 3	16577.1214
42	chr13	111802997	111807699	ARHGEF7	Rho guanine nucleotide exchange factor 7	16563.2652
43	chr17	46795513	46807902	MIR3185	microRNA 3185	16429.0529
44	chr11	65649873	65660445	CCDC85B	coiled-coil domain containing 85B	16245.9924
45	chr7	38343920	38360160	TARP	TCR gamma alternate reading frame protein	16147.432
46	chr13	110220166	110231591	IRS2	insulin receptor substrate 2	16137.8125
47	chr21	46931277	46943782	COL18A1	collagen type XVIII alpha 1 chain	16063.923
48	chr21	38069525	38082854	SIM2	single-minded family bHLH transcription factor 2	15880.1706
49	chr17	79476469	79487946	ACTG1	actin gamma 1	15857.7709
50	chr9	139682680	139699797	CCDC183	coiled-coil domain containing 183	15680.8837
51	chr19	13101378	13129125	NFIX	nuclear factor I X	15616.0116
52	chr11	67904094	67916826	CHKA	choline kinase alpha	15350.9724
53	chr21	45341791	45351179	AGPAT3	1-acylglycerol-3-phosphate O-acyltransferase 3	15185.09
54	chr11	67570469	67576651	FAM86C2P	family with sequence similarity 86, member A pseudogene	15184.2284
55	chr11	65335272	65345853	SSSCA1	Sjogren syndrome/scleroderma autoantigen 1	15119.1909
56	chr11	63635545	63646518	MARK2	microtubule affinity regulating kinase 2	15109.821
57	chr11	67395314	67402864	NUDT8	nudix hydrolase 8	15014.685
58	chrX	40004652	40036241	BCOR	BCL6 corepressor	14919.4847
59	chr11	64071181	64077111	ESRRA	estrogen related receptor alpha	14747.91
60	chr19	18598403	18619025	ELL	elongation factor for RNA polymerase II	14464.2708
61	chr5	137798958	137803788	EGR1	early growth response 1	14419.965
62	chr6	34502957	34521152	SPDEF	SAM pointed domain containing ETS transcription factor	14363.133
63	chr12	125203439	125232496	SCARB1	scavenger receptor class B member 1	14354.158
64	chr11	66111597	66116178	B4GAT1	beta-1,4-glucuronyltransferase 1	14350.8987
65	chr15	90289537	90302901	MESP1	mesoderm posterior bHLH transcription factor 1	14213.9504
66	chr11	63603861	63609683	MARK2	microtubule affinity regulating kinase 2	14128.8296
67	chr11	118786625	118801815	BCL9L	B-cell CLL/lymphoma 9-like	13999.104
68	chr9	137247903	137271599	MIR4669	microRNA 4669	13994.8576
69	chr17	79338162	79367830	LOC100130370	uncharacterized LOC100130370	13979.5616
70	chr7	5458019	5470292	TNRC18	trinucleotide repeat containing 18	13948.2645
71	chr17	80245453	80257151	CSNK1D	casein kinase 1 delta	13865.6394
72	chr8	135731453	135767533	ZFAT	zinc finger and AT-hook domain containing	13706.792
73	chr17	79677305	79695633	SLC25A10	solute carrier family 25 member 10	13485.7424
74	chr17	81008290	81023614	B3GNTL1	UDP-GlcNAc:betaGal beta-1,3-N-acetylglucosaminyltransferase-like 1	13475.9256
75	chr11	67118382	67126123	POLD4	DNA polymerase delta 4, accessory subunit	13470.1141
76	chr11	66382346	66387011	RBM14-RBM4	RBM14-RBM4 readthrough	13395.5475
77	chr7	73239144	73247376	CLDN4	claudin 4	13257.636
78	chr1	19392005	19414870	UBR4	ubiquitin protein ligase E3 component n-recognin 4	13245.6945
79	chr11	67269436	67277338	CDK2AP2	cyclin dependent kinase 2 associated protein 2	13230.3186
80	chr11	67977724	67983835	KMT5B	lysine methyltransferase 5B	13111.1505
81	chr9	132243912	132259501	LINC00963	long intergenic non-protein coding RNA 963	13076.0532
82	chr21	44742178	44766101	SIK1	salt inducible kinase 1	13061.958
83	chr11	65379325	65385629	PCNX3	pecanex homolog 3 (Drosophila)	13047.3888
84	chr1	17221786	17241735	CROCC	ciliary rootlet coiled-coil, rootletin	12938.9214
85	chr11	64609021	64617015	CDC42BPG	CDC42 binding protein kinase gamma	12852.7532

Rank	chr	start	end	Gene	Gene description	H3K27ac signal
86	chr19	2031841	2062449	MKNK2	MAP kinase interacting serine/threonine kinase 2	12711.5024
87	chr11	64642798	64647659	EHD1	EH domain containing 1	12660.9606
88	chr11	67230953	67237459	TMEM134	transmembrane protein 134	12628.7966
89	chr19	56145447	56156388	ZNF580	zinc finger protein 580	12602.9379
90	chr17	79004299	79032075	BAIAP2	BAI1 associated protein 2	12593.6384
91	chr19	18526930	18544408	SSBP4	single stranded DNA binding protein 4	12538.7172
92	chr12	52509288	52522382	ATG101	autophagy related 101	12529.6486
93	chr19	52528469	52532480	ZNF614	zinc finger protein 614	12495.4683
94	chr8	38613601	38628034	TACC1	transforming acidic coiled-coil containing protein 1	12478.7718
95	chr17	27913332	27923471	ANKRD13B	ankyrin repeat domain 13B	12443.5947
96	chr17_gl000205_random	83881	88749	MGC70870	C-terminal binding protein 2 pseudogene	12360.3388
97	chr21	46284204	46295357	PTTG1IP	pituitary tumor-transforming 1 interacting protein	12254.9164
98	chr11	63794380	63807552	OTUB1	OTU deubiquitinase, ubiquitin aldehyde binding 1	12251.2772
99	chr21	38337429	38353707	HLCS	holocarboxylase synthetase	12174.3162
100	chr11	64050410	64056688	GPR137	G protein-coupled receptor 137	12166.1362
101	chr5	180668454	180676365	RACK1	receptor for activated C kinase 1	11940.8634
102	chr6	34110778	34123879	GRM4	glutamate metabotropic receptor 4	11903.5686
103	chr21	37859448	37883781	CLDN14	claudin 14	11867.2041
104	chr17	77773586	77789678	CBX8	chromobox 8	11803.482
105	chr6	35691347	35701262	FKBP5	FK506 binding protein 5	11793.8925
106	chr11	64779686	64784641	ARL2-SNX15	ARL2-SNX15 readthrough (NMD candidate)	11788.4405
107	chr11	65147109	65153282	SLC25A45	solute carrier family 25 member 45	11786.7262
108	chr11	125983241	125987236	CDON	cell adhesion associated, oncogene regulated	11766.4735
109	chr5	180233032	180239325	MGAT1	mannosyl (alpha-1,3-)-glycoprotein beta-1,2-N-acetylglucosaminyltransferase	11750.2896
110	chr5	179708633	179730172	MAPK9	mitogen-activated protein kinase 9	11654.7529
111	chr6	21586728	21596482	SOX4	SRY-box 4	11619.9402
112	chr17	27050966	27056686	TLCD1	TLC domain containing 1	11616.748
113	chr3	128204414	128216760	GATA2	GATA binding protein 2	11575.6096
114	chr11	61354115	61379070	RPLP0P2	ribosomal protein lateral stalk subunit P0 pseudogene 2	11526.7145
115	chr15	93351030	93365629	ASB9P1	ankyrin repeat and SOCS box containing 9 pseudogene 1	11525.9105
116	chr3	128052455	128069358	DNAJB8-AS1	DNAJB8 antisense RNA 1	11507.5624
117	chr11	114033931	114051801	NNMT	nicotinamide N-methyltransferase	11306.349
118	chr11	66245219	66250195	DPP3	dipeptidyl peptidase 3	11255.712
119	chr17	27068721	27076775	TRAF4	TNF receptor associated factor 4	11100.8282
120	chr9	110215888	110229055	KLF4	Kruppel like factor 4	11023.4124
121	chr11	66484183	66497235	SPTBN2	spectrin beta, non-erythrocytic 2	10983.258
122	chr1	234735181	234749545	IRF2BP2	interferon regulatory factor 2 binding protein 2	10972.6596
123	chr9	112967371	112972267	C9orf152	chromosome 9 open reading frame 152	10889.6832
124	chr7	73155596	73166074	ABHD11	abhydrolase domain containing 11	10642.5046
125	chr1	16993128	16995114	MIR3675	microRNA 3675	10601.0694
126	chr8	134306140	134312073	NDRG1	N-myc downstream regulated 1	10557.1802
127	chr11	66821147	66826934	RHOD	ras homolog family member D	10446.1137
128	chr22	23863040	23882239	IGLL1	immunoglobulin lambda like polypeptide 1	10430.8167
129	chr10	1479463	1503329	ADARB2-AS1	ADARB2 antisense RNA 1	10415.1224
130	chr8	126440600	126448296	TRIB1	tribbles pseudokinase 1	10374.9776

Rank	chr	start	end	Gene	Gene description	H3K27ac signal
131	chr17	26821601	26827527	FOXN1	forkhead box N1	10257.3134
132	chr1	16967618	16973092	MST1P2	macrophage stimulating 1 pseudogene 2	10207.3678
133	chr21	43012515	43022930	LINC00111	long intergenic non-protein coding RNA 111	10155.6665
134	chr15	89667426	89674821	ABHD2	abhydrolase domain containing 2	10148.898
135	chr19	39327483	39343932	HNRNPL	heterogeneous nuclear ribonucleoprotein L	10127.6493
136	chr11	64803717	64811019	SAC3D1	SAC3 domain containing 1	10100.8566
137	chr3	13133268	13155710	IQSEC1	IQ motif and Sec7 domain 1	10078.7022
138	chr8	38589957	38594703	TACC1	transforming acidic coiled-coil containing protein 1	10071.012
139	chr11	64331915	64349411	SLC22A11	solute carrier family 22 member 11	10016.46
140	chr11	65323160	65329106	LTBP3	latent transforming growth factor beta binding protein 3	9980.361
141	chr11	64214745	64218602	LOC100996455	uncharacterized LOC100996455	9966.1023
142	chr14	105938537	105950049	CRIP2	cysteine rich protein 2	9954.4264
143	chr3	13020315	13037501	IQSEC1	IQ motif and Sec7 domain 1	9930.0708
144	chr3	42517344	42532653	VIPR1	vasoactive intestinal peptide receptor 1	9927.8865
145	chr10	46989908	46999118	GPRIN2	G protein regulated inducer of neurite outgrowth 2	9922.854
146	chr17	17588438	17601761	RAI1	retinoic acid induced 1	9914.9766
147	chr1	17065963	17068404	CROCC	ciliary rootlet coiled-coil, rootletin	9851.1437
148	chr17	79301749	79321192	TMEM105	transmembrane protein 105	9812.8821
149	chr11	67795919	67800948	NDUFS8	NADH:ubiquinone oxidoreductase core subunit S8	9773.3586
150	chr8	140866205	140870743	KCNK9	potassium two pore domain channel subfamily K member 9	9765.3222
151	chr8	134270645	134283504	NDRG1	N-myc downstream regulated 1	9740.6925
152	chr21	45844396	45859508	TRPM2	transient receptor potential cation channel subfamily M member 2	9710.9712
153	chr8	101961576	101966361	YWHAZ	tyrosine 3-monooxygenase/tryptophan 5-monooxygenase activation protein zeta	9695.8455
154	chr21	43915001	43919567	RSPH1	radial spoke head 1 homolog	9695.4444
155	chr11	63436398	63440216	ATL3	atlastin GTPase 3	9683.5934
156	chr19	18474758	18492168	GDF15	growth differentiation factor 15	9660.809
157	chr21	44156440	44170257	PDE9A	phosphodiesterase 9A	9605.5784
158	chr19	49374876	49380099	PPP1R15A	protein phosphatase 1 regulatory subunit 15A	9588.3834
159	chr19	49989515	49994670	RPL13A	ribosomal protein L13a	9529.533
160	chr15	101437235	101443606	LRRK1	leucine rich repeat kinase 1	9525.2821
161	chr6	11092891	11097320	SMIM13	small integral membrane protein 13	9481.1603
162	chr17	80839479	80849604	TBCD	tubulin folding cofactor D	9478.0125
163	chr2	28816988	28845590	PLB1	phospholipase B1	9461.5416
164	chr17	77804444	77818636	CBX4	chromobox 4	9444.776
165	chr14	96341551	96355607	TUNAR	TCL1 upstream neural differentiation-associated RNA	9430.1704
166	chr2	238383214	238412626	MLPH	melanophilin	9408.8988
167	chr19	39644165	39659016	PAK4	p21 (RAC1) activated kinase 4	9347.2194
168	chr11	65554469	65563437	OVOL1	ovo like transcriptional repressor 1	9327.6168
169	chr17	48608664	48621626	EPN3	epsin 3	9257.4604
170	chr17	2898194	2909418	OR1D5	olfactory receptor family 1 subfamily D member 5	9217.1488
171	chr19	54691901	54697094	TSEN34	tRNA splicing endonuclease subunit 34	9150.5853
172	chr11	65767376	65772503	EIF1AD	eukaryotic translation initiation factor 1A domain containing	9057.8709
173	chr19	51371281	51381819	KLK2	kallikrein related peptidase 2	8986.8064
174	chr19	39892327	39904089	MIR4530	microRNA 4530	8966.1726
175	chr3	53177697	53191395	PRKCD	protein kinase C delta	8924.247

Rank	chr	start	end	Gene	Gene description	H3K27ac signal
176	chr20	35483531	35493170	SOGA1	suppressor of glucose, autophagy associated 1	8915.1111
177	chr8	101496126	101507818	ANKRD46	ankyrin repeat domain 46	8874.228
178	chr17	27275516	27280446	PHF12	PHD finger protein 12	8871.042
179	chr15	78497956	78514489	ACSBG1	acyl-CoA synthetase bubblegum family member 1	8757.5301
180	chr3	39181715	39195081	CSRNP1	cysteine and serine rich nuclear protein 1	8683.8902
181	chr8	144819861	144824278	FAM83H-AS1	FAM83H antisense RNA 1 (head to head)	8606.5245
182	chr1	26097307	26124207	SELENON	selenoprotein N	8605.31
183	chr19	50369054	50374630	PNKP	polynucleotide kinase 3'-phosphatase	8593.7312
184	chr15	90578029	90591252	ZNF710	zinc finger protein 710	8563.2148
185	chr17	44336510	44345346	LRRC37A	leucine rich repeat containing 37A	8556.7824
186	chr7	5567333	5572121	ACTB	actin beta	8505.4032
187	chr2	20304509	20326763	LAPTM4A	lysosomal protein transmembrane 4 alpha	8503.2534
188	chr5	179321374	179324403	TBC1D9B	TBC1 domain family member 9B	8466.055
189	chr21	37527528	37530224	CBR3-AS1	CBR3 antisense RNA 1	8432.5488
190	chr2	242822829	242835580	RTP5	receptor transporter protein 5 (putative)	8415.66
191	chr21	45552607	45556825	C21orf33	chromosome 21 open reading frame 33	8415.3318
192	chr21	43932750	43945766	SLC37A1	solute carrier family 37 member 1	8391.4152
193	chr10	47129112	47134994	HNRNPA1P33	heterogeneous nuclear ribonucleoprotein A1 pseudogene 33	8383.0264
194	chr17	34888752	34892028	PIGW	phosphatidylinositol glycan anchor biosynthesis class W	8346.2652
195	chr20	56277669	56284322	PMEPA1	prostate transmembrane protein, androgen induced 1	8341.5314
196	chr13	20692204	20703142	GJA3	gap junction protein alpha 3	8324.9118
197	chr17	43660728	43664687	LOC644172	mitogen-activated protein kinase 8 interacting protein 1 pseudogene	8293.3132
198	chr3	193848194	193860963	HES1	hes family bHLH transcription factor 1	8287.081
199	chr1	16839053	16842919	CROCCP3	ciliary rootlet coiled-coil, rootletin pseudogene 3	8216.4098
200	chr14	100027021	100046772	CCDC85C	coiled-coil domain containing 85C	8202.5903
201	chr13	112726162	112728464	SOX1	SRY-box 1	8200.1844
202	chr15	75106753	75111650	LMAN1L	lectin, mannose binding 1 like	8195.1295
203	chr3	112989119	112994705	BOC	BOC cell adhesion associated, oncogene regulated	8062.2738
204	chr15	90691296	90705192	SEMA4B	semaphorin 4B	8038.836
205	chr8	142126078	142138497	DENND3	DENN domain containing 3	7959.3371
206	chr5	176727486	176732149	PRELID1	PRELI domain containing 1	7854.3572
207	chr4	77133797	77138121	FAM47E	family with sequence similarity 47 member E	7787.524
208	chr10	104177667	104183219	PSD	pleckstrin and Sec7 domain containing	7748.9264

Online Table VIII: ERG-dependent super-enhancers in HUVEC. Super-enhancer-associated gene symbols and description. Log_2 fold change (FC) reported from differential analysis comparing ChIP-seq signal in siERG against siCtl in the 1015 super-enhancers identified by H3K27ac enrichment in siCtl. This table spans 14 pages.

Gene	Description	Log_2FC
PALD1	phosphatase domain containing, paladin 1	-1.85
TSPAN18	tetraspanin 18	-1.83
LARS2-AS1	LARS2 antisense RNA 1	-1.76
GDPD5	glycerophosphodiester phosphodiesterase domain containing 5	-1.71
AQP1	aquaporin 1 (Colton blood group)	-1.57
TNS1	tensin 1	-1.48
N4BP3	NEDD4 binding protein 3	-1.45
PITRM1	pitrilysin metallopeptidase 1	-1.45
ARL11	ADP ribosylation factor like GTPase 11	-1.42
CLDN5	claudin 5	-1.41
KLF13	Kruppel like factor 13	-1.36
PCDH12	protocadherin 12	-1.33
MYO10	myosin X	-1.28
CCR7	C-C motif chemokine receptor 7	-1.27
C20orf204	chromosome 20 open reading frame 204	-1.24
IQSEC1	IQ motif and Sec7 domain 1	-1.23
WSCD1	WSC domain containing 1	-1.21
RP1L1	RP1 like 1	-1.16
LY86	lymphocyte antigen 86	-1.14
DLL4	delta like canonical Notch ligand 4	-1.10
RALB	RAS like proto-oncogene B	-1.08
FAM192A	family with sequence similarity 192 member A	-1.07
PSMG1	proteasome assembly chaperone 1	-1.06
NOTCH4	notch 4	-1.04
LDB2	LIM domain binding 2	-1.02
MYBBP1A	MYB binding protein 1a	-1.01
VPS53	VPS53, GARP complex subunit	-0.99
MTMR9LP	myotubularin related protein 9-like, pseudogene	-0.97
NRARP	NOTCH regulated ankyrin repeat protein	-0.96
SOX18	SRY-box 18	-0.93
LINC01968	long intergenic non-protein coding RNA 1968	-0.91
SUN1	Sad1 and UNC84 domain containing 1	-0.91
MMS19	MMS19 homolog, cytosolic iron-sulfur assembly component	-0.90
LOC100128531	uncharacterized LOC100128531	-0.90
LDLRAP1	low density lipoprotein receptor adaptor protein 1	-0.88
C10orf25	chromosome 10 open reading frame 25	-0.88
MIR1301	microRNA 1301	-0.87
JAM3	junctional adhesion molecule 3	-0.87
SLC43A3	solute carrier family 43 member 3	-0.85
MIR4761	microRNA 4761	-0.85
TAOK2	TAO kinase 2	-0.84
CYB561	cytochrome b561	-0.83
SPRY4	sprouty RTK signaling antagonist 4	-0.82
C19orf54	chromosome 19 open reading frame 54	-0.82
CAMK2B	calcium/calmodulin dependent protein kinase II beta	-0.81
LINC01558	long intergenic non-protein coding RNA 1558	-0.80
ETS1	ETS proto-oncogene 1, transcription factor	-0.80
LXN	latexin	-0.78
ADGRF5	adhesion G protein-coupled receptor F5	-0.78
TSEN54	tRNA splicing endonuclease subunit 54	-0.78
TECPR1	tectonin beta-propeller repeat containing 1	-0.77
CYFIP1	cytoplasmic FMR1 interacting protein 1	-0.77
IFT122	intraflagellar transport 122	-0.77
GIMAP8	GTPase, IMAP family member 8	-0.77
FAM53B	family with sequence similarity 53 member B	-0.77
ARHGAP27	Rho GTPase activating protein 27	-0.77
GGA3	golgi associated, gamma adaptin ear containing, ARF binding protein 3	-0.76
AFAP1L1	actin filament associated protein 1 like 1	-0.75
DYSF	dysferlin	-0.74
ANGPT2	angiopoietin 2	-0.74
SMNDC1	survival motor neuron domain containing 1	-0.74
MGMT	O-6-methylguanine-DNA methyltransferase	-0.74
SLC27A3	solute carrier family 27 member 3	-0.73
SPRY1	sprouty RTK signaling antagonist 1	-0.73

PDE2A	phosphodiesterase 2A	-0.73
SEC14L1	SEC14 like lipid binding 1	-0.73
CCDC85C	coiled-coil domain containing 85C	-0.72
SEC14L1	SEC14 like lipid binding 1	-0.72
ADORA2A	adenosine A2a receptor	-0.72
ADGRG1	adhesion G protein-coupled receptor G1	-0.71
CEP68	centrosomal protein 68	-0.70
PPP1R16B	protein phosphatase 1 regulatory subunit 16B	-0.70
PDGFB	platelet derived growth factor subunit B	-0.70
MIR4711	microRNA 4711	-0.70
GSEC	G-quadruplex forming sequence containing lncRNA	-0.70
CD34	CD34 molecule	-0.69
EFCC1	EF-hand and coiled-coil domain containing 1	-0.69
TMEM204	transmembrane protein 204	-0.69
MYO18A	myosin XVIIIA	-0.69
NID1	nidogen 1	-0.68
PXDN	peroxidasin	-0.68
ZNF366	zinc finger protein 366	-0.68
ANKS1A	ankyrin repeat and sterile alpha motif domain containing 1A	-0.68
RAPGEF1	Rap guanine nucleotide exchange factor 1	-0.67
IQCK	IQ motif containing K	-0.67
ADARB1	adenosine deaminase, RNA specific B1	-0.67
GRB10	growth factor receptor bound protein 10	-0.66
PIM3	Pim-3 proto-oncogene, serine/threonine kinase	-0.66
ITPKB	inositol-trisphosphate 3-kinase B	-0.65
PODXL	podocalyxin like	-0.65
ARHGAP12	Rho GTPase activating protein 12	-0.65
LINC00926	long intergenic non-protein coding RNA 926	-0.65
SEC14L1	SEC14 like lipid binding 1	-0.64
STK10	serine/threonine kinase 10	-0.62
SOCS3	suppressor of cytokine signaling 3	-0.61
ARHGEF7	Rho guanine nucleotide exchange factor 7	-0.60
VASH1	vasohibin 1	-0.60
RDX	radixin	-0.59
HMG20A	high mobility group 20A	-0.59
LINC00895	long intergenic non-protein coding RNA 895	-0.59
JAK1	Janus kinase 1	-0.58
GPR146	G protein-coupled receptor 146	-0.58
NUDT7	nudix hydrolase 7	-0.58
MIR4720	microRNA 4720	-0.58
HOMER3	homer scaffolding protein 3	-0.58
SLC19A1	solute carrier family 19 member 1	-0.58
STS	steroid sulfatase	-0.58
PKD1L1	polycystin 1 like 1, transient receptor potential channel interacting	-0.58
PREX1	phosphatidylinositol-3,4,5-trisphosphate dependent Rac exchange factor 1	-0.58
AP1B1	adaptor related protein complex 1 beta 1 subunit	-0.58
RNU6-34P	RNA, U6 small nuclear 34, pseudogene	-0.57
EPAS1	endothelial PAS domain protein 1	-0.56
PITRM1	pitrilysin metallopeptidase 1	-0.56
CMKLR1	chemerin chemokine-like receptor 1	-0.56
PPM1F	protein phosphatase, Mg2+/Mn2+ dependent 1F	-0.56
TIE1	tyrosine kinase with immunoglobulin like and EGF like domains 1	-0.55
DLX2	distal-less homeobox 2	-0.55
MIR5193	microRNA 5193	-0.55
MIR4296	microRNA 4296	-0.55
CALM1	calmodulin 1	-0.55
PPP1R13B	protein phosphatase 1 regulatory subunit 13B	-0.55
GALNT15	polypeptide N-acetylgalactosaminyltransferase 15	-0.54
NDST1	N-deacetylase and N-sulfotransferase 1	-0.54
AGFG2	ArfGAP with FG repeats 2	-0.54
KCNJ1	potassium voltage-gated channel subfamily J member 1	-0.53
WARS	tryptophanyl-tRNA synthetase	-0.53
GCHFR	GTP cyclohydrolase I feedback regulator	-0.53
EFNA1	ephrin A1	-0.52
FAM78A	family with sequence similarity 78 member A	-0.52
PPCDC	phosphopantothenoylcysteine decarboxylase	-0.52
FGD5	FYVE, RhoGEF and PH domain containing 5	-0.51
ITPR3	inositol 1,4,5-trisphosphate receptor type 3	-0.51
SOX17	SRY-box 17	-0.51
ABHD2	abhydrolase domain containing 2	-0.51
WSCD1	WSC domain containing 1	-0.51
PRKD2	protein kinase D2	-0.51

CELA3B	chymotrypsin like elastase family member 3B	-0.50
POLR1A	RNA polymerase I subunit A	-0.50
LRRFIP1	LRR binding FLII interacting protein 1	-0.50
NNMT	nicotinamide N-methyltransferase	-0.50
SLC9A3R2	SLC9A3 regulator 2	-0.50
DLGAP4	DLG associated protein 4	-0.50
NDUFS2	NADH:ubiquinone oxidoreductase core subunit S2	-0.49
GPR17	G protein-coupled receptor 17	-0.49
LINC02363	long intergenic non-protein coding RNA 2363	-0.49
SH2D3C	SH2 domain containing 3C	-0.49
FADS3	fatty acid desaturase 3	-0.49
LRRC32	leucine rich repeat containing 32	-0.49
SEC14L1	SEC14 like lipid binding 1	-0.49
DEPP1	DEPP1, autophagy regulator	-0.48
TNFRSF1B	TNF receptor superfamily member 1B	-0.46
TMEM140	transmembrane protein 140	-0.46
DOCK9	dedicator of cytokinesis 9	-0.46
PGF	placental growth factor	-0.46
ARHGAP27P1	Rho GTPase activating protein 27 pseudogene 1	-0.46
CALCRL	calcitonin receptor like receptor	-0.45
UBAC1	UBA domain containing 1	-0.45
CDK2AP1	cyclin dependent kinase 2 associated protein 1	-0.45
SPTLC2	serine palmitoyltransferase long chain base subunit 2	-0.45
DOCK6	dedicator of cytokinesis 6	-0.45
RALGAPA2	Ral GTPase activating protein catalytic alpha subunit 2	-0.45
ABCA4	ATP binding cassette subfamily A member 4	-0.44
PIK3C2B	phosphatidylinositol-4-phosphate 3-kinase catalytic subunit type 2 beta	-0.44
MFSD4A	major facilitator superfamily domain containing 4A	-0.44
HEIH	hepatocellular carcinoma up-regulated EZH2-associated long non-coding RNA	-0.44
GSN	gelsolin	-0.44
TSPAN15	tetraspanin 15	-0.44
MCAM	melanoma cell adhesion molecule	-0.44
HDAC7	histone deacetylase 7	-0.44
LTA4H	leukotriene A4 hydrolase	-0.44
NUDT14	nudix hydrolase 14	-0.44
CD93	CD93 molecule	-0.44
BCOR	BCL6 corepressor	-0.44
HAAO	3-hydroxyanthranilate 3,4-dioxygenase	-0.43
PRKAR1B	protein kinase cAMP-dependent type I regulatory subunit beta	-0.43
PML	promyelocytic leukemia	-0.43
TBCD	tubulin folding cofactor D	-0.43
MIR4748	microRNA 4748	-0.43
PGLS	6-phosphogluconolactonase	-0.43
BIN1	bridging integrator 1	-0.42
IKBKB	inhibitor of nuclear factor kappa B kinase subunit beta	-0.42
EEF1G	eukaryotic translation elongation factor 1 gamma	-0.42
SEMA6B	semaphorin 6B	-0.42
TNFAIP8L1	TNF alpha induced protein 8 like 1	-0.42
RPS10P7	ribosomal protein S10 pseudogene 7	-0.41
CCDC12	coiled-coil domain containing 12	-0.41
FAM43A	family with sequence similarity 43 member A	-0.41
GIMAP4	GTPase, IMAP family member 4	-0.41
EGFL7	EGF like domain multiple 7	-0.41
CAVIN3	caveolae associated protein 3	-0.41
RAPGEF3	Rap guanine nucleotide exchange factor 3	-0.41
HERC1	HECT and RLD domain containing E3 ubiquitin protein ligase family member 1	-0.41
EEF2K	eukaryotic elongation factor 2 kinase	-0.41
ARHGEF15	Rho guanine nucleotide exchange factor 15	-0.41
GNB1L	G protein subunit beta 1 like	-0.41
KIAA1671	KIAA1671	-0.41
DCST1-AS1	DCST1 antisense RNA 1	-0.40
SH3TC1	SH3 domain and tetratricopeptide repeats 1	-0.40
HBEGF	heparin binding EGF like growth factor	-0.40
MIR4673	microRNA 4673	-0.40
LYZL2	lysozyme like 2	-0.40
CTBP2	C-terminal binding protein 2	-0.40
ARHGEF17	Rho guanine nucleotide exchange factor 17	-0.40
ARRB1	arrestin beta 1	-0.40
HDAC5	histone deacetylase 5	-0.40
RHBDF2	rhomboid 5 homolog 2	-0.40
SMTN	smoothelin	-0.40
ANKRD55	ankyrin repeat domain 55	-0.39

RGS3	regulator of G protein signaling 3	-0.39
FRY	FRY microtubule binding protein	-0.39
MIR3183	microRNA 3183	-0.39
MIR4729	microRNA 4729	-0.39
GPR3	G protein-coupled receptor 3	-0.38
CDC42EP3	CDC42 effector protein 3	-0.38
ZFP36L2	ZFP36 ring finger protein like 2	-0.38
RAB11FIP5	RAB11 family interacting protein 5	-0.38
MIR4266	microRNA 4266	-0.38
RPL32P3	ribosomal protein L32 pseudogene 3	-0.38
PCDH1	protocadherin 1	-0.38
LRRC32	leucine rich repeat containing 32	-0.38
ARSA	arylsulfatase A	-0.38
MAST4	microtubule associated serine/threonine kinase family member 4	-0.37
RPL35	ribosomal protein L35	-0.37
PGAM1P5	phosphoglycerate mutase 1 pseudogene 5	-0.37
PIF1	PIF1 5'-to-3' DNA helicase	-0.37
ZNF710	zinc finger protein 710	-0.37
HSPB9	heat shock protein family B (small) member 9	-0.37
GNGT2	G protein subunit gamma transducin 2	-0.37
ABHD17A	abhydrolase domain containing 17A	-0.37
ABHD16B	abhydrolase domain containing 16B	-0.37
STARD8	StAR related lipid transfer domain containing 8	-0.37
GIPC2	GIPC PDZ domain containing family member 2	-0.36
LMNA	lamin A/C	-0.36
TNS1	tensin 1	-0.36
DKFZp451B082	uncharacterized LOC401282	-0.36
PODXL	podocalyxin like	-0.36
AGAP3	ArfGAP with GTPase domain, ankyrin repeat and PH domain 3	-0.36
PCNX3	pecanex homolog 3	-0.36
CDKN1B	cyclin dependent kinase inhibitor 1B	-0.36
FLJ21408	uncharacterized LOC400512	-0.36
IGFBP4	insulin like growth factor binding protein 4	-0.36
ICAM2	intercellular adhesion molecule 2	-0.36
LRRFIP1	LRR binding FLII interacting protein 1	-0.34
TPRG1L	tumor protein p63 regulated 1 like	-0.33
LINC01126	long intergenic non-protein coding RNA 1126	-0.33
SH3BP5	SH3 domain binding protein 5	-0.33
HLA-E	major histocompatibility complex, class I, E	-0.33
EXD3	exonuclease 3'-5' domain containing 3	-0.33
BMF	Bcl2 modifying factor	-0.33
MAPKBP1	mitogen-activated protein kinase binding protein 1	-0.33
PIGV	phosphatidylinositol glycan anchor biosynthesis class V	-0.32
TGFBR2	transforming growth factor beta receptor 2	-0.32
GFOD1	glucose-fructose oxidoreductase domain containing 1	-0.32
LOC254896	uncharacterized LOC254896	-0.32
EGFL7	EGF like domain multiple 7	-0.32
CHSY1	chondroitin sulfate synthase 1	-0.32
RIPOR1	RHO family interacting cell polarization regulator 1	-0.32
SUMO1P1	SUMO1 pseudogene 1	-0.32
ELOVL1	ELOVL fatty acid elongase 1	-0.31
INPP5D	inositol polyphosphate-5-phosphatase D	-0.31
APBB2	amyloid beta precursor protein binding family B member 2	-0.31
NOTCH4	notch 4	-0.31
FKBP5	FK506 binding protein 5	-0.31
RALGDS	ral guanine nucleotide dissociation stimulator	-0.31
KLF6	Kruppel like factor 6	-0.31
TRAF7	TNF receptor associated factor 7	-0.31
BANP	BTG3 associated nuclear protein	-0.31
MIR4745	microRNA 4745	-0.31
LAMA5	laminin subunit alpha 5	-0.31
NA	NA	-0.30
SLFNL1-AS1	SLFNL1 antisense RNA 1	-0.30
DNASE1L3	deoxyribonuclease 1 like 3	-0.30
GBA2	glucosylceramidase beta 2	-0.30
FOXO1	forkhead box O1	-0.30
KAT7	lysine acetyltransferase 7	-0.30
CDC25B	cell division cycle 25B	-0.30
PREX1	phosphatidylinositol-3,4,5-trisphosphate dependent Rac exchange factor 1	-0.30
FAAP20	Fanconi anemia core complex associated protein 20	-0.29
SOX13	SRY-box 13	-0.29
GNAI2	G protein subunit alpha i2	-0.29

IQCJ-SCHIP1	IQCJ-SCHIP1 readthrough	-0.29
NT5E	5'-nucleotidase ecto	-0.29
KLF9	Kruppel like factor 9	-0.29
DUSP5	dual specificity phosphatase 5	-0.29
MIR4688	microRNA 4688	-0.29
FMNL3	formin like 3	-0.29
WDFY1	WD repeat and FYVE domain containing 1	-0.28
FAM124B	family with sequence similarity 124 member B	-0.28
TMEM173	transmembrane protein 173	-0.28
DENND3	DENN domain containing 3	-0.28
SERPINH1	serpin family H member 1	-0.28
ISG20	interferon stimulated exonuclease gene 20	-0.28
CMIP	c-Maf inducing protein	-0.28
ALOX12-AS1	ALOX12 antisense RNA 1	-0.28
JUP	junction plakoglobin	-0.28
KLF2	Kruppel like factor 2	-0.28
FAM129C	family with sequence similarity 129 member C	-0.28
MIR3194	microRNA 3194	-0.28
SLC9A1	solute carrier family 9 member A1	-0.26
TDRD10	tudor domain containing 10	-0.26
TNR	tenascin R	-0.26
HEG1	heart development protein with EGF like domains 1	-0.26
RAI14	retinoic acid induced 14	-0.26
FSCN1	fascin actin-bundling protein 1	-0.26
EFCAB1	EF-hand calcium binding domain 1	-0.26
FAM213A	family with sequence similarity 213 member A	-0.26
UVRAG	UV radiation resistance associated	-0.26
ANKRD11	ankyrin repeat domain 11	-0.26
ATP8B1	ATPase phospholipid transporting 8B1	-0.26
COQ8B	coenzyme Q8B	-0.26
ADA	adenosine deaminase	-0.26
PLEKHG5	pleckstrin homology and RhoGEF domain containing G5	-0.25
ATP2B4	ATPase plasma membrane Ca2+ transporting 4	-0.25
ERGIC1	endoplasmic reticulum-golgi intermediate compartment 1	-0.25
PPP1R35	protein phosphatase 1 regulatory subunit 35	-0.25
DAB2IP	DAB2 interacting protein	-0.25
ADM	adrenomedullin	-0.25
TXNRD2	thioredoxin reductase 2	-0.25
HSPG2	heparan sulfate proteoglycan 2	-0.24
RXFP4	relaxin/insulin like family peptide receptor 4	-0.24
NES	nestin	-0.24
GPX1	glutathione peroxidase 1	-0.24
ZNF366	zinc finger protein 366	-0.24
CSNK1A1	casein kinase 1 alpha 1	-0.24
RNF144B	ring finger protein 144B	-0.24
ANKRD13D	ankyrin repeat domain 13D	-0.24
PHLDA1	pleckstrin homology like domain family A member 1	-0.24
ELK3	ELK3, ETS transcription factor	-0.24
CAVIN1	caveolae associated protein 1	-0.24
LSM4	LSM4 homolog, U6 small nuclear RNA and mRNA degradation associated	-0.24
ASAP2	ArfGAP with SH3 domain, ankyrin repeat and PH domain 2	-0.23
VGLL4	vestigial like family member 4	-0.23
SEMA3F	semaphorin 3F	-0.23
TNK2	tyrosine kinase non receptor 2	-0.23
LEMD2	LEM domain containing 2	-0.23
PDE7B	phosphodiesterase 7B	-0.23
CLDN15	claudin 15	-0.23
CAV1	caveolin 1	-0.23
TRIB1	tribbles pseudokinase 1	-0.23
FAM69B	family with sequence similarity 69 member B	-0.23
LZTS2	leucine zipper tumor suppressor 2	-0.23
GRASP	general receptor for phosphoinositides 1 associated scaffold protein	-0.23
TNS2	tensin 2	-0.23
DAD1	defender against cell death 1	-0.23
EHD4	EH domain containing 4	-0.23
GNA15	G protein subunit alpha 15	-0.23
ELF4	E74 like ETS transcription factor 4	-0.23
CLCN6	chloride voltage-gated channel 6	-0.21
RHOC	ras homolog family member C	-0.21
S100A10	S100 calcium binding protein A10	-0.21
SRGAP2	SLIT-ROBO Rho GTPase activating protein 2	-0.21
HOXD-AS2	HOXD cluster antisense RNA 2	-0.21

SASH1	SAM and SH3 domain containing 1	-0.21
ZNF277	zinc finger protein 277	-0.21
PREX2	phosphatidylinositol-3,4,5-trisphosphate dependent Rac exchange factor 2	-0.21
RAPGEF1	Rap guanine nucleotide exchange factor 1	-0.21
TOR4A	torsin family 4 member A	-0.21
WSB1	WD repeat and SOCS box containing 1	-0.21
EGLN2	egl-9 family hypoxia inducible factor 2	-0.21
LINC00313	long intergenic non-protein coding RNA 313	-0.21
CMTM7	CKLF like MARVEL transmembrane domain containing 7	-0.20
TMEM14EP	transmembrane protein 14E, pseudogene	-0.20
LHFPL3-AS2	LHFPL3 antisense RNA 2	-0.20
INS-IGF2	INS-IGF2 readthrough	-0.20
RELT	RELT, TNF receptor	-0.20
HIP1R	huntingtin interacting protein 1 related	-0.20
MTMR10	myotubularin related protein 10	-0.20
CANT1	calcium activated nucleotidase 1	-0.20
MKNK2	MAP kinase interacting serine/threonine kinase 2	-0.20
GADD45B	growth arrest and DNA damage inducible beta	-0.20
LENG8	leukocyte receptor cluster member 8	-0.20
PLCG1	phospholipase C gamma 1	-0.20
VPS13D	vacuolar protein sorting 13 homolog D	-0.19
CLIC4	chloride intracellular channel 4	-0.19
PCBP1-AS1	PCBP1 antisense RNA 1	-0.19
VGLL4	vestigial like family member 4	-0.19
TACC3	transforming acidic coiled-coil containing protein 3	-0.19
FAM198B	family with sequence similarity 198 member B	-0.19
MSH5-SAPCD1	MSH5-SAPCD1 readthrough (NMD candidate)	-0.19
SHB	SH2 domain containing adaptor protein B	-0.19
MIR126	microRNA 126	-0.19
JCAD	junctional cadherin 5 associated	-0.19
MIR548Z	microRNA 548z	-0.19
IL4R	interleukin 4 receptor	-0.19
UNC13D	unc-13 homolog D	-0.19
CIC	capicua transcriptional repressor	-0.19
RIN2	Ras and Rab interactor 2	-0.19
TAL1	TAL bHLH transcription factor 1, erythroid differentiation factor	-0.18
EPAS1	endothelial PAS domain protein 1	-0.18
LINC01816	long intergenic non-protein coding RNA 1816	-0.18
MIR3692	microRNA 3692	-0.18
TNRC18	trinucleotide repeat containing 18	-0.18
TACC2	transforming acidic coiled-coil containing protein 2	-0.18
NEAT1	nuclear paraspeckle assembly transcript 1 (non-protein coding)	-0.18
FGD4	FYVE, RhoGEF and PH domain containing 4	-0.18
CMIP	c-Maf inducing protein	-0.18
GRAP	GRB2-related adaptor protein	-0.18
ARHGAP23	Rho GTPase activating protein 23	-0.18
SAMD14	sterile alpha motif domain containing 14	-0.18
SIPA1L3	signal induced proliferation associated 1 like 3	-0.18
S100A16	S100 calcium binding protein A16	-0.16
NUAK2	NUAK family kinase 2	-0.16
EPAS1	endothelial PAS domain protein 1	-0.16
SSFA2	sperm specific antigen 2	-0.16
SH3BP2	SH3 domain binding protein 2	-0.16
NRM	nurim	-0.16
CD109	CD109 molecule	-0.16
RPS6KA2	ribosomal protein S6 kinase A2	-0.16
SLC25A25	solute carrier family 25 member 25	-0.16
GRK5	G protein-coupled receptor kinase 5	-0.16
BCAT1	branched chain amino acid transaminase 1	-0.16
ATP11A	ATPase phospholipid transporting 11A	-0.16
CLEC14A	C-type lectin domain containing 14A	-0.16
MTA1	metastasis associated 1	-0.16
HOXB3	homeobox B3	-0.16
TRIM47	tripartite motif containing 47	-0.16
NOP53	NOP53 ribosome biogenesis factor	-0.16
TRIB3	tribbles pseudokinase 3	-0.16
EPAS1	endothelial PAS domain protein 1	-0.15
NPAS2	neuronal PAS domain protein 2	-0.15
H1FOO	H1 histone family member O oocyte specific	-0.15
PTPA	protein phosphatase 2 phosphatase activator	-0.15
CCDC85B	coiled-coil domain containing 85B	-0.15
SLC35F2	solute carrier family 35 member F2	-0.15

LATS2	large tumor suppressor kinase 2	-0.15
COL4A1	collagen type IV alpha 1 chain	-0.15
IRF2BPL	interferon regulatory factor 2 binding protein like	-0.15
MYZAP	myocardial zonula adherens protein	-0.15
LIG1	DNA ligase 1	-0.15
CASTOR1	cytosolic arginine sensor for mTORC1 subunit 1	-0.15
C1QTNF6	C1q and TNF related 6	-0.15
PLA2G6	phospholipase A2 group VI	-0.15
TMSB4X	thymosin beta 4, X-linked	-0.15
F11R	F11 receptor	-0.14
EPAS1	endothelial PAS domain protein 1	-0.14
MIR4435-1	microRNA 4435-1	-0.14
IGF2BP2-AS1	IGF2BP2 antisense RNA 1	-0.14
MIR4645	microRNA 4645	-0.14
GFOD1	glucose-fructose oxidoreductase domain containing 1	-0.14
MIR4648	microRNA 4648	-0.14
SOX7	SRY-box 7	-0.14
SNCG	synuclein gamma	-0.14
TSSC4	tumor suppressing subtransferable candidate 4	-0.14
FADS2	fatty acid desaturase 2	-0.14
MALAT1	metastasis associated lung adenocarcinoma transcript 1 (non-protein coding)	-0.14
MIR4489	microRNA 4489	-0.14
SETD1B	SET domain containing 1B	-0.14
PRKCH	protein kinase C eta	-0.14
PRKCH	protein kinase C eta	-0.14
ARHGAP17	Rho GTPase activating protein 17	-0.14
MTSS1L	MTSS1L, I-BAR domain containing	-0.14
TRIM25	tripartite motif containing 25	-0.14
NFATC1	nuclear factor of activated T-cells 1	-0.14
STX10	syntaxin 10	-0.14
MIR3190	microRNA 3190	-0.14
LINC00656	long intergenic non-protein coding RNA 656	-0.14
MIR4258	microRNA 4258	-0.12
MEF2D	myocyte enhancer factor 2D	-0.12
FRMD4B	FERM domain containing 4B	-0.12
TMEM44	transmembrane protein 44	-0.12
MIR143	microRNA 143	-0.12
SPARC	secreted protein acidic and cysteine rich	-0.12
MIR1260B	microRNA 1260b	-0.12
MIR4740	microRNA 4740	-0.12
LOC642852	uncharacterized LOC642852	-0.12
MSN	moesin	-0.12
HPCAL1	hippocalcin like 1	-0.11
VAMP5	vesicle associated membrane protein 5	-0.11
PHLDB2	pleckstrin homology like domain family B member 2	-0.11
RGS12	regulator of G protein signaling 12	-0.11
WRNIP1	Werner helicase interacting protein 1	-0.11
BRI3	brain protein I3	-0.11
SPAAR	small regulatory polypeptide of amino acid response	-0.11
AQP7P1	aquaporin 7 pseudogene 1	-0.11
ITGA11	integrin subunit alpha 11	-0.11
LOC646938	TBC1 domain family member 2B pseudogene	-0.11
MIR4516	microRNA 4516	-0.11
LOC100287036	uncharacterized LOC100287036	-0.11
CEACAM16	carcinoembryonic antigen related cell adhesion molecule 16	-0.11
GNG11	G protein subunit gamma 11	-0.10
SLC25A45	solute carrier family 25 member 45	-0.10
SH2B3	SH2B adaptor protein 3	-0.10
TBC1D2B	TBC1 domain family member 2B	-0.10
NTAN1	N-terminal asparagine amidase	-0.10
SCARF1	scavenger receptor class F member 1	-0.10
SMG6	SMG6, nonsense mediated mRNA decay factor	-0.10
TIMP2	TIMP metallopeptidase inhibitor 2	-0.10
TIMP2	TIMP metallopeptidase inhibitor 2	-0.10
LDLR	low density lipoprotein receptor	-0.10
PNKP	polynucleotide kinase 3'-phosphatase	-0.10
MDS2	myelodysplastic syndrome 2 translocation associated	-0.08
TMEM39B	transmembrane protein 39B	-0.08
LINC01135	long intergenic non-protein coding RNA 1135	-0.08
TNS1	tensin 1	-0.08
TRAK1	trafficking kinesin protein 1	-0.08
FAM198B	family with sequence similarity 198 member B	-0.08

CXXC5	CXXC finger protein 5	-0.08
FOXC1	forkhead box C1	-0.08
EPHB4	EPH receptor B4	-0.08
LOC100507156	uncharacterized LOC100507156	-0.08
ST3GAL1	ST3 beta-galactoside alpha-2,3-sialyltransferase 1	-0.08
MIR541	microRNA 541	-0.08
CRIP2	cysteine rich protein 2	-0.08
MAP2K3	mitogen-activated protein kinase kinase 3	-0.08
CIRBP	cold inducible RNA binding protein	-0.08
JAG1	jagged 1	-0.08
PISD	phosphatidylserine decarboxylase	-0.08
PEAR1	platelet endothelial aggregation receptor 1	-0.07
LOC148696	uncharacterized LOC148696	-0.07
ARF1	ADP ribosylation factor 1	-0.07
FEZ2	fasciculation and elongation protein zeta 2	-0.07
MALL	mal, T-cell differentiation protein like	-0.07
MIR1244-2	microRNA 1244-2	-0.07
LINC00696	long intergenic non-protein coding RNA 696	-0.07
TM4SF18	transmembrane 4 L six family member 18	-0.07
BRD2	bromodomain containing 2	-0.07
KMT2E-AS1	KMT2E antisense RNA 1 (head to head)	-0.07
C8orf58	chromosome 8 open reading frame 58	-0.07
AKAP2	A-kinase anchoring protein 2	-0.07
RXRA	retinoid X receptor alpha	-0.07
FES	FES proto-oncogene, tyrosine kinase	-0.07
ST3GAL2	ST3 beta-galactoside alpha-2,3-sialyltransferase 2	-0.07
LOC100129617	uncharacterized LOC100129617	-0.07
MSL1	male specific lethal 1 homolog	-0.07
CUEDC1	CUE domain containing 1	-0.07
NFIC	nuclear factor I C	-0.07
PIP5K1C	phosphatidylinositol-4-phosphate 5-kinase type 1 gamma	-0.07
NFIX	nuclear factor I X	-0.07
APP	amyloid beta precursor protein	-0.07
TAGLN2	transgelin 2	-0.06
LIMD1-AS1	LIMD1 antisense RNA 1	-0.06
ACTB	actin beta	-0.06
TNFRSF10D	TNF receptor superfamily member 10d	-0.06
PPP2R2A	protein phosphatase 2 regulatory subunit Balpha	-0.06
MRPL15	mitochondrial ribosomal protein L15	-0.06
SH2D4B	SH2 domain containing 4B	-0.06
MIR4681	microRNA 4681	-0.06
SWAP70	switching B-cell complex subunit SWAP70	-0.06
SLC3A2	solute carrier family 3 member 2	-0.06
FZD4	frizzled class receptor 4	-0.06
ANKRD33	ankyrin repeat domain 33	-0.06
MIR5188	microRNA 5188	-0.06
ZFP36L1	ZFP36 ring finger protein like 1	-0.06
TUBB6	tubulin beta 6 class V	-0.06
MIR1227	microRNA 1227	-0.06
SOGA1	suppressor of glucose, autophagy associated 1	-0.06
IFFO2	intermediate filament family orphan 2	-0.04
TINAGL1	tubulointerstitial nephritis antigen like 1	-0.04
LINC01135	long intergenic non-protein coding RNA 1135	-0.04
LINC01132	long intergenic non-protein coding RNA 1132	-0.04
LINC01132	long intergenic non-protein coding RNA 1132	-0.04
CCDC12	coiled-coil domain containing 12	-0.04
LOC153684	uncharacterized LOC153684	-0.04
DUSP1	dual specificity phosphatase 1	-0.04
BMPER	BMP binding endothelial regulator	-0.04
VOPP1	VOPP1, WBP1/VOPP1 family member	-0.04
TONSL	tonsoku like, DNA repair protein	-0.04
NFIB	nuclear factor I B	-0.04
DAPK1	death associated protein kinase 1	-0.04
DDIT4	DNA damage inducible transcript 4	-0.04
TSPAN14	tetraspanin 14	-0.04
AHNAK	AHNAK nucleoprotein	-0.04
NECTIN2	nectin cell adhesion molecule 2	-0.04
RTEL1-TNFRSF6B	RTEL1-TNFRSF6B readthrough (NMD candidate)	-0.04
CARD10	caspase recruitment domain family member 10	-0.04
PDGFB	platelet derived growth factor subunit B	-0.04
LMNA	lamin A/C	-0.03
CFLAR-AS1	CFLAR antisense RNA 1	-0.03

BHLHE40-AS1	BHLHE40 antisense RNA 1	-0.03
TREX1	three prime repair exonuclease 1	-0.03
AFF1	AF4/FMR2 family member 1	-0.03
TNIP1	TNFAIP3 interacting protein 1	-0.03
ATP6V1G2-DDX39B	ATP6V1G2-DDX39B readthrough (NMD candidate)	-0.03
DNAJB12	DnaJ heat shock protein family (Hsp40) member B12	-0.03
ZMIZ1-AS1	ZMIZ1 antisense RNA 1	-0.03
UBASH3B	ubiquitin associated and SH3 domain containing B	-0.03
SENCR	smooth muscle and endothelial cell enriched migration/differentiation-associated lncRNA	-0.03
SSH1	slingshot protein phosphatase 1	-0.03
NCOR2	nuclear receptor corepressor 2	-0.03
ZFP36L1	ZFP36 ring finger protein like 1	-0.03
CCDC9B	coiled-coil domain containing 9B	-0.03
ERI2	ERI1 exoribonuclease family member 2	-0.03
TK2	thymidine kinase 2, mitochondrial	-0.03
BCAR1	BCAR1, Cas family scaffolding protein	-0.03
LOC284454	uncharacterized LOC284454	-0.03
GRAMD1A	GRAM domain containing 1A	-0.03
COL18A1	collagen type XVIII alpha 1 chain	-0.03
ZNF436-AS1	ZNF436 antisense RNA 1	-0.01
PDE4B	phosphodiesterase 4B	-0.01
LRRC8C	leucine rich repeat containing 8 VRAC subunit C	-0.01
SNED1	sushi, nidogen and EGF like domains 1	-0.01
POMGNT2	protein O-linked mannose N-acetylglucosaminyltransferase 2 (beta 1,4-)	-0.01
ITGB5	integrin subunit beta 5	-0.01
XXYLT1-AS2	XXYLT1 antisense RNA 2	-0.01
ARHGAP24	Rho GTPase activating protein 24	-0.01
LAMA4	laminin subunit alpha 4	-0.01
HOXA-AS3	HOXA cluster antisense RNA 3	-0.01
TACC1	transforming acidic coiled-coil containing protein 1	-0.01
AZIN1	antizyme inhibitor 1	-0.01
FAM129B	family with sequence similarity 129 member B	-0.01
POLR2L	RNA polymerase II subunit L	-0.01
AMN1	antagonist of mitotic exit network 1 homolog	-0.01
SMAD6	SMAD family member 6	-0.01
RAI1	retinoic acid induced 1	-0.01
STK11	serine/threonine kinase 11	-0.01
ARRDC2	arrestin domain containing 2	-0.01
CBX7	chromobox 7	-0.01
FHL3	four and a half LIM domains 3	0.00
ZFP36L2	ZFP36 ring finger protein like 2	0.00
MIR26B	microRNA 26b	0.00
AMOTL2	angiomotin like 2	0.00
SOX4	SRY-box 4	0.00
IL6	interleukin 6	0.00
NOS3	nitric oxide synthase 3	0.00
LINC00589	long intergenic non-protein coding RNA 589	0.00
PDCD1LG2	programmed cell death 1 ligand 2	0.00
PRRC2B	proline rich coiled-coil 2B	0.00
GATA3	GATA binding protein 3	0.00
TRDMT1	tRNA aspartic acid methyltransferase 1	0.00
NRP1	neuropilin 1	0.00
ZMIZ1-AS1	ZMIZ1 antisense RNA 1	0.00
MIR4691	microRNA 4691	0.00
PXN	paxillin	0.00
CDH5	cadherin 5	0.00
PSMB3	proteasome subunit beta 3	0.00
RNF213	ring finger protein 213	0.00
TGIF1	TGFB induced factor homeobox 1	0.00
SBNO2	strawberry notch homolog 2	0.00
GPI	glucose-6-phosphate isomerase	0.00
PARVB	parvin beta	0.00
LOC100506801	uncharacterized LOC100506801	0.01
S1PR1	sphingosine-1-phosphate receptor 1	0.01
RASSF1	Ras association domain family member 1	0.01
MECOM	MDS1 and EVI1 complex locus	0.01
BDH1	3-hydroxybutyrate dehydrogenase 1	0.01
LOC648987	uncharacterized LOC648987	0.01
PDLIM4	PDZ and LIM domain 4	0.01
ARAP3	ArfGAP with RhoGAP domain, ankyrin repeat and PH domain 3	0.01
FLOT1	flotillin 1	0.01
SUFU	SUFU negative regulator of hedgehog signaling	0.01

BAG3	BCL2 associated athanogene 3	0.01
CTNND1	catenin delta 1	0.01
C15orf53	chromosome 15 open reading frame 53	0.01
MNT	MAX network transcriptional repressor	0.01
PICART1	p53-inducible cancer-associated RNA transcript 1	0.01
PECAM1	platelet and endothelial cell adhesion molecule 1	0.01
FKBP1A-SDCBP2	FKBP1A-SDCBP2 readthrough (NMD candidate)	0.01
LOC100131496	uncharacterized LOC100131496	0.01
CTSZ	cathepsin Z	0.01
FBXW4P1	F-box and WD repeat domain containing 4 pseudogene 1	0.01
PLXNB2	plexin B2	0.01
DHRS3	dehydrogenase/reductase 3	0.03
HSPG2	heparan sulfate proteoglycan 2	0.03
LINC01135	long intergenic non-protein coding RNA 1135	0.03
SNORA14B	small nucleolar RNA, H/ACA box 14B	0.03
ANKRD28	ankyrin repeat domain 28	0.03
TMEM212	transmembrane protein 212	0.03
MGAT4B	mannosyl (alpha-1,3-)-glycoprotein beta-1,4-N-acetylglucosaminyltransferase, isozyme B	0.03
CPNE5	copine 5	0.03
HSPB1	heat shock protein family B (small) member 1	0.03
PTK2	protein tyrosine kinase 2	0.03
ITPRIP	inositol 1,4,5-trisphosphate receptor interacting protein	0.03
ROBO4	roundabout guidance receptor 4	0.03
IFFO1	intermediate filament family orphan 1	0.03
MMP14	matrix metallopeptidase 14	0.03
ACTN1-AS1	ACTN1 antisense RNA 1	0.03
EML1	echinoderm microtubule associated protein like 1	0.03
AHNAK2	AHNAK nucleoprotein 2	0.03
MIR1469	microRNA 1469	0.03
LOC729683	uncharacterized LOC729683	0.03
DLGAP1-AS1	DLGAP1 antisense RNA 1	0.03
CSRP1	cysteine and glycine rich protein 1	0.04
KLF7	Kruppel like factor 7	0.04
PXDC1	PX domain containing 1	0.04
BMP6	bone morphogenetic protein 6	0.04
MIR1322	microRNA 1322	0.04
KANK1	KN motif and ankyrin repeat domains 1	0.04
RGS3	regulator of G protein signaling 3	0.04
PRPF18	pre-mRNA processing factor 18	0.04
FRMD4A	FERM domain containing 4A	0.04
PSMC3	proteasome 26S subunit, ATPase 3	0.04
ETV6	ETS variant 6	0.04
CDK17	cyclin dependent kinase 17	0.04
MBNL2	muscleblind like splicing regulator 2	0.04
RASA3	RAS p21 protein activator 3	0.04
RASA3	RAS p21 protein activator 3	0.04
PRRT2	proline rich transmembrane protein 2	0.04
LASP1	LIM and SH3 protein 1	0.04
CDC42EP4	CDC42 effector protein 4	0.04
ITGB4	integrin subunit beta 4	0.04
GGT5	gamma-glutamyltransferase 5	0.04
CDC42EP1	CDC42 effector protein 1	0.04
LOC730668	dynein heavy chain -like pseudogene	0.04
TGFBR2	transforming growth factor beta receptor 2	0.06
NEDD9	neural precursor cell expressed, developmentally down-regulated 9	0.06
PNMA2	PNMA family member 2	0.06
MIR4669	microRNA 4669	0.06
FRMD4A	FERM domain containing 4A	0.06
FAM107B	family with sequence similarity 107 member B	0.06
ZNF438	zinc finger protein 438	0.06
VWF	von Willebrand factor	0.06
EMP1	epithelial membrane protein 1	0.06
KRT80	keratin 80	0.06
DUSP6	dual specificity phosphatase 6	0.06
COL4A1	collagen type IV alpha 1 chain	0.06
MIR4710	microRNA 4710	0.06
RNF166	ring finger protein 166	0.06
PITPNA	phosphatidylinositol transfer protein alpha	0.06
SREBF1	sterol regulatory element binding transcription factor 1	0.06
STAT5A	signal transducer and activator of transcription 5A	0.06
MIR5010	microRNA 5010	0.06
KPNA2	karyopherin subunit alpha 2	0.06

RPTOR	regulatory associated protein of MTOR complex 1	0.06
ELL	elongation factor for RNA polymerase II	0.06
ERG	ERG, ETS transcription factor	0.06
CROCC	ciliary rootlet coiled-coil, rootletin	0.07
MCL1	MCL1, BCL2 family apoptosis regulator	0.07
GATA2	GATA binding protein 2	0.07
LINC01565	long intergenic non-protein coding RNA 1565	0.07
KDR	kinase insert domain receptor	0.07
ARHGEF28	Rho guanine nucleotide exchange factor 28	0.07
FOXK1	forkhead box K1	0.07
CAPZA2	capping actin protein of muscle Z-line alpha subunit 2	0.07
B4GALT1	beta-1,4-galactosyltransferase 1	0.07
LOC100133920	methylenetetrahydrofolate dehydrogenase (NADP+ dependent) 1 like pseudogene	0.07
MIR4672	microRNA 4672	0.07
TNKS1BP1	tankyrase 1 binding protein 1	0.07
FOXC2	forkhead box C2	0.07
SLC7A5	solute carrier family 7 member 5	0.07
MYL12B	myosin light chain 12B	0.07
HM13-AS1	HM13 antisense RNA 1	0.07
C21orf33	chromosome 21 open reading frame 33	0.07
TACSTD2	tumor associated calcium signal transducer 2	0.08
GADD45A	growth arrest and DNA damage inducible alpha	0.08
HDGF	heparin binding growth factor	0.08
CAVIN2	caveolae associated protein 2	0.08
AP2M1	adaptor related protein complex 2 mu 1 subunit	0.08
ANKRD55	ankyrin repeat domain 55	0.08
PLEC	plectin	0.08
NINJ1	ninjurin 1	0.08
PHLDB1	pleckstrin homology like domain family B member 1	0.08
CDK17	cyclin dependent kinase 17	0.08
NFKBIA	NFKB inhibitor alpha	0.08
SMAD6	SMAD family member 6	0.08
KCNN4	potassium calcium-activated channel subfamily N member 4	0.08
BCL3	B-cell CLL/lymphoma 3	0.08
BCL2L1	BCL2 like 1	0.08
SEC14L2	SEC14 like lipid binding 2	0.08
SSC4D	scavenger receptor cysteine rich family member with 4 domains	0.10
UBE2H	ubiquitin conjugating enzyme E2 H	0.10
PDGFRL	platelet derived growth factor receptor like	0.10
SLC25A37	solute carrier family 25 member 37	0.10
VAV2	vav guanine nucleotide exchange factor 2	0.10
ANKRD2	ankyrin repeat domain 2	0.10
MICAL2	microtubule associated monooxygenase, calponin and LIM domain containing 2	0.10
MIR4693	microRNA 4693	0.10
NINJ2	ninjurin 2	0.10
ITGA5	integrin subunit alpha 5	0.10
MIR5580	microRNA 5580	0.10
MIR4505	microRNA 4505	0.10
TXNRD2	thioredoxin reductase 2	0.10
LIMK2	LIM domain kinase 2	0.10
SH3BP4	SH3 domain binding protein 4	0.11
LOC100506178	uncharacterized LOC100506178	0.11
CAV2	caveolin 2	0.11
MIR1207	microRNA 1207	0.11
MYOF	myoferlin	0.11
DKK3	dickkopf WNT signaling pathway inhibitor 3	0.11
C11orf91	chromosome 11 open reading frame 91	0.11
RPSAP52	ribosomal protein SA pseudogene 52	0.11
AJUBA	ajuba LIM protein	0.11
ZBTB7A	zinc finger and BTB domain containing 7A	0.11
ERF	ETS2 repressor factor	0.11
CLDN14	claudin 14	0.11
COL18A1-AS2	COL18A1 antisense RNA 2	0.11
LOC100129534	small nuclear ribonucleoprotein polypeptide N pseudogene	0.12
EPHA2	EPH receptor A2	0.12
LOC100129046	uncharacterized LOC100129046	0.12
FOSL2	FOS like 2, AP-1 transcription factor subunit	0.12
TTYH3	tweety family member 3	0.12
UHRF2	ubiquitin like with PHD and ring finger domains 2	0.12
PTPRE	protein tyrosine phosphatase, receptor type E	0.12
C12orf75	chromosome 12 open reading frame 75	0.12
MPG	N-methylpurine DNA glycosylase	0.12

JUNB	JunB proto-oncogene, AP-1 transcription factor subunit	0.12
CAPN12	calpain 12	0.12
RTEL1-TNFRSF6B	RTEL1-TNFRSF6B readthrough (NMD candidate)	0.12
RNF19B	ring finger protein 19B	0.14
ARHGAP29	Rho GTPase activating protein 29	0.14
PALMD	palmdelphin	0.14
PLXNA2	plexin A2	0.14
MGAT5	mannosyl (alpha-1,6-)-glycoprotein beta-1,6-N-acetyl-glucosaminyltransferase	0.14
ZNF366	zinc finger protein 366	0.14
PARP12	poly(ADP-ribose) polymerase family member 12	0.14
MTUS1	microtubule associated scaffold protein 1	0.14
IL15RA	interleukin 15 receptor subunit alpha	0.14
CORO1C	coronin 1C	0.14
MIR4316	microRNA 4316	0.14
INO80C	INO80 complex subunit C	0.14
GPX4	glutathione peroxidase 4	0.14
SMIM24	small integral membrane protein 24	0.14
MIR3189	microRNA 3189	0.14
NOL4L	nucleolar protein 4 like	0.14
MYH9	myosin heavy chain 9	0.14
MRPL37	mitochondrial ribosomal protein L37	0.15
LINC01119	long intergenic non-protein coding RNA 1119	0.15
LIMS1	LIM zinc finger domain containing 1	0.15
SNRK-AS1	SNRK antisense RNA 1	0.15
FLNB	filamin B	0.15
UQCC2	ubiquinol-cytochrome c reductase complex assembly factor 2	0.15
SYNJ2-IT1	SYNJ2 intronic transcript 1	0.15
NOD1	nucleotide binding oligomerization domain containing 1	0.15
MIR1205	microRNA 1205	0.15
HK1	hexokinase 1	0.15
EXOC6	exocyst complex component 6	0.15
KHNYN	KH and NYN domain containing	0.15
TTC7B	tetratricopeptide repeat domain 7B	0.15
CENPN	centromere protein N	0.15
ENO3	enolase 3	0.15
SH3GL1	SH3 domain containing GRB2 like 1, endophilin A2	0.15
SDCBP2-AS1	SDCBP2 antisense RNA 1	0.15
TGM2	transglutaminase 2	0.15
NRP2	neuropilin 2	0.16
SLC29A1	solute carrier family 29 member 1 (Augustine blood group)	0.16
SERPINE1	serpin family E member 1	0.16
ARHGAP22	Rho GTPase activating protein 22	0.16
SAMD4A	sterile alpha motif domain containing 4A	0.16
SPHK1	sphingosine kinase 1	0.16
SPC24	SPC24, NDC80 kinetochore complex component	0.16
SSBP4	single stranded DNA binding protein 4	0.16
MIR3619	microRNA 3619	0.16
TRAPPC3	trafficking protein particle complex 3	0.18
EVA1B	eva-1 homolog B	0.18
WWTR1-AS1	WWTR1 antisense RNA 1	0.18
CTSB	cathepsin B	0.18
TM2D2	TM2 domain containing 2	0.18
MIR5094	microRNA 5094	0.18
PIEZO1	piezo type mechanosensitive ion channel component 1	0.18
GATA6-AS1	GATA6 antisense RNA 1 (head to head)	0.18
LINC00184	long intergenic non-protein coding RNA 184	0.19
FLJ42351	uncharacterized LOC400999	0.19
SLC6A6	solute carrier family 6 member 6	0.19
FLNB	filamin B	0.19
HOTAIRM1	HOXA transcript antisense RNA, myeloid-specific 1	0.19
CUBN	cubilin	0.19
VSIG2	V-set and immunoglobulin domain containing 2	0.19
TNFRSF1A	TNF receptor superfamily member 1A	0.19
SNTB2	syntrophin beta 2	0.19
ZNF521	zinc finger protein 521	0.19
TMEM189	transmembrane protein 189	0.19
MED15	mediator complex subunit 15	0.19
MACF1	microtubule-actin crosslinking factor 1	0.20
PLK3	polo like kinase 3	0.20
LEPR	leptin receptor	0.20
TNFSF18	TNF superfamily member 18	0.20
IRAK2	interleukin 1 receptor associated kinase 2	0.20

MAML3	mastermind like transcriptional coactivator 3	0.20
SYNPO	synaptopodin	0.20
MTHFD1L	methylenetetrahydrofolate dehydrogenase (NADP+ dependent) 1 like	0.20
CREB3L2	cAMP responsive element binding protein 3 like 2	0.20
FBXO32	F-box protein 32	0.20
SCD	stearoyl-CoA desaturase	0.20
NCOR2	nuclear receptor corepressor 2	0.20
ZFP36L1	ZFP36 ring finger protein like 1	0.20
C15orf54	chromosome 15 open reading frame 54 (putative)	0.20
SMAD3	SMAD family member 3	0.20
PTTG1IP	PTTG1 interacting protein	0.20
TRIOBP	TRIO and F-actin binding protein	0.20
FAM50B	family with sequence similarity 50 member B	0.21
NCOA7	nuclear receptor coactivator 7	0.21
MTHFD1L	methylenetetrahydrofolate dehydrogenase (NADP+ dependent) 1 like	0.21
NFIB	nuclear factor I B	0.21
ENG	endoglin	0.21
PHYHD1	phytanoyl-CoA dioxygenase domain containing 1	0.21
MIR4492	microRNA 4492	0.21
ETS1	ETS proto-oncogene 1, transcription factor	0.21
SLC38A2	solute carrier family 38 member 2	0.21
TM6SF1	transmembrane 6 superfamily member 1	0.21
RARA	retinoic acid receptor alpha	0.21
TPM4	tropomyosin 4	0.21
MIR3191	microRNA 3191	0.21
DDAH1	dimethylarginine dimethylaminohydrolase 1	0.23
HS1BP3	HCLS1 binding protein 3	0.23
LOC100288911	uncharacterized LOC100288911	0.23
STPG4	sperm-tail PG-rich repeat containing 4	0.23
NCRUPAR	non-protein coding RNA, upstream of F2R/PAR1	0.23
ARMS2	age-related maculopathy susceptibility 2	0.23
FTH1	ferritin heavy chain 1	0.23
DDX47	DEAD-box helicase 47	0.23
NCOR2	nuclear receptor corepressor 2	0.23
RHOJ	ras homolog family member J	0.23
SMG1P3	SMG1 pseudogene 3	0.23
CDH13	cadherin 13	0.23
ITGB3	integrin subunit beta 3	0.23
IGF2BP1	insulin like growth factor 2 mRNA binding protein 1	0.23
CCDC57	coiled-coil domain containing 57	0.23
HAUS7	HAUS augmin like complex subunit 7	0.23
TMEM82	transmembrane protein 82	0.24
BCL10	B-cell CLL/lymphoma 10	0.24
EGLN1	egl-9 family hypoxia inducible factor 1	0.24
RAI14	retinoic acid induced 14	0.24
INTS1	integrator complex subunit 1	0.24
LAMB1	laminin subunit beta 1	0.24
LINC00840	long intergenic non-protein coding RNA 840	0.24
KCTD10	potassium channel tetramerization domain containing 10	0.24
THBS1	thrombospondin 1	0.24
GLIS2	GLIS family zinc finger 2	0.24
SMURF2	SMAD specific E3 ubiquitin protein ligase 2	0.24
PREX1	phosphatidylinositol-3,4,5-trisphosphate dependent Rac exchange factor 1	0.24
SPSB1	splA/ryanodine receptor domain and SOCS box containing 1	0.25
CAPN2	calpain 2	0.25
ARHGAP18	Rho GTPase activating protein 18	0.25
SBF2-AS1	SBF2 antisense RNA 1	0.25
IQCD	IQ motif containing D	0.25
SNX29P2	sorting nexin 29 pseudogene 2	0.25
SPTBN1	spectrin beta, non-erythrocytic 1	0.26
RND3	Rho family GTPase 3	0.26
LRRC8A	leucine rich repeat containing 8 VRAC subunit A	0.26
MICAL2	microtubule associated monooxygenase, calponin and LIM domain containing 2	0.26
MYEOV	myeloma overexpressed	0.26
MT1A	metallothionein 1A	0.26
MAFF	MAF bZIP transcription factor F	0.26
TRIO	trio Rho guanine nucleotide exchange factor	0.28
LINC00963	long intergenic non-protein coding RNA 963	0.28
COL4A2	collagen type IV alpha 2 chain	0.28
IRF2BPL	interferon regulatory factor 2 binding protein like	0.28
GADD45B	growth arrest and DNA damage inducible beta	0.28
BACE2	beta-site APP-cleaving enzyme 2	0.28

C1orf100	chromosome 1 open reading frame 100	0.29
CSRNP1	cysteine and serine rich nuclear protein 1	0.29
SCAMP1	secretory carrier membrane protein 1	0.29
SNORD124	small nucleolar RNA, C/D box 124	0.29
SMIM29	small integral membrane protein 29	0.30
BICD2	BICD cargo adaptor 2	0.30
WBP1L	WW domain binding protein 1 like	0.30
TPP1	tripeptidyl peptidase 1	0.30
NNMT	nicotinamide N-methyltransferase	0.30
ABCC1	ATP binding cassette subfamily C member 1	0.30
PIR-FIGF	PIR-FIGF readthrough	0.30
ERRFI1	ERBB receptor feedback inhibitor 1	0.31
MIR4684	microRNA 4684	0.31
NISCH	nischarin	0.31
FOXP1	forkhead box P1	0.31
MIR3138	microRNA 3138	0.31
JADE2	jade family PHD finger 2	0.31
ZYX	zyxin	0.31
STK24	serine/threonine kinase 24	0.31
ANXA2	annexin A2	0.31
AFAP1	actin filament associated protein 1	0.32
LINC00607	long intergenic non-protein coding RNA 607	0.33
DAP	death associated protein	0.33
ZSWIM6	zinc finger SWIM-type containing 6	0.33
HRH1	histamine receptor H1	0.34
TBC1D2	TBC1 domain family member 2	0.34
ME3	malic enzyme 3	0.34
LYL1	LYL1, basic helix-loop-helix family member	0.34
CCDC97	coiled-coil domain containing 97	0.34
MIR216B	microRNA 216b	0.36
PTPN1	protein tyrosine phosphatase, non-receptor type 1	0.36
TMBIM1	transmembrane BAX inhibitor motif containing 1	0.37
ARMC9	armadillo repeat containing 9	0.37
ACTN1-AS1	ACTN1 antisense RNA 1	0.37
MAP3K14	mitogen-activated protein kinase kinase kinase 14	0.37
TBC1D22A	TBC1 domain family member 22A	0.37
HHIP	hedgehog interacting protein	0.38
NEK6	NIMA related kinase 6	0.38
CCDC81	coiled-coil domain containing 81	0.38
NCOR2	nuclear receptor corepressor 2	0.38
TANC1	tetratricopeptide repeat, ankyrin repeat and coiled-coil containing 1	0.39
PLAT	plasminogen activator, tissue type	0.39
MIR1204	microRNA 1204	0.39
SMG6	SMG6, nonsense mediated mRNA decay factor	0.39
DLC1	DLC1 Rho GTPase activating protein	0.40
TKT	transketolase	0.41
TMEM170B	transmembrane protein 170B	0.41
COL13A1	collagen type XIII alpha 1 chain	0.41
TRAM2	translocation associated membrane protein 2	0.42
NA	NA	0.42
ZNF469	zinc finger protein 469	0.42
ADTRP	androgen dependent TFPI regulating protein	0.43
RALA	RAS like proto-oncogene A	0.44
EHD1	EH domain containing 1	0.44
CLCF1	cardiotrophin like cytokine factor 1	0.44
DNMBP-AS1	DNMBP antisense RNA 1	0.45
C15orf54	chromosome 15 open reading frame 54 (putative)	0.45
KIRREL1	kirre like nephrin family adhesion molecule 1	0.46
TANC1	tetratricopeptide repeat, ankyrin repeat and coiled-coil containing 1	0.46
TMEM65	transmembrane protein 65	0.46
PFKP	phosphofructokinase, platelet	0.46
PLCB3	phospholipase C beta 3	0.46
PTPRE	protein tyrosine phosphatase, receptor type E	0.48
LTBP2	latent transforming growth factor beta binding protein 2	0.48
IGFBP7-AS1	IGFBP7 antisense RNA 1	0.49
MIR4668	microRNA 4668	0.49
MIR21	microRNA 21	0.51
MLLT1	MLLT1, super elongation complex subunit	0.53
BCAR3	BCAR3, NSP family adaptor protein	0.56
EVA1C	eva-1 homolog C	0.56
MIR4305	microRNA 4305	0.57
EFEMP1	EGF containing fibulin extracellular matrix protein 1	0.58

FHL2	four and a half LIM domains 2	0.58
FLRT2	fibronectin leucine rich transmembrane protein 2	0.59
TRERNA1	translation regulatory long non-coding RNA 1	0.59
ZBTB17	zinc finger and BTB domain containing 17	0.62
DPP9	dipeptidyl peptidase 9	0.62
SZT2	SZT2, KICSTOR complex subunit	0.63
F2RL1	F2R like trypsin receptor 1	0.63
FOXL1	forkhead box L1	0.63
PDLIM7	PDZ and LIM domain 7	0.64
SPSB1	splA/ryanodine receptor domain and SOCS box containing 1	0.77
NPLOC4	NPL4 homolog, ubiquitin recognition factor	0.78
PIP5K1P1	phosphatidylinositol-4-phosphate 5-kinase type 1 pseudogene 1	0.84
MIR3658	microRNA 3658	0.85
MMP2	matrix metallopeptidase 2	0.88
TXNDC5	thioredoxin domain containing 5	1.00
		-1.85

Online Table IX: Sequencing and transcriptional data used in this study. Information on the ChIP-seq, DNase-seq and microarray transcriptional datasets publicly accessed or generated.

Experiment	Factor	Cell type	Condition	GEO ID	Lab
**ChIP-seq**	ERG	HUVEC	untreated	GSM3557980 (This paper)	Anna Randi, Imperial College London
**ChIP-seq**	ERG	VCaP	untreated	GSM717395	Edwin Cheung, Genome Institute of Singapore
**ChIP-seq**	ERG	VCaP	untreated	GSM353637	Arul Chinnaiyan, Northwestern University
**DNaseI hypersensitivity**		HUVEC	untreated	GSM816646	Greg Crawford, Duke University
**ChIP-seq**	H3K27ac	HUVEC	untreated	GSM733691	Bradley Bernstein, Broad Institute
**ChIP-seq**	H3K4me1	HUVEC	untreated	GSM733690	Bradley Bernstein, Broad Institute
**ChIP-seq**	H3K27me3	HUVEC	untreated	GSM733688	Bradley Bernstein, Broad Institute
**ChIP-seq**	H3K27ac	VCaP	untreated	GSM1328982	Arul Chinnaiyan, University of Michigan
**ChIP-seq**	H3K4me1	VCaP	untreated	GSM353631	Arul Chinnaiyan, Northwestern University
**ChIP-seq**	H3K27me3	VCaP	untreated	GSM353621	Arul Chinnaiyan, Northwestern University
**ChIP-seq**	H3K27ac	HUVEC	siCtl	GSM3557982 (This paper)	Anna Randi, Imperial College London
**ChIP-seq**	H3K27ac	HUVEC	siERG	GSM3557983 (This paper)	Anna Randi, Imperial College London
**ChIP-seq**	MED1	HUVEC	siCtl	GSM3557984 (This paper)	Anna Randi, Imperial College London
**ChIP-seq**	MED1	HUVEC	siERG	GSM3557985 (This paper)	Anna Randi, Imperial College London
**ChIP-seq**	GATA2	HUVEC	untreated	GSM935347	Peggy Farnham, University of South California
**ChIP-seq**	cFOS	HUVEC	untreated	GSM935585	Peggy Farnham, University of South California
**ChIP-seq**	cJUN	HUVEC	untreated	GSM935278	Peggy Farnham, University of South California
**Microarray**		HUVEC	siCtl v siERG	GSE32984 (GSM816988-GSM816990, GSM816985-GSM816987)	Anna Randi, Imperial College London
**Microarray**		VCaP	siCtl v siERG	GSE53994 (GSM1305158-GSM1305166)	Ralf Kittler, The University of Texas Southwestern
