# Supplementary material for: The Transcription Factor ERG Regulates Super-Enhancers Associated With an Endothelial-Specific Gene Expression Program
Source: Circ Res. 2019 Mar 20;124(9):1337–49. doi: 10.1161/CIRCRESAHA.118.313788 (PMC6493686; doi:10.1161/CIRCRESAHA.118.313788)
Supplement: Supplementary file 2 [file res-124-1337-s002.pdf]

Gemma Bridges-Lyman

From: Joan Ponsà Cobas <joan.ponsa.cobas@gmail.com>
Sent: Thursday, March 14, 2019 9:11 AM
To: circres
Subject: CIRCRES/2018/313788DR2: The transcription factor ERG regulates super-enhancers associated with an endothelial-specific gene expression program

*** **CAUTION:** This email originated from outside of the **American Heart Association**. Do **not** click links or open attachments unless you recognize the sender and know the content is safe. ***

To whom it may concern,

I contact you regarding “CIRCRES/2018/313788DR2: The transcription factor ERG regulates super-enhancers associated with an endothelial-specific gene expression program.” , to inform that I give permission for my name to be acknowledged.

Regards,

Joan
